# Supplementary material for: Risk and response adapted therapy following autologous stem cell transplant in patients with newly diagnosed multiple myeloma (RADAR (UK-MRA Myeloma XV Trial): study protocol for a phase II/III randomised controlled trial
Source: BMJ Open. 2022 Nov 16;12(11):e063037. doi: 10.1136/bmjopen-2022-063037 (PMC9677008; doi:10.1136/bmjopen-2022-063037)
Supplement: Supplementary data [file bmjopen-2022-063037supp001.pdf]

RADAR: Protocol Paper Attachments

Contents

1 Glossary of Terms 2
2 List of Sites in the RADAR Trial 4
3 Spirit Checklist: Reporting checklist for protocol of a clinical trial. 5
4 Excluded Concomitant Medication: 11
5 Exploratory analysis 13
6 Subgroup analysis 14
7 Informed Consent Material 16
Bone Marrow Consent 16
Part A: Initial Treatment and Further Study Consent 19
Part B: Randomisation 1 Treatment Group 22
Part C: Randomisation 2 Treatment Group 24
Part D: Randomisation 3 Treatment Group 26
8 Central Sample Analysis 28
9 References 31

1 Glossary of Terms

AE	Adverse event
ALT	Alanine aminotransferase
ANC	Absolute neutrophil count
AR	Adverse reaction
ASCT	Autologous stem cell transplantation
β2M	Beta2-microglobulin
BD	Twice daily
CI	Chief Investigator
CR	Complete response
CRF	Case report form
CRUK	Cancer Research United Kingdom
CTCAE	Common Terminology Criteria for Adverse Events
CTRU	Clinical Trials Research Unit
Cy	Cyclophosphamide
D	Dexamethasone
DMEC	Data Monitoring and Ethics Committee
DSUR	Developmental Safety Update Report
ECOG	Eastern Cooperative Oncology Group
eCRF	Electronic Case Report Form
EORTC	European Organisation for Research and Treatment of Cancer
FISH	Fluorescence in situ hybridization test
FWER	Family-wise error rate
GCP	Good Clinical Practice
HDM	High-dose melphalan
HMDS	Haematological Malignancy Diagnostic Service
IMiD®	Immunomodulatory Drug
IMP	Investigational medicinal product
IMWG	International Myeloma Working Group
IPD	Individual participant data
Isa	Isatuximab
ISS	International Staging System
ITT	Intention to treat
LFT	Liver function test
MedDRA	Medical Dictionary for Regulatory Activities
mg	Milligram
MGUS	Monoclonal gammopathy of undetermined significance
MHRA	Medicines and Healthcare Products Regulatory Agency
MM	Multiple myeloma
MR	Minimal response
MRD	Minimal Residual Disease
NCRI	National Cancer Research Institute
NDMM	Newly diagnosed multiple myeloma
NICE	National Institute for Health and Care Excellence
OS	Overall survival
PD	Progressive disease
PFS	Progression-free survival

PFS2	Time to second progression
PI	Principal Investigator
PIS	Patient Information Sheet
PP	Paraprotein
PR	Partial response
PS	Performance status
QoL	Quality of life
R	Lenalidomide
R1-3	Randomisation 1-3
RCT	Randomised controlled trials
RDE	Remote Data Entry
REC	Research Ethics Committee
R-ISS	Revised International Staging System
RSI	Reference Safety Information
SC	Sub-cutaneous
SD	Stable disease
SAE	Serious adverse event
SAP	Statistical analysis plan
SAR	Serious adverse reaction
SmPC	Summary of Product Characteristics
SOP	Standard operating procedure
SPM	Secondary Primary Malignancy
SSOP	Study site operating procedure
SUSAR	Suspected unexpected serious adverse reaction
TE	Transplant eligible
TMG	Trial Management Group
TNE	Transplant non-eligible
TSC	Trial Steering Committee
U&Es	Urea & electrolytes
UK-MRA	United Kingdom Myeloma Research Alliance
ULN	Upper limit of normal
VGPR	Very good partial response

2 List of Sites in the RADAR Trial

Aberdeen Royal Infirmary	Royal Bournemouth Hospital	University Hospital Wishaw
Royal Sussex County Hospital	Poole General Hospital	St Richard's Hospital
Chesterfield Royal Hospital	Salisbury District Hospital	Worthing Hospital
Derriford Hospital	Sunderland Royal Hospital	The Christie
Kent & Canterbury Hospital, Canterbury	South Tyneside District Hospital	Cheltenham General Hospital
Queen Elizabeth the Queen Mother Hospital	Southmead Hospital Bristol	Gloucestershire Royal Hospital
William Harvey Hospital	St. Helens	The Clatterbridge Centre
Freeman Hospital	Whiston Hospital	
Queen Elizabeth Hospital Gateshead	St George's Hospital (Tooting)	
Hammersmith Hospital	Torbay Hospital	
Manchester Royal Infirmary	University College London Hospital	
Wythenshawe Hospital	Leicester Royal Infirmary	
Milton Keynes University Hospital	Worcestershire Royal Hospital	
Perth Royal Infirmary	Alexandra Hospital, Redditch	
Ninewells Hospital	York Hospital	
Northampton General Hospital	University Hospital of Wales, Cardiff	
Churchill Hospital	Stratford Hospital	
Horton General Hospital	Warwick Hospital	
Queen Alexandra Hospital, Portsmouth	New Cross Hospital	
Queen Elizabeth Hospital, Birmingham	University Hospital Coventry	
Raigmore Hospital, Inverness	University Hospital Monklands	
Rotherham General Hospital	University Hospital Hairmyres	

3 Spirit Checklist: Reporting checklist for protocol of a clinical trial.

Based on the SPIRIT guidelines.

Chan A-W, Tetzlaff JM, Gøtzsche PC, Altman DG, Mann H, Berlin J, Dickersin K, Hróbjartsson A, Schulz KF, Parulekar WR, Krleža-Jerić K, Laupacis A, Moher D. SPIRIT 2013 Explanation and Elaboration: Guidance for protocols of clinical trials. BMJ. 2013;346:e7586

		Reporting Item	Page Number
**Administrative information**			
Title	#1	Descriptive title identifying the study design, population, interventions, and, if applicable, trial acronym	1
Trial registration	#2a	Trial identifier and registry name. If not yet registered, name of intended registry	1,2,3
Trial registration: data set	#2b	All items from the World Health Organization Trial Registration Data Set	NA
Protocol version	#3	Date and version identifier	29
Funding	#4	Sources and types of financial, material, and other support	32
Roles and responsibilities: contributorship	#5a	Names, affiliations, and roles of protocol contributors	1, 31
Roles and responsibilities: sponsor contact information	#5b	Name and contact information for the trial sponsor	31,32
Roles and responsibilities: sponsor and funder	#5c	Role of study sponsor and funders, if any, in study design; collection, management, analysis, and interpretation of data; writing of the report; and the decision to submit the report for publication, including whether they will have ultimate authority over any of these activities	32
Roles and responsibilities: committees	#5d	Composition, roles, and responsibilities of the coordinating centre, steering committee, endpoint adjudication committee, data management team, and other individuals or groups overseeing the trial, if applicable (see Item 21a for data monitoring committee)	26,27
**Introduction**			
Background and rationale	#6a	Description of research question and justification for undertaking the trial, including summary of relevant	6,7

		studies (published and unpublished) examining benefits and harms for each intervention	
Background and rationale: choice of comparators	#6b	Explanation for choice of comparators	6,7
Objectives	#7	Specific objectives or hypotheses	7, 18
Trial design	#8	Description of trial design including type of trial (eg, parallel group, crossover, factorial, single group), allocation ratio, and framework (eg, superiority, equivalence, non-inferiority, exploratory)	8
**Methods: Participants, interventions, and outcomes**			
Study setting	#9	Description of study settings (eg, community clinic, academic hospital) and list of countries where data will be collected. Reference to where list of study sites can be obtained	8
Eligibility criteria	#10	Inclusion and exclusion criteria for participants. If applicable, eligibility criteria for study centres and individuals who will perform the interventions (eg, surgeons, psychotherapists)	8-14
Interventions: description	#11a	Interventions for each group with sufficient detail to allow replication, including how and when they will be administered	15-17
Interventions: modifications	#11b	Criteria for discontinuing or modifying allocated interventions for a given trial participant (eg, drug dose change in response to harms, participant request, or improving / worsening disease)	17
Interventions: adherance	#11c	Strategies to improve adherence to intervention protocols, and any procedures for monitoring adherence (eg, drug tablet return; laboratory tests)	17
Interventions: concomitant care	#11d	Relevant concomitant care and interventions that are permitted or prohibited during the trial	17
Outcomes	#12	Primary, secondary, and other outcomes, including the specific measurement variable (eg, systolic blood pressure), analysis metric (eg, change from baseline, final value, time to event), method of aggregation (eg, median, proportion), and time point for each outcome. Explanation of the clinical relevance of chosen efficacy and harm outcomes is strongly recommended	18

Participant timeline	#13	Time schedule of enrolment, interventions (including any run-ins and washouts), assessments, and visits for participants. A schematic diagram is highly recommended (see Figure)	18,19
Sample size	#14	Estimated number of participants needed to achieve study objectives and how it was determined, including clinical and statistical assumptions supporting any sample size calculations	20
Recruitment	#15	Strategies for achieving adequate participant enrolment to reach target sample size	23
**Methods: Assignment of interventions (for controlled trials)**			
Allocation: sequence generation	#16a	Method of generating the allocation sequence (eg, computer-generated random numbers), and list of any factors for stratification. To reduce predictability of a random sequence, details of any planned restriction (eg, blocking) should be provided in a separate document that is unavailable to those who enrol participants or assign interventions	23
Allocation concealment mechanism	#16b	Mechanism of implementing the allocation sequence (eg, central telephone; sequentially numbered, opaque, sealed envelopes), describing any steps to conceal the sequence until interventions are assigned	23
Allocation: implementation	#16c	Who will generate the allocation sequence, who will enrol participants, and who will assign participants to interventions	23
Blinding (masking)	#17a	Who will be blinded after assignment to interventions (eg, trial participants, care providers, outcome assessors, data analysts), and how	NA
Blinding (masking): emergency unblinding	#17b	If blinded, circumstances under which unblinding is permissible, and procedure for revealing a participant's allocated intervention during the trial	NA
**Methods: Data collection, management, and analysis**			
Data collection plan	#18a	Plans for assessment and collection of outcome, baseline, and other trial data, including any related processes to promote data quality (eg, duplicate measurements, training of assessors) and a description of study instruments (eg, questionnaires,	23

		laboratory tests) along with their reliability and validity, if known. Reference to where data collection forms can be found, if not in the protocol	
Data collection plan: retention	#18b	Plans to promote participant retention and complete follow-up, including list of any outcome data to be collected for participants who discontinue or deviate from intervention protocols	23
Data management	#19	Plans for data entry, coding, security, and storage, including any related processes to promote data quality (eg, double data entry; range checks for data values). Reference to where details of data management procedures can be found, if not in the protocol	23
Statistics: outcomes	#20a	Statistical methods for analysing primary and secondary outcomes. Reference to where other details of the statistical analysis plan can be found, if not in the protocol	18
Statistics: additional analyses	#20b	Methods for any additional analyses (eg, subgroup and adjusted analyses)	24
Statistics: analysis population and missing data	#20c	Definition of analysis population relating to protocol non-adherence (eg, as randomised analysis), and any statistical methods to handle missing data (eg, multiple imputation)	24
**Methods: Monitoring**			
Data monitoring: formal committee	#21a	Composition of data monitoring committee (DMC); summary of its role and reporting structure; statement of whether it is independent from the sponsor and competing interests; and reference to where further details about its charter can be found, if not in the protocol. Alternatively, an explanation of why a DMC is not needed	27
Data monitoring: interim analysis	#21b	Description of any interim analyses and stopping guidelines, including who will have access to these interim results and make the final decision to terminate the trial	27
Harms	#22	Plans for collecting, assessing, reporting, and managing solicited and spontaneously reported adverse events and other unintended effects of trial interventions or trial conduct	27
Auditing	#23	Frequency and procedures for auditing trial conduct, if any, and whether the process will be independent from investigators and the sponsor	29

**Ethics and dissemination**			
Research ethics approval	#24	Plans for seeking research ethics committee / institutional review board (REC / IRB) approval	29
Protocol amendments	#25	Plans for communicating important protocol modifications (eg, changes to eligibility criteria, outcomes, analyses) to relevant parties (eg, investigators, REC / IRBs, trial participants, trial registries, journals, regulators)	29
Consent or assent	#26a	Who will obtain informed consent or assent from potential trial participants or authorised surrogates, and how (see Item 32)	30
Consent or assent: ancillary studies	#26b	Additional consent provisions for collection and use of participant data and biological specimens in ancillary studies, if applicable	30,31
Confidentiality	#27	How personal information about potential and enrolled participants will be collected, shared, and maintained in order to protect confidentiality before, during, and after the trial	30
Declaration of interests	#28	Financial and other competing interests for principal investigators for the overall trial and each study site	32
Data access	#29	Statement of who will have access to the final trial dataset, and disclosure of contractual agreements that limit such access for investigators	30
Ancillary and post trial care	#30	Provisions, if any, for ancillary and post-trial care, and for compensation to those who suffer harm from trial participation	32
Dissemination policy: trial results	#31a	Plans for investigators and sponsor to communicate trial results to participants, healthcare professionals, the public, and other relevant groups (eg, via publication, reporting in results databases, or other data sharing arrangements), including any publication restrictions	31
Dissemination policy: authorship	#31b	Authorship eligibility guidelines and any intended use of professional writers	31
Dissemination policy: reproducible research	#31c	Plans, if any, for granting public access to the full protocol, participant-level dataset, and statistical code	31
**Appendices**			
Informed consent materials	#32	Model consent form and other related documentation given to participants and authorised surrogates	(Supplementary Material)

Biological specimens	#33	Plans for collection, laboratory evaluation, and storage of biological specimens for genetic or molecular analysis in the current trial and for future use in ancillary studies, if applicable	(Supplementary Material)

None The SPIRIT Explanation and Elaboration paper is distributed under the terms of the Creative Commons Attribution License CC-BY-NC. This checklist can be completed online using https://www.goodreports.org/, a tool made by the EQUATOR Network in collaboration with Penelope.ai

4 Excluded Concomitant Medication:

Excluded concomitant Medication:
1. Systemic treatment with any of the following metabolising enzyme inducers and inhibitors should be avoided, unless there is no appropriate alternative medication for the participant's use (if there were to be a drug-drug interaction with an inducer, cyclophosphamide and bortezomib metabolite exposure would be increased):<br>a. Strong CYP3A inducers: rifampin, rifapentine, rifabutin, carbamazepine, phenytoin, and phenobarbital<br>b. Strong CYP3A inhibitors<br><br>2. Excluded foods and dietary supplements include St. John's Wort, green tea and supplements containing green tea extracts<br><br>3. The following medicinal products and procedures are **prohibited** during this study:<br>a. Any antineoplastic treatment with activity against MM, other than study drugs<br>b. Platelet transfusions within 3 days prior to study drug dosing for any dosing day, or prior to randomisation in order to meet eligibility criteria.<br>c. Additional steroid therapy (not as trial IMP) (except before commencing trial treatment up to a maximum of 160mg dexamethasone equivalent as per inclusion criteria or steroids used to manage hypersensitivity to trial medication)<br>d. Radiotherapy for the treatment of symptoms related to disease progression.<br>e. Continuous use of clarithromycin (short courses of 5-10 days are permitted for treatment of infective episodes when indicated)<br>f. Hormone replacement therapy and erythropoietic agents and other agents that increase the risk of thrombosis (applicable during treatment with lenalidomide)

Quality of Life and Health Economics questionnaires

Timepoints for all participants up to 100 days post-ASCT

- Baseline
- End of induction
- 100 days post-ASCT

***R1 pathway participants**

- After cycles 6, 12, 24 and 36 (for participants randomised to 'stop isatuximab' at R1, the cycle 24 and 36 questionnaires should still be completed at equivalent timepoints)

***Timepoints for R2 and R3 pathway participants receiving R or RIsa**

- After cycles 6, 12 and 24 of maintenance

***Timepoints for R2 and R3 pathway participants receiving RBorD+R or RBorIsaD+RIsa**

- After cycles 3, 9 and 21 of maintenance.

**Note that participants who stop trial treatment should complete QoL at the equivalent time points (unless the participant wishes to withdraw consent for any further data collection)*

5 Exploratory analysis

Exploratory analysis will consider; the effect of post-ASCT consolidation regimens utilising combinations of lenalidomide, bortezomib, dexamethasone, and isatuximab on immune function in the bone marrow of participants following ASCT, using high dimensional characterisation of the immune microenvironment, immune subsets, phenotype and transcriptome; T cell receptor (TCR) repertoire in the bone marrow of participants following ASCT and consolidation and/or maintenance; correlation of baseline immune profile, TCR diversity and function with disease response, including MRD status and timing of disease relapse; ex-vivo functional studies with participant derived samples used to generate explants or immune-competent patient derived xenografts (icPDX); and to review infection and re-infection rates of COVID-19 in participants during induction, post-ASCT and during post-ASCT consolidation and maintenance treatment. All exploratory analysis to be conducted by the trial statisticians will be pre-specified in the statistical analysis plan.

6 Subgroup analysis

Subgroup analysis will be conducted to determine whether a selection of patient characteristics, haematology / local serological results, biochemistry results and cytogenetic/molecular results as well as response assessments (full list below) are prognostic of the endpoints; PFS, PFS2, OS, attainment of ≥VGPR and attainment of MRD negativity, as defined for all randomisations. Note that the subgroups listed under the Response subheading will not be analysed for the last two endpoints.

Patient Characteristics

- Age (years) at trial registration (≤65, >65)
- Sex (Male / Female)
- ECOG performance status at trial registration (0, 1, ≥2)
- ISS at trial registration (Stage I, Stage II, Stage III)
- R-ISS at trial registration (Stage I, Stage II, Stage III)

Haematology / Local Serological Results

- Haemoglobin concentration at trial registration (<100, ≥100 g/L)
- Platelets at trial registration (<150, ≥150 x10^9/L)
- White blood cells at trial registration (< LLN (Lower limit of normal), ≥LLN)
- Neutrophil count at trial registration (<LLN, ≥LLN)
- Lymphocyte count at trial registration (<LLN, ≥LLN)
- Plasma cells (%) in bone marrow at trial registration (<60%, ≥60%)

Biochemistry Results

- β-2 microglobulin concentration at trial registration (<3.5, 3.5–<5.5, ≥5.5 mg/L)
- Serum creatinine concentration at trial registration (<175, ≥175 µmol/L)
- Corrected serum calcium concentration at trial registration (<2.75, ≥2.75 mmol/L)
- Serum albumin at trial registration (<ULN (Upper limit of normal) , ≥ULN)
- Lactate dehydrogenase at trial registration (<ULN, ≥ULN)
- C-Reactive protein at trial registration (<ULN, ≥ULN)
- Serum bilirubin at trial registration (<ULN, ≥ULN)
- ALT/AST at trial registration (<ULN, ≥ULN)

Cytogenetic/molecular Results

- t(4,14) at trial registration (detected, not detected)
- t(14,16) at trial registration (detected, not detected)
- t(14,20) at trial registration (detected, not detected)
- del(17p) at trial registration (detected, not detected)
- 1q gain at trial registration (detected, not detected)
- Hyperdiploidy at trial registration (detected, not detected)
- The number of high-risk adverse lesions at trial registration (R1 and R2: 0, 1 unable to determine, R3: 2, ≥3, or unable to determine)

Response

- Response to RCyBorD induction (<VGPR, ≥ VGPR)
- Response at 100 days post-ASCT (<VGPR, ≥ VGPR)
- Response at the end of consolidation (<VGPR, ≥ VGPR)
- Response to 12m post-ASCT treatment (<VGPR, ≥ VGPR)
- MRD response to RCyBorD induction (R3 Only: MRD positive, MRD Negative)
- MRD at 100 days post-ASCT (R3 Only: MRD positive, MRD negative)
- MRD response to 6m post-ASCT treatment (R3 Only: MRD positive, MRD Negative)
- MRD response to 12m post-ASCT treatment (R3 Only MRD positive, MRD Negative)

Further subgroup analyses may be undertaken, as appropriate and all the subgroups will be fully defined within the statistical analysis plan according to the current criteria [1-3].

7 Informed Consent Material

Bone Marrow Consent

Delete this line, then print on Trust/Hospital headed paper

Participant ID:	Initials:
Date of Birth:	NHS or CHI number for Scotland/Hospital Number :
EudraCT Number:	Principal Investigator:

CANCER RESEARCH UK

RADAR (UK-MRA Myeloma XV)

INFORMED CONSENT DOCUMENT FOR BONE MARROW AND BLOOD SAMPLE

Please initial each box

1. I confirm that I have read and understand the information sheet for the bone marrow sample request and have had the opportunity to ask questions. ☐
2. I understand that my providing bone marrow samples is voluntary and that I am free to withdraw at any time without my medical care or legal rights being affected. ☐
3. I understand that the samples will be sent along with information about me, which will include NHS number or CHI number for Scotland and may include my name. ☐
4. I agree to these samples being stored and used for research investigations that form part of the RADAR study, and understand that this will include genetic research (for example, analysis of DNA). ☐

5. I understand that a unique reference number will be allocated to the samples which may allow them to be linked back to me in future for research purposes.
6. I understand that my data may be shared on a collaborative basis with researchers in the UK and potentially, centres abroad.
7. I agree to allow any information provided to be used for healthcare and/or further medical research upon the understanding that my identity will remain anonymous wherever possible.
8. I understand that results of my laboratory tests may be shared with other research teams if I am taking part in another clinical study being run by the University of Leeds.
9. I agree to a copy of this Consent Form being sent to the Clinical Trials Research Unit.
10. I agree for my details (which will include date of birth and NHS number or CHI number for Scotland) to be submitted to NHS Digital or other central UK NHS bodies so that information about my health, and mortality should this become available, may be obtained by the CTRU if necessary.
11. I agree to provide bone marrow samples.

Please indicate your wishes in the below scenarios:

Please initial

A) If I am diagnosed with a plasma cell dyscrasia, other than myeloma, I give permission for the samples sent to the central laboratories to be stored and used in future research that receives ethical approval. I understand that the samples and data collected from them may be shared with researchers, possibly in other countries.

Yes No

B) If I am diagnosed with myeloma but do not take part in the RADAR study, I give permission for the samples sent to the central laboratories to be stored and used in future research that receives ethical approval. I understand that the samples and data collected from them may be shared with researchers, possibly outside the EEA.

Yes No

Patient (to be completed by the patient):

Signature…….

Name (block capitals)………………………………………………………………………………………………….

Date…….

Investigator:

I have explained the request to the above named patient and he/she has indicated his/her willingness for samples of bone marrow to be sent to the RADAR laboratories.

Signature……

Name (block capitals)……………………………………………………………………………………………….

Date………

(If used)Translator:

Signature……

Name (block capitals)……………………………………………………………………………………………….

Date………

(1 copy for patient; 1 for the CTRU; 1 held in patient notes, original stored in Investigator Site File)

Part A: Initial Treatment and Further Study Consent

Delete this line, then print on Trust/Hospital headed paper

Participant ID:	Initials:
Date of Birth:	NHS or CHI number for Scotland/Hospital Number:
EudraCT Number:	Principal Investigator:

CANCER RESEARCH UK

RADAR (UK-MRA Myeloma XV)

INFORMED CONSENT DOCUMENT PART A

Initial Treatment and Further Study Information

PLEASE INITIAL EACH BOX

12. I confirm that I have read and understand the Part A information document for the RADAR study and have had the opportunity to ask questions. ☐

13. I understand that my participation in this study is voluntary and that I am free to withdraw at any time without my medical care or legal rights being affected. I understand that even if I withdraw from the above study, the data and samples collected from me will be used in analysing the results of the study, and in some cases further information about any unwanted effects of my treatment may need to be collected by the study team. ☐

14. I understand that my healthcare records may be looked at by authorised individuals from the study team, regulatory bodies or Sponsor in order to check that the study is being carried out correctly. ☐

15. I am aware that I am required to have samples taken and sent to laboratories and I understand that this will include genetic research (for example, analysis of DNA). I understand that the samples will be sent along with information about me, which will include NHS number, or CHI number for Scotland, and may include my name. ☐

16. I understand that results or reports from imaging scans, blood tests or other investigations (including for additional cancers) may be sent to the Clinical Trials Research Unit for central review by the research team. ☐

17. I agree to allow any information or results arising from this study to be used for healthcare and/or further medical research upon the understanding that my identity will remain anonymous wherever possible. ☐

18. I understand that my data and results of my laboratory tests (blood, urine and bone marrow sample collections) may be shared with other research teams if I am taking part in another clinical study being run by the University of Leeds. ☐

19. I agree for my details (which will include date of birth and NHS number or CHI number for Scotland) to be submitted to NHS Digital or other central UK NHS bodies so that information about my health may be obtained by the CTRU if necessary. ☐

20. I understand that my GP, or any other doctor treating me, will be notified of my participation in this study. ☐

21. I understand that some of my data may be passed to other organisations, such as the pharmaceutical companies Sanofi and Celgene, (possibly in other countries where the data protection standards and laws may be different from the UK) to monitor the safety of the treatment(s) that I am receiving. I understand that my identity will remain anonymous. ☐

22. I agree to a copy of this Consent Form being sent to the Clinical Trials Research Unit. ☐

23. I agree to take part in the study. ☐

The following parts of the study are OPTIONAL (see section 3 of the Participant Information Sheet). Even if you agree to take part in the RADAR study, you do not have to agree to these optional parts. Please initial yes or no.

Please initial

A) I agree to take part in the Quality of Life and Healthcare Resource Use studies by completing questionnaires at timepoints throughout the study.

Yes ☐ No ☐

B) I give permission for samples that I provide to be used in future research that receives ethical approval, and I understand that this includes the bone marrow samples provided at the time of my diagnosis (if I consented separately to collection of the diagnostic bone marrow sample in anticipation of participation in this study). I understand that my samples and data collected from them may be shared on a collaborative basis with researchers in the UK and, potentially, centres abroad.

Yes ☐ No ☐

Patient (to be completed by the patient):

I agree to take part in this study.

Signature…….

Name (block capitals)……………………………………………………………………………………………………….

Date……..

Investigator:

I have explained the study to the above named patient, and he/she has indicated his/her willingness to participate in this study.

Signature……

Name (block capitals)……………………………………………………………………………………………………….

Date……

(If used) Translator:

Signature……

Name (block capitals)……………………………………………………………………………………………………….

Date……

(1 copy for patient; 1 for the CTRU; 1 held in patient notes, original stored in Investigator Site File)

Part B: Randomisation 1 Treatment Group

Delete this line, then print on Trust/Hospital headed paper

Participant ID:	Initials:
Date of Birth:	NHS or CHI number for Scotland/Hospital Number:
EudraCT Number:	Principal Investigator:

CANCER RESEARCH UK

RADAR (UK-MRA Myeloma XV)

INFORMED CONSENT DOCUMENT PART B

R1 Treatment Group

PLEASE INITIAL EACH BOX

24. I confirm that I have read and understand the Part B information document for the RADAR study and have had the opportunity to ask questions. ☐

25. I agree to continue my participation in the study as part of the R1 Treatment Group. ☐

Patient (to be completed by the patient):

I agree to take part in this study.

Signature……

Name (block capitals)……………………………………………………………………………………………………

Date………

Investigator:

I have explained the study to the above named patient, and he/she has indicated his/her willingness to participate in this study.

Signature……

Name (block capitals)……………………………………………………………………………………………………

Date………

(If used) Translator:

Signature…………………………………………..………………………………………………………………………

Name (block capitals)…………………………………………….……………………………………………………..

Date…………………………………………………..……………………………………………………………………

(1 copy for patient; 1 for the CTRU; 1 held in patient notes, original stored in Investigator Site File)

Part C: Randomisation 2 Treatment Group

Delete this line, then print on Trust/Hospital headed paper

Participant ID:	Initials:
Date of Birth:	NHS or CHI number for Scotland/Hospital Number:
EudraCT Number:	Principal Investigator:

CANCER RESEARCH UK

RADAR (UK-MRA Myeloma XV)

INFORMED CONSENT DOCUMENT PART C

R2 Treatment Group

PLEASE INITIAL EACH BOX

26. I confirm that I have read and understand the Part C information sheet for the RADAR study and have had the opportunity to ask questions. ☐

27. I agree to continue my participation in the study as part of the R2 Treatment Group. ☐

Patient (to be completed by the patient):

I agree to take part in this study.

Signature………

Name (block capitals)……………………………………………………………………………………………

Date……….....

Investigator:

I have explained the study to the above named patient, and he/she has indicated his/her willingness to participate in this study.

Signature………

Name (block capitals)……………………………………………………………………………………………

Date……

(If used) Translator:

Signature………

Name (block capitals)……………………………………………………………………………………………….

Date……

(1 copy for patient; 1 for the CTRU; 1 held in patient notes, original stored in Investigator Site File)

Part D: Randomisation 3 Treatment Group

Delete this line, then print on Trust/Hospital headed paper

Participant ID:	Initials:
Date of Birth:	NHS or CHI number for Scotland/Hospital Number:
EudraCT Number:	Principal Investigator:

CANCER RESEARCH UK

RADAR (UK-MRA Myeloma XV)

INFORMED CONSENT DOCUMENT PART D

R3 Treatment Group

PLEASE INITIAL EACH BOX

28. I confirm that I have read and understand the Part D information sheet for the RADAR study and have had the opportunity to ask questions. ☐

29. I agree to continue my participation in the study as part of the R3 Treatment Group. ☐

Patient (to be completed by the patient):

I agree to take part in this study.

Signature……….

Name (block capitals)……………………………………………………………………………………………………….

Date………..

Investigator:

I have explained the study to the above named patient, and he/she has indicated his/her willingness to participate in this study.

Signature………

Name (block capitals)……………………………………………………………………………………………………….

Date……….

(If used) Translator:

Signature……………………………………………...………………………………………………………………

Name (block capitals)……………………………………………………………………………………………….

Date…………………………………………………..………………………………………………………………

(1 copy for patient; 1 for the CTRU; 1 held in patient notes, original stored in Investigator Site File)

8 Central Sample Analysis

The trial has 3 central laboratories receiving samples for trial purposes. The time points and the reason for analysis for each central sample can be found in Figure 2. Details on the analysis of each sample is below. Note that participants can consent for their samples to be used in future research and hence each laboratory can store their trial samples for use in future studies. The storage details are also included below.

<u>Birmingham</u>

The Birmingham University Clinical Immunology Laboratory holds UKAS ISO 15189:2012 - Medical Laboratories accreditation. The Trial-related Laboratory Work will be performed using standard assays to the same quality standard as during routine clinical care. The Laboratory will be responsible for the analysis of whole clotted blood, serum and urine collected from participants of the Research Project in accordance with the Protocol. The laboratory will measure disease response and Disease progression, defined according to the IMWG 2016 response criteria for Multiple Myeloma [21 26-28]. This will include assessment of prognostic markers, markers of disease activity and M-protein levels. Analyses will include serum levels of beta-2 microglobulin, immunoglobulins IgG, IgA and IgM, kappa and lambda free immunoglobulin light chains. Serum electrophoresis and immunofixation, M-protein quantitation including total protein and densitometry. Urine immunofixation and free light chain quantitation will be performed where appropriate. As the trial proceeds new biomarkers of myeloma disease activity are likely to become available and will also be measured. The Laboratory will ensure that the Materials are received, handled, analysed, stored and destroyed in line with the individual consent provided.

<u>HMDS</u>

The presence of minimal residual disease will be assessed in bone marrow aspirates using a validated flow cytometry assay (sensitivity ≤0.001%) performed at a single central laboratory (HMDS, Leeds Teaching Hospitals Trust). A minimum of 2,500,000 cells will be evaluated with an eight-colour antibody combination of CD138, CD38, CD45, CD19, CD56, CD27, CD81 and CD117. Additional antigens for detection of CD38-negative plasma cells will be utilised as required. % neoplastic plasma cells and % normal plasma cells as a percentage of leucocytes will be reported. Limit of detection (LoD) and limit of quantification (LoQ) will also be calculated for each sample.
Samples will be used for trial purposes only and will not be stored for future research.

UCL

Bone marrow aspirates, peripheral blood and bone marrow trephine processing within UCL Myeloma Lab for RADAR clinical trial

OBJECTIVES

To provide molecular and genetic data on bone marrow immune profiles of newly diagnosed myeloma patients prior and post autologous stem cell transplantation. This will also be analysed between study arms to compare how different treatment regimes alert immune landscape in patients in particular anti-CD38 mAb Isatuximab.

METHODS

BMA and PB:

1. Sites are requested to collect samples and ship as per Lab Manual together with completed sample request form.

2. All samples will be processed in Myeloma Lab at UCL Cancer Institute.

3. UCL ECMC GCLP Facility will serve as storage facility for the following materials: PB plasma, BM serum, pellets (BM, PB, CD138+ and CD138-), protein lysates, extracted RNA and DNA. All material to be stored at -80˚C.

4. Myeloma Lab will serve as processing facility for all samples and storing of the following materials: BM MNCs (mononuclear cells), PB MNCs, CD138+ cells and CD138- cells. All material to be stored in liquid nitrogen.

5. Both -80˚C freezers and liquid nitrogen tanks are temperature monitored with systems that alert when temperature is out of acceptable range.

6. All samples and material extracted will be logged into the trial specific lab book and database with a unique trial ID, lab ID and time-point.

7. Upon arrival bone marrow aspirates and peripheral blood will be centrifuged. Serum (from BMA) and plasma (from PB) will be collected, aliquoted into 1-1.5ml aliquots and frozen at -80˚C.

8. BMA and PB mononuclear cells will then be isolated and enriched using Ficoll density gradient centrifugation.

9. PB MNCs will then be frozen as pellets and/or cryopreserved.

10. Depending on the tumour burden in the BM MNCs samples will be either frozen as MNCs pellets and/or cryopreserved or tumour cells will be isolated.

11. Tumour will be isolated via column-based MACS cell positive selection using CD138 magnetic beads (MicroBeads).

12. Depending on the number of cells isolated, CD138+ plasma cells will be frozen and stored as pellets and/or protein lysates and/or cryopreserved.

13. CD138- cells will be frozen and stored as pellets and/or cryopreserved.

Bone marrow trephine:

1. Sites are requested to collect, process and ship samples as per Lab Manual together with completed sample request form.

2. Myeloma lab will serve as storage facility for bone marrow trephine slides.

3. Upon arrival all samples will be logged into trial specific database with a unique trial ID, lab ID and time-point.

4. Upon arrival slides will be labelled and stored in the air-tight slide storage box.

ANALYSIS

1. High-dimensional flow cytometry will be used to profile immune context of patient bone marrow mononuclear cells prior and post-transplant.

2. Selected samples will be analysed further using transcriptomic profiling, T cell receptor (TCR) sequencing or single cell sequencing and multiplex immunofluorescence/immunohistochemistry on bone marrow trephine biopsies.

9 References

1. Oxman, A.D., *Subgroup analyses.* BMJ, 2012. **344**.
2. Sun, X., et al., *Is a subgroup effect believable? Updating criteria to evaluate* the *credibility of subgroup analyses.* BMJ, 2010. **340**.
3. Sun, X., et al., *Credibility of claims of subgroup effects in randomised controlled trials: systematic review.* BMJ, 2012. **344**.
