# Supplementary material for: Risk and response adapted therapy following autologous stem cell transplant in patients with newly diagnosed multiple myeloma (RADAR (UK-MRA Myeloma XV Trial): study protocol for a phase II/III randomised controlled trial
Source: BMJ Open. 2022 Nov 16;12(11):e063037. doi: 10.1136/bmjopen-2022-063037 (PMC9677008; doi:10.1136/bmjopen-2022-063037)
Supplement: Author's manuscript [file bmjopen-2022-063037.draft_revisions.pdf]

BMJ Open is committed to open peer review. As part of this commitment we make the peer review history of every article we publish publicly available.

When an article is published we post the peer reviewers' comments and the authors' responses online. We also post the versions of the paper that were used during peer review. These are the versions that the peer review comments apply to.

The versions of the paper that follow are the versions that were submitted during the peer review process. They are not the versions of record or the final published versions. They should not be cited or distributed as the published version of this manuscript.

BMJ Open is an open access journal and the full, final, typeset and author-corrected version of record of the manuscript is available on our site with no access controls, subscription charges or pay-per-view fees (http://bmjopen.bmj.com).

If you have any questions on BMJ Open's open peer review process please email info.bmjopen@bmj.com

Risk and response adapted therapy following autologous stem cell transplant in patients with newly diagnosed multiple myeloma (RADAR (UK-MRA Myeloma XV Trial): study protocol for a Phase II/III randomised controlled trial

Journal:	*BMJ Open*
Manuscript ID	bmjopen-2022-063037
Article Type:	Protocol
Date Submitted by the Author:	22-Mar-2022

Complete List of Authors:	Royle, Kara-Louise; University of Leeds Clinical Trials Research Unit<br>Coulson, Amy; University of Leeds Clinical Trials Research Unit<br>Ramasamy, Karthik; Oxford University Hospitals NHS Trust, Haematology; Oxford University, Radcliffe Department of Medicine<br>Cairns, David; University of Leeds Clinical Trials Research Unit<br>Hockaday, Anna; University of Leeds Clinical Trials Research Unit<br>Quezada, Sergio; UCL Cancer Institute<br>Drayson, Mark ; University of Birmingham<br>Kaiser, Martin; Institute of Cancer Research<br>Owen, Roger; St James's University Hospital, HMDS<br>Auner, Holger; Imperial College London, Department of Immunology and Inflammation; Imperial College London, Langmuir Centre for Myeloma Research<br>Cook, Gordon; University of Leeds Clinical Trials Research Unit; St James's University Hospital, Leeds Cancer Centre<br>Meads, David; University of Leeds, Leeds Institute of Health Sciences<br>Olivier, Catherine; University of Leeds Clinical Trials Research Unit<br>Barnard, Lorna; University of Leeds Clinical Trials Research Unit<br>Paterson, Andrea; University of Leeds Clinical Trials Research Unit<br>Lambkin, Rhiannon; University of Leeds Clinical Trials Research Unit<br>Dawkins, Bryony ; University of Leeds Leeds Institute of Health Sciences, Academic Unit of Health Economics<br>Chapman, Mike; University of Cambridge, Department of Haematology<br>Pratt, Guy; Queen Elizabeth Hospital<br>Popat, Rakesh; University College London Hospitals NHS Foundation Trust<br>Jackson, Graham; Leeds Teaching Hospitals NHS Trust, Department of Haematology<br>Bygrave, Ceri; University Hospital of Wales<br>Sive, Jonathan; University College London Hospitals NHS Foundation Trust<br>de Tute, Ruth; St James's University Hospital, HMDS<br>Chantry, Andrew; Royal Hallamshire Hospital, Department of Haematology<br>Parrish, Christopher; Leeds Teaching Hospitals NHS Trust, Haematology<br>Cook, Mark; University of Birmingham Institute of Cancer and Genomic Sciences; Bristol Myers Squibb

	Asher, Samir; UCL Cancer Institute<br>Yong, Kwee; UCL Cancer Institute
Keywords:	Myeloma < ONCOLOGY, Myeloma < HAEMATOLOGY, Clinical trials < THERAPEUTICS, Cancer genetics < GENETICS, STATISTICS & RESEARCH METHODS, Protocols & guidelines < HEALTH SERVICES ADMINISTRATION & MANAGEMENT

Risk and response adapted therapy following autologous stem cell transplant in patients with newly diagnosed multiple myeloma (RADAR (UK-MRA Myeloma XV Trial): study protocol for a Phase II/III randomised controlled trial

Kara-Louise Royle[1], Amy Beth Coulson[1], Karthik Ramasamy[2], David A Cairns[1], Anna Hockaday[1], Sergio Quezada[3], Mark Drayson[4], Martin Kaiser[5], Roger Owen[6], Holger W. Auner[7], Gordon Cook[1,8], David Meads[19], Catherine Olivier[1], Lorna Barnard[1], Rhiannon Lambkin[1], Andrea Paterson[1], Bryony Dawkins[19], Mike Chapman[9], Guy Pratt[10], Rakesh Popat[11], Graham Jackson[12], Ceri Bygrave[13], Jonathan Sive[11], Ruth de Tute[6], Andrew Chantry[14], Chris Parrish[1,16,] Mark Cook[16,17], Samir Asher[18], Kwee Yong [18]

1. Clinical Trials Research Unit, Leeds Institute of Clinical Trials Research, University of Leeds, Leeds, United Kingdom
2. Oxford University Hospitals NHS Foundation Trust, Radcliffe Department of Medicine, Oxford University
3. Cancer Immunotherapy Group, UCL Cancer Institute, 72 Huntley Street, London WC1E 6BT
4. Clinical Immunology Service, Institute of Immunology and Immunotherapy, Medical School, University of Birmingham, Edgbaston, Birmingham, B15 2TT
5. The Institute of Cancer Research (ICR), Centre for Myeloma Research, Division of Molecular Pathology, Brookes Lawley Building, 15 Cotswold Road, London, Surrey, SM2 5NG
6. HMDS, St James's University Hospital, Leeds, LS9 7TF
7. Centre for Haematology, Imperial College London, London, W12 0NN
8. Leeds Cancer Centre, St James's University Hospital, Leeds, United Kingdom
9. Department of Haematology, University of Cambridge, Cambridge, CB2 0XY
10. Department of Haematology; Queen Elizabeth Hospital, Birmingham, B15 2TH
11. University College London Hospitals NHS Foundation Trust, NW1 2BU
12. Northern Centre for Cancer Care, Freeman Hospital, Newcastle-Upon-Tyne, NE7 7DN
13. University Hospital of Wales, Heath Park, Cardiff, CF14 4XW
14. Department of Haematology, Royal Hallamshire Hospital, Sheffield, S10 2RX
15. Leeds Teaching Hospitals NHS Trust, St James's Institute of Oncology, Beckett Street, Leeds, LS9 7TF, UK
16. University of Birmingham Institute of Cancer and Genomic Sciences
17. Bristol Myers Squibb, Boudry Switzerland
18. UCL Cancer Institute, London, WC1E 6BT
19. Academic Unit of Health Economics, Leeds Institute of Health Sciences, University of Leeds, Leeds, United Kingdom

*corresponding author

Miss Kara-Louise Royle,

Clinical Trials Research Unit, Leeds Institute of Clinical Trials Research, University of Leeds, Leeds, United Kingdom, LS2 9JT

Tel: 0113 343 8366

For peer review only - http://bmjopen.bmj.com/site/about/guidelines.xhtml

For peer review only

Email: k.l.royle@leeds.ac.uk

Word count: 8343

For peer review only - http://bmjopen.bmj.com/site/about/guidelines.xhtml

Abstract

Introduction

Multiple myeloma is a plasma cell malignancy that accounts for 1-2% of newly diagnosed cancers. At diagnosis, approximately 20% of patients can be identified, using cytogenetics, to have inferior survival (high-risk). Additionally, standard-risk patients, with detectable disease (minimal residual disease (MRD)-positive) post-autologus stem cell transplant (ASCT), fare worse compared to those who do not (MRD-negative). Research is required to determine whether a risk-adapted approach post-ASCT could further improve patient outcomes.

Methods

RADAR is a UK, multi-centre, risk-adapted, response-guided, open-label, randomised controlled trial for transplant-eligible newly diagnosed multiple myeloma patients, using combinations of lenalidomide (R), cyclophosphamide (Cy), bortezomib (Bor), dexamethasone (D) and isatuximab (Isa).

Participants receive RCyBorD(x4) induction therapy, followed by high-dose melphalan and ASCT. Post-ASCT, there are three pathways.

1. A phase III discontinuation design to assess de-escalating therapy in standard-risk MRD-negative patients. Participants receive 12 cycles of Isa maintenance. Those who remain MRD-negative are randomised to either continue or stop treatment.
2. A phase II/III multi-arm multi-stage design to test treatment strategies for treatment escalation in standard-risk MRD-positive patients. Participants are randomised to either; R, RBorD(x4) +R, RIsa, or RBorIsaD(x4) + RIsa.
3. A phase II design to assess the activity of intensive treatment strategies in high-risk patients. Participants are randomised to RBorD(x4) +R or RBorIsaD(x4) + RIsa.

1400 participants will be registered to allow for 500, 450 and 172 participants in each pathway. Randomisations are equal and treatment is given until disease progression or intolerance.

Ethics and dissemination

Ethical approval was granted by the London–Central Research Ethics Committee (20/LO/0238) and capacity and capability confirmed by the appropriate local research and development department for each participating centre prior to opening recruitment. Participant informed consent is required before trial registration and re-confirmed post-ASCT. Results will be disseminated by conference presentations and peer-reviewed publications.

Trial registration

ISCRTN 46841867

Key Words: Newly Diagnosed, Multiple Myeloma, Transplant-eligible, Risk-adapted, Treatment escalation, Treatment de-escalation

For peer review only - http://bmjopen.bmj.com/site/about/guidelines.xhtml

Article Summary

Strengths and limitations of the study

- Utilises baseline cytogenetic and dynamic MRD-based stratification within its design
  - The genetic risk platform is based on defined adverse lesions detected by FISH (fluorescence in situ hybridization test): t(4;14), t(14;16), t(14;20), del(17p), gain(1q); thus high-risk patients are defined as having at least two of these adverse lesions. For standard risk patients there is stratification after ASCT, based on MRD status.
- Adaptive
  - New experimental treatment arms are expected to be added into the trial as new evidence emerges. At the time of the trial set-up three experimental treatments have been identified in the standard-risk MRD-positive pathway and two experimental treatment strategies have been identified in the genetic high-risk pathway. The current trial size allows for the addition of at least one further experimental treatment strategy in the MRD-positive pathway which incorporates a multi-arm, multi-stage trial design, if all treatment strategies successfully pass to stage 2. There may be the opportunity to add an additional treatment strategy in the high-risk pathways as evidence emerges.
- Trial addresses an unmet need in treatment
  - The majority of patients will respond to an effective induction regimen followed by ASCT, however the key question in RADAR focusses on post ASCT treatment strategies. Patients are stratified to a pathway depending on genetic risk and MRD status and allocated a treatment pathway specific to their response.
- De-escalation strategy –
  - Patients with standard risk disease and MRD negativity after 1 year of maintenance isatuximab will be randomised to stop treatment. Those patients with sustained MRD negativity during a treatment-free period will be assessed with impacts on quality of life and patient well-being.

Limitations

- Poor responders lost in induction phase
  - Patients achieving <PR to induction therapy will not be able to proceed with on-study ASCT and will have to be treated off -trial
- Excludes patients with impaired renal impairment
  - Patients with a CrCl <30 ml/min following correction of reversible causes will be excluded from the trial
- Definition of risk used in the trial may not be aligned to other international definitions, which may limit generalisability of outcomes

For peer review only - http://bmjopen.bmj.com/site/about/guidelines.xhtml

Introduction

Multiple Myeloma

Multiple myeloma (MM) is the second most common haematological malignancy with over 5500 patients diagnosed in the UK each year.[1] Whilst there is no established cure, survival has extended markedly in the last 15 years such that patients diagnosed today can expect to live a median of 6 years, and 30% will live for 10 years or longer.[1 2]

These advances have been made possible due to the now standard use of “novel agents” including proteasome inhibitors and immunomodulatory agents (IMiD® agents) in conjunction with high-dose melphalan (HDM) supported by autologous stem cell transplantation (ASCT). Whilst MM is predominantly a disease of older people, around 40% of patients are young and fit enough at diagnosis to be eligible for ASCT, a strategy shown in several randomised trials (RCT) to extend progression-free survival (PFS).[2-4]

Improved response rates and increased survival poses challenges for the evaluation of new regimens in a timely manner. Recent studies re-evaluating the role of ASCT in the era of novel agents have confirmed superior PFS in patients allocated to ASCT compared to those allocated to chemotherapy with deferred ASCT.[5] No overall survival (OS) benefit has been seen yet, although this probably reflects the frequent use of salvage ASCT at second remission in most trial patients. Such studies utilise post-ASCT continuous therapy (lenalidomide maintenance) [6] that can extend PFS and OS,[7] but may also impact on quality of life (partly due to treatment-related toxicity), and are not without long-term risk.[8] It is thus imperative to identify those patients who are likely to benefit from extended maintenance therapy, in order to reduce health care costs and minimise toxicity for those who do not benefit.

Existing Evidence: Induction Therapy

Treatment with lenalidomide (R), cyclophosophamide (Cy), bortezomib (Bor) and dexamethasone (D) (RCyBorD) was studied as first line therapy in the Phase 2 EVOLUTION study, where it produced high response rates and deep responses compared to CyBorD or LenBorD.[9]

Existing Evidence: High-Risk Patients

At diagnosis, a small subgroup of patients (approximately 20%) can be identified on the basis of genetic features to have inferior survival regardless of treatment regimen (high-risk patients).[10-12] For such patients, and for those patients with refractory disease (no response to first line therapy), outcomes have remained poor despite the introduction of novel agents[13-15]. Whilst high-risk patients often show a good response to induction therapy, the durability of the response is inferior to standard-risk patients. Using the R-ISS which incorporates high risk cytogenetic features into the the International Staging System (ISS), PFS time is 66 months for R-ISS stage I, 42 months for R-ISS stage II, and 29 months for R-ISS stage III)[16]. The attainment of MRD-negative disease is also critical.[17-19] The activity of the RBorD regimen in patients with high-risk disease looks promising[20], and there is a question of whether an additional benefit may be gained through the use of anti-CD38 immunotherapy. Anti-CD38[21 22] antibodies have single agent activity, but also impressive efficacy in combination, particulary with IMiD agents, lenalidomide and pomalidomide. In the RADAR trial the anti-CD38 antibody isatuximab (Isa) (SAR650984) will be used. Isatuximab is a naked immunoglobulin (Ig) G1 derived

For peer review only - http://bmjopen.bmj.com/site/about/guidelines.xhtml

monoclonal antibody (mAb) that binds selectively to a unqiue epitope on the human surface antigen called CD38 [23].

Existing Evidence: Standard-Risk Patients

With increasingly effective multi-drug regimens being used for induction treatment, and responses further deepened following high-dose therapy and ASCT, the major challenge for standard-risk participants is how to maintain their disease control for as long as possible. The benefit of post-ASCT consolidation approaches remains controversial, with inconsistent evidence identifying both appropriate patient groups and treatment strategies.

Maintenance lenalidomide (R) has been studied within several large studies and has consistently been shown to increase PFS, confimed within a recent meta-analysis.[7] The impact of long-term lenalidomide therapy on bone marrow and immune function, and general wellbeing are not completely understood. Thus there is an urgent need to identify those patients who benefit most from this approach, as well as an evaluation of other agents that utilise the host immune system to improve outcomes, such as antibodies. It is also of benefit to explore the option and effect of stopping maintenance therapy in patients who have ahcieved and maintained the deepest response

Existing Evidence: Use of MRD for stratifying treatment in standard-risk patients

MRD status is a clinically relevant biomarker, and an emerging clinical trial endpoint with recent guidance published by the International Myeloma Working Group (IMWG).[19 24]

Several studies have shown that patients who have detectable disease in their bone marrow (MRD-positive) following ASCT suffer earlier relapses and shorter survival when compared with those who are MRD-negative.[25 26] These results have been combined in a recent meta-analysis.[26] Hence these patients should be the focus of investigative strategies aimed at deepening disease response and converting them from MRD-positive to MRD-negative.

Patients without detectable disease in their bone marrow (MRD-negative), have longer PFS and OS .[27] In the IFM2009 study that randomised patients to receive ASCT upfront or to continue chemotherapy, patients who were MRD-negative fared equally well, regardless of whether they had received ASCT, or chemotherapy alone. This would suggest that those who have achieved MRD-negative response may not need to undergo intensive consolidation therapy, and instead may benefit from a de-escalation of therapy.

Aims and objectives

RADAR aims to address the most important contemporary therapeutic questions for newly diagnosed multiple myeloma transplant eligible (NDMM TE) patients post-ASCT by considering three different post-ASCT treatment pathways:

1. A de-escalation treatment strategy is used to determine whether ceasing Isa maintenance treatment, after 12 cycles of continuous treatment, is non-inferior to long-term Isa treatment, in terms of progression-free survival (PFS) in participants who are standard-risk at diagnosis (participants who have ≤1 high-risk cytogenetic lesion) and are MRD-negative at 100 days post-ASCT and 12 months (cycles) after.

For peer review only - http://bmjopen.bmj.com/site/about/guidelines.xhtml

2. A treatment escalation strategy is used for participants who are standard-risk at diagnosis (participants who have ≤1 high-risk cytogenetic lesion) and MRD-positive at 100 days post ASCT. The trial aims to compare the activity and efficacy of post-ASCT consolidation and maintenance treatment between combinations of R, Bor, D and Isa to R alone in terms of conversion to MRD-negativity.
3. Dose-intensive consolidation and maintenance strategies will be studied for participants who are high-risk at diagnosis (participants who have >1 high-risk cytogenetic lesion). The activity of post-ASCT strategies containing R, Bor, D and Isa will be assessed in terms of PFS rate.

Trial Design

RADAR is a national, multi-centre, risk-adapted, response-guided open-label, randomised controlled trial for NDMM TE patients: phase II/III for standard-risk, MRD-Positive participants, phase III randomised discontinuation design for standard-risk, MRD-Negative participants, phase II randomised for high-risk participants. The following report details the trial protocol and follows the structure of the SPIRIT statement.[28] The SPIRIT checklist can be found within the supplementary material.

Methods:

Setting

The RADAR trial will be conducted at 70 centres in the United Kingdom (See supplementary material), as identified via a feasibility assessment to determine those most appropriate to participate in the trial.

Potential participants will be identified by the research team at the time they are referred to the haematology outpatient department with suspected multiple myeloma. A smaller number of participants may be identified during inpatient admissions. Invitation to participate in the trial and provision of information will be made either during their first consultation, when routine diagnostic tests will be performed and potential treatment options discussed, or at the time they receive their diagnostic test results.

Eligibility criteria

Adults (18 years and older) with previously untreated multiple myeloma requiring therapy, as defined by IMWG diagnostic criteria, who are eligible for stem cell transplant and capable of giving informed consent will be assessed for eligibility. Eligibility will be confirmed prior to; registration, the start of isatuximab maintenance treatment in the Randomisation 1 pathway and each randomisation by the principal investigator (PI) or an appropriately authorised medically trained delegate and will be recorded in the participants' medical records and on the relevant electronic case report form (eCRF).

To be eligible for each point in the trial the participants must meet all the inclusion criteria and none of the exclusion criteria presented in Table 1 for trial registration, Table 2 for isatuximab treatment in the R1 pathway, Table 3 for Randomisation 1 (R1), Table 4 for Randomisation 2 (R2), Table 5 for Randomisation 3 (R3).

For peer review only - http://bmjopen.bmj.com/site/about/guidelines.xhtml

Table 1: Inclusion and Exclusion Criteria for Main Trial Registration

Inclusion Criteria:

1. Previously untreated patients with multiple myeloma requiring therapy, defined as having myeloma defining events or with biomarkers of malignancy according to IMWG diagnostic criteria
2. Eligible for stem cell transplant
3. Eastern Cooperative Oncology Group (ECOG) performance status 0–2 (except in cases where ECOG >2 is due to effects of myeloma eg spinal cord compression);
4. Total bilirubin ≤ 1.5 x upper limit of normal (ULN)
5. Alanine aminotransferase (ALT) and/or aspartate aminotransferase (AST) ≤ 3 x ULN (if ALT **and** AST are tested, both must meet this criteria)
6. Adequate marrow function:
   - neutrophils ≥1.0 × 10^9/L (unless the participant has a known/suspected diagnosis of familial or racial neutropenia in which case an ANC ≥0.75 x 10^9/L is allowed),
   - haemaglobin (Hb) ≥ 80g/L. Blood *transfusions within 3 days prior to eligibility assessments are* ***not*** *permitted*,
   - platelets ≥ 75 × 10^9/L (in the case of heavy bone marrow infiltration (>50%) which is, in the opinion of the investigator, the cause of the thrombocytopaenia and provided appropriate supportive measures and patient monitoring are in place, a platelet count of ≥50 × 10^9/L is permitted. *Platelet transfusions within 3 days prior to eligibility assessments are* ***not*** *permitted.*
7. Creatinine clearance (CrCl) ≥ 30 mL/minute, according to the Cockcroft-Gault formula, following correction of reversible causes (e.g. dehydration, hypercalcaemia, sepsis)
8. Able to swallow oral medication
9. Aged at least 18 years
10. Agree to follow the pregnancy prevention guidelines:

    Female participants who:

    a) Are not of childbearing potential (Appendix 7), OR

    b) If they are of childbearing potential, agree to practice 2 effective methods of contraception, at the same time, from the time of signing the informed consent form until 12 months after the last dose of cyclophosphamide or 5 months after the last dose of study drug, whichever is longest, OR

    c) Agree to practice true abstinence when this is in line with the preferred and usual lifestyle of the subject from the time of signing the informed consent form until 12 months after the last dose of cyclophosphamide or 5 months after the last dose of study drug, whichever is longest. (Periodic abstinence [e.g. calendar, ovulation, symptothermal, post-ovulation methods] and withdrawal are not acceptable methods of contraception.)

    d) Agree to not donate oocytes during the entire study treatment period and until 12 months after the last dose of cyclophosphamide or 5 months after the last dose of study drug, whichever is longest.

    Male participants, even if surgically sterilised (i.e. status post-vasectomy), must agree to one of the following:

    a) Agree to practice effective barrier contraception during the entire study treatment period and until 6 months after the last dose of cyclophosphamide or 5 months after the last dose of study drug, whichever is longest OR

    b) Agree to practice true abstinence when this is in line with the preferred and usual lifestyle of the subject, during the entire study treatment period and until 6 months after the last dose of cyclophosphamide or 5 months after the last dose of study drug, whichever is longest. (Periodic abstinence (e.g. calendar, ovulation, symptothermal, post-ovulation methods] and withdrawal are not acceptable methods of contraception).

    c) Agree to not donate sperm during the entire study treatment period and until 6 months after the last dose of cyclophosphamide or 5 months after the last dose of study drug, whichever is longest.

    Contraception for female and male participants must be in accordance with (and participants must consent to) the Celgene Pregnancy Prevention Plan.

    If female and of childbearing potential, they must have a negative pregnancy test performed by a healthcare professional within 14 days prior to registration.
11. Able to provide written informed consent.

Exclusion Criteria

1. Asymptomatic (smouldering) MM, monoclonal gammopathy of undetermined significance (MGUS), solitary plasmacytoma of bone, or extramedullary plasmacytoma (without evidence of MM)

For peer review only

2. Received previous treatment for MM, with the exception of local radiotherapy to relieve bone pain or spinal cord compression, prior bisphosphonate treatment, or corticosteroids as long as the total dose does not exceed the equivalent of 160mg dexamethasone. *This criteria is not applicable at R1, R2 and R3 when participants will have received previous treatment for MM as part of this trial.*
3. Unstable angina or myocardial infarction within 4 months prior to registration (or at any time since registration for participants starting isatuximab maintenance, R1, R2 and R3), NYHA Class III or IV heart failure, uncontrolled angina, history of severe coronary artery disease, severe uncontrolled ventricular arrhythmias, sick sinus syndrome, or electrocardiographic evidence of acute ischemia or Grade 3 conduction system abnormalities unless subject has a pacemaker
4. Cardiac disorder identified according to local practice (eg left ventricular ejection fraction, LVEF; results from formal measurements acceptable within 28 days prior to registration)
5. Significant neuropathy (Grade ≥3, or Grade 2 with pain)
6. Prior malignancy that required treatment or has shown evidence of recurrence (except for non-melanoma skin cancer or adequately treated cervical carcinoma in situ) during the 5 years prior to registration. Cancer treated with curative intent for >5 years previously and without evidence of recurrence will be allowed.
7. Pregnant, lactating or breastfeeding female participants
8. Known resistance, intolerance or hypersensitivity to any component of the planned therapies, except in the case of hypersensitivity which is amenable to premedication with steroids or H2 blocker. Intolerance includes hereditary problems of galactose intolerance, the Lapp lactase deficiency or glucose-galactose malabsorption.
9. Major surgery within 14 days before registration (or starting isatuximab maintenance, R1, R2 and R3). This would include surgical intervention for relief of cord compression but does not include vertebroplasty or kyphoplasty.
10. Known gastrointestinal (GI) disease or GI procedure that could interfere with the oral absorption or tolerance of trial treatment, including difficulty swallowing.
11. Active systemic infection
12. Participant is hepatitis B surface antigen positive, hepatitis C antibody positive or HIV positive (participants who are hepatitis C core antibody positive but have been successfully treated for the disease may still be eligible – please consult the CTRU/CI). Participants must have hepatitis and HIV screening conducted within 28 days prior to registration.
13. Any other medical or psychiatric condition which, in the opinion of the investigator, contraindicates the participant's participation in this study.
14. Receipt of live vaccination within 30 days prior to registration, for the duration of the study and for 3 months after the last dose of study drug.
15. Participant has risk factors for thromboembolism including the use of agents which may increase their risk of thrombosis, such as hormone replacement therapy (*this exclusion criteria is applicable only at registration and when starting R2 or R3 pathway*)
16. Participant has risk factors for seizures (*this exclusion criteria is applicable only at registration and when starting R2 or R3 pathway*

For peer review only - http://bmjopen.bmj.com/site/about/guidelines.xhtml

Table 2: Inclusion and Exclusion criteria for Isatuximab treatment (R1 Pathway)

**Inclusion Criteria:**
1. Standard-risk (participant is not **confirmed** to have at least two of these genetically adverse lesions: t(4;14), t(14;16), t(14;20), del(17p), gain(1q), as confirmed by the CTRU.<br>2. 4 cycles of RCyBorD received<br>3. MRD-negative (proportion of malignant cells in the bone marrow is < 1 in 100,000, confirmed by HMDS central lab) at 100 days post-ASCT<br>4. Received ≥100mg/m^2 high-dose melphalan and ASCT<br>5. Eastern Cooperative Oncology Group (ECOG) performance status 0–2 (except in cases where ECOG >2 is due to effects of myeloma eg spinal cord compression)<br>6. Total bilirubin <= 1.5 x upper limit of normal (ULN)<br>7. Alanine aminotransferase (ALT) and/or aspartate aminotransferase (AST) ≤ 3 x ULN (if ALT **and** AST are tested, both must meet this criteria)<br>8. Adequate marrow function:<br>a) neutrophils ≥1.0 × 10^9/L (unless the participant has a known/suspected diagnosis of familial or racial neutropenia in which case an ANC ≥0.75 x 10^9/L is allowed),<br>b) Hb≥ 80g/L. Blood transfusions within 3 days prior to eligibility assessments are not permitted,<br>c) platelets ≥ 75 × 10^9/L. Platelet transfusions within 3 days prior to eligibility assessments are not permitted<br>9. Creatinine clearance (CrCl) ≥ 30 mL/minute, according to the Cockcroft-Gault formula, following correction of reversible causes (e.g. dehydration, hypercalcaemia, sepsis)<br>10. Agree to follow the pregnancy prevention guidelines:<br>Female participants who:<br>a) Are not of childbearing potential, OR<br>b) If they are of childbearing potential, agree to practice 2 effective methods of contraception, at the same time, from the time of signing the informed consent form until 12 months after the last dose of cyclophosphamide or until 5 months after the last dose of study drug, whichever is longest OR<br>c) Agree to practice true abstinence when this is in line with the preferred and usual lifestyle of the subject, from the time of signing the informed consent form until 12 months after the last dose of cyclophosphamide or until 5 months after the last dose of study drug, whichever is longest. (Periodic abstinence [e.g. calendar, ovulation, symptothermal, post-ovulation methods] and withdrawal are not acceptable methods of contraception.)<br>d) Agree to not donate oocytes during the entire study treatment period until 12 months after the last dose of cyclophosphamide or 5 months after the last dose of study drug, whichever is longest.<br>Male participants, even if surgically sterilised (i.e. status post-vasectomy), must agree to one of the following:<br>e) Agree to practice effective barrier contraception during the entire study treatment period and until 6 months after the last dose of cyclophosphamide or until 5 months after the last dose of study drug, whichever is longest OR<br>f) Agree to practice true abstinence when this is in line with the preferred and usual lifestyle of the subject during the entire study treatment period and until 6 months after the last dose of cyclophosphamide or until 5 months after the last dose of study drug, whichever is longest.. (Periodic abstinence [e.g. calendar, ovulation, symptothermal, post-ovulation methods] and withdrawal are not acceptable methods of contraception).<br>g) Agree to not donate sperm during the entire study treatment period until 6 months after the last dose of cyclophosphamide or 5 months after the last dose of study drug, whichever is longest.<br>11. Signed the Informed Consent Document for the R1 treatment pathway.<br>a)
**Exclusion Criteria:**
1. Disease progression (according to IMWG criteria)<br>2. MRD-positive (proportion of malignant cells in the bone marrow is ≥ 1 in 100,000, confirmed by HMDS central lab) at 100 days post-ASCT<br>3. Registration exclusion criteria (cardiac risk assessment, HIV and Hepatitis B and C testing do not need to be repeated)

For peer review only - http://bmjopen.bmj.com/site/about/guidelines.xhtml

Table 3: Inclusion and Exclusion criteria for Randomisation 1

Inclusion Criteria:

1. Standard-risk (participant is not **confirmed** to have at least two of these genetically adverse lesions: t(4;14), t(14;16), t(14;20), del(17p), gain(1q), as confirmed by the CTRU.
2. 12 cycles of isatuximab maintenance received
3. MRD-negative (proportion of malignant cells in the bone marrow is < 1 in 100,000, confirmed by HMDS central lab) at 100 days post-ASCT
4. MRD-negative (proportion of malignant cells in the bone marrow is < 1 in 100,000, confirmed by HMDS central lab) after 12 cycles of isatuximab
5. Received ≥100mg/m^2 high-dose melphalan and ASCT
6. Eastern Cooperative Oncology Group (ECOG) performance status 0–2 (except in cases where ECOG >2 is due to effects of myeloma eg spinal cord compression)
7. Total bilirubin <= 1.5 x upper limit of normal (ULN)
8. Alanine aminotransferase (ALT) and/or aspartate aminotransferase (AST) ≤ 3 x ULN (if ALT and AST are tested, both must meet this criteria)
9. Adequate marrow function:
   - neutrophils ≥1.0 × 10^9/L (unless the participant has a known/suspected diagnosis of familial or racial neutropenia in which case an ANC ≥0.75 x 10^9/L is allowed),
   - Hb≥ 80g/L. Blood transfusions within 3 days prior to eligibility assessments are not permitted,
   - platelets ≥ 75 × 10^9/L. Platelet transfusions within 3 days prior to eligibility assessments are not permitted
10. Creatinine clearance (CrCl) ≥ 30 mL/minute, according to the Cockcroft-Gault formula, following correction of reversible causes (e.g. dehydration, hypercalcaemia, sepsis)
11. Agree to follow the pregnancy prevention guidelines:

    Female participants who:

    a) Are not of childbearing potential, OR
    b) If they are of childbearing potential, agree to practice 2 effective methods of contraception, at the same time, from the time of signing the informed consent form until 5 months after the last dose of study drug, OR
    c) Agree to practice true abstinence when this is in line with the preferred and usual lifestyle of the subject from the time of signing the informed consent form until 5 months after the last dose of study drug. (Periodic abstinence [e.g. calendar, ovulation, symptothermal, post-ovulation methods] and withdrawal are not acceptable methods of contraception.)
    d) Agree to not donate oocytes during the entire study treatment period and until 5 months after the last dose of study drug.

    Male participants, even if surgically sterilised (i.e. status post-vasectomy), must agree to one of the following:

    a) Agree to practice effective barrier contraception during the entire study treatment period and until 5 months after the last dose of study drug, OR
    b) Agree to practice true abstinence when this is in line with the preferred and usual lifestyle of the subject during the entire study treatment period and until 5 months after the last dose of study drug. (Periodic abstinence [e.g. calendar, ovulation, symptothermal, post-ovulation methods] and withdrawal are not acceptable methods of contraception).
    c) Agree to not donate sperm during the entire study treatment period and until 5 months after the last dose of study drug.
12. Signed the Informed Consent Document for the R1 treatment pathway.

Exclusion Criteria

1. Disease progression (according to IMWG criteria)
2. MRD-positive (proportion of malignant cells in the bone marrow is ≥ 1 in 100,000, confirmed by HMDS central lab) at 100 days post-ASCT or after 12 cycles of isatuximab
3. Registration exclusion criteria (cardiac risk assessment, HIV and Hepatitis B and C testing do not need to be repeated)

For peer review only - http://bmjopen.bmj.com/site/about/guidelines.xhtml

Table 4: Inclusion and Exclusion Criteria for Randomisation 2

Inclusion Criteria:

1. Standard-risk (participant is not **confirmed** to have at least two of these genetically adverse lesions: t(4;14), t(14;16), t(14;20), del(17p), gain(1q) as confirmed by CTRU.
2. 4 cycles of RCyBorD received
3. MRD-positive (proportion of malignant cells in the bone marrow is ≥ 1 in 100,000, confirmed by HMDS central lab) at 100 days post-ASCT
4. Received ≥100mg/m^2 high-dose melphalan and ASCT
5. Eastern Cooperative Oncology Group (ECOG) performance status 0–2 (except in cases where ECOG >2 is due to effects of myeloma eg spinal cord compression)
6. Total bilirubin <= 1.5 x upper limit of normal (ULN)
7. Alanine aminotransferase (ALT) and/or aspartate aminotransferase (AST) ≤ 3 x ULN (if ALT and AST are tested, both must meet this criteria)
8. Adequate marrow function:
   - neutrophils ≥1.0 × 10^9/L (unless the participant has a known/suspected diagnosis of familial or racial neutropenia in which case an ANC ≥0.75 x 10^9/L is allowed),
   - Hb≥ 80g/L
   - platelets ≥ 75 × 10^9/L. Platelet transfusions within 3 days prior to eligibility assessments are not permitted
9. Creatinine clearance (CrCl) ≥ 30 mL/minute, according to the Cockcroft-Gault formula, following correction of reversible causes (e.g. dehydration, hypercalcaemia, sepsis)
10. Agree to follow the pregnancy prevention guidelines:

    Female participants who:

    a) Are not of childbearing potential, OR
    b) If they are of childbearing potential, agree to practice 2 effective methods of contraception, at the same time, from the time of signing the informed consent form until 12 months after the last dose of cyclophosphamide or 5 months after the last dose of study drug, whichever is longest OR
    c) Agree to practice true abstinence when this is in line with the preferred and usual lifestyle of the subject, from the time of signing the informed consent form until 12 months after the last dose of cyclophosphamide or 5 months after the last dose of study drug, whichever is longest. (Periodic abstinence [e.g. calendar, ovulation, symptothermal, post-ovulation methods] and withdrawal are not acceptable methods of contraception.)
    d) Agree to not donate oocytes during the entire study treatment period and until 12 months after the last dose of cyclophosphamide or 5 months after the last dose of study drug, whichever is longest.

    Male participants, even if surgically sterilised (i.e. status post-vasectomy), must agree to one of the following:

    a) Agree to practice effective barrier contraception during the entire study treatment period and until 6 months after the last dose of cyclophosphamide or 5 months after the last dose of study drug, whichever is longest OR
    b) Agree to practice true abstinence when this is in line with the preferred and usual lifestyle of the subject during the entire study treatment period and until 6 months after the last dose of cyclophosphamide or 5 months after the last dose of study drug, whichever is longest. (Periodic abstinence [e.g. calendar, ovulation, symptothermal, post-ovulation methods] and withdrawal are not acceptable methods of contraception).
    c) Agree to not donate sperm during the entire study treatment period and until until 6 months after the last dose of cyclophosphamide or 5 months after the last dose of study drug, whichever is longest.
11. Signed the Informed Consent Document for the R2 treatment pathway.

Exclusion Criteria:

1. Disease progression (according to IMWG criteria)
2. Registration exclusion criteria (cardiac risk assessment, HIV and Hepatitis B and C testing do not need to be repeated)

For peer review only - http://bmjopen.bmj.com/site/about/guidelines.xhtml

Table 5: Inclusion and Exclusion criteria for Randomisation 3

Inclusion Criteria:

1. High-risk (participant is **confirmed** to have at least two of these genetically adverse lesions: t(4;14), t(14;16), t(14;20), del(17p), gain(1q)) as confirmed by CTRU.
2. 4 cycles of RCyBord received
3. Received ≥100mg/m^2 high-dose melphalan and ASCT
4. Eastern Cooperative Oncology Group (ECOG) performance status 0–2 (except in cases where ECOG >2 is due to effects of myeloma eg spinal cord compression)
5. Total bilirubin <= 1.5 x upper limit of normal (ULN)
6. Alanine aminotransferase (ALT) and/or aspartate aminotransferase (AST) ≤ 3 x ULN (if ALT and AST are tested, both must meet this criteria)
7. Adequate marrow function:
   - neutrophils ≥1.0 × 10^9/L (unless the participant has a known/suspected diagnosis of familial or racial neutropenia in which case an ANC ≥0.75 x 109/L is allowed),
   - Hb≥ 80g/L
   - platelets ≥ 75 × 10^9/L. Platelet transfusions within 3 days prior to eligibility assessments are not permitted
   - Creatinine clearance (CrCl) ≥ 30 mL/minute, according to the Cockcroft-Gault formula, following correction of reversible causes (e.g. dehydration, hypercalcaemia, sepsis)
8. Creatinine clearance (CrCl) ≥ 30 mL/minute, according to the Cockcroft-Gault formula, following correction of reversible causes (e.g. dehydration, hypercalcaemia, sepsis)
9. Agree to follow the pregnancy prevention guidelines:

   Female participants who:

   a) Are not of childbearing potential, OR
   b) If they are of childbearing potential, agree to practice 2 effective methods of contraception, at the same time, from the time of signing the informed consent form until 12 months after the last dose of cyclophosphamide or 5 months after the last dose of study drug, whichever is longest OR
   c) Agree to practice true abstinence when this is in line with the preferred and usual lifestyle of the subject from the time of signing the informed consent form until 12 months after the last dose of cyclophosphamide or 5 months after the last dose of study drug, whichever is longest. (Periodic abstinence [e.g. calendar, ovulation, symptothermal, post-ovulation methods] and withdrawal are not acceptable methods of contraception.)
   d) Agree to not donate oocytes during the entire study treatment period and until 12 months after the last dose of cyclophosphamide or 5 months after the last dose of study drug, whichever is longest.

   Male participants, even if surgically sterilised (i.e. status post-vasectomy), must agree to one of the following:

   a) Agree to practice effective barrier contraception during the entire study treatment period and until 6 months after the last dose of cyclophosphamide or 5 months after the last dose of study drug, whichever is longest OR
   b) Agree to practice true abstinence when this is in line with the preferred and usual lifestyle of the subject during the entire study treatment period and until 6 months after the last dose of cyclophosphamide or 5 months after the last dose of study drug, whichever is longest. (Periodic abstinence [e.g. calendar, ovulation, symptothermal, post-ovulation methods] and withdrawal are not acceptable methods of contraception).
   c) Agree to not donate sperm during the entire study treatment period and until 6 months after the last dose of cyclophosphamide or 5 months after the last dose of study drug, whichever is longest.
10. Signed the Informed Consent Document for the R3 treatment pathway.

Exclusion Criteria

1. Disease progression (according to IMWG criteria)
2. Registration exclusion criteria (cardiac risk assessment, HIV and Hepatitis B and C testing do not need to be repeated)

For peer review only - http://bmjopen.bmj.com/site/about/guidelines.xhtml

Interventions and Dosing

Intervention Schedule

All participants will receive four, 21 day, cycles of induction treatment with RCyBorD. During this time, the genetics risk status of the participant will be analysed and determined from bone marrow samples prior induction treatment (from diagnosis or trial-specific procedures). Participants will either be allocated as high risk (defined as the presence of at least 2 of these adverse lesions: t(4,14), t(14,16(, t(14,20), del(17p) or gain(1q)) or standard risk. At the end of the induction cycle participants will be assessed for response In the absence of disease progression or intolerance, participants with at least a partial response (PR) to will proceed to HDM and ASCT which will be carried out according to local practice. At 100 days post-ASCT participants will be assessed for MRD and response and will follow one of three pathways of post ASCT treatment, provided they meet the eligibility criteria:

1. Participants identified as standard risk and who are MRD-negative post-ASCT will be treated according to the R1 pathway. All participants will receive 12 cycles of Isa maintenance treatment. Those who remain MRD-negative following cycle 12, will be randomised to continue or stop Isa maintenance treatment.
2. Participants identified as standard-risk and who are MRD-positive post-ASCT and have at least a minimal response (MR) at 100 days post ASCT, will proceed to R2. Eligible participants will be randomised between: R maintenance; RBorD consolidation and R maintenance (RBorD + R); RISa maintenance; or RBorIsaD consolidation and RIsa maintenance (RBorIsaD + RIsa).
3. Participants identified as high-risk and have at least a partial response (PR) at 100 days post ASCT will proceed to R3. These participants will be randomised to receive either RBorD consolidation, followed by R maintenance; or RBorIsaD consolidation followed by RIsa maintenance.

All randomisations are balanced across the treatment arms. A computer generated minimisation programme that incorporates a random element will be used to ensure treatment groups are well balanced for the stratification factors of each randomisation (See Table 6).

Table 6: Stratification Factors

Randomisation:	Stratification factors:
1	• Centre<br>• Number of high risk lesions at trial registration (0 or 1 or unable to determine)
2	• Centre<br>• Number of high risk lesions at trial registration (0 or 1 or unable to determine)<br>• Response at 100 days post-ASCT ($< VGPR, \geq VGPR$)
3	• Centre<br>• Number of high risk lesions at trial registration (2, or ≥ 3 , or unable to determine)<br>• Response at 100 days post-ASCT ($< VGPR, \geq VGPR$)

For peer review only

For peer review only - http://bmjopen.bmj.com/site/about/guidelines.xhtml

Table 7 summarises the starting doses for the induction treatment and each post-ASCT treatment pathway. Following ASCT all treatments will continue until disease progression or intolerance. Response will be assessed at the end of each cycle according to the IMWG 2016 response criteria[24 29-31]. Treatment intolerance will be assessed throughout each treatment cycle, according to the national cancer institute (NCI) common terminology criteria for AE (CTCAE) Version 5. All consolidation cycles are 21 days in length, maintenance cycles are 28 days in length.

Table 7: Dosing schedule for RCyBorD induction and each randomisation pathways

Trial Registration- Induction (Each cycle = 21 days)		
**Treatment (Route)**	**Induction – All participants (Starting Dose)**	**Days**
**Bortezomib (SC)**	1.3mg/m^2	1, 8, 15
**Cyclophosphamide (PO)**	500mg	1, 8
**Lenalidomide (PO)**	25mg	1-14
**Dexamethasone (PO)**	40mg	1, 8, 15
**Isatuximab Pre-Randomisation 1 (Each cycle = 28 day)**		
**Treatment (Route)**	**R1 pathway participants Only (Starting Dose)**	**Days**
**Isatuximab (IV)**	10mg/kg	1, 8, 15 and 22 cycle 1 then days 1 and 15 from cycle 2 onwards
**Randomisation 1: Isatuximab maintenance (Each Cycle = 28 days)**		
**Treatment**	**Isatuximab (Days)**	**"Stop Isatuximab" (Days)**
**Isatuximab (IV)**	10mg/kg (Day 1 Only)	None
**Randomisation 2: MRD-positive treatment escalation pathway**		
**Treatment Arm 1 (Control) : Lenalidomide (R) maintenance**		
**Treatment (Route)**	**Starting Dose (Days)**	
**Lenalidomide (PO)**	10mg/kg* (1-21)	
**Treatment Arm 2: RBorD consolidation + R Maintenance**		
**Treatment**	**Consolidation: Cycle 1-4 (each cycle = 21 days)**	**Maintenance (each Cycle = 28 days)**
**Bortezomib (SC)**	1.3mg/m^2 (1, 8, 15)	NA
**Lenalidomide (PO)**	15mg* (1-14)	10mg* (1-21)
**Dexamethasone (PO)**	20mg (1, 8, 15)	NA
**Treatment Arm 3:RISa Maintenance**		
**Treatment**	**Maintenance (Days)**	
**Lenalidomide (PO)**	10mg* (Day 1-21)	
**Isatuximab (IV)**	10mg/kg (1, 8, 15 and 22 cycle 1 then days 1 and 15 from cycle 2 onwards)	
**Treatment Arm 4: RBorIsaD + RIsa**		
Treatment	Consolidation Cycle 1-4: Each cycle = 21 days (Days)	Maintenance: Each cycle = 28 days (Days)
**Bortezomib (SC)**	1.3mg/m^2 (1, 8, 15)	NA
**Lenalidomide (PO)**	15mg* (1-14)	10mg* (1-21)
**Isatuximab (IV)**	10mg/kg (1, 8, 15 cycle 1 then days 1 and 8 cycle 2, then days 1 and 15 cycle 3 onwards)	10mg/kg (1 and 15)
**Dexamethasone (PO)**	20mg (1, 8, 15)	NA
**Randomisation 3: High Risk Treatment Pathway**		
**Treatment Arm 1: RBorD Consolidation and R maintenance**		
**Treatment**	**Consolidation: Cycle 1-4 (each cycle = 21 days)**	**Maintenance (each Cycle = 28 days)**
**Bortezomib (SC)**	1.3mg/m^2 (1, 8, 15)	NA
**Lenalidomide (PO)**	15mg* (1-14)	10mg* (1-21)

For peer review only - http://bmjopen.bmj.com/site/about/guidelines.xhtml

**Dexamethasone (PO)**	20mg (1, 8, 15)	NA
**Treatment Arm 2: RBorIsaD consolidation + RIsa Maintenance**		
**Treatment**	**Consolidation Cycle 1-4: Each cycle = 21 days (Days)**	**Maintenance: Each cycle = 28 days (Days)**
**Bortezomib (SC)**	1.3mg/m^2 (1, 8, 15)	NA
**Lenalidomide (PO)**	15mg* (1-14)	10mg* (1-21)
**Isatuximab (IV)**	10mg/kg (1, 8, 15 cycle 1 then days 1 and 8 cycle 2, then days 1 and 15 cycle 3 onwards)	10mg/kg (1 and 15)
**Dexamethasone (PO)**	20mg (1, 8, 15)	NA

*or final dose administrated at the end of induction or consolidation treatment if lower

Intervention Adherence

Throughout the trial R will be taken orally and swallowed whole at the same time on the scheduled days. D and Cy will be taken orally, whilst Bor will be administrated via a subcutaneous injection. Bortezomib may also be administered at home if this is in line with local policies and if appropriate procedures are in place. Isa will be given as an intravenous infusion and at a facility capable of managing hypersensitivity reactions.

To monitor adherence to the oral trial medicines, participants will complete a participant diary card which will be reviewed at each trial visit. Non-compliance will be reported to the CTRU and any unused capsules will be returned to the pharmacy.

Dose Modifications and Discontinuations

With the exception of Isa, which can only be interrupted and resumed, dose modifications are permitted in the management of toxicities. When the dose of any drug is reduced the dose cannot be re-escalated in this trial.

If one drug in a combination is stopped, treatment with the other drugs in combination can continue. Scenarios in which the participant may remain on trial following consultation with the Chief Investigator (CI) are if they: discontinue two or more drugs during combination therapy; cease treatment for more than three weeks in between any treatment cycle or are delayed in starting maintenance of consolidation beyond 120 days post-ASCT. Note that if Bor, R or D is stopped during the pre-ASCT induction treatment due to toxicity, the participant will only be able to be treated on the standard-risk-MRD-negative pathway (assuming they meet the relevant inclusion criteria).

If treatment is discontinued early, the participant will be treated off trial at the discretion of their treating clinician.

Concomitant medication:

Local support care protocols, including anti-emetic schedules, tumour lysis syndrome prevention, venous thromboembolism prophylaxis and prophylactic antimicrobial therapy should be followed. The excluded concomitant medications and procedures whilst receiving trial treatment are listed in supplementary material.

Concomitant medication, disease and other malignancies will be recorded at eligibility.

For peer review only - http://bmjopen.bmj.com/site/about/guidelines.xhtml

Outcomes:

Primary outcomes

PFS-R1 is defined as the time from R1 to the time of first documented evidence of disease progression or death from any cause. Individuals who are lost to follow-up or progression-free at the time of analysis will be censored at their last known date to be alive and progression-free.

Attainment of MRD-negativity is defined as a binary endpoint. MRD-negativity will be determined at 6 months post-R2 (end of cycle 6 post-ASCT treatment for participants allocated to maintenance only strategies and end of cycle 7 post-ASCT treatment for participants allocated to maintenance and consolidation strategies) according to the IMWG 2016 response criteria.

The PFS rate is defined as the proportion of participants who are alive and progression-free 28 months post-R3.

Disease progression is defined according to the IMWG 2016 response criteria for Multiple Myeloma [24 29-31].

Secondary outcomes:

The secondary outcomes of this trial are to assess; PFS for R2 and R3, Time to progression, Time to second PFS event (PFS2), Overall survival (OS), Survival after progression, Objective response rate (ORR), Attainment of Very Good Partial Response ($\geq$ VGPR), Attainment of MRD negativity, Time to improved response, Time to next treatment, Treatment compliance and total amount of therapy delivered, Toxicity and Safety, including the incidence of second malignancies, Quality of life (QoL) including EORTC-QLQ-C30, EORTC-QLQ-MY20 and EQ-5D-3L and Cost effectiveness.

Exploratory and subgroup analyses

An overview of the planned exploratory and subgroup analysis can be found in the supplementary material. These include genetic and molecular analysis of participant samples conducted by the respective central laboratories, as well as additional analysis to review the infection and re-infection rates of covid-19 in participants during induction, post-ASCT and during post ASCT consolidation.

Participant's timelines

The full trial schema can be seen in Figure 1. The schedule of assessments at each time point are presented in Figure 2.

Trial Entry

Participants will enter into the trial at one of two points in their pathway, this will either be at bone marrow registration or at main trial registration.

Trial Consent

Participants who enter the trial at bone marrow registration will provide consent to have bone marrow samples taken and sent to central laboratories for analysis. If the participant does not have myeloma

For peer review only - http://bmjopen.bmj.com/site/about/guidelines.xhtml

or decides to not take part in RADAR, they will have the option of consenting to their samples being used in future research.

All participants will be required to provide written informed consent for the trial prior to trial registration (for initial trial treatment and further trial information) and prior to starting their post-ASCT treatment pathway once their post-ASCT assessments have been completed (for their pathway specific treatment). Optional consent regarding QoL and healthcare resource use questionnaires and the use of samples for future research will also be obtained within the first consent process.

Consent forms are included in supplementary material.

Trial Registration

Following initial consent, participant eligibility for trial registration will be assessed. Trial samples for blood and urine will be taken for all participants and bone marrow samples will be taken for those who did not enter the trial through bone marrow registration.

Genetic Risk

Bone marrow samples from diagnosis will be analysed at time of diagnosis at local cytogenetics laboratories for FISH-defined high-risk makers. Original/copy of the FISH testing report will also be sent to CTRU where they will be reviewed by the CI/Trial management team for risk stratification. Participants will either be categorised high-risk (defined as the presence of at least 2 of the following adverse lesions: t(4;14), t(4;16), t(14:20), del(17p) or gain(1q)) or standard-risk.

Participants will commence RCyBorD treatment once a diagnosis of symptomatic myeloma has been confirmed by the treating physician, and ideally when it is known that the FISH testing has been successful.

Where risk status can be determined from the first sample participants can continue on the trial. If status cannot be confirmed and the FISH report shows a full test failure i.e. no information relating to adverse lesions is available, a second attempt will be made to collect a bone marrow sample prior to treatment starting. If partial results are obtained from the FISH sample, it may be possible that the CI/ trial management group (TMG) will be able to determine risk status using the assumption that translocations are mutually exclusive and that some markers are rare. If risk status can be determined a second bone marrow sample is not required. However, this must be confirmed by the CTRU.

If, after two bone marrow samples, the FISH results for some or all the adverse lesions are inconclusive participants risk will be determined using a combination of the results from the two samples. The most appropriate pathway will be determined by the CI. Note this may result in the participants stratification factor being 'unable to determine' at randomisation. If a second bone marrow has been requested by CTRU but the collection was not attempted, the participants will be taken off the study (after ASCT, if induction treatment was already started)

Induction and ASCT

Following trial registration, once all central samples have been taken and risk status confirmed, participants will receive induction treatment as described in the intervention section. In the absence

For peer review only - http://bmjopen.bmj.com/site/about/guidelines.xhtml

For peer review only

of disease progression or treatment intolerance participants with at least a partial response (PR) to will proceed to HDM and ASCT.

Consenting participants will complete QoL and resource use questionnaires at the end of RCyBorD induction treatment.

All participants who go through ASCT will have a bone marrow sample taken at 100 days (+/- 14) post-ASCT. If an MRD result cannot be obtained at the first attempt, the bone marrow will be repeated once (within 28 days after 100 days post-ASCT time point). If the second attempt fails, the participant will be considered MRD-positive.

Post-ASCT Treatment

Participants who receive all 4 cycles of induction treatment followed by ASCT will be assessed for eligibility into their assigned randomisation pathways determined by genetic risk and MRD results and treated as described in the intervention schedule.

Consenting participants in the R1 pathway will complete QoL and resource use questionnaires after cycle 6, 12, 24 and 36 (participants randomised to “stop Isa” will complete the cycle 24 and 36 QoL and resource use questionnaires at equivalent time points).

Similarly, consenting participants to receive R or RIsa within pathway R2 and R3 will complete QoL and resource use questionnaires after cycles 6, 12 and 24 of maintenance. Those randomised to receive RBorIsa+R or RBorIsaD+RIsa will complete QoL and resource use questionnaires after cycles 3, 9 and 21 of maintenance.

Trial Follow-up

Participants who discontinue during induction and until 100 days post-ASCT will be followed up for data pertaining to safety, details of salvage treatment, progression (including second progression), and survival at standard care visits. Follow ups will be conducted every 2 months up until disease progression, death or the end of trial. Similar follow-up will be completed for participants who discontinue their allocated trial treatment, which started post-ASCT. In addition consenting participants will still complete QoL and resource use questionnaires at appropriate time points.

All types of adverse event/reaction will be collected until 60 days after the last dose of protocol treatment.

Following disease progression all participants will be followed up annually until death, or until the end of the trial.

Sample size

In total 1400 participants will be recruited into the trial. Accounting for participants who will be lost to follow-up, this allows for 500, 450 and 172 participants to enter each randomisation, respectively.

For peer review only - http://bmjopen.bmj.com/site/about/guidelines.xhtml

For peer review only

Randomisation 1

Data from our previous trial Myeloma XI, suggests that approximately 50 participants will lose their MRD-negative status during the first 12 cycles of Isa maintenance. In addition, Myeloma XI estimated median PFS with continuous R to be 50 months amongst TE participants.[32] Under these assumptions and allowing for our randomisation being 12 months later, 500 participants allows for 80% power to test a non-inferiority margin of 10% at 2 years post-R1 (this equates to a hazard ratio of 1.38) with a one-sided 5% significance level, allowing for approximately 20% further drop-out after R1 amongst participants unwilling to cease treatment or being lost to follow-up.

A power of 80% will be attained when 238 PFS events have been observed. These calculations assume that PFS follow an exponential distribution and that there will be 3 years of recruitment to R1 and 2 further years of follow-up (in order that all participants can have PFS at 2 years post-R1 assessed).

Global Health Status/Quality of Life is considered a key secondary endpoint of R1 as the conclusion that the cessation of continuous treatment is non-inferior should not come at the expense of quality of life. Ignoring those required to account for participants lost to follow-up, the 406 participants recruited above give >95% power to detect a difference of 10 points at 2 years post-R1, where 10 points represents an accepted medium magnitude clinically relevant difference.[33] The calculation uses data collected from the intensive pathway of MRC Myeloma IX to estimate that the standard deviation in the Global Health Status/QoL subscale of EORTC-QLQ-C30 as 27.54 along with a two-sided 5% significance level.[34]

Randomisation 2

R2 is a multi-arm, multi-stage drug/drug-combination assessment platform with seamless phase II (activity) and phase III (efficacy) stages. Each experimental arm is compared with a designated control arm (R maintenance).

Early data from Myeloma XI and similar data from IFM/DFCI 2009 [35] suggest that a 10% upgrade in MRD-negative rate after 6 months continuous R treatment is achievable. It is thought that RBorD consolidation followed by attenuated continuous R or continuous R combined with antibody or immunotherapy could lead to a 30% increase in MRD-negative rate, a 20% upgrade of increase at 6 months post-randomisation when compared to continuous R treatment, as good as therapy combinations containing an alternative proteasome inhibitor.[33]

Following the strategy of Bratton and colleagues [36] for a multi-arm multi-stage trial with:

- N=2 stages (Stage 1 (activity) and Stage 2 (efficacy)) and
- J=4 experimental arms (RBorD+R, R+Isa, RBorIsaD + RIsa and TBC) and a single control arm (R)
- Where allocation is equal across each arms (1:1:1:1:1) and
- The power in Stage 1 and Stage 2 is 95% and 85%, respectively,
- the one-sided significance level in Stage 1 and Stage 2 is 25% and 1%, respectively,
- 10 participants per month are entered at R2, Stage 1 (activity) analysis and reporting requires 1 month and 5% of participants drop-out.

For peer review only - http://bmjopen.bmj.com/site/about/guidelines.xhtml

This equates to 40 participants in each arm at Stage I and 50 further participants in each arm at Stage 2. This will require a maximum of 46 months of recruitment plus a further 6 months of follow-up for the final participant. Overall, this design has a power of 83.4% and a one-sided family-wise error rate (FWER) of ~3%.

Randomisation 2 has been powered assuming four experimental arms and that all experimental arms will continue to stage 2. In the case that only three experimental arms are implemented or at least one experimental arm is concluded to show a lack of activity after stage 1, the trial will have a higher power than stated above.

Randomisation 3

Each arm of R3 will take the form of one-stage treatment design proposed by Simon, Wittes and Ellenberg [37] where the A'Hern design [38] is first implemented to determine treatment activity in both arms. Treatment activity will be determined by considering the PFS rate at 28 months. Data from Myeloma XI demonstrated a median PFS for high-risk participants in the intensive pathway of approximately 19 months (unpublished data). It is thought that for the treatments proposed to be investigated further in a Phase III trial they should increase the median PFS from 19 months to at least 28 months (9 month increase), i.e. increasing the PFS rate at 28 months from 34.7% to 50%.

In order to have an 80% power of demonstrating that the one-sided 95% confidence interval of the PFS rate at 28 months excludes 34.7%, 81 evaluable participants are required, where a treatment arm will be deemed a "success" if at least 37 out of 81 participants are progression-free at 28 months. To account for approximately 5% of participants being unevaluable at 28 months post-R3, 86 participants will be randomised to each treatment arm i.e. 172 participants are required to be entered into R3.

Total Study Sample Size

Accounting for those participants who will be lost-to-follow-up R1, R2 and R3 require 500, 450, and 172 participants to enter each randomisation respectively.

Considering R1 and R2, it is assumed that 50 participants will lose their MRD-negative status during the 12 months of continuous treatment (Myeloma XI, unpublished data). Therefore 550 participants are required to enter the MRD-negative part of the standard-risk pathway. It is assumed that 55% of standard-risk participants will be MRD-negative at the point of assessment therefore approximately 1000 participants are required to reach the MRD assessment stage of the standard-risk pathway [35 39] It is assumed that 90% of all those recruited will reach this point therefore 1112 participants are required to enter the standard-risk pathway[5] . It is anticipated that 80% of all those recruited will be standard-risk (Myeloma XI, unpublished data) therefore at least 1390 participants are required to be recruited to the trial. Rounding this we obtain an overall sample size of 1400.

This gives 280 participants who are expected to enter the high-risk pathway, using the same logic as in the standard-risk pathway if 90% of participants reach R3 then there will be approximately 252 participants recruited to R3. Given the calculations above it is plausible that another arm could be added to the high-risk pathway at a later date.

Recruitment:

For peer review only - http://bmjopen.bmj.com/site/about/guidelines.xhtml

It is planned that 1400 participants will be recruited for a total of 36 months. Once all centres are open, the recruitment target is 38 participants a month (equating to approximately 30 standard risk and 8 high-risk participants).

In order to ensure the trial will meet the target sample size within the recruitment period, site set-up was prioritised whilst the trial was preparing to open to recruitment. In addition, the trial team are actively engaging with Principal Investigators at sites who support the trial to ensure that those sites open quickly and are maintaining regular communication with open sites to ensure that they continue to recruit to the trial.

The trial opened to recruitment on 11/05/2021.

Assignment of treatment allocations:

Each of the registration and randomisation procedures will be conducted centrally using the University of Leeds Clinical Trial Research Unit (CTRU) automated 24-hour web base and telephone system.

Registration

If a patient is suspected to have myeloma they will enter the trial at the time of routine diagnostic tests, through bone marrow registration. Once diagnosis is confirmed locally and the research team consider the patient potentially eligible for the trial, patients will be provided with full verbal explanation of the trial and full participant information sheet and informed consent documents to consider. Once the patient has provided informed consent to the first stage of the trial they will be registered into the trial at Bone Marrow Registration.

Patients who have already had Myeloma confirmed prior to being approached for the trial, and those who decline consent at bone marrow registration, will enter at main trial registration.

Data Collection

Data collection will primarily be via Remote Data Entry (RDE) on electronic case report forms (eCRFs), provided by the CTRU at the University of Leeds, and will be entered by staff at the research site. Access to the live RADAR database will be provided by the CTRU following sites being authorised to open to recruitment; guidance on RDE and completing eCRFs will be provided. Data regarding informed consent, QoL, Healthcare Resource Use questionnaires and in-patient stay forms will be collected via paper case report forms.

QoL and Healthcare Resource Use questionnaires will be completed by the participant in clinic or at home and sealed in an envelope before handing back to the site research team who will return them to the CTRU via post.

Data Management

All information collected during the course of the trial will be kept strictly confidential. Information will be held securely on paper and electronically at the CTRU. The CTRU will comply with all aspects of the Data Protection Act 2018.

For peer review only

Data will be monitored for quality and completeness by the CTRU. Missing data will be chased until it is received, confirmed as not available or the trial is at analysis. The CTRU/Sponsor will reserve the right to intermittently conduct source data verification exercises on a sample of participants, which will be carried out by staff from the CTRU/Sponsor. Source data verification will involve direct access to participant notes at the participating hospital sites and the ongoing central collection of copies of Informed Consent Documents and other relevant investigation reports.

Statistical methods

Statistical analysis is the responsibility of the CTRU statisticians, with the exception of the cost-effectiveness analysis, which will be undertaken by the health economists at the University of Leeds. A full statistical analysis plan (SAP) and Health Economics Analysis Plan (HEAP) will be written and approved before any analyses are undertaken.

The analysis population will depend on the randomisation. Separate intention to treat (ITT), per protocol (PP) and safety populations will be defined for each randomisation. In either case the ITT population will include participants according to their randomisation allocation, regardless of eligibility whether they prematurely discontinued the treatment, or did not comply with the regimen. The PP population will exclude individuals who do not receive their randomisation allocation or who are found to be ineligible following randomisation. The safety population will consist of all participants who receive at least one dose of the trial treatments and analyse participants according to what they received rather than what they were randomised to.

With the exception of two interim analyses, no other formal analysis is planned until participants have attained the primary endpoint for each of the randomisations.

Primary endpoint analyses

<u>Randomisation 1</u>

The primary endpoint for R1 will be attained 2 years after the final participant has been randomised to R1.

As R1 is a non-inferiority trial, both PP and ITT population analyses will be used as co-primary analyses and both are required to demonstrate statistical significance of non-inferiority where any discrepancies will be reported.

PFS at R1 will be assessed using Cox proportional hazards regression to adjust for the stratification factors of R1 excluding centre, and presented using Kaplan-Meier estimates of the survival function. The treatment cessation strategy will be declared non-inferior to the continuous treatment strategy if the upper bound of the one-sided 95% confidence interval for the hazard ratio is less than 1.38. Note no adjustment will be made for interim analysis due to it being a test for inferiority (see below) and not non-inferiority.

<u>Randomisation 2</u>

The primary endpoint for R2 will be attained 6 months after the final participant has been randomised to R2. Primary analysis will be undertaken on the ITT population.

For peer review only - http://bmjopen.bmj.com/site/about/guidelines.xhtml

An overall one-sided approximately 3% FWER will be applied accounting for each pair-wise comparison over each stage and all comparisons. This equates to a 25% and 1% one sided significance level at interim and final analysis. MRD-negative rate will be compared between each pair-wise comparison (experimental vs control) in turn using logistic regression and adjusted for the minimisation factors of R2 excluding centre.

<u>Randomisation 3</u>

The primary endpoint for R3 will be attained 28 months after the final participant has been randomised to R3.

Primary analysis will be undertaken on the ITT population. The number of participants who are alive and progression-free at 28 months post R3 will be summarised separately within each treatment arm and exact one sided 95% confidence intervals calculated. The null hypothesis is that the percentage of participants alive and progression-free at 28 months post R3 is 34.7%. A treatment arm is deemed a "success" if the one sided 95% confidence intervals of the PFS rate at 28 months excludes 34.71%. The treatment will then be proposed to be investigated further in a phase III trial.

No multiple imputation is planned for the primary endpoints.

Secondary endpoint analyses

Overall survival and other time-to-event endpoints will be analysed using methods similar to those described for progression-free survival. All binary endpoints will be analysed using similar methods to those described for the attainment of MRD negativity endpoint.

For both R1 and R3, each experimental arm will be independently compared to historical control data using molecularly matched individual participant data (IPD) from Myeloma XI+(TE) and assessed for superiority in terms of progression-free survival. Further details of the molecular matching of IPD will be described in the statistical analysis plan, but it is anticipated that propensity score matching may be an appropriate technique. In each case a one-sided 10% significance level will be applied for each comparison with historical data.

The number and proportion of participants in each response category will be summarised by allocated treatment and exact 95% two-sided confidence intervals will be calculated. The difference in proportions for each response category will be presented with corresponding 95% two-sided confidence intervals.

Quality of life will be summarised using mean scores adjusted for baseline and two-sided 95% CI for each EORTC QLQ-C30 and EORTC QLQ-MY20 module symptom, role and functioning domain at each assessment time-point. Similar summaries will be produced for quality-adjusted life years (QALYs) as scored by the EQ-5D 3 Level (EQ-5D-3L) questionnaire. For the key secondary analysis of R1, the Global Health Status / Quality of Life subscale at two years post-R1, will be compared between the arms using a multivariable linear regression model, adjusting for the R1 stratification factors.

For peer review only - http://bmjopen.bmj.com/site/about/guidelines.xhtml

Exploratory and subgroup analyses

A series of subgroup and exploratory analyses will be undertaken. Subgroup analyses will follow the same structure as the main analysis of the respective endpoint. Where appropriate interaction terms will be added to the regression models to account for the subgroup being investigated. Subgroup and exploratory analyses may, by chance, generate false negative or positive results. Those carried out will be interpreted with caution.

The analysis for the exploratory outcomes will be detailed in the SAP.

Health economics

The economic evaluation will take a decision modelling approach to assess the cost-effectiveness of treatment options for risk stratified treatment vs standard non-stratified treatment over participants' lifetime. This will allow several research questions to be incorporated within one model. The model will be developed using best practice[40] and the model structure, health states and parameter values will be derived from the trial outputs, published literature, and expert clinical opinion. Data to model stratified treatment options will be drawn from the RADAR trial results while parameters for standard non-stratified treatment will be obtained from the wider literature.

Reflecting the main trial analysis of effectiveness, the economic evaluation will assess the cost-effectiveness of treatment options at each of the randomisations in RADAR. For standard risk participants at R1, the cost-effectiveness analysis will compare treatment with isatuximab for 12 cycles only to long-term isatuximab treatment, continued until disease progression. For standard risk participants who are MRD-positive at 100 days post-ASCT in terms of conversion to MRD negativity at R2, the cost-effectiveness of post-ASCT consolidation and maintenance treatments in the treatment of standard-risk participants will be evaluated. For high-risk participants at R3, the cost-effectiveness of post-ASCT consolidation and maintenance treatment strategies containing lenalidomide, bortezomib, dexamethasone and isatuximab separately will be evaluated. In addition, the cost-effectiveness of stratified treatment pathways will be compared with standard non-stratified treatments.

All analyses will be conducted following NICE reference case guidelines[41]. As such, outcomes will be measured in QALYs based on EQ-5D [42] and EORTC QLQ-C30 [43] utilities and costs will be calculated from a health and personal social services perspective. Cost-effectiveness will be determined in comparison to the NICE cost-effectiveness threshold of £20,000 per QALY gained. Deterministic and probabilistic (using Monte Carlo simulations) sensitivity analyses will be conducted to explore the impact of parameter value changes on estimates of cost-effectiveness and to determine the level of uncertainty around the results of the lifetime analysis. In addition, value of information analysis will be conducted to estimate the value of further research.

Trial Oversight

The TMG includes the CI, CTRU team and co-investigators that are assigned responsibility for the clinical set up, ongoing management, promotion of the trial and interpretation of the results. The trial steering committee (TSC), consisting of independent clinicians and statisticians, will provide overall

For peer review only - http://bmjopen.bmj.com/site/about/guidelines.xhtml

supervision on the trial, including trial progress, adherence to protocol, participant safety and consideration of new information.

Data Monitoring

An independent data monitoring and ethic committee (DMEC) will review the safety and ethics of the trial. Detailed reports will be prepared by the CTRU for the DMEC meetings which will take place at approximately yearly intervals. Additional reports will also be provided at intervals between these meetings. The committee will also review cumulative safety data by arm on an ongoing basis along with individual SAE/SAR listings. After each annual review, the DMEC will make their recommendations to the TSC about the continuation of the trial.

Interim Analyses

Full interim reports will be presented to the DMEC in confidence annually and following the planned interim analyses which are expected to take place when half the number of events (119) have been observed in the treatment de-escalation pathway (R1) and at the end of the activity stage 1 treatment escalation pathway (R2), approximately 6 months after the 40th participant has been randomised.

The interim analysis of R1 will be a one-sided test for inferiority conducted at the 5% significance level. PFS at R1 will be assessed using Cox proportional hazards regression to adjust for the minimisation factors (excluding centre), and presented using Kaplan-Meier estimates of the survival function.

The interim analysis for R2 will be conducted at the end of Stage I (the phase II part) of the randomisation which is due 6 months following the randomisation of the 40th participant. This analysis will compare each experimental arm with the control arm in separate tests of superiority using a one-sided 25% significance level and 95% power to determine whether any of the experimental arms show lack of activity i.e. the null hypothesis in the superiority test is not rejected. Should an arm show lack of activity it will not be continued to the second stage of the randomisation (the phase III part). To account for the interim analysis and to give an overall significance level of 1% and power of 80% for each individual comparison the final analysis will be conducted using a one-sided 1% significance level and 85% power. Recruitment will continue seamlessly between activity and efficacy in these scenarios. Formal powered pair-wise comparisons are not undertaken comparing experimental arms at either stage.

The DMEC committee members, in light of the interim analysis, will make their recommendations to the TSC about the continuation of the different aspects of the trial.

Harms

<u>Adverse events</u>

Adverse events are any untoward medical occurrence in a participant or clinical trial subject administered a medicinal product and which does not necessarily have a causal relationship with this treatment. AEs can be defined as any unintentional, unfavourable clinical signs or symptoms; any new illness or disease, or the deterioration of existing disease or illness; or clinically relevant deterioration in any laboratory assessments or clinical test. Due to the nature of multiple myeloma and its

For peer review only - http://bmjopen.bmj.com/site/about/guidelines.xhtml

For peer review only

treatment, participants are likely to experience several adverse events throughout the course of the disease.

All AEs, both related and unrelated to myeloma treatment will be collected on the relevant eCRF from trial registration until 60 days after the last dose of protocol treatment and will be evaluated in accordance with the National Cancer Institute Common Terminology Criteria for Adverse Events 5.0 (NCI-CTCAE). AEs and ARs should continue to be collected for participants who are randomised to 'stop isatuximab' at R1 (and until 60 days after last on-trial assessment), even though they will not be receiving any trial drugs during this period.

Adverse Events of special interest

An AE of special interest (serious or non-serious) is one of scientific and medical concern specific to isatuximab, for which ongoing monitoring and rapid communication by the investigator to the sponsor is considered to be appropriate. Isatuximab AEs of special interest include; ≥Grade 3 infusion reactions (IRs), Pregnancies, Symptomatic overdoses with IMP, and Second Primary Malignancies (SPMs).

Serious adverse events

Serious Adverse Events (SAEs) are defined as any untoward medical occurrence or effect that at any dose results in death; or are life threatening (at the time of the event); or require in-patient hospitalisation or prolongation of existing hospitalisation; or result in persistent or significant disability or incapacity; or result in a congenital anomaly or birth defect; or any other important medical event. All SAEs will be reported from registration until 60 days post the last dose of the trial drug. SAEs should continue to be collected for participants who are randomised to 'stop isatuximab' at R1 (and until 60 days after last on-study assessment), even though they will not be receiving any study drugs during this period.

Serious Adverse Reactions (SARs) are SAEs that are deemed possibly related to any dose administered of any trial treatment. Suspected unexpected serious adverse events (SUSARs) are SARs which are not listed in the reference safety information for that medicinal product. SARs and SUSARs will be reported from the date of first trial dose and for the duration of the trial.

Secondary malignancies

All new primary malignancies (SPMs) or suspected malignancies occurring from the date of trial registration and for the duration of the trial will be recorded. SPMs will be summarised and reviewed by an appointed member of the TMG who will determine whether trial treatment should continue.

Pregnancies

Participants registered into RADAR agree to follow the Celgene approved Pregnancy Prevention Programme. Pregnancies and suspected pregnancies (including a positive pregnancy test regardless of age or disease status) in a female participant must be reported throughout the study and for 12 months after the last dose of cyclophosphamide or for 5 months following cessation of protocol treatment, whichever is longest. Pregnancies and suspected pregnancies (including a positive pregnancy test regardless of age or disease status) in a male participant's partner must be reported

For peer review only - http://bmjopen.bmj.com/site/about/guidelines.xhtml

throughout the study and for 6 months after the last dose of cyclophosphamide or for 5 months following cessation of protocol treatment, whichever is longest.

Safety Analyses

Safety analyses will summarise all SUSARs, SARs, SAEs, AEs and ARs, respective to where they occurred within the trial pathway (Induction, ASCT, Pre-R1, Post-R1, Post-R2, Post-R3). Safety data will be presented by treatment group for the safety population in addition to relationship to trial treatment.

Auditing

The CTRU and the trial sponsor have procedures in place to ensure that serious breaches of Good Clinical Practice (GCP) or the trial protocol are picked up and reported. A triggered monitoring plan will ensure that sites at risk are monitored accordingly.

Patient and Public Involvement

RADAR has been developed following extensive discussion with the UK myeloma community. Patients were first involved into the research at the grant application stage, and were incorporated into the development of the primary endpoint of R1, in which they confirmed a research question involving the stopping of treatment given there was no detectable myeloma would be of interest. The trial consent and participant information documents were reviewed for clarity by a patient representative. To ensure that a patient perspective is considered throughout the trial a PPI advisory group, including 4-8 members will feed into TMG meetings, and plan to host quarterly meetings to discuss urgent matters. It is envisioned that the PPI group, may help with writing summaries of results that can be published on patient forums and websites and maybe even shared directly with patients.

Ethics and Dissemination

Research and ethics approval

Ethics approval has been obtained from the London – Central Research Ethics Committee (20/LO/0238). In addition capacity and capability has been confirmed by the appropriate research and development department for each participating centre prior to opening to participant recruitment into the trial. This included but was not limited to; having access to the non-trial supplied IMP (Bor, Cy and D), being able to conduct FISH as SoC, capacity of staff, ability to complete all protocol required assessments/investigations, and ability to archive trials documents.

Participants will be required to provide informed consent before joining the trial.

Protocol Amendments

The trial opened to recruitment on 11/05/2021 using protocol version 2, dated 29th April 2020. This publication is written based from protocol version 3 dated July 2021. An amendment to protocol version 3 is anticipated Mid-2022, which should include the following:

High-Risk Pathway

For peer review only - http://bmjopen.bmj.com/site/about/guidelines.xhtml

The cytogenetic abnormality del(1p) will be added to the definition of high-risk. In addition, Isa will be added to induction regimen for high-risk patients following their first cycle of RCyBorD (whilst their risk status is confirmed). Furthermore, R3 will be removed and high-risk patients will receive RBorIsaD + RIsa only post-ASCT. The pathway will follow a single-arm, single stage, phase II, three outcome design, with the new primary objective to assess the activity of RCyBorD+Isa induction followed by ASCT and RBorD+Isa consolidation and RIsa maintenance in terms of PFS at 18 months post trial registration.

Other changes

- The removal of the phone registration and randomisation;
- The clarification of allowing prescribed medication to remove risks of thromboembolism before preceding onto treatment in the exclusion criteria for registration (and for starting post ASCT treatment)
- An update of exclusion criteria to allow for previous plasmacytomas.

Consent or assent

The PI retains overall responsibility for the informed consent of participants at their site and must ensure that any medically qualified person delegated responsibility to participate in the informed consent process is duly authorised, trained and competent to participate according to the ethically approved protocol, principles of GCP and Declaration of Helsinki. Written consent will be obtained and signed by a medically qualified member of the site research team. Informed consent must be obtained, and the participant must be registered into the trial prior to the participant undergoing procedures that are specifically for the purposes of the trial and are out-with standard routine care at the participating site. This include the confirmation of optional consent (QoL/ Healthcare resource use questionnaires and decision regarding consent for samples to be used in future research). At any stage, participants can withdraw consent without repercussion.

Confidentiality

All information collected during the course of the trial will be kept strictly confidential. Information will be held securely on paper and electronically at the Clinical Trials Research Unit (CTRU). The CTRU will comply with all aspects of the Data Protection Act 2018.

Access to data

The CTRU will control the final trial datasets, and any requests for data will be reviewed by the trial management group in the first instance. Only requests that have a methodologically sound proposal and whose proposed use of the data has been approved by the independent trial steering committee, based on the principles of a controlled access approach, and subject to existing contractual agreements with the funder, will be considered. Proposals should be directed to the corresponding author in the first instance; to gain access, data requestors will need to sign a data access agreement.

For peer review only - http://bmjopen.bmj.com/site/about/guidelines.xhtml

Ancillary and post-trial agreements

Participants who stop trial treatment due to progression or any point prior to the end of trial will be treated off-trial at the discretion of their treating clinician. Those who stop prior to disease progression will be followed up 2 monthly until disease progression, death or the end of the trial. Following disease progression all participants will be followed up annually until death, or until the end of the trial for post progression endpoints.

Participants in the R1 pathway who are MRD-positive at 12 months post 12 cycles of isatuximab and therefore aren't eligible for randomisation 1 will continue to receive isatuximab maintenance, these participants would continue to be monitored, and data collected, as previously but trial samples would not be collected.

Dissemination policy

Authorship will be in keeping with the UK-MRA publication policy and due acknowledgement to participants, local investigators, funders and NCRI Haematological Oncology Study Group support made. The success of the trial depends upon the collaboration of all participants. For this reason, credit for the main results will be given to all those who have collaborated in the trial, through authorship and contributor-ship. Uniform requirements for authorship for manuscripts submitted to medical journals will guide authorship decisions alongside the guidance of the UK Myeloma Research Alliance Authorship Policy.

To maintain the scientific integrity of the trial, data will not be released prior to the end of the trial or a primary endpoint being reached, either for trial publication or oral presentation purposes, without the permission of the Trial Steering Committee and the (Co-)Chief Investigators. In addition, individual collaborators must not publish data concerning their participants that is directly relevant to the questions posed in the trial until the main results of the trial have been published and following written consent from the Sponsor.

For peer review only

Appendices

Informed consent material

The consent forms which are to be completed by the participant at Bone Marrow Registration and/or Trial Registration and prior to each randomisation pathways are included in supplementary material.

Biological specimens

The collection of central samples for laboratory analysis is summarised in Figures 3 and 4. The analysis to be conducted for trial purposes is stated in the supplementary material. Additional analysis may be carried out by each central laboratory provided the appropriate consent for sample use in future research has been provided by the participant at trial entry.

Author Contributions

The RADAR trial was conceived by KY and MC and designed by them in collaboration with DAC. All authors inputted into the development of the protocol and participant information sheet. The first

For peer review only - http://bmjopen.bmj.com/site/about/guidelines.xhtml

draft of the protocol paper was written by KLR and ABC. All authors reviewed and approved the final manuscript.

Funding

This trial is funded by Cancer Research UK, Sanofi Genzyme and Celgene: A BMS Company. The funders had no role in the design, collection, analysis or collection of data; in writing the manuscript; or in the decision to submit the manuscript for publication.

The trial sponsor is responsible for the overall conduct of the trial as defined by Directive 2001/20/EC is University of Leeds, UoL/LTHT Joint Sponsor QA office (CTIMPs); Research & Innovation Centre/Faculty of Medicine & Health; Leeds Teaching Hospitals NHS Trust/University of Leeds; St James University Hospital; Leeds LS9 7TF.

Competing Interest Statement

ABC, KLR, DAC, AH, CO, LBai, LBar, AP, RL report grants and non-financial support from BMS/Celgene, grants and non-financial support from Merck Sharpe & Dohme, grants and non-financial support from Amgen, grants and non-financial support from Takeda, during the conduct of the trial. DAC also reports travel support from Celgene Corporation.

KY, RDT, CB, GJ, GP, MC, BD, DM, GC, HA, KR,SA, AC have no conflicting interests to declare. MC declares, Bristol Myers Squibb- employee, Honoraria/travel support in the last 3 years from, Amgen ,BMS/Celgene, Janssen, Takeda, Abbvie. CP declares BMS/Celgene Ad boards and speaker fees, Sanotif – Ad board, speaker fees, conference registration fees. JS reports Carrying out consultancy work (Advisory Board) for Sanofi. And an educational speaking engagement for Celgene/BMS RP declares; Honoraria – Jannsen, BMS, Abbvie, GSK. Consultancy: GSK, Janssen. Meeting support: Janssen, Takeda, BMS. RO declares- Janssen - advisory board, honoraria, Celegene – honoraria, Beigene - advisory board, honoraria, Astra Zeneca – honoraria. MK declares inter-relationships: AbbVie: consultancy; Amgen: honoraria; BMS/Celgene: consultancy, research funding (institution); GSK: consultancy; Janssen: consultancy, research funding (institution); Karyopharm: consultancy; Pfizer: consultancy; SeattleGenetics: consultancy; Takeda: consultancy; Sanofi: honoraria. MD reports owning stock in Abingdon Health. SQ is the founder and CSO of Achilles therapeutics a company developing T cell therapies for solid tumours.

For peer review only - http://bmjopen.bmj.com/site/about/guidelines.xhtml

References

1. Cancer Research UK. Myeloma Incidence Statistics 2015 [Available from: http://www.cancerresearchuk.org/health-professional/cancer-statistics/statistics-by-cancer-type/myeloma/incidence accessed 21st October 2018 2018.
2. Kristinsson SY, Anderson WF, Landgren O. Improved long-term survival in multiple myeloma up to the age of 80 years. *Leukemia* 2014;28(6):1346-8. doi: 10.1038/leu.2014.23
3. Child JA, Morgan GJ, Davies FE, et al. High-dose chemotherapy with hematopoietic stem-cell rescue for multiple myeloma. *N Engl J Med* 2003;348(19):1875-83. doi: 10.1056/NEJMoa022340
4. Attal M, Harousseau JL, Stoppa AM, et al. A prospective, randomized trial of autologous bone marrow transplantation and chemotherapy in multiple myeloma. Intergroupe Francais du Myelome. *N Engl J Med* 1996;335(2):91-7. doi: 10.1056/NEJM199607113350204
5. Attal M, Lauwers-Cances V, Hulin C, et al. Lenalidomide, Bortezomib, and Dexamethasone with Transplantation for Myeloma. *N Engl J Med* 2017;376(14):1311-20. doi: 10.1056/NEJMoa1611750
6. Weber DM, Chen C, Niesvizky R, et al. Lenalidomide plus dexamethasone for relapsed multiple myeloma in North America. *N Engl J Med* 2007;357(21):2133-42. doi: 10.1056/NEJMoa070596
7. McCarthy PL, Holstein SA, Petrucci MT, et al. Lenalidomide Maintenance After Autologous Stem-Cell Transplantation in Newly Diagnosed Multiple Myeloma: A Meta-Analysis. *Journal of clinical oncology : official journal of the American Society of Clinical Oncology* 2017;35(29):3279-89. doi: 10.1200/JCO.2017.72.6679
8. Delforge M, Minuk L, Eisenmann JC, et al. Health-related quality-of-life in patients with newly diagnosed multiple myeloma in the FIRST trial: lenalidomide plus low-dose dexamethasone versus melphalan, prednisone, thalidomide. *Haematologica* 2015;100(6):826-33. doi: 10.3324/haematol.2014.120121
9. Kumar S, Flinn I, Richardson PG, et al. Randomized, multicenter, phase 2 study (EVOLUTION) of combinations of bortezomib, dexamethasone, cyclophosphamide, and lenalidomide in previously untreated multiple myeloma. *Blood* 2012;119(19):4375-82. doi: 10.1182/blood-2011-11-395749
10. Sonneveld P, Avet-Loiseau H, Lonial S, et al. Treatment of multiple myeloma with high-risk cytogenetics: a consensus of the International Myeloma Working Group. *Blood* 2016;127(24):2955-62. doi: 10.1182/blood-2016-01-631200
11. Joseph NS, Gentili S, Kaufman JL, et al. High-risk Multiple Myeloma: Definition and Management. *Clin Lymphoma Myeloma Leuk* 2017;17S:S80-S87. doi: 10.1016/j.clml.2017.02.018
12. Chng WJ, Dispenzieri A, Chim CS, et al. IMWG consensus on risk stratification in multiple myeloma. *Leukemia* 2014;28(2):269-77. doi: 10.1038/leu.2013.247
13. Shah V, Sherborne AL, Walker BA, et al. Prediction of outcome in newly diagnosed myeloma: a meta-analysis of the molecular profiles of 1905 trial patients. *Leukemia* 2018;32(1):102-10. doi: 10.1038/leu.2017.179
14. Boyd KD, Ross FM, Chiecchio L, et al. A novel prognostic model in myeloma based on co-segregating adverse FISH lesions and the ISS: analysis of patients treated in the MRC Myeloma IX trial. *Leukemia* 2012;26(2):349-55. doi: 10.1038/leu.2011.204
15. Kuiper R, Broyl A, de Knegt Y, et al. A gene expression signature for high-risk multiple myeloma. *Leukemia* 2012;26(11):2406-13. doi: 10.1038/leu.2012.127
16. Palumbo A, Avet-Loiseau H, Oliva S, et al. Revised International Staging System for Multiple Myeloma: A Report From International Myeloma Working Group. *Journal of clinical oncology : official journal of the American Society of Clinical Oncology* 2015;33(26):2863-9. doi: 10.1200/JCO.2015.61.2267
17. Paiva B, Vidriales MB, Cervero J, et al. Multiparameter flow cytometric remission is the most relevant prognostic factor for multiple myeloma patients who undergo autologous stem cell transplantation. *Blood* 2008;112(10):4017-23. doi: 10.1182/blood-2008-05-159624

For peer review only - http://bmjopen.bmj.com/site/about/guidelines.xhtml

18. Rawstron AC, Child JA, de Tute RM, et al. Minimal residual disease assessed by multiparameter flow cytometry in multiple myeloma: impact on outcome in the Medical Research Council Myeloma IX Study. *Journal of clinical oncology : official journal of the American Society of Clinical Oncology* 2013;31(20):2540-7. doi: 10.1200/JCO.2012.46.2119
19. Paiva B, van Dongen JJM, Orfao A. New criteria for response assessment: role of minimal residual disease in multiple myeloma. *Blood* 2015;125(20):3059-68. doi: 10.1182/blood-2014-11-568907
20. Sidiqi MH, Aljama MA, Bin Riaz I, et al. Bortezomib, lenalidomide, and dexamethasone (VRd) followed by autologous stem cell transplant for multiple myeloma. *Blood Cancer Journal* 2018;8(11):106. doi: 10.1038/s41408-018-0147-7
21. Facon T, Kumar S, Plesner T, et al. Daratumumab plus Lenalidomide and Dexamethasone for Untreated Myeloma. *The New England journal of medicine* 2019;380(22):2104-15. doi: 10.1056/NEJMoa1817249
22. Attal M, Richardson PG, Rajkumar SV, et al. Isatuximab plus pomalidomide and low-dose dexamethasone versus pomalidomide and low-dose dexamethasone in patients with relapsed and refractory multiple myeloma (ICARIA-MM): a randomised, multicentre, open-label, phase 3 study. *The Lancet (British edition)* 2019;394(10214):2096-107. doi: 10.1016/S0140-6736(19)32556-5
23. Deckert J, Wetzel MC, Bartle LM, et al. SAR650984, a novel humanized CD38-targeting antibody, demonstrates potent antitumor activity in models of multiple myeloma and other CD38+ hematologic malignancies. *Clin Cancer Res* 2014;20(17):4574-83. doi: 10.1158/1078-0432.CCR-14-0695
24. Kumar S, Paiva B, Anderson KC, et al. International Myeloma Working Group consensus criteria for response and minimal residual disease assessment in multiple myeloma. *The Lancet Oncology* 2016;17(8):e328-46. doi: 10.1016/S1470-2045(16)30206-6
25. Lahuerta JJ, Paiva B, Vidriales MB, et al. Depth of Response in Multiple Myeloma: A Pooled Analysis of Three PETHEMA/GEM Clinical Trials. *Journal of clinical oncology : official journal of the American Society of Clinical Oncology* 2017;35(25):2900-10. doi: 10.1200/JCO.2016.69.2517
26. Munshi NC, Avet-Loiseau H, Rawstron AC, et al. Association of Minimal Residual Disease With Superior Survival Outcomes in Patients With Multiple Myeloma: A Meta-analysis. *JAMA Oncol* 2017;3(1):28-35. doi: 10.1001/jamaoncol.2016.3160
27. Avet-Loiseau H, Bahlis NJ, Chng WJ, et al. Ixazomib significantly prolongs progression-free survival in high-risk relapsed/refractory myeloma patients. *Blood* 2017;130(24):2610-18. doi: 10.1182/blood-2017-06-791228
28. Chan A-W, Tetzlaff JM, Gøtzsche PC, et al. SPIRIT 2013 explanation and elaboration: guidance for protocols of clinical trials. *BMJ (Online)* 2013;346(jan08 15):e7586-e86. doi: 10.1136/bmj.e7586
29. Blade J, Samson D, Reece D, et al. Criteria for evaluating disease response and progression in patients with multiple myeloma treated by high-dose therapy and haemopoietic stem cell transplantation. Myeloma Subcommittee of the EBMT. European Group for Blood and Marrow Transplant. *Br J Haematol* 1998;102(5):1115-23.
30. Durie BG, Harousseau JL, Miguel JS, et al. International uniform response criteria for multiple myeloma. *Leukemia* 2006;20(9):1467-73. doi: 10.1038/sj.leu.2404284
31. Rajkumar SV, Harousseau JL, Durie B, et al. Consensus recommendations for the uniform reporting of clinical trials: report of the International Myeloma Workshop Consensus Panel 1. *Blood* 2011;117(18):4691-5. doi: 10.1182/blood-2010-10-299487
32. Jackson GH, Davies FE, Pawlyn C, et al. Lenalidomide Is a Highly Effective Maintenance Therapy in Myeloma Patients of All Ages; Results of the Phase III Myeloma XI Study. *Blood* 2016;128(22):1143-43.
33. Cocks K, King MT, Velikova G, et al. Evidence-based guidelines for determination of sample size and interpretation of the European Organisation for the Research and Treatment of Cancer

For peer review only - http://bmjopen.bmj.com/site/about/guidelines.xhtml

Quality of Life Questionnaire Core 30. *Journal of clinical oncology : official journal of the American Society of Clinical Oncology* 2011;29(1):89-96. doi: 10.1200/JCO.2010.28.0107
34. Royle KL, Gregory WM, Cairns DA, et al. Quality of life during and following sequential treatment of previously untreated patients with multiple myeloma: findings of the Medical Research Council Myeloma IX randomised study. *Br J Haematol* 2018;182(6):816-29. doi: 10.1111/bjh.15459
35. Avet-Loiseau H, Corre J, Lauwers-Cances V, et al. Evaluation of Minimal Residual Disease (MRD) By Next Generation Sequencing (NGS) Is Highly Predictive of Progression Free Survival in the IFM/DFCI 2009 Trial. American Society of Haematology (57th Annual Meeting and Exposition). Orlando, FL, 2015.
36. Bratton DJ, Phillips PP, Parmar MK. A multi-arm multi-stage clinical trial design for binary outcomes with application to tuberculosis. *BMC Med Res Methodol* 2013;13:139. doi: 10.1186/1471-2288-13-139
37. Simon R, Wittes RE, Ellenberg SS. Randomized phase II clinical trials. *Cancer Treat Rep* 1985;69(12):1375-81.
38. A'Hern RP. Sample size tables for exact single-stage phase II designs. *Stat Med* 2001;20(6):859-66. doi: 10.1002/sim.721
39. Reeder CB, Reece DE, Kukreti V, et al. Once- versus twice-weekly bortezomib induction therapy with CyBorD in newly diagnosed multiple myeloma. *Blood* 2010;115(16):3416-7. doi: 10.1182/blood-2010-02-271676
40. Caro JJ, Briggs AH, Siebert U, et al. Modeling good research practices--overview: a report of the ISPOR-SMDM Modeling Good Research Practices Task Force-1. *Med Decis Making* 2012;32(5):667-77. doi: 10.1177/0272989X12454577
41. Guide to the methods of technology appraisal 2013. London: NICE 2013.
42. Dolan P, Roberts J. Modelling valuations for Eq-5d health states: an alternative model using differences in valuations. *Med Care* 2002;40(5):442-6.
43. Rowen D, Brazier J, Young T, et al. Deriving a preference-based measure for cancer using the EORTC QLQ-C30. *Value Health* 2011;14(5):721-31. doi: 10.1016/j.jval.2011.01.004

For peer review only

For peer review only - http://bmjopen.bmj.com/site/about/guidelines.xhtml

Figure Legends/ Captions

Figure 1: Flow chart of participant's timelines in the RADAR study

Figure 2: Schedule of local investigations

Figure 3: Central Investigations from diagnosis up to 100 days post-ASCT

Figure 4: Central Investigations for the R1, R2 and R3 pathways

For peer review only

For peer review only - http://bmjopen.bmj.com/site/about/guidelines.xhtml

Figure 1: Flowchart of Participants timelines in the RADAR Study

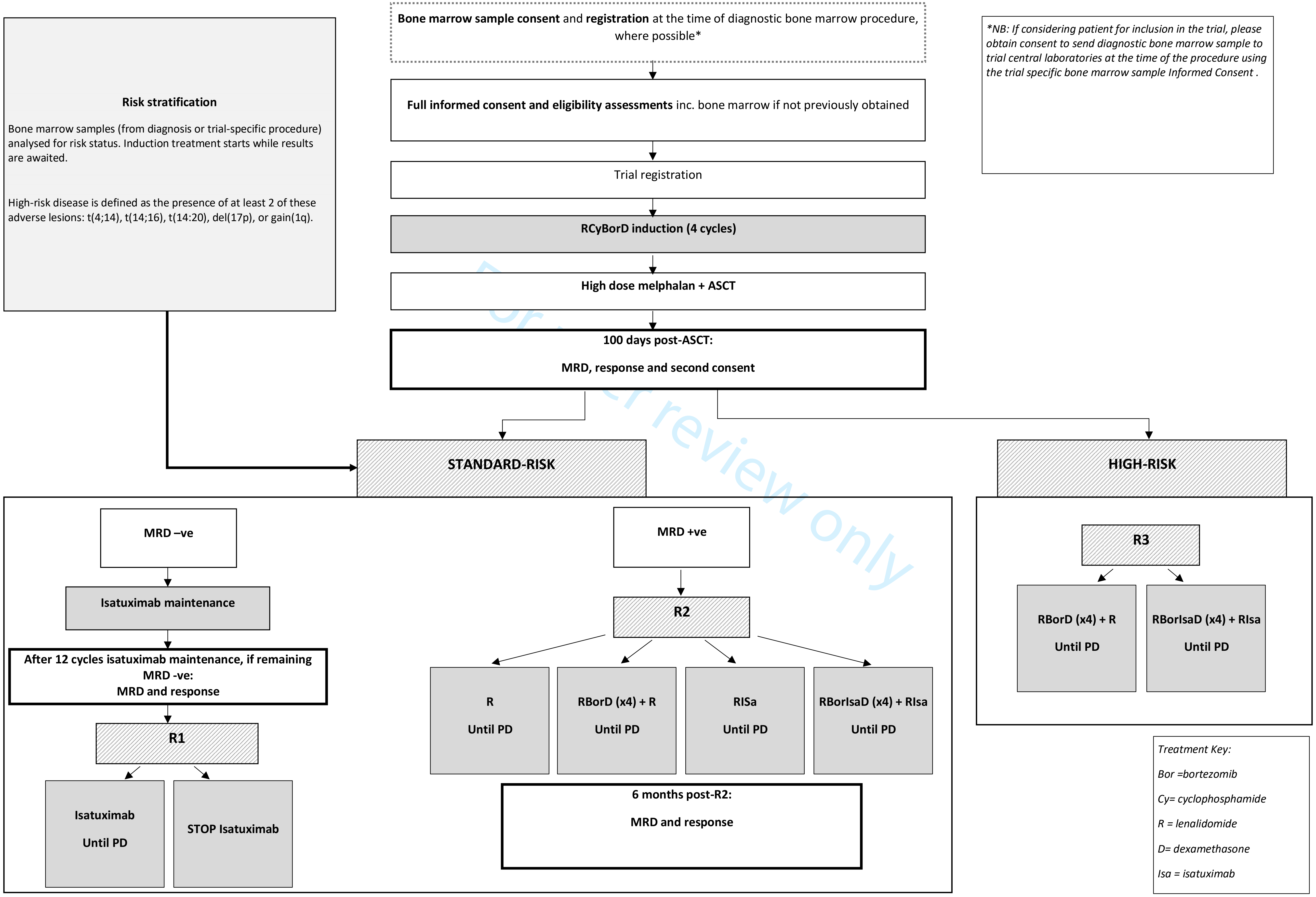

For peer review only

Figure 2: Schedule of Local Investigations

	RCyBorD Induction – All participants				R1 treatment pathway			R2 and R3 treatment pathway<br>R2 participants on R or RIsa arm: After 3,6,12 and 18 cycles**		
	**Pre-registration/baseline** (within 14 days prior to registration, unless otherwise stated)	**Day 1 (or day -1) of each cycle during RCyBorD induction††**	**End of RCyBorD induction**	**100 days post-ASCT** (within 14 days prior to R2, start of Isatuximab maintenance or R3)	**Day 1 of each cycle of isatuximab treatment††**	**On the day of isatuximab dose††**	**Follow up for participants coming off study during R1 treatment pathway: every 2 months**	**Day 1 of each cycle of treatment††**	**On day of (and prior to) every isatuximab dose††**	**Follow up for participants coming off study during R2/R3 treatment pathway: every 2 months**
Written informed consent	✓<br>Consent to be taken prior to all assessments, permitted outside the 14-day window			✓<br>(permitted outside the 14-day window)						
Physical examination	✓	✓	✓	✓	✓			✓		
ECOG	✓	✓	✓	✓	✓			✓		
Covid vaccination status	✓	✓	✓	✓	✓		✓	✓		✓
Medical history (including review for prior cancers and history of Covid infection)	✓		✓	✓						
Assessment of cardiac risk*	✓(results from formal measurements within 28 days prior to registration)	If clinically indicated	✓	If clinically indicated	If clinically indicated			If clinically indicated		
ECG					Every 3 months in participants receiving Isatuximab			Every 3 months in participants receiving Isatuximab		
Thyroid function monitoring*	✓(within 28 days prior to registration)	If clinically indicated	✓	If clinically indicated	If clinically indicated			If clinically indicated		
FBC, LFTs, albumin, LDH, U&Es, calcium, and creatinine¶	✓	✓	✓	✓	✓	✓ FBC, U&E and creatinine only		✓	✓ FBC, U&E and creatinine only	
Hepatitis B, (surface antigen and core antibody), ,hepatitis C and HIV	⍰(within 28 days prior to registration)									
β2-microglobulin (ß2M)	✓									
C-Reactive protein (CRP)	✓									
Calculated creatinine clearance (using Cockcroft-Gault formula)	✓	✓	✓	✓	✓			✓		
Serum paraprotein and immunofixation, serum free light chains and urinary free light chain detection	✓	✓	✓	✓	✓		✓ (if performed as part of standard care)	✓		✓ (If performed as standard of care)
Bone marrow (aspirate and trephine)<br>*See Table below for details of central investigations*	✓ (within 6 weeks prior to registration)	✓(recommended only if a first occurrence of CR or sCR is suspected)	✓	✓	✓(recommended only if a first occurrence of CR or sCR is suspected)		✓ (if performed as part of standard care)	✓ (recommended only if first occurrence of CR/sCR is suspected)		✓ (If performed as standard of care)
Pregnancy prevention counselling	✓	At least every 28 days during lenalidomide treatment								
Pregnancy test† (for females of childbearing potential)	✓	✓¥			✓			✓¥		
Cross-sectional imaging according to local practice, to include the thorax (low dose whole body CT recommended)	Within 3 months before registration§	If clinically indicated. ‡			If clinically indicated			If clinically indicated		
Assessment of adverse events (including adverse events and second primary malignancy)		Monitor throughout study and report on relevant treatment eCRFs (from first treatment dose until 60 days post last treatment dose)			Monitor throughout study and report on relevant treatment eCRFs (from first treatment dose until 60 days post last treatment dose) . AEs should continue to be monitored for participants randomised to 'stop Isatuximab' at R1 (and until 60 days after last on-study assessment).			Monitor throughout study and report on relevant treatment eCRFs (from first treatment dose until 60 days post last treatment dose)		

**Performed as part of standard of care.*
*** ECGs should be scheduled to align with other required face-to-face visits*
¶ The FBC should be repeated mid-cycle 1 (Day 14 +/-3 days) or more frequently and during subsequent cycles if there is a concern about cytopenias
† Females of childbearing potential (FCBP) must have a negative pregnancy test performed by a healthcare professional in accordance with the Celgene Lenalidomide Pregnancy Prevention Plan
†† May be performed within the 48 hours prior to the specified day
*** In addition to standard Day 1 assessments*
¥ All FCBP: The first pregnancy test must be performed within 10 to 14 days prior to the start of lenalidomide and the second pregnancy test must be performed within 24 hours prior to the start of lenalidomide.
FCBP with regular or no menstrual cycles: within 24 hours before start of lenalidomide treatment; weekly for the first 28 days of study participation; 4-weekly as a minimum during lenalidomide treatment; at study discontinuation; and 4 weeks after last dose of lenalidomide
FCBP with irregular menstrual cycles: within 24 hours before start of lenalidomide treatment; weekly for the first 28 days of study participation; 2-weekly as a minimum during lenalidomide treatment; at study discontinuation; and at 2 and 4 weeks after last dose of lenalidomide

N.B. During the COVID-19 pandemic, face-to-face assessments must be carried out at least at the beginning of cycles 1 and 2 and at the end of consolidation. Subsequently, participants must be seen by a clinician at least every 3 cycles (and more frequently if clinically indicated or there are concerns).

Figure 3: Central Investigations from diagnosis up to 100 days post-ASCT

Sample	Investigation	Diagnosis<br><br>(if patient consented on NHS form and trial bone marrow Informed Consent Document)[1]	Trial consent<br>Consented to Myeloma XV trial and central laboratory investigations	Baseline (post trial consent and before the start of treatment)	Day 1 of Cycle 1 RCyBorD induction	End of induction cycle 1	End of induction cycle 2	End of RCyBorD induction	100 days post-ASCT and prior to consolidation/ maintenance
0.5-1 mL EDTA bone marrow aspirate<br><br>**SEND TO HMDS, LEEDS**	Minimal Residual Disease	✓		✓<br><br>(if not sent previously)				✓	✓
10 mL clotted peripheral blood<br><br>Random urine sample<br><br>**SEND TO BIRMINGHAM**	Disease parameters (including paraproteins) for response assessments and covid-19 antibody response<br><br>M-protein intereference assay			✓	✓<br><br>**Only if baseline sample not within 14 days**	✓	✓	✓	✓
10 mL EDTA bone marrow aspirate<br><br>10 mL blood<br><br>**SEND TO UCL, LONDON**	Tumour DNA for neoantigens<br><br>cfDNA<br><br>Immune profiling	✓<br><br>Bone marrow only		✓<br><br>BMA (if not sent previously) & blood	✓<br><br>**Cycle 1 only**<br><br>10 mL blood (**only if baseline sample is not from within 14 days prior to cycle 1 day 1**)				✓
Bone marrow trephine (6 sections)<br><br>**SEND TO UCL, LONDON**	Immunostaining for immune function	✓		✓<br><br>(if not sent previously					✓

[1] Baseline bone marrow samples may be taken before trial consent, provided that patients have consented to these samples being taken using the standard NHS consent form. The patient must have provided written informed trial consent on the trial bone marrow Informed Consent Document before these samples can be sent to the central laboratories.

For peer review only

Figure 4: Central Investigations for the R1, R2 and R3 pathways

Sample	Investigation	Standard-risk MRD-negative participants (R1 pathway)	Standard-risk MRD-positive participants on R or RIsa arm (R2 pathway)			Standard-risk MRD-positive participants on RBorD+R or RBorIsaD+RIsa arm (R2 pathway)			High-risk participants (R3 pathway)			All participants
		After cycles 6, 12 and 24 of maintenance *(or at equivalent time-points if randomised to stop receiving Isa)*	After cycle 3	After cycles 6, 12 and 18	After cycle 24	After consolidation cycle 4	After maintenance cycles 3, 9 and 15	After maintenance cycle 21	After consolidation cycle 4	After maintenance cycles 3, 9 and 15	After maintenance cycle 21	Disease progression
**0.5-1 mL EDTA bone marrow aspirate** SEND TO HMDS, LEEDS	Minimal Residual Disease	✓	✓	✓		✓	✓		✓	✓		✓
**10 mL clotted peripheral blood** **Random urine sample** SEND TO BIRMINGHAM	Disease parameters (including paraproteins) for response assessments and covid-19 antibody response	✓	✓	✓	✓(blood only)	✓	✓	✓(blood only)	✓	✓	✓(blood only)	✓
	M-protein interference assay											
**10 mL EDTA bone marrow aspirate** **10 mL blood** SEND TO UCL, LONDON	Tumour DNA for neoantigens cfDNA Immune profiling	✓ (only after 6 cycles)	✓			✓			✓			✓
**Bone marrow trephine** SEND TO UCL, LONDON	Immuno-chemistry for immune function		✓			✓			✓			✓

RADAR: Protocol Paper Attachments

Contents

1 Glossary of Terms 2
2 List of Sites in the RADAR Trial 4
3 Spirit Checklist: Reporting checklist for protocol of a clinical trial. 5
4 Excluded Concomitant Medication: 11
5 Exploratory analysis 13
6 Subgroup analysis 14
7 Informed Consent Material 16
Bone Marrow Consent 16
Part A: Initial Treatment and Further Study Consent 19
Part B: Randomisation 1 Treatment Group 22
Part C: Randomisation 2 Treatment Group 24
Part D: Randomisation 3 Treatment Group 26
8 Central Sample Analysis 28
9 References 31

For peer review only

1 Glossary of Terms

AE	Adverse event
ALT	Alanine aminotransferase
ANC	Absolute neutrophil count
AR	Adverse reaction
ASCT	Autologous stem cell transplantation
β2M	Beta2-microglobulin
BD	Twice daily
CI	Chief Investigator
CR	Complete response
CRF	Case report form
CRUK	Cancer Research United Kingdom
CTCAE	Common Terminology Criteria for Adverse Events
CTRU	Clinical Trials Research Unit
Cy	Cyclophosphamide
D	Dexamethasone
DMEC	Data Monitoring and Ethics Committee
DSUR	Developmental Safety Update Report
ECOG	Eastern Cooperative Oncology Group
eCRF	Electronic Case Report Form
EORTC	European Organisation for Research and Treatment of Cancer
FISH	Fluorescence in situ hybridization test
FWER	Family-wise error rate
GCP	Good Clinical Practice
HDM	High-dose melphalan
HMDS	Haematological Malignancy Diagnostic Service
IMiD®	Immunomodulatory Drug
IMP	Investigational medicinal product
IMWG	International Myeloma Working Group
IPD	Individual participant data
Isa	Isatuximab
ISS	International Staging System
ITT	Intention to treat
LFT	Liver function test
MedDRA	Medical Dictionary for Regulatory Activities
mg	Milligram
MGUS	Monoclonal gammopathy of undetermined significance
MHRA	Medicines and Healthcare Products Regulatory Agency
MM	Multiple myeloma
MR	Minimal response
MRD	Minimal Residual Disease
NCRI	National Cancer Research Institute
NDMM	Newly diagnosed multiple myeloma
NICE	National Institute for Health and Care Excellence
OS	Overall survival
PD	Progressive disease
PFS	Progression-free survival

For peer review only

PFS2	Time to second progression
PI	Principal Investigator
PIS	Patient Information Sheet
PP	Paraprotein
PR	Partial response
PS	Performance status
QoL	Quality of life
R	Lenalidomide
R1-3	Randomisation 1-3
RCT	Randomised controlled trials
RDE	Remote Data Entry
REC	Research Ethics Committee
R-ISS	Revised International Staging System
RSI	Reference Safety Information
SC	Sub-cutaneous
SD	Stable disease
SAE	Serious adverse event
SAP	Statistical analysis plan
SAR	Serious adverse reaction
SmPC	Summary of Product Characteristics
SOP	Standard operating procedure
SPM	Secondary Primary Malignancy
SSOP	Study site operating procedure
SUSAR	Suspected unexpected serious adverse reaction
TE	Transplant eligible
TMG	Trial Management Group
TNE	Transplant non-eligible
TSC	Trial Steering Committee
U&Es	Urea & electrolytes
UK-MRA	United Kingdom Myeloma Research Alliance
ULN	Upper limit of normal
VGPR	Very good partial response

For peer review only

2 List of Sites in the RADAR Trial

Aberdeen Royal Infirmary	Royal Bournemouth Hospital	University Hospital Wishaw
Royal Sussex County Hospital	Poole General Hospital	St Richard's Hospital
Chesterfield Royal Hospital	Salisbury District Hospital	Worthing Hospital
Derriford Hospital	Sunderland Royal Hospital	The Christie
Kent & Canterbury Hospital, Canterbury	South Tyneside District Hospital	Cheltenham General Hospital
Queen Elizabeth the Queen Mother Hospital	Southmead Hospital Bristol	Gloucestershire Royal Hospital
William Harvey Hospital	St. Helens	The Clatterbridge Centre
Freeman Hospital	Whiston Hospital	
Queen Elizabeth Hospital Gateshead	St George's Hospital (Tooting)	
Hammersmith Hospital	Torbay Hospital	
Manchester Royal Infirmary	University College London Hospital	
Wythenshawe Hospital	Leicester Royal Infirmary	
Milton Keynes University Hospital	Worcestershire Royal Hospital	
Perth Royal Infirmary	Alexandra Hospital, Redditch	
Ninewells Hospital	York Hospital	
Northampton General Hospital	University Hospital of Wales, Cardiff	
Churchill Hospital	Stratford Hospital	
Horton General Hospital	Warwick Hospital	
Queen Alexandra Hospital, Portsmouth	New Cross Hospital	
Queen Elizabeth Hospital, Birmingham	University Hospital Coventry	
Raigmore Hospital, Inverness	University Hospital Monklands	
Rotherham General Hospital	University Hospital Hairmyres	

For peer review only

3 Spirit Checklist: Reporting checklist for protocol of a clinical trial.

Based on the SPIRIT guidelines.

Chan A-W, Tetzlaff JM, Gøtzsche PC, Altman DG, Mann H, Berlin J, Dickersin K, Hróbjartsson A, Schulz KF, Parulekar WR, Krleža-Jerić K, Laupacis A, Moher D. SPIRIT 2013 Explanation and Elaboration: Guidance for protocols of clinical trials. BMJ. 2013;346:e7586

		Reporting Item	Page Number
**Administrative information**			
Title	#1	Descriptive title identifying the study design, population, interventions, and, if applicable, trial acronym	1
Trial registration	#2a	Trial identifier and registry name. If not yet registered, name of intended registry	1,2,3
Trial registration: data set	#2b	All items from the World Health Organization Trial Registration Data Set	NA
Protocol version	#3	Date and version identifier	29
Funding	#4	Sources and types of financial, material, and other support	32
Roles and responsibilities: contributorship	#5a	Names, affiliations, and roles of protocol contributors	1, 31
Roles and responsibilities: sponsor contact information	#5b	Name and contact information for the trial sponsor	31,32
Roles and responsibilities: sponsor and funder	#5c	Role of study sponsor and funders, if any, in study design; collection, management, analysis, and interpretation of data; writing of the report; and the decision to submit the report for publication, including whether they will have ultimate authority over any of these activities	32
Roles and responsibilities: committees	#5d	Composition, roles, and responsibilities of the coordinating centre, steering committee, endpoint adjudication committee, data management team, and other individuals or groups overseeing the trial, if applicable (see Item 21a for data monitoring committee)	26,27
**Introduction**			
Background and rationale	#6a	Description of research question and justification for undertaking the trial, including summary of relevant	6,7

For peer review only

		studies (published and unpublished) examining benefits and harms for each intervention	
Background and rationale: choice of comparators	#6b	Explanation for choice of comparators	6,7
Objectives	#7	Specific objectives or hypotheses	7, 18
Trial design	#8	Description of trial design including type of trial (eg, parallel group, crossover, factorial, single group), allocation ratio, and framework (eg, superiority, equivalence, non-inferiority, exploratory)	8
**Methods: Participants, interventions, and outcomes**			
Study setting	#9	Description of study settings (eg, community clinic, academic hospital) and list of countries where data will be collected. Reference to where list of study sites can be obtained	8
Eligibility criteria	#10	Inclusion and exclusion criteria for participants. If applicable, eligibility criteria for study centres and individuals who will perform the interventions (eg, surgeons, psychotherapists)	8-14
Interventions: description	#11a	Interventions for each group with sufficient detail to allow replication, including how and when they will be administered	15-17
Interventions: modifications	#11b	Criteria for discontinuing or modifying allocated interventions for a given trial participant (eg, drug dose change in response to harms, participant request, or improving / worsening disease)	17
Interventions: adherence	#11c	Strategies to improve adherence to intervention protocols, and any procedures for monitoring adherence (eg, drug tablet return; laboratory tests)	17
Interventions: concomitant care	#11d	Relevant concomitant care and interventions that are permitted or prohibited during the trial	17
Outcomes	#12	Primary, secondary, and other outcomes, including the specific measurement variable (eg, systolic blood pressure), analysis metric (eg, change from baseline, final value, time to event), method of aggregation (eg, median, proportion), and time point for each outcome. Explanation of the clinical relevance of chosen efficacy and harm outcomes is strongly recommended	18

For peer review only

Participant timeline	#13	Time schedule of enrolment, interventions (including any run-ins and washouts), assessments, and visits for participants. A schematic diagram is highly recommended (see Figure)	18,19
Sample size	#14	Estimated number of participants needed to achieve study objectives and how it was determined, including clinical and statistical assumptions supporting any sample size calculations	20
Recruitment	#15	Strategies for achieving adequate participant enrolment to reach target sample size	23
**Methods: Assignment of interventions (for controlled trials)**			
Allocation: sequence generation	#16a	Method of generating the allocation sequence (eg, computer-generated random numbers), and list of any factors for stratification. To reduce predictability of a random sequence, details of any planned restriction (eg, blocking) should be provided in a separate document that is unavailable to those who enrol participants or assign interventions	23
Allocation concealment mechanism	#16b	Mechanism of implementing the allocation sequence (eg, central telephone; sequentially numbered, opaque, sealed envelopes), describing any steps to conceal the sequence until interventions are assigned	23
Allocation: implementation	#16c	Who will generate the allocation sequence, who will enrol participants, and who will assign participants to interventions	23
Blinding (masking)	#17a	Who will be blinded after assignment to interventions (eg, trial participants, care providers, outcome assessors, data analysts), and how	NA
Blinding (masking): emergency unblinding	#17b	If blinded, circumstances under which unblinding is permissible, and procedure for revealing a participant's allocated intervention during the trial	NA
**Methods: Data collection, management, and analysis**			
Data collection plan	#18a	Plans for assessment and collection of outcome, baseline, and other trial data, including any related processes to promote data quality (eg, duplicate measurements, training of assessors) and a description of study instruments (eg, questionnaires,	23

For peer review only

		laboratory tests) along with their reliability and validity, if known. Reference to where data collection forms can be found, if not in the protocol	
Data collection plan: retention	#18b	Plans to promote participant retention and complete follow-up, including list of any outcome data to be collected for participants who discontinue or deviate from intervention protocols	23
Data management	#19	Plans for data entry, coding, security, and storage, including any related processes to promote data quality (eg, double data entry; range checks for data values). Reference to where details of data management procedures can be found, if not in the protocol	23
Statistics: outcomes	#20a	Statistical methods for analysing primary and secondary outcomes. Reference to where other details of the statistical analysis plan can be found, if not in the protocol	18
Statistics: additional analyses	#20b	Methods for any additional analyses (eg, subgroup and adjusted analyses)	24
Statistics: analysis population and missing data	#20c	Definition of analysis population relating to protocol non-adherence (eg, as randomised analysis), and any statistical methods to handle missing data (eg, multiple imputation)	24
**Methods: Monitoring**			
Data monitoring: formal committee	#21a	Composition of data monitoring committee (DMC); summary of its role and reporting structure; statement of whether it is independent from the sponsor and competing interests; and reference to where further details about its charter can be found, if not in the protocol. Alternatively, an explanation of why a DMC is not needed	27
Data monitoring: interim analysis	#21b	Description of any interim analyses and stopping guidelines, including who will have access to these interim results and make the final decision to terminate the trial	27
Harms	#22	Plans for collecting, assessing, reporting, and managing solicited and spontaneously reported adverse events and other unintended effects of trial interventions or trial conduct	27
Auditing	#23	Frequency and procedures for auditing trial conduct, if any, and whether the process will be independent from investigators and the sponsor	29

For peer review only

**Ethics and dissemination**			
Research ethics approval	#24	Plans for seeking research ethics committee / institutional review board (REC / IRB) approval	29
Protocol amendments	#25	Plans for communicating important protocol modifications (eg, changes to eligibility criteria, outcomes, analyses) to relevant parties (eg, investigators, REC / IRBs, trial participants, trial registries, journals, regulators)	29
Consent or assent	#26a	Who will obtain informed consent or assent from potential trial participants or authorised surrogates, and how (see Item 32)	30
Consent or assent: ancillary studies	#26b	Additional consent provisions for collection and use of participant data and biological specimens in ancillary studies, if applicable	30,31
Confidentiality	#27	How personal information about potential and enrolled participants will be collected, shared, and maintained in order to protect confidentiality before, during, and after the trial	30
Declaration of interests	#28	Financial and other competing interests for principal investigators for the overall trial and each study site	32
Data access	#29	Statement of who will have access to the final trial dataset, and disclosure of contractual agreements that limit such access for investigators	30
Ancillary and post trial care	#30	Provisions, if any, for ancillary and post-trial care, and for compensation to those who suffer harm from trial participation	32
Dissemination policy: trial results	#31a	Plans for investigators and sponsor to communicate trial results to participants, healthcare professionals, the public, and other relevant groups (eg, via publication, reporting in results databases, or other data sharing arrangements), including any publication restrictions	31
Dissemination policy: authorship	#31b	Authorship eligibility guidelines and any intended use of professional writers	31
Dissemination policy: reproducible research	#31c	Plans, if any, for granting public access to the full protocol, participant-level dataset, and statistical code	31
**Appendices**			
Informed consent materials	#32	Model consent form and other related documentation given to participants and authorised surrogates	(Supplementary Material)

For peer review only

Biological specimens	#33	Plans for collection, laboratory evaluation, and storage of biological specimens for genetic or molecular analysis in the current trial and for future use in ancillary studies, if applicable	(Supplementary Material)

None The SPIRIT Explanation and Elaboration paper is distributed under the terms of the Creative Commons Attribution License CC-BY-NC. This checklist can be completed online using https://www.goodreports.org/, a tool made by the EQUATOR Network in collaboration with Penelope.ai

For peer review only

4 Excluded Concomitant Medication:

Excluded concomitant Medication:
1. Systemic treatment with any of the following metabolising enzyme inducers and inhibitors should be avoided, unless there is no appropriate alternative medication for the participant's use (if there were to be a drug-drug interaction with an inducer, cyclophosphamide and bortezomib metabolite exposure would be increased):<br>a. Strong CYP3A inducers: rifampin, rifapentine, rifabutin, carbamazepine, phenytoin, and phenobarbital<br>b. Strong CYP3A inhibitors<br><br>2. Excluded foods and dietary supplements include St. John's Wort, green tea and supplements containing green tea extracts<br><br>3. The following medicinal products and procedures are **prohibited** during this study:<br>a. Any antineoplastic treatment with activity against MM, other than study drugs<br>b. Platelet transfusions within 3 days prior to study drug dosing for any dosing day, or prior to randomisation in order to meet eligibility criteria.<br>c. Additional steroid therapy (not as trial IMP) (except before commencing trial treatment up to a maximum of 160mg dexamethasone equivalent as per inclusion criteria or steroids used to manage hypersensitivity to trial medication)<br>d. Radiotherapy for the treatment of symptoms related to disease progression.<br>e. Continuous use of clarithromycin (short courses of 5-10 days are permitted for treatment of infective episodes when indicated)<br>f. Hormone replacement therapy and erythropoietic agents and other agents that increase the risk of thrombosis (applicable during treatment with lenalidomide)

For peer review only

Quality of Life and Health Economics questionnaires

Timepoints for all participants up to 100 days post-ASCT

- Baseline
- End of induction
- 100 days post-ASCT

***R1 pathway participants**

- After cycles 6, 12, 24 and 36 (for participants randomised to 'stop isatuximab' at R1, the cycle 24 and 36 questionnaires should still be completed at equivalent timepoints)

***Timepoints for R2 and R3 pathway participants receiving R or RIsa**

- After cycles 6, 12 and 24 of maintenance

***Timepoints for R2 and R3 pathway participants receiving RBorD+R or RBorIsaD+RIsa**

- After cycles 3, 9 and 21 of maintenance.

**Note that participants who stop trial treatment should complete QoL at the equivalent time points (unless the participant wishes to withdraw consent for any further data collection)*

For peer review only

5 Exploratory analysis

Exploratory analysis will consider; the effect of post-ASCT consolidation regimens utilising combinations of lenalidomide, bortezomib, dexamethasone, and isatuximab on immune function in the bone marrow of participants following ASCT, using high dimensional characterisation of the immune microenvironment, immune subsets, phenotype and transcriptome; T cell receptor (TCR) repertoire in the bone marrow of participants following ASCT and consolidation and/or maintenance; correlation of baseline immune profile, TCR diversity and function with disease response, including MRD status and timing of disease relapse; ex-vivo functional studies with participant derived samples used to generate explants or immune-competent patient derived xenografts (icPDX); and to review infection and re-infection rates of COVID-19 in participants during induction, post-ASCT and during post-ASCT consolidation and maintenance treatment. All exploratory analysis to be conducted by the trial statisticians will be pre-specified in the statistical analysis plan.

For peer review only

6 Subgroup analysis

Subgroup analysis will be conducted to determine whether a selection of patient characteristics, haematology / local serological results, biochemistry results and cytogenetic/molecular results as well as response assessments (full list below) are prognostic of the endpoints; PFS, PFS2, OS, attainment of ≥VGPR and attainment of MRD negativity, as defined for all randomisations. Note that the subgroups listed under the Response subheading will not be analysed for the last two endpoints.

Patient Characteristics

- Age (years) at trial registration (≤65, >65)
- Sex (Male / Female)
- ECOG performance status at trial registration (0, 1, ≥2)
- ISS at trial registration (Stage I, Stage II, Stage III)
- R-ISS at trial registration (Stage I, Stage II, Stage III)

Haematology / Local Serological Results

- Haemoglobin concentration at trial registration (<100, ≥100 g/L)
- Platelets at trial registration (<150, ≥150 $x10^9$/L)
- White blood cells at trial registration (< LLN (Lower limit of normal), ≥LLN)
- Neutrophil count at trial registration (<LLN, ≥LLN)
- Lymphocyte count at trial registration (<LLN, ≥LLN)
- Plasma cells (%) in bone marrow at trial registration (<60%, ≥60%)

Biochemistry Results

- β-2 microglobulin concentration at trial registration (<3.5, 3.5–<5.5, ≥5.5 mg/L)
- Serum creatinine concentration at trial registration (<175, ≥175 µmol/L)
- Corrected serum calcium concentration at trial registration (<2.75, ≥2.75 mmol/L)
- Serum albumin at trial registration (<ULN (Upper limit of normal) , ≥ULN)
- Lactate dehydrogenase at trial registration (<ULN, ≥ULN)
- C-Reactive protein at trial registration (<ULN, ≥ULN)
- Serum bilirubin at trial registration (<ULN, ≥ULN)
- ALT/AST at trial registration (<ULN, ≥ULN)

Cytogenetic/molecular Results

- t(4,14) at trial registration (detected, not detected)
- t(14,16) at trial registration (detected, not detected)
- t(14,20) at trial registration (detected, not detected)
- del(17p) at trial registration (detected, not detected)
- 1q gain at trial registration (detected, not detected)
- Hyperdiploidy at trial registration (detected, not detected)
- The number of high-risk adverse lesions at trial registration (R1 and R2: 0, 1 unable to determine, R3: 2, ≥3, or unable to determine)

Response

For peer review only - http://bmjopen.bmj.com/site/about/guidelines.xhtml

- Response to RCyBorD induction (<VGPR, ≥ VGPR)
- Response at 100 days post-ASCT (<VGPR, ≥ VGPR)
- Response at the end of consolidation (<VGPR, ≥ VGPR)
- Response to 12m post-ASCT treatment (<VGPR, ≥ VGPR)
- MRD response to RCyBorD induction (R3 Only: MRD positive, MRD Negative)
- MRD at 100 days post-ASCT (R3 Only: MRD positive, MRD negative)
- MRD response to 6m post-ASCT treatment (R3 Only: MRD positive, MRD Negative)
- MRD response to 12m post-ASCT treatment (R3 Only MRD positive, MRD Negative)

Further subgroup analyses may be undertaken, as appropriate and all the subgroups will be fully defined within the statistical analysis plan according to the current criteria [1-3].

For peer review only

7 Informed Consent Material

Bone Marrow Consent

Delete this line, then print on Trust/Hospital headed paper

Participant ID:	Initials:
Date of Birth:	NHS or CHI number for Scotland/Hospital Number :
EudraCT Number:	Principal Investigator:

CANCER RESEARCH UK

RADAR (UK-MRA Myeloma XV)

INFORMED CONSENT DOCUMENT FOR BONE MARROW AND BLOOD SAMPLE

Please initial each box

1. I confirm that I have read and understand the information sheet for the bone marrow sample request and have had the opportunity to ask questions. ☐
2. I understand that my providing bone marrow samples is voluntary and that I am free to withdraw at any time without my medical care or legal rights being affected. ☐
3. I understand that the samples will be sent along with information about me, which will include NHS number or CHI number for Scotland and may include my name. ☐
4. I agree to these samples being stored and used for research investigations that form part of the RADAR study, and understand that this will include genetic research (for example, analysis of DNA). ☐

For peer review only

5. I understand that a unique reference number will be allocated to the samples which may allow them to be linked back to me in future for research purposes. ☐

6. I understand that my data may be shared on a collaborative basis with researchers in the UK and potentially, centres abroad. ☐

7. I agree to allow any information provided to be used for healthcare and/or further medical research upon the understanding that my identity will remain anonymous wherever possible. ☐

8. I understand that results of my laboratory tests may be shared with other research teams if I am taking part in another clinical study being run by the University of Leeds. ☐

9. I agree to a copy of this Consent Form being sent to the Clinical Trials Research Unit. ☐

10. I agree for my details (which will include date of birth and NHS number or CHI number for Scotland) to be submitted to NHS Digital or other central UK NHS bodies so that information about my health, and mortality should this become available, may be obtained by the CTRU if necessary. ☐

11. I agree to provide bone marrow samples. ☐

Please indicate your wishes in the below scenarios:

Please initial

A) If I am diagnosed with a plasma cell dyscrasia, other than myeloma, I give permission for the samples sent to the central laboratories to be stored and used in future research that receives ethical approval. I understand that the samples and data collected from them may be shared with researchers, possibly in other countries.

- -

Yes ☐ No ☐

B) If I am diagnosed with myeloma but do not take part in the RADAR study, I give permission for the samples sent to the central laboratories to be stored and used in future research that receives ethical approval. I understand that the samples and data collected from them may be shared with researchers, possibly outside the EEA.

Yes ☐ No ☐

Patient (to be completed by the patient):

Signature……….

Name (block capitals)………………………………………………………………………………………………………..

Date……….

Investigator:

For peer review only

I have explained the request to the above named patient and he/she has indicated his/her willingness for samples of bone marrow to be sent to the RADAR laboratories.

Signature……

Name (block capitals)…………………………………………………………………………………………………….

Date……

(If used)Translator:

Signature……

Name (block capitals)…………………………………………………………………………………………………….

Date……

(1 copy for patient; 1 for the CTRU; 1 held in patient notes, original stored in Investigator Site File)

For peer review only

Part A: InitialTreatment and Further Study Consent

Delete this line, then print on Trust/Hospital headed paper

Participant ID:	Initials:
Date of Birth:	NHS or CHI number for Scotland/Hospital Number:
EudraCT Number:	Principal Investigator:

CANCER RESEARCH UK

RADAR (UK-MRA Myeloma XV)

INFORMED CONSENT DOCUMENT PART A

Initial Treatment and Further Study Information

PLEASE INITIAL EACH BOX

12. I confirm that I have read and understand the Part A information document for the RADAR study and have had the opportunity to ask questions. ☐

13. I understand that my participation in this study is voluntary and that I am free to withdraw at any time without my medical care or legal rights being affected. I understand that even if I withdraw from the above study, the data and samples collected from me will be used in analysing the results of the study, and in some cases further information about any unwanted effects of my treatment may need to be collected by the study team. ☐

14. I understand that my healthcare records may be looked at by authorised individuals from the study team, regulatory bodies or Sponsor in order to check that the study is being carried out correctly. ☐

15. I am aware that I am required to have samples taken and sent to laboratories and I understand that this will include genetic research (for example, analysis of DNA). I understand that the samples will be sent along with information about me, which will include NHS number, or CHI number for Scotland, and may include my name. ☐

For peer review only

16. I understand that results or reports from imaging scans, blood tests or other investigations (including for additional cancers) may be sent to the Clinical Trials Research Unit for central review by the research team.

17. I agree to allow any information or results arising from this study to be used for healthcare and/or further medical research upon the understanding that my identity will remain anonymous wherever possible.

18. I understand that my data and results of my laboratory tests (blood, urine and bone marrow sample collections) may be shared with other research teams if I am taking part in another clinical study being run by the University of Leeds.

19. I agree for my details (which will include date of birth and NHS number or CHI number for Scotland) to be submitted to NHS Digital or other central UK NHS bodies so that information about my health may be obtained by the CTRU if necessary.

20. I understand that my GP, or any other doctor treating me, will be notified of my participation in this study.

21. I understand that some of my data may be passed to other organisations, such as the pharmaceutical companies Sanofi and Celgene, (possibly in other countries where the data protection standards and laws may be different from the UK) to monitor the safety of the treatment(s) that I am receiving. I understand that my identity will remain anonymous.

22. I agree to a copy of this Consent Form being sent to the Clinical Trials Research Unit.

23. I agree to take part in the study.

The following parts of the study are OPTIONAL (see section 3 of the Participant Information Sheet). Even if you agree to take part in the RADAR study, you do not have to agree to these optional parts. Please initial yes or no.

Please initial

A) I agree to take part in the Quality of Life and Healthcare Resource Use studies by completing questionnaires at timepoints throughout the study.

Yes No

B) I give permission for samples that I provide to be used in future research that receives ethical approval, and I understand that this includes the bone marrow samples provided at the time of my diagnosis (if I consented separately to collection of the diagnostic bone marrow sample in anticipation of participation in this study). I understand that my samples and data collected from them may be shared on a collaborative basis with researchers in the UK and, potentially, centres abroad.

Yes No

For peer review only

Patient (to be completed by the patient):

I agree to take part in this study.

Signature……….

Name (block capitals)……………………………………………………………………………………………….

Date……..

Investigator:

I have explained the study to the above named patient, and he/she has indicated his/her willingness to participate in this study.

Signature……….

Name (block capitals)……………………………………………………………………………………………….

Date……

(If used) Translator:

Signature……….

Name (block capitals)……………………………………………………………………………………………….

Date……

(1 copy for patient; 1 for the CTRU; 1 held in patient notes, original stored in Investigator Site File)

For peer review only

Part B: Randomisation 1 Treatment Group

Delete this line, then print on Trust/Hospital headed paper

Participant ID:	Initials:
Date of Birth:	NHS or CHI number for Scotland/Hospital Number:
EudraCT Number:	Principal Investigator:

CANCER RESEARCH UK

RADAR (UK-MRA Myeloma XV)

INFORMED CONSENT DOCUMENT PART B

R1 Treatment Group

PLEASE INITIAL EACH BOX

24. I confirm that I have read and understand the Part B information document for the RADAR study and have had the opportunity to ask questions. ☐

25. I agree to continue my participation in the study as part of the R1 Treatment Group. ☐

Patient (to be completed by the patient):

I agree to take part in this study.

Signature……

Name (block capitals)……………………………………………………………………………………………………

Date……

Investigator:

I have explained the study to the above named patient, and he/she has indicated his/her willingness to participate in this study.

Signature……

Name (block capitals)……………………………………………………………………………………………………

Date……

For peer review only

(If used) Translator:

Signature……………………………………………..……………………………………………………………………

Name (block capitals)…………………………………………….………………………………………………….

Date……………………………………………………..……………………………………………………………………

(1 copy for patient; 1 for the CTRU; 1 held in patient notes, original stored in Investigator Site File)

For peer review only

Part C: Randomisation 2 Treatment Group

Delete this line, then print on Trust/Hospital headed paper

Participant ID:	Initials:
Date of Birth:	NHS or CHI number for Scotland/Hospital Number:
EudraCT Number:	Principal Investigator:

CANCER RESEARCH UK

RADAR (UK-MRA Myeloma XV)

INFORMED CONSENT DOCUMENT PART C

R2 Treatment Group

PLEASE INITIAL EACH BOX

26. I confirm that I have read and understand the Part C information sheet for the RADAR study and have had the opportunity to ask questions. ☐

27. I agree to continue my participation in the study as part of the R2 Treatment Group. ☐

Patient (to be completed by the patient):

I agree to take part in this study.

Signature……….

Name (block capitals)……………………………………………………………………………………………….

Date…….

Investigator:

I have explained the study to the above named patient, and he/she has indicated his/her willingness to participate in this study.

Signature……….

Name (block capitals)……………………………………………………………………………………………….

For peer review only

Date………

(If used) Translator:

Signature……

Name (block capitals)……………………………………………………………………………………………………….

Date………

(1 copy for patient; 1 for the CTRU; 1 held in patient notes, original stored in Investigator Site File)

For peer review only

Part D: Randomisation 3 Treatment Group

Delete this line, then print on Trust/Hospital headed paper

Participant ID:	Initials:
Date of Birth:	NHS or CHI number for Scotland/Hospital Number:
EudraCT Number:	Principal Investigator:

CANCER RESEARCH UK

RADAR (UK-MRA Myeloma XV)

INFORMED CONSENT DOCUMENT PART D

R3 Treatment Group

PLEASE INITIAL EACH BOX

28. I confirm that I have read and understand the Part D information sheet for the RADAR study and have had the opportunity to ask questions. ☐

29. I agree to continue my participation in the study as part of the R3 Treatment Group. ☐

Patient (to be completed by the patient):

I agree to take part in this study.

Signature……

Name (block capitals)………………………………………………………………………………………………….

Date……

Investigator:

I have explained the study to the above named patient, and he/she has indicated his/her willingness to participate in this study.

Signature……

Name (block capitals)………………………………………………………………………………………………….

Date……

For peer review only

(If used) Translator:

Signature………

Name (block capitals)……….

Date……

(1 copy for patient; 1 for the CTRU; 1 held in patient notes, original stored in Investigator Site File)

For peer review only

8 Central Sample Analysis

The trial has 3 central laboratories receiving samples for trial purposes. The time points and the reason for analysis for each central sample can be found in Figure 2. Details on the analysis of each sample is below. Note that participants can consent for their samples to be used in future research and hence each laboratory can store their trial samples for use in future studies. The storage details are also included below.

Birmingham

The Birmingham University Clinical Immunology Laboratory holds UKAS ISO 15189:2012 - Medical Laboratories accreditation. The Trial-related Laboratory Work will be performed using standard assays to the same quality standard as during routine clinical care. The Laboratory will be responsible for the analysis of whole clotted blood, serum and urine collected from participants of the Research Project in accordance with the Protocol. The laboratory will measure disease response and Disease progression, defined according to the IMWG 2016 response criteria for Multiple Myeloma [21 26-28]. This will include assessment of prognostic markers, markers of disease activity and M-protein levels. Analyses will include serum levels of beta-2 microglobulin, immunoglobulins IgG, IgA and IgM, kappa and lambda free immunoglobulin light chains. Serum electrophoresis and immunofixation, M-protein quantitation including total protein and densitometry. Urine immunofixation and free light chain quantitation will be performed where appropriate. As the trial proceeds new biomarkers of myeloma disease activity are likely to become available and will also be measured. The Laboratory will ensure that the Materials are received, handled, analysed, stored and destroyed in line with the individual consent provided.

HMDS

The presence of minimal residual disease will be assessed in bone marrow aspirates using a validated flow cytometry assay (sensitivity ⩽0.001%) performed at a single central laboratory (HMDS, Leeds Teaching Hospitals Trust). A minimum of 2,500,000 cells will be evaluated with an eight-colour antibody combination of CD138, CD38, CD45, CD19, CD56, CD27, CD81 and CD117. Additional antigens for detection of CD38-negative plasma cells will be utilised as required. % neoplastic plasma cells and % normal plasma cells as a percentage of leucocytes will be reported. Limit of detection (LoD) and limit of quantification (LoQ) will also be calculated for each sample.
Samples will be used for trial purposes only and will not be stored for future research.

For peer review only

UCL

Bone marrow aspirates, peripheral blood and bone marrow trephine processing within UCL Myeloma Lab for RADAR clinical trial

OBJECTIVES

To provide molecular and genetic data on bone marrow immune profiles of newly diagnosed myeloma patients prior and post autologous stem cell transplantation. This will also be analysed between study arms to compare how different treatment regimes alert immune landscape in patients in particular anti-CD38 mAb Isatuximab.

METHODS

BMA and PB:

1. Sites are requested to collect samples and ship as per Lab Manual together with completed sample request form.

2. All samples will be processed in Myeloma Lab at UCL Cancer Institute.

3. UCL ECMC GCLP Facility will serve as storage facility for the following materials: PB plasma, BM serum, pellets (BM, PB, CD138+ and CD138-), protein lysates, extracted RNA and DNA. All material to be stored at -80˚C.

4. Myeloma Lab will serve as processing facility for all samples and storing of the following materials: BM MNCs (mononuclear cells), PB MNCs, CD138+ cells and CD138- cells. All material to be stored in liquid nitrogen.

5. Both -80˚C freezers and liquid nitrogen tanks are temperature monitored with systems that alert when temperature is out of acceptable range.

6. All samples and material extracted will be logged into the trial specific lab book and database with a unique trial ID, lab ID and time-point.

7. Upon arrival bone marrow aspirates and peripheral blood will be centrifuged. Serum (from BMA) and plasma (from PB) will be collected, aliquoted into 1-1.5ml aliquots and frozen at -80˚C.

8. BMA and PB mononuclear cells will then be isolated and enriched using Ficoll density gradient centrifugation.

9. PB MNCs will then be frozen as pellets and/or cryopreserved.

10. Depending on the tumour burden in the BM MNCs samples will be either frozen as MNCs pellets and/or cryopreserved or tumour cells will be isolated.

11. Tumour will be isolated via column-based MACS cell positive selection using CD138 magnetic beads (MicroBeads).

12. Depending on the number of cells isolated, CD138+ plasma cells will be frozen and stored as pellets and/or protein lysates and/or cryopreserved.

13. CD138- cells will be frozen and stored as pellets and/or cryopreserved.

Bone marrow trephine:

1. Sites are requested to collect, process and ship samples as per Lab Manual together with completed sample request form.

2. Myeloma lab will serve as storage facility for bone marrow trephine slides.

3. Upon arrival all samples will be logged into trial specific database with a unique trial ID, lab ID and time-point.

4. Upon arrival slides will be labelled and stored in the air-tight slide storage box.

ANALYSIS

1. High-dimensional flow cytometry will be used to profile immune context of patient bone marrow mononuclear cells prior and post-transplant.

2. Selected samples will be analysed further using transcriptomic profiling, T cell receptor (TCR) sequencing or single cell sequencing and multiplex immunofluorescence/immunohistochemistry on bone marrow trephine biopsies.

For peer review only

9 References

1. Oxman, A.D., *Subgroup analyses.* BMJ, 2012. **344**.
2. Sun, X., et al., *Is a subgroup effect believable? Updating criteria to evaluate* the *credibility of subgroup analyses.* BMJ, 2010. **340**.
3. Sun, X., et al., *Credibility of claims of subgroup effects in randomised controlled trials: systematic review.* BMJ, 2012. **344**.

For peer review only

BMJ Open

Risk and response adapted therapy following autologous stem cell transplant in patients with newly diagnosed multiple myeloma (RADAR (UK-MRA Myeloma XV Trial): study protocol for a Phase II/III randomised controlled trial

Journal:	*BMJ Open*
Manuscript ID	bmjopen-2022-063037.R1
Article Type:	Protocol
Date Submitted by the Author:	28-Jul-2022
Complete List of Authors:	Royle, Kara-Louise; University of Leeds Clinical Trials Research Unit<br>Coulson, Amy; University of Leeds Clinical Trials Research Unit<br>Ramasamy, Karthik; Oxford University Hospitals NHS Trust, Haematology; Oxford University, Radcliffe Department of Medicine<br>Cairns, David; University of Leeds Clinical Trials Research Unit<br>Hockaday, Anna; University of Leeds Clinical Trials Research Unit<br>Quezada, Sergio; UCL Cancer Institute<br>Drayson, Mark ; University of Birmingham<br>Kaiser, Martin; Institute of Cancer Research<br>Owen, Roger; St James's University Hospital, HMDS<br>Auner, Holger; Imperial College London, Department of Immunology and Inflammation; Imperial College London, Langmuir Centre for Myeloma Research<br>Cook, Gordon; University of Leeds Clinical Trials Research Unit; St James's University Hospital, Leeds Cancer Centre<br>Meads, David; University of Leeds, Leeds Institute of Health Sciences<br>Olivier, Catherine; University of Leeds Clinical Trials Research Unit<br>Barnard, Lorna; University of Leeds Clinical Trials Research Unit<br>Paterson, Andrea; University of Leeds Clinical Trials Research Unit<br>Lambkin, Rhiannon; University of Leeds Clinical Trials Research Unit<br>Dawkins, Bryony ; University of Leeds Leeds Institute of Health Sciences, Academic Unit of Health Economics<br>Chapman, Mike; University of Cambridge, Department of Haematology<br>Pratt, Guy; Queen Elizabeth Hospital<br>Popat, Rakesh; University College London Hospitals NHS Foundation Trust<br>Jackson, Graham; Leeds Teaching Hospitals NHS Trust, Department of Haematology<br>Bygrave, Ceri; University Hospital of Wales<br>Sive, Jonathan; University College London Hospitals NHS Foundation Trust<br>de Tute, Ruth; St James's University Hospital, HMDS<br>Chantry, Andrew; Royal Hallamshire Hospital, Department of Haematology<br>Parrish, Christopher; Leeds Teaching Hospitals NHS Trust, Haematology<br>Cook, Mark; University of Birmingham Institute of Cancer and Genomic Sciences; Bristol Myers Squibb

	Asher, Samir; UCL Cancer Institute<br>Yong, Kwee; UCL Cancer Institute
**Primary Subject Heading**:	Oncology
Secondary Subject Heading:	Haematology (incl blood transfusion)
Keywords:	Myeloma < ONCOLOGY, Myeloma < HAEMATOLOGY, Clinical trials < THERAPEUTICS, Cancer genetics < GENETICS, STATISTICS & RESEARCH METHODS, Protocols & guidelines < HEALTH SERVICES ADMINISTRATION & MANAGEMENT

SCHOLARONE™
Manuscripts

Risk and response adapted therapy following autologous stem cell transplant in patients with newly diagnosed multiple myeloma (RADAR (UK-MRA Myeloma XV Trial): study protocol for a Phase II/III randomised controlled trial

Kara-Louise Royle[1], Amy Beth Coulson[1], Karthik Ramasamy[2], David A Cairns[1], Anna Hockaday[1], Sergio Quezada[3], Mark Drayson[4], Martin Kaiser[5], Roger Owen[6], Holger W. Auner[7], Gordon Cook[1,8], David Meads[19], Catherine Olivier[1], Lorna Barnard[1], Rhiannon Lambkin[1], Andrea Paterson[1], Bryony Dawkins[19], Mike Chapman[9], Guy Pratt[10], Rakesh Popat[11], Graham Jackson[12], Ceri Bygrave[13], Jonathan Sive[11], Ruth de Tute[6], Andrew Chantry[14], Christopher Parrish[1,16,] Mark Cook[16,17], Samir Asher[18], Kwee Yong [18]

1. Clinical Trials Research Unit, Leeds Institute of Clinical Trials Research, University of Leeds, Leeds, United Kingdom
2. Oxford University Hospitals NHS Foundation Trust, Radcliffe Department of Medicine, Oxford University
3. Cancer Immunotherapy Group, UCL Cancer Institute, 72 Huntley Street, London WC1E 6BT
4. Clinical Immunology Service, Institute of Immunology and Immunotherapy, Medical School, University of Birmingham, Edgbaston, Birmingham, B15 2TT
5. The Institute of Cancer Research (ICR), Centre for Myeloma Research, Division of Molecular Pathology, Brookes Lawley Building, 15 Cotswold Road, London, Surrey, SM2 5NG
6. HMDS, St James's University Hospital, Leeds, LS9 7TF
7. Centre for Haematology, Imperial College London, London, W12 0NN
8. Leeds Cancer Centre, St James's University Hospital, Leeds, United Kingdom
9. Department of Haematology, University of Cambridge, Cambridge, CB2 0XY
10. Department of Haematology; Queen Elizabeth Hospital, Birmingham, B15 2TH
11. University College London Hospitals NHS Foundation Trust, NW1 2BU
12. Northern Centre for Cancer Care, Freeman Hospital, Newcastle-Upon-Tyne, NE7 7DN
13. University Hospital of Wales, Heath Park, Cardiff, CF14 4XW
14. Department of Haematology, Royal Hallamshire Hospital, Sheffield, S10 2RX
15. Leeds Teaching Hospitals NHS Trust, St James's Institute of Oncology, Beckett Street, Leeds, LS9 7TF, UK
16. University of Birmingham Institute of Cancer and Genomic Sciences
17. Bristol Myers Squibb, Boudry Switzerland
18. UCL Cancer Institute, London, WC1E 6BT
19. Academic Unit of Health Economics, Leeds Institute of Health Sciences, University of Leeds, Leeds, United Kingdom

*corresponding author
Miss Kara-Louise Royle,
Clinical Trials Research Unit, Leeds Institute of Clinical Trials Research, University of Leeds, Leeds, United Kingdom, LS2 9JT

Tel: 0113 343 8366

For peer review only - http://bmjopen.bmj.com/site/about/guidelines.xhtml

Email: k.l.royle@leeds.ac.uk

Word count: 8343

For peer review only

For peer review only - http://bmjopen.bmj.com/site/about/guidelines.xhtml

Abstract

Introduction

Multiple myeloma is a plasma cell malignancy that accounts for 1-2% of newly diagnosed cancers. At diagnosis, approximately 20% of patients can be identified, using cytogenetics, to have inferior survival (high-risk). Additionally, standard-risk patients, with detectable disease (minimal residual disease (MRD)-positive) post-autologus stem cell transplant (ASCT), fare worse compared to those who do not (MRD-negative). Research is required to determine whether a risk-adapted approach post-ASCT could further improve patient outcomes.

Methods

RADAR is a UK, multi-centre, risk-adapted, response-guided, open-label, randomised controlled trial for transplant-eligible newly diagnosed multiple myeloma patients, using combinations of lenalidomide (R), cyclophosphamide (Cy), bortezomib (Bor), dexamethasone (D) and isatuximab (Isa).

Participants receive RCyBorD(x4) induction therapy, followed by high-dose melphalan and ASCT. Post-ASCT, there are three pathways.

1. A phase III discontinuation design to assess de-escalating therapy in standard-risk MRD-negative patients. Participants receive 12 cycles of Isa maintenance. Those who remain MRD-negative are randomised to either continue or stop treatment.
2. A phase II/III multi-arm multi-stage design to test treatment strategies for treatment escalation in standard-risk MRD-positive patients. Participants are randomised to either; R, RBorD(x4) +R, RIsa, or RBorIsaD(x4) + RIsa.
3. A phase II design to assess the activity of intensive treatment strategies in high-risk patients. Participants are randomised to RBorD(x4) +R or RBorIsaD(x4) + RIsa.

1400 participants will be registered to allow for 500, 450 and 172 participants in each pathway. Randomisations are equal and treatment is given until disease progression or intolerance.

Ethics and dissemination

Ethical approval was granted by the London–Central Research Ethics Committee (20/LO/0238) and capacity and capability confirmed by the appropriate local research and development department for each participating centre prior to opening recruitment. Participant informed consent is required before trial registration and re-confirmed post-ASCT. Results will be disseminated by conference presentations and peer-reviewed publications.

Trial registration

ISCRTN 46841867

Key Words: Newly Diagnosed, Multiple Myeloma, Transplant-eligible, Risk-adapted, Treatment escalation, Treatment de-escalation

For peer review only

Article Summary

Strengths and limitations of the study

Strengths

- RADAR trial treatment is personalised through the utilisation of baseline cytogenetic and dynamic MRD-based stratification within its design
- RADAR is adaptive, new experimental treatment arms are expected to be added as new evidence emerges.
- RADAR has the potential to address an unmet need in post-ASCT treatment of multiple myeloma patients.
- RADAR includes the evaluation of a de-escalation strategy which will determine whether patients responding to treatment need to continue treatment until progression.

Limitations

- Patients achieving <PR to induction therapy will not proceed with on-study ASCT and will be treated off -trial, there is no trial pathway for these participants.
- RADAR excludes patients with impaired renal impairment (defined as CrCl <30 ml/min)
- The definition of high-risk used in RADAR may not be aligned to other international definitions, which may limit generalisability of outcomes

For peer review only

Introduction

Multiple Myeloma

Multiple myeloma (MM) is the second most common haematological malignancy with over 5500 patients diagnosed in the UK each year.[1] Whilst there is no established cure, survival has extended markedly in the last 15 years such that patients diagnosed today can expect to live a median of 6 years, and 30% will live for 10 years or longer.[1, 2]

These advances have been made possible due to the now standard use of "novel agents" including proteasome inhibitors and immunomodulatory agents (IMiD® agents) in conjunction with high-dose melphalan (HDM) supported by autologous stem cell transplantation (ASCT). Whilst MM is predominantly a disease of older people, around 40% of patients are young and fit enough at diagnosis to be eligible for ASCT, a strategy shown in several randomised trials (RCT) to extend progression-free survival (PFS).[2-4]

Improved response rates and increased survival poses challenges for the evaluation of new regimens in a timely manner. Recent studies re-evaluating the role of ASCT in the era of novel agents have confirmed superior PFS in patients allocated to ASCT compared to those allocated to chemotherapy with deferred ASCT.[5] No overall survival (OS) benefit has been seen yet, although this probably reflects the frequent use of salvage ASCT at second remission in most trial patients. Such studies utilise post-ASCT continuous therapy (lenalidomide maintenance) [6] that can extend PFS and OS,[7] but may also impact on quality of life (partly due to treatment-related toxicity), and are not without long-term risk.[8] It is thus imperative to identify those patients who are likely to benefit from extended maintenance therapy, in order to reduce health care costs and minimise toxicity for those who do not benefit.

Existing Evidence: Induction Therapy

Treatment with lenalidomide (R), cyclophosophamide (Cy), bortezomib (Bor) and dexamethasone (D) (RCyBorD) was studied as first line therapy in the Phase 2 EVOLUTION study, where it produced high response rates and deep responses compared to CyBorD or LenBorD.[9]

Existing Evidence: High-Risk Patients

At diagnosis, a small subgroup of patients (approximately 20%) can be identified on the basis of genetic features to have inferior survival regardless of treatment regimen (high-risk patients).[10-12] For such patients, and for those patients with refractory disease (no response to first line therapy), outcomes have remained poor despite the introduction of novel agents[13-15]. Whilst high-risk patients often show a good response to induction therapy, the durability of the response is inferior to standard-risk patients. Using the R-ISS which incorporates high risk cytogenetic features into the the International Staging System (ISS), PFS time is 66 months for R-ISS stage I, 42 months for R-ISS stage II, and 29 months for R-ISS stage III)[16]. The attainment of MRD-negative disease is also critical.[17-19] The activity of the RBorD regimen in patients with high-risk disease looks promising[20], and there is a question of whether an additional benefit may be gained through the use of anti-CD38 immunotherapy. Anti-CD38[21, 22] antibodies have single agent activity, but also impressive efficacy in combination, particulary with IMiD agents, lenalidomide and pomalidomide. In the RADAR trial the anti-CD38 antibody isatuximab (Isa) (SAR650984) will be used. Isatuximab is a naked immunoglobulin

For peer review only - http://bmjopen.bmj.com/site/about/guidelines.xhtml

(Ig) G1 derived monoclonal antibody (mAb) that binds selectively to a unqiue epitope on the human surface antigen called CD38 [23].

Existing Evidence: Standard-Risk Patients

With increasingly effective multi-drug regimens being used for induction treatment, and responses further deepened following high-dose therapy and ASCT, the major challenge for standard-risk participants is how to maintain their disease control for as long as possible. The benefit of post-ASCT consolidation approaches remains controversial, with inconsistent evidence identifying both appropriate patient groups and treatment strategies.

Maintenance lenalidomide (R) has been studied within several large studies and has consistently been shown to increase PFS, confimed within a recent meta-analysis.[7] The impact of long-term lenalidomide therapy on bone marrow and immune function, and general wellbeing are not completely understood. Thus there is an urgent need to identify those patients who benefit most from this approach, as well as an evaluation of other agents that utilise the host immune system to improve outcomes, such as antibodies. It is also of benefit to explore the option and effect of stopping maintenance therapy in patients who have ahcieved and maintained the deepest response

Existing Evidence: Use of MRD for stratifying treatment in standard-risk patients

MRD status is a clinically relevant biomarker, and an emerging clinical trial endpoint with recent guidance published by the International Myeloma Working Group (IMWG).[19, 24]

Several studies have shown that patients who have detectable disease in their bone marrow (MRD-positive) following ASCT suffer earlier relapses and shorter survival when compared with those who are MRD-negative.[25, 26] These results have been combined in a recent meta-analysis.[26] Hence these patients should be the focus of investigative strategies aimed at deepening disease response and converting them from MRD-positive to MRD-negative.

Patients without detectable disease in their bone marrow (MRD-negative), have longer PFS and OS .[27] In the IFM2009 study that randomised patients to receive ASCT upfront or to continue chemotherapy, patients who were MRD-negative fared equally well, regardless of whether they had received ASCT, or chemotherapy alone. This would suggest that those who have achieved MRD-negative response may not need to undergo intensive consolidation therapy, and instead may benefit from a de-escalation of therapy.

Aims and objectives

RADAR aims to address the most important contemporary therapeutic questions for newly diagnosed multiple myeloma transplant eligible (NDMM TE) patients post-ASCT by considering three different post-ASCT treatment pathways:

1. A de-escalation treatment strategy is used to determine whether ceasing Isa maintenance treatment, after 12 cycles of continuous treatment, is non-inferior to long-term Isa treatment, in terms of progression-free survival (PFS) in participants who are standard-risk at diagnosis (participants who have ≤1 high-risk cytogenetic lesion) and are MRD-negative at 100 days post-ASCT and 12 months (cycles) after.

For peer review only - http://bmjopen.bmj.com/site/about/guidelines.xhtml

2. A treatment escalation strategy is used for participants who are standard-risk at diagnosis (participants who have ≤1 high-risk cytogenetic lesion) and MRD-positive at 100 days post ASCT. The trial aims to compare the activity and efficacy of post-ASCT consolidation and maintenance treatment between combinations of R, Bor, D and Isa to R alone in terms of conversion to MRD-negativity.
3. Dose-intensive consolidation and maintenance strategies will be studied for participants who are high-risk at diagnosis (participants who have >1 high-risk cytogenetic lesion). The activity of post-ASCT strategies containing R, Bor, D and Isa will be assessed in terms of PFS rate.

Trial Design

RADAR is a national, multi-centre, risk-adapted, response-guided open-label, randomised controlled trial for NDMM TE patients: phase II/III for standard-risk, MRD-Positive participants, phase III randomised discontinuation design for standard-risk, MRD-Negative participants, phase II randomised for high-risk participants. The following report details the trial protocol and follows the structure of the SPIRIT statement.[28] The SPIRIT checklist can be found within the supplementary material.

Methods:

Setting

The RADAR trial will be conducted at 70 centres in the United Kingdom (See supplementary material), as identified via a feasibility assessment to determine those most appropriate to participate in the trial.

Potential participants will be identified by the research team at the time they are referred to the haematology outpatient department with suspected multiple myeloma. A smaller number of participants may be identified during inpatient admissions. Invitation to participate in the trial and provision of information will be made either during their first consultation, when routine diagnostic tests will be performed and potential treatment options discussed, or at the time they receive their diagnostic test results.

Eligibility criteria

Adults (18 years and older) with previously untreated multiple myeloma requiring therapy, as defined by IMWG diagnostic criteria, who are eligible for stem cell transplant and capable of giving informed consent will be assessed for eligibility. Eligibility will be confirmed prior to; registration, the start of isatuximab maintenance treatment in the Randomisation 1 pathway and each randomisation by the principal investigator (PI) or an appropriately authorised medically trained delegate and will be recorded in the participants' medical records and on the relevant electronic case report form (eCRF).

To be eligible for each point in the trial the participants must meet all the inclusion criteria and none of the exclusion criteria presented in Table 1 for trial registration, Table 2 for isatuximab treatment in the R1 pathway, Table 3 for Randomisation 1 (R1), Table 4 for Randomisation 2 (R2), Table 5 for Randomisation 3 (R3).

For peer review only - http://bmjopen.bmj.com/site/about/guidelines.xhtml

Table 1: Inclusion and Exclusion Criteria for Main Trial Registration

Inclusion Criteria:

1. Previously untreated patients with multiple myeloma requiring therapy, defined as having myeloma defining events or with biomarkers of malignancy according to IMWG diagnostic criteria
2. Eligible for stem cell transplant
3. Eastern Cooperative Oncology Group (ECOG) performance status 0–2 (except in cases where ECOG >2 is due to effects of myeloma eg spinal cord compression);
4. Total bilirubin ≤ 1.5 x upper limit of normal (ULN)
5. Alanine aminotransferase (ALT) and/or aspartate aminotransferase (AST) ≤ 3 x ULN (if ALT **and** AST are tested, both must meet this criteria)
6. Adequate marrow function:
   - neutrophils ≥1.0 × 10^9/L (unless the participant has a known/suspected diagnosis of familial or racial neutropenia in which case an ANC ≥0.75 x 10^9/L is allowed),
   - haemaglobin (Hb) ≥ 80g/L. Blood *transfusions within 3 days prior to eligibility assessments are **not** permitted*,
   - platelets ≥ 75 × 10^9/L (in the case of heavy bone marrow infiltration (>50%) which is, in the opinion of the investigator, the cause of the thrombocytopaenia and provided appropriate supportive measures and patient monitoring are in place, a platelet count of ≥50 × 10^9/L is permitted. *Platelet transfusions within 3 days prior to eligibility assessments are **not** permitted.*
7. Creatinine clearance (CrCl) ≥ 30 mL/minute, according to the Cockcroft-Gault formula, following correction of reversible causes (e.g. dehydration, hypercalcaemia, sepsis)
8. Able to swallow oral medication
9. Aged at least 18 years
10. Agree to follow the pregnancy prevention guidelines:

    Female participants who:

    a) Are not of childbearing potential (Appendix 7), OR

    b) If they are of childbearing potential, agree to practice 2 effective methods of contraception, at the same time, from the time of signing the informed consent form until 12 months after the last dose of cyclophosphamide or 5 months after the last dose of study drug, whichever is longest, OR

    c) Agree to practice true abstinence when this is in line with the preferred and usual lifestyle of the subject from the time of signing the informed consent form until 12 months after the last dose of cyclophosphamide or 5 months after the last dose of study drug, whichever is longest. (Periodic abstinence [e.g. calendar, ovulation, symptothermal, post-ovulation methods] and withdrawal are not acceptable methods of contraception.)

    d) Agree to not donate oocytes during the entire study treatment period and until 12 months after the last dose of cyclophosphamide or 5 months after the last dose of study drug, whichever is longest.

    Male participants, even if surgically sterilised (i.e. status post-vasectomy), must agree to one of the following:

    a) Agree to practice effective barrier contraception during the entire study treatment period and until 6 months after the last dose of cyclophosphamide or 5 months after the last dose of study drug, whichever is longest OR

    b) Agree to practice true abstinence when this is in line with the preferred and usual lifestyle of the subject, during the entire study treatment period and until 6 months after the last dose of cyclophosphamide or 5 months after the last dose of study drug, whichever is longest. (Periodic abstinence (e.g. calendar, ovulation, symptothermal, post-ovulation methods] and withdrawal are not acceptable methods of contraception).

    c) Agree to not donate sperm during the entire study treatment period and until 6 months after the last dose of cyclophosphamide or 5 months after the last dose of study drug, whichever is longest.

    Contraception for female and male participants must be in accordance with (and participants must consent to) the Celgene Pregnancy Prevention Plan.

    If female and of childbearing potential, they must have a negative pregnancy test performed by a healthcare professional within 14 days prior to registration.
11. Able to provide written informed consent.

Exclusion Criteria

1. Asymptomatic (smouldering) MM, monoclonal gammopathy of undetermined significance (MGUS), solitary plasmacytoma of bone, or extramedullary plasmacytoma (without evidence of MM)

For peer review only - http://bmjopen.bmj.com/site/about/guidelines.xhtml

2. Received previous treatment for MM, with the exception of local radiotherapy to relieve bone pain or spinal cord compression, prior bisphosphonate treatment, or corticosteroids as long as the total dose does not exceed the equivalent of 160mg dexamethasone. *This criteria is not applicable at R1, R2 and R3 when participants will have received previous treatment for MM as part of this trial.*
3. Unstable angina or myocardial infarction within 4 months prior to registration (or at any time since registration for participants starting isatuximab maintenance, R1, R2 and R3), NYHA Class III or IV heart failure, uncontrolled angina, history of severe coronary artery disease, severe uncontrolled ventricular arrhythmias, sick sinus syndrome, or electrocardiographic evidence of acute ischemia or Grade 3 conduction system abnormalities unless subject has a pacemaker
4. Cardiac disorder identified according to local practice (eg left ventricular ejection fraction, LVEF; results from formal measurements acceptable within 28 days prior to registration)
5. Significant neuropathy (Grade ≥3, or Grade 2 with pain)
6. Prior malignancy that required treatment or has shown evidence of recurrence (except for non-melanoma skin cancer or adequately treated cervical carcinoma in situ) during the 5 years prior to registration. Cancer treated with curative intent for >5 years previously and without evidence of recurrence will be allowed.
7. Pregnant, lactating or breastfeeding female participants
8. Known resistance, intolerance or hypersensitivity to any component of the planned therapies, except in the case of hypersensitivity which is amenable to premedication with steroids or H2 blocker. Intolerance includes hereditary problems of galactose intolerance, the Lapp lactase deficiency or glucose-galactose malabsorption.
9. Major surgery within 14 days before registration (or starting isatuximab maintenance, R1, R2 and R3). This would include surgical intervention for relief of cord compression but does not include vertebroplasty or kyphoplasty.
10. Known gastrointestinal (GI) disease or GI procedure that could interfere with the oral absorption or tolerance of trial treatment, including difficulty swallowing.
11. Active systemic infection
12. Participant is hepatitis B surface antigen positive, hepatitis C antibody positive or HIV positive (participants who are hepatitis C core antibody positive but have been successfully treated for the disease may still be eligible – please consult the CTRU/CI). Participants must have hepatitis and HIV screening conducted within 28 days prior to registration.
13. Any other medical or psychiatric condition which, in the opinion of the investigator, contraindicates the participant's participation in this study.
14. Receipt of live vaccination within 30 days prior to registration, for the duration of the study and for 3 months after the last dose of study drug.
15. Participant has risk factors for thromboembolism including the use of agents which may increase their risk of thrombosis, such as hormone replacement therapy (*this exclusion criteria is applicable only at registration and when starting R2 or R3 pathway*)
16. Participant has risk factors for seizures (*this exclusion criteria is applicable only at registration and when starting R2 or R3 pathway*

For peer review only

For peer review only - http://bmjopen.bmj.com/site/about/guidelines.xhtml

Table 2: Inclusion and Exclusion criteria for Isatuximab treatment (R1 Pathway)

Inclusion Criteria:

1. Standard-risk (participant is not **confirmed** to have at least two of these genetically adverse lesions: t(4;14), t(14;16), t(14;20), del(17p), gain(1q), as confirmed by the CTRU.
2. 4 cycles of RCyBorD received
3. MRD-negative (proportion of malignant cells in the bone marrow is < 1 in 100,000, confirmed by HMDS central lab) at 100 days post-ASCT
4. Received ≥100mg/m^2 high-dose melphalan and ASCT
5. Eastern Cooperative Oncology Group (ECOG) performance status 0–2 (except in cases where ECOG >2 is due to effects of myeloma eg spinal cord compression)
6. Total bilirubin <= 1.5 x upper limit of normal (ULN)
7. Alanine aminotransferase (ALT) and/or aspartate aminotransferase (AST) ≤ 3 x ULN (if ALT **and** AST are tested, both must meet this criteria)
8. Adequate marrow function:
   a) neutrophils ≥1.0 × 10^9/L (unless the participant has a known/suspected diagnosis of familial or racial neutropenia in which case an ANC ≥0.75 x 10^9/L is allowed),
   b) Hb≥ 80g/L. Blood transfusions within 3 days prior to eligibility assessments are not permitted,
   c) platelets ≥ 75 × 10^9/L. Platelet transfusions within 3 days prior to eligibility assessments are not permitted
9. Creatinine clearance (CrCl) ≥ 30 mL/minute, according to the Cockcroft-Gault formula, following correction of reversible causes (e.g. dehydration, hypercalcaemia, sepsis)
10. Agree to follow the pregnancy prevention guidelines:
    Female participants who:
    a) Are not of childbearing potential, OR
    b) If they are of childbearing potential, agree to practice 2 effective methods of contraception, at the same time, from the time of signing the informed consent form until 12 months after the last dose of cyclophosphamide or until 5 months after the last dose of study drug, whichever is longest OR
    c) Agree to practice true abstinence when this is in line with the preferred and usual lifestyle of the subject, from the time of signing the informed consent form until 12 months after the last dose of cyclophosphamide or until 5 months after the last dose of study drug, whichever is longest. (Periodic abstinence [e.g. calendar, ovulation, symptothermal, post-ovulation methods] and withdrawal are not acceptable methods of contraception.)
    d) Agree to not donate oocytes during the entire study treatment period until 12 months after the last dose of cyclophosphamide or 5 months after the last dose of study drug, whichever is longest.
    Male participants, even if surgically sterilised (i.e. status post-vasectomy), must agree to one of the following:
    e) Agree to practice effective barrier contraception during the entire study treatment period and until 6 months after the last dose of cyclophosphamide or until 5 months after the last dose of study drug, whichever is longest OR
    f) Agree to practice true abstinence when this is in line with the preferred and usual lifestyle of the subject during the entire study treatment period and until 6 months after the last dose of cyclophosphamide or until 5 months after the last dose of study drug, whichever is longest.. (Periodic abstinence [e.g. calendar, ovulation, symptothermal, post-ovulation methods] and withdrawal are not acceptable methods of contraception).
    g) Agree to not donate sperm during the entire study treatment period until 6 months after the last dose of cyclophosphamide or 5 months after the last dose of study drug, whichever is longest.
11. Signed the Informed Consent Document for the R1 treatment pathway.
    a)

Exclusion Criteria:

1. Disease progression (according to IMWG criteria)
2. MRD-positive (proportion of malignant cells in the bone marrow is ≥ 1 in 100,000, confirmed by HMDS central lab) at 100 days post-ASCT
3. Registration exclusion criteria (cardiac risk assessment, HIV and Hepatitis B and C testing do not need to be repeated)

For peer review only - http://bmjopen.bmj.com/site/about/guidelines.xhtml

Table 3: Inclusion and Exclusion criteria for Randomisation 1

Inclusion Criteria:

1. Standard-risk (participant is not **confirmed** to have at least two of these genetically adverse lesions: t(4;14), t(14;16), t(14;20), del(17p), gain(1q), as confirmed by the CTRU.
2. 12 cycles of isatuximab maintenance received
3. MRD-negative (proportion of malignant cells in the bone marrow is < 1 in 100,000, confirmed by HMDS central lab) at 100 days post-ASCT
4. MRD-negative (proportion of malignant cells in the bone marrow is < 1 in 100,000, confirmed by HMDS central lab) after 12 cycles of isatuximab
5. Received ≥100mg/m^2 high-dose melphalan and ASCT
6. Eastern Cooperative Oncology Group (ECOG) performance status 0–2 (except in cases where ECOG >2 is due to effects of myeloma eg spinal cord compression)
7. Total bilirubin <= 1.5 x upper limit of normal (ULN)
8. Alanine aminotransferase (ALT) and/or aspartate aminotransferase (AST) ≤ 3 x ULN (if ALT and AST are tested, both must meet this criteria)
9. Adequate marrow function:
   - neutrophils ≥1.0 × 10^9/L (unless the participant has a known/suspected diagnosis of familial or racial neutropenia in which case an ANC ≥0.75 x 10^9/L is allowed),
   - Hb≥ 80g/L. Blood transfusions within 3 days prior to eligibility assessments are not permitted,
   - platelets ≥ 75 × 10^9/L. Platelet transfusions within 3 days prior to eligibility assessments are not permitted
10. Creatinine clearance (CrCl) ≥ 30 mL/minute, according to the Cockcroft-Gault formula, following correction of reversible causes (e.g. dehydration, hypercalcaemia, sepsis)
11. Agree to follow the pregnancy prevention guidelines:

    Female participants who:

    a) Are not of childbearing potential, OR
    b) If they are of childbearing potential, agree to practice 2 effective methods of contraception, at the same time, from the time of signing the informed consent form until 5 months after the last dose of study drug, OR
    c) Agree to practice true abstinence when this is in line with the preferred and usual lifestyle of the subject from the time of signing the informed consent form until 5 months after the last dose of study drug. (Periodic abstinence [e.g. calendar, ovulation, symptothermal, post-ovulation methods] and withdrawal are not acceptable methods of contraception.)
    d) Agree to not donate oocytes during the entire study treatment period and until 5 months after the last dose of study drug.

    Male participants, even if surgically sterilised (i.e. status post-vasectomy), must agree to one of the following:

    a) Agree to practice effective barrier contraception during the entire study treatment period and until 5 months after the last dose of study drug, OR
    b) Agree to practice true abstinence when this is in line with the preferred and usual lifestyle of the subject during the entire study treatment period and until 5 months after the last dose of study drug. (Periodic abstinence [e.g. calendar, ovulation, symptothermal, post-ovulation methods] and withdrawal are not acceptable methods of contraception).
    c) Agree to not donate sperm during the entire study treatment period and until 5 months after the last dose of study drug.
12. Signed the Informed Consent Document for the R1 treatment pathway.

Exclusion Criteria

1. Disease progression (according to IMWG criteria)
2. MRD-positive (proportion of malignant cells in the bone marrow is ≥ 1 in 100,000, confirmed by HMDS central lab) at 100 days post-ASCT or after 12 cycles of isatuximab
3. Registration exclusion criteria (cardiac risk assessment, HIV and Hepatitis B and C testing do not need to be repeated)

For peer review only

For peer review only - http://bmjopen.bmj.com/site/about/guidelines.xhtml

Table 4: Inclusion and Exclusion Criteria for Randomisation 2

Inclusion Criteria:

1. Standard-risk (participant is not **confirmed** to have at least two of these genetically adverse lesions: t(4;14), t(14;16), t(14;20), del(17p), gain(1q) as confirmed by CTRU.
2. 4 cycles of RCyBorD received
3. MRD-positive (proportion of malignant cells in the bone marrow is ≥ 1 in 100,000, confirmed by HMDS central lab) at 100 days post-ASCT
4. Received ≥100mg/m^2 high-dose melphalan and ASCT
5. Eastern Cooperative Oncology Group (ECOG) performance status 0–2 (except in cases where ECOG >2 is due to effects of myeloma eg spinal cord compression)
6. Total bilirubin <= 1.5 x upper limit of normal (ULN)
7. Alanine aminotransferase (ALT) and/or aspartate aminotransferase (AST) ≤ 3 x ULN (if ALT and AST are tested, both must meet this criteria)
8. Adequate marrow function:
   - neutrophils ≥1.0 × 10^9/L (unless the participant has a known/suspected diagnosis of familial or racial neutropenia in which case an ANC ≥0.75 x 10^9/L is allowed),
   - Hb≥ 80g/L
   - platelets ≥ 75 × 10^9/L. Platelet transfusions within 3 days prior to eligibility assessments are not permitted
9. Creatinine clearance (CrCl) ≥ 30 mL/minute, according to the Cockcroft-Gault formula, following correction of reversible causes (e.g. dehydration, hypercalcaemia, sepsis)
10. Agree to follow the pregnancy prevention guidelines:

    Female participants who:

    a) Are not of childbearing potential, OR

    b) If they are of childbearing potential, agree to practice 2 effective methods of contraception, at the same time, from the time of signing the informed consent form until 12 months after the last dose of cyclophosphamide or 5 months after the last dose of study drug, whichever is longest OR

    c) Agree to practice true abstinence when this is in line with the preferred and usual lifestyle of the subject, from the time of signing the informed consent form until 12 months after the last dose of cyclophosphamide or 5 months after the last dose of study drug, whichever is longest. (Periodic abstinence [e.g. calendar, ovulation, symptothermal, post-ovulation methods] and withdrawal are not acceptable methods of contraception.)

    d) Agree to not donate oocytes during the entire study treatment period and until 12 months after the last dose of cyclophosphamide or 5 months after the last dose of study drug, whichever is longest.

    Male participants, even if surgically sterilised (i.e. status post-vasectomy), must agree to one of the following:

    a) Agree to practice effective barrier contraception during the entire study treatment period and until 6 months after the last dose of cyclophosphamide or 5 months after the last dose of study drug, whichever is longest OR

    b) Agree to practice true abstinence when this is in line with the preferred and usual lifestyle of the subject during the entire study treatment period and until 6 months after the last dose of cyclophosphamide or 5 months after the last dose of study drug, whichever is longest. (Periodic abstinence [e.g. calendar, ovulation, symptothermal, post-ovulation methods] and withdrawal are not acceptable methods of contraception).

    c) Agree to not donate sperm during the entire study treatment period and until until 6 months after the last dose of cyclophosphamide or 5 months after the last dose of study drug, whichever is longest.
11. Signed the Informed Consent Document for the R2 treatment pathway.

Exclusion Criteria:

1. Disease progression (according to IMWG criteria)
2. Registration exclusion criteria (cardiac risk assessment, HIV and Hepatitis B and C testing do not need to be repeated)

For peer review only - http://bmjopen.bmj.com/site/about/guidelines.xhtml

Table 5: Inclusion and Exclusion criteria for Randomisation 3

Inclusion Criteria:

1. High-risk (participant is **confirmed** to have at least two of these genetically adverse lesions: t(4;14), t(14;16), t(14;20), del(17p), gain(1q)) as confirmed by CTRU.
2. 4 cycles of RCyBord received
3. Received ≥100mg/m^2 high-dose melphalan and ASCT
4. Eastern Cooperative Oncology Group (ECOG) performance status 0–2 (except in cases where ECOG >2 is due to effects of myeloma eg spinal cord compression)
5. Total bilirubin <= 1.5 x upper limit of normal (ULN)
6. Alanine aminotransferase (ALT) and/or aspartate aminotransferase (AST) ≤ 3 x ULN (if ALT and AST are tested, both must meet this criteria)
7. Adequate marrow function:
   - neutrophils ≥1.0 × 10^9/L (unless the participant has a known/suspected diagnosis of familial or racial neutropenia in which case an ANC ≥0.75 x 109/L is allowed),
   - Hb≥ 80g/L
   - platelets ≥ 75 × 10^9/L. Platelet transfusions within 3 days prior to eligibility assessments are not permitted
   - Creatinine clearance (CrCl) ≥ 30 mL/minute, according to the Cockcroft-Gault formula, following correction of reversible causes (e.g. dehydration, hypercalcaemia, sepsis)
8. Creatinine clearance (CrCl) ≥ 30 mL/minute, according to the Cockcroft-Gault formula, following correction of reversible causes (e.g. dehydration, hypercalcaemia, sepsis)
9. Agree to follow the pregnancy prevention guidelines:

   Female participants who:

   a) Are not of childbearing potential, OR
   b) If they are of childbearing potential, agree to practice 2 effective methods of contraception, at the same time, from the time of signing the informed consent form until 12 months after the last dose of cyclophosphamide or 5 months after the last dose of study drug, whichever is longest OR
   c) Agree to practice true abstinence when this is in line with the preferred and usual lifestyle of the subject from the time of signing the informed consent form until 12 months after the last dose of cyclophosphamide or 5 months after the last dose of study drug, whichever is longest. (Periodic abstinence [e.g. calendar, ovulation, symptothermal, post-ovulation methods] and withdrawal are not acceptable methods of contraception.)
   d) Agree to not donate oocytes during the entire study treatment period and until 12 months after the last dose of cyclophosphamide or 5 months after the last dose of study drug, whichever is longest.

   Male participants, even if surgically sterilised (i.e. status post-vasectomy), must agree to one of the following:

   a) Agree to practice effective barrier contraception during the entire study treatment period and until 6 months after the last dose of cyclophosphamide or 5 months after the last dose of study drug, whichever is longest OR
   b) Agree to practice true abstinence when this is in line with the preferred and usual lifestyle of the subject during the entire study treatment period and until 6 months after the last dose of cyclophosphamide or 5 months after the last dose of study drug, whichever is longest. (Periodic abstinence [e.g. calendar, ovulation, symptothermal, post-ovulation methods] and withdrawal are not acceptable methods of contraception).
   c) Agree to not donate sperm during the entire study treatment period and until 6 months after the last dose of cyclophosphamide or 5 months after the last dose of study drug, whichever is longest.
10. Signed the Informed Consent Document for the R3 treatment pathway.

Exclusion Criteria

1. Disease progression (according to IMWG criteria)
2. Registration exclusion criteria (cardiac risk assessment, HIV and Hepatitis B and C testing do not need to be repeated)

For peer review only - http://bmjopen.bmj.com/site/about/guidelines.xhtml

Interventions and Dosing

Intervention Schedule

All participants will receive four, 21 day, cycles of induction treatment with RCyBorD. During this time, the genetics risk status of the participant will be analysed and determined from bone marrow samples prior induction treatment (from diagnosis or trial-specific procedures). Participants will either be allocated as high risk (defined as the presence of at least 2 of these adverse lesions: t(4,14), t(14,16(, t(14,20), del(17p) or gain(1q)) or standard risk. At the end of the induction cycle participants will be assessed for response In the absence of disease progression or intolerance, participants with at least a partial response (PR) to will proceed to HDM and ASCT which will be carried out according to local practice. At 100 days post-ASCT participants will be assessed for MRD and response and will follow one of three pathways of post ASCT treatment, provided they meet the eligibility criteria:

1. Participants identified as standard risk and who are MRD-negative post-ASCT will be treated according to the R1 pathway. All participants will receive 12 cycles of Isa maintenance treatment. Those who remain MRD-negative following cycle 12, will be randomised to continue or stop Isa maintenance treatment.
2. Participants identified as standard-risk and who are MRD-positive post-ASCT and have at least a minimal response (MR) at 100 days post ASCT, will proceed to R2. Eligible participants will be randomised between: R maintenance; RBorD consolidation and R maintenance (RBorD + R); RISa maintenance; or RBorIsaD consolidation and RIsa maintenance (RBorIsaD + RIsa).
3. Participants identified as high-risk and have at least a partial response (PR) at 100 days post ASCT will proceed to R3. These participants will be randomised to receive either RBorD consolidation, followed by R maintenance; or RBorIsaD consolidation followed by RIsa maintenance.

All randomisations are balanced across the treatment arms. A computer generated minimisation programme that incorporates a random element will be used to ensure treatment groups are well balanced for the stratification factors of each randomisation (See Table 6).

Table 6: Stratification Factors

Randomisation:	Stratification factors:
1	• Centre<br>• Number of high risk lesions at trial registration (0 or 1 or unable to determine)
2	• Centre<br>• Number of high risk lesions at trial registration (0 or 1 or unable to determine)<br>• Response at 100 days post-ASCT ($< VGPR, \geq VGPR$)
3	• Centre<br>• Number of high risk lesions at trial registration (2, or ≥ 3 , or unable to determine)<br>• Response at 100 days post-ASCT ($< VGPR, \geq VGPR$)

For peer review only - http://bmjopen.bmj.com/site/about/guidelines.xhtml

Table 7 summarises the starting doses for the induction treatment and each post-ASCT treatment pathway. Following ASCT all treatments will continue until disease progression or intolerance. Response will be assessed at the end of each cycle according to the IMWG 2016 response criteria[24, 29-31]. Treatment intolerance will be assessed throughout each treatment cycle, according to the national cancer institute (NCI) common terminology criteria for AE (CTCAE) Version 5. All consolidation cycles are 21 days in length, maintenance cycles are 28 days in length.

Table 7: Dosing schedule for RCyBorD induction and each randomisation pathways

**Trial Registration- Induction (Each cycle = 21 days)**		
**Treatment (Route)**	**Induction – All participants (Starting Dose)**	**Days**
**Bortezomib (SC)**	1.3mg/m^2	1, 8, 15
**Cyclophosphamide (PO)**	500mg	1, 8
**Lenalidomide (PO)**	25mg	1-14
**Dexamethasone (PO)**	40mg	1, 8, 15
**Isatuximab Pre-Randomisation 1 (Each cycle = 28 day)**		
**Treatment (Route)**	**R1 pathway participants Only (Starting Dose)**	**Days**
**Isatuximab (IV)**	10mg/kg	1, 8, 15 and 22 cycle 1 then days 1 and 15 from cycle 2 onwards
**Randomisation 1: Isatuximab maintenance (Each Cycle = 28 days)**		
**Treatment**	**Isatuximab (Days)**	**"Stop Isatuximab" (Days)**
**Isatuximab (IV)**	10mg/kg (Day 1 Only)	None
**Randomisation 2: MRD-positive treatment escalation pathway**		
**Treatment Arm 1 (Control) : Lenalidomide (R) maintenance**		
**Treatment (Route)**	**Starting Dose (Days)**	
**Lenalidomide (PO)**	10mg/kg* (1-21)	
**Treatment Arm 2: RBorD consolidation + R Maintenance**		
**Treatment**	**Consolidation: Cycle 1-4 (each cycle = 21 days)**	**Maintenance (each Cycle = 28 days)**
**Bortezomib (SC)**	1.3mg/m^2 (1, 8, 15)	NA
**Lenalidomide (PO)**	15mg* (1-14)	10mg* (1-21)
**Dexamethasone (PO)**	20mg (1, 8, 15)	NA
**Treatment Arm 3:RISa Maintenance**		
**Treatment**	**Maintenance (Days)**	
**Lenalidomide (PO)**	10mg* (Day 1-21)	
**Isatuximab (IV)**	10mg/kg (1, 8, 15 and 22 cycle 1 then days 1 and 15 from cycle 2 onwards)	
**Treatment Arm 4: RBorIsaD + RIsa**		
Treatment	Consolidation Cycle 1-4: Each cycle = 21 days (Days)	Maintenance: Each cycle = 28 days (Days)
**Bortezomib (SC)**	1.3mg/m^2 (1, 8, 15)	NA
**Lenalidomide (PO)**	15mg* (1-14)	10mg* (1-21)
**Isatuximab (IV)**	10mg/kg (1, 8, 15 cycle 1 then days 1 and 8 cycle 2, then days 1 and 15 cycle 3 onwards)	10mg/kg (1 and 15)
**Dexamethasone (PO)**	20mg (1, 8, 15)	NA
**Randomisation 3: High Risk Treatment Pathway**		
**Treatment Arm 1: RBorD Consolidation and R maintenance**		
**Treatment**	**Consolidation: Cycle 1-4 (each cycle = 21 days)**	**Maintenance (each Cycle = 28 days)**
**Bortezomib (SC)**	1.3mg/m^2 (1, 8, 15)	NA
**Lenalidomide (PO)**	15mg* (1-14)	10mg* (1-21)

For peer review only - http://bmjopen.bmj.com/site/about/guidelines.xhtml

**Dexamethasone (PO)**	20mg (1, 8, 15)	NA
**Treatment Arm 2: RBorIsaD consolidation + RIsa Maintenance**		
**Treatment**	**Consolidation Cycle 1-4: Each cycle = 21 days (Days)**	**Maintenance: Each cycle = 28 days (Days)**
**Bortezomib (SC)**	1.3mg/m^2 (1, 8, 15)	NA
**Lenalidomide (PO)**	15mg* (1-14)	10mg* (1-21)
**Isatuximab (IV)**	10mg/kg (1, 8, 15 cycle 1 then days 1 and 8 cycle 2, then days 1 and 15 cycle 3 onwards)	10mg/kg (1 and 15)
**Dexamethasone (PO)**	20mg (1, 8, 15)	NA

*or final dose administrated at the end of induction or consolidation treatment if lower

Intervention Adherence

Throughout the trial R will be taken orally and swallowed whole at the same time on the scheduled days. D and Cy will be taken orally, whilst Bor will be administrated via a subcutaneous injection. Bortezomib may also be administered at home if this is in line with local policies and if appropriate procedures are in place. Isa will be given as an intravenous infusion and at a facility capable of managing hypersensitivity reactions.

To monitor adherence to the oral trial medicines, participants will complete a participant diary card which will be reviewed at each trial visit. Non-compliance will be reported to the CTRU and any unused capsules will be returned to the pharmacy.

Dose Modifications and Discontinuations

With the exception of Isa, which can only be interrupted and resumed, dose modifications are permitted in the management of toxicities. Upfront dose modifications in response to liver and/or renal impairment are also permitted within the protocol. When the dose of any drug is reduced the dose cannot be re-escalated in this trial.

If one drug in a combination is stopped, treatment with the other drugs in combination can continue. Scenarios in which the participant may remain on trial following consultation with the Chief Investigator (CI) are if they: discontinue two or more drugs during combination therapy; cease treatment for more than three weeks in between any treatment cycle or are delayed in starting maintenance of consolidation beyond 120 days post-ASCT. Note that if Bor, R or D is stopped during the pre-ASCT induction treatment due to toxicity, the participant will only be able to be treated on the standard-risk-MRD-negative pathway (assuming they meet the relevant inclusion criteria).

If treatment is discontinued early, the participant will be treated off trial at the discretion of their treating clinician.

Concomitant medication:

Local support care protocols, including anti-emetic schedules, tumour lysis syndrome prevention, venous thromboembolism prophylaxis and prophylactic antimicrobial therapy should be followed. The excluded concomitant medications and procedures whilst receiving trial treatment are listed in supplementary material.

For peer review only - http://bmjopen.bmj.com/site/about/guidelines.xhtml

Concomitant medication, disease and other malignancies will be recorded at eligibility.

Outcomes:

Primary outcomes

PFS-R1 is defined as the time from R1 to the time of first documented evidence of disease progression or death from any cause. Individuals who are lost to follow-up or progression-free at the time of analysis will be censored at their last known date to be alive and progression-free.

Attainment of MRD-negativity is defined as a binary endpoint. MRD-negativity will be determined at 6 months post-R2 (end of cycle 6 post-ASCT treatment for participants allocated to maintenance only strategies and end of cycle 7 post-ASCT treatment for participants allocated to maintenance and consolidation strategies) according to the IMWG 2016 response criteria.

The PFS rate is defined as the proportion of participants who are alive and progression-free 28 months post-R3.

Disease progression is defined according to the IMWG 2016 response criteria for Multiple Myeloma [24, 29-31].

Secondary outcomes:

The secondary outcomes of this trial are to assess; PFS for R2 and R3, Time to progression, Time to second PFS event (PFS2), Overall survival (OS), Survival after progression, Objective response rate (ORR), Attainment of Very Good Partial Response (≥ VGPR), Attainment of MRD negativity, Duration of MRD Negativity, Time to improved response, Time to next treatment, Treatment compliance and total amount of therapy delivered, Toxicity and Safety, including the incidence of second malignancies, Quality of life (QoL) including EORTC-QLQ-C30, EORTC-QLQ-MY20 and EQ-5D-3L and Cost effectiveness.

Exploratory and subgroup analyses

An overview of the planned exploratory and subgroup analysis can be found in the supplementary material. These include genetic and molecular analysis of participant samples conducted by the respective central laboratories, as well as additional analysis to review the infection and re-infection rates of covid-19 in participants during induction, post-ASCT and during post ASCT consolidation.

Participant's timelines

The full trial schema can be seen in Figure 1. The schedule of assessments at each time point are presented in Figure 2.

Trial Entry

Participants will enter into the trial at one of two points in their pathway, this will either be at bone marrow registration or at main trial registration.

For peer review only - http://bmjopen.bmj.com/site/about/guidelines.xhtml

Trial Consent

Participants who enter the trial at bone marrow registration will provide consent to have bone marrow samples taken and sent to central laboratories for analysis. If the participant does not have myeloma or decides to not take part in RADAR, they will have the option of consenting to their samples being used in future research.

All participants will be required to provide written informed consent for the trial prior to trial registration (for initial trial treatment and further trial information) and prior to starting their post-ASCT treatment pathway once their post-ASCT assessments have been completed (for their pathway specific treatment). Optional consent regarding QoL and healthcare resource use questionnaires and the use of samples for future research will also be obtained within the first consent process.

Consent forms are included in supplementary material.

Trial Registration

Following initial consent, participant eligibility for trial registration will be assessed. Trial samples for blood and urine will be taken for all participants and bone marrow samples will be taken for those who did not enter the trial through bone marrow registration.

Genetic Risk

Bone marrow samples from diagnosis will be analysed at time of diagnosis at local cytogenetics laboratories for FISH-defined high-risk makers. Original/copy of the FISH testing report will also be sent to CTRU where they will be reviewed by the CI/Trial management team for risk stratification. Participants will either be categorised high-risk (defined as the presence of at least 2 of the following adverse lesions: t(4;14), t(4;16), t(14:20), del(17p) or gain(1q)) or standard-risk.

Participants will commence RCyBorD treatment once a diagnosis of symptomatic myeloma has been confirmed by the treating physician, and ideally when it is known that the FISH testing has been successful.

Where risk status can be determined from the first sample participants can continue on the trial. If status cannot be confirmed and the FISH report shows a full test failure i.e. no information relating to adverse lesions is available, a second attempt will be made to collect a bone marrow sample prior to treatment starting. If partial results are obtained from the FISH sample, it may be possible that the CI/ trial management group (TMG) will be able to determine risk status using the assumption that translocations are mutually exclusive and that some markers are rare. If risk status can be determined a second bone marrow sample is not required. However, this must be confirmed by the CTRU.

If, after two bone marrow samples, the FISH results for some or all the adverse lesions are inconclusive participants risk will be determined using a combination of the results from the two samples. The most appropriate pathway will be determined by the CI. Note this may result in the participants stratification factor being 'unable to determine' at randomisation. If a second bone marrow has been requested by CTRU but the collection was not attempted, the participants will be taken off the study (after ASCT, if induction treatment was already started)

For peer review only - http://bmjopen.bmj.com/site/about/guidelines.xhtml

Induction and ASCT

Following trial registration, once all central samples have been taken and risk status confirmed, participants will receive induction treatment as described in the intervention section. In the absence of disease progression or treatment intolerance participants with at least a partial response (PR) to will proceed to HDM and ASCT.

Consenting participants will complete QoL and resource use questionnaires at the end of RCyBorD induction treatment.

All participants who go through ASCT will have a bone marrow sample taken at 100 days (+/- 14) post-ASCT. If an MRD result cannot be obtained at the first attempt, the bone marrow will be repeated once (within 28 days after 100 days post-ASCT time point). If the second attempt fails, the participant will be considered MRD-positive.

Post-ASCT Treatment

Participants who receive all 4 cycles of induction treatment followed by ASCT will be assessed for eligibility into their assigned randomisation pathways determined by genetic risk and MRD results and treated as described in the intervention schedule.

Consenting participants in the R1 pathway will complete QoL and resource use questionnaires after cycle 6, 12, 24 and 36 (participants randomised to “stop Isa” will complete the cycle 24 and 36 QoL and resource use questionnaires at equivalent time points).

Similarly, consenting participants to receive R or RIsa within pathway R2 and R3 will complete QoL and resource use questionnaires after cycles 6, 12 and 24 of maintenance. Those randomised to receive RBorIsa+R or RBorIsaD+RIsa will complete QoL and resource use questionnaires after cycles 3, 9 and 21 of maintenance.

Post-ASCT treatment (including observation only in the R1 pathway) is continued until disease progression, participant or clinician withdrawal or death. After treatment discontinuation, the participant is treated off trial by their local clinician and followed-up as per the trial follow-up schedule.

Trial Follow-up

Participants who discontinue during induction and until 100 days post-ASCT will be followed up for data pertaining to safety, details of salvage treatment, progression (including second progression), and survival at standard care visits. Follow ups will be conducted every 2 months up until disease progression, death or the end of trial. Similar follow-up will be completed for participants who discontinue their allocated trial treatment, which started post-ASCT. In addition consenting participants will still complete QoL and resource use questionnaires at appropriate time points.

All types of adverse event/reaction will be collected until 60 days after the last dose of protocol treatment.

For peer review only - http://bmjopen.bmj.com/site/about/guidelines.xhtml

For peer review only

Following disease progression all participants will be followed up annually until death, or until the end of the trial.

Sample size

In total 1400 participants will be recruited into the trial. Accounting for participants who will be lost to follow-up, this allows for 500, 450 and 172 participants to enter each randomisation, respectively.

Randomisation 1

Data from our previous trial Myeloma XI, suggests that approximately 50 participants will lose their MRD-negative status during the first 12 cycles of Isa maintenance. In addition, Myeloma XI estimated median PFS with continuous R to be 50 months amongst TE participants.[32] Under these assumptions and allowing for our randomisation being 12 months later, 500 participants allows for 80% power to test a non-inferiority margin of 10% at 2 years post-R1 (this equates to a hazard ratio of 1.38) with a one-sided 5% significance level, allowing for approximately 20% further drop-out after R1 amongst participants unwilling to cease treatment or being lost to follow-up.

A power of 80% will be attained when 238 PFS events have been observed. These calculations assume that PFS follow an exponential distribution and that there will be 3 years of recruitment to R1 and 2 further years of follow-up (in order that all participants can have PFS at 2 years post-R1 assessed).

Global Health Status/Quality of Life is considered a key secondary endpoint of R1 as the conclusion that the cessation of continuous treatment is non-inferior should not come at the expense of quality of life. Ignoring those required to account for participants lost to follow-up, the 406 participants recruited above give >95% power to detect a difference of 10 points at 2 years post-R1, where 10 points represents an accepted medium magnitude clinically relevant difference.[33] The calculation uses data collected from the intensive pathway of MRC Myeloma IX to estimate that the standard deviation in the Global Health Status/QoL subscale of EORTC-QLQ-C30 as 27.54 along with a two-sided 5% significance level.[34]

Randomisation 2

R2 is a multi-arm, multi-stage drug/drug-combination assessment platform with seamless phase II (activity) and phase III (efficacy) stages. Each experimental arm is compared with a designated control arm (R maintenance).

Early data from Myeloma XI and similar data from IFM/DFCI 2009 [35] suggest that a 10% upgrade in MRD-negative rate after 6 months continuous R treatment is achievable. It is thought that RBorD consolidation followed by attenuated continuous R or continuous R combined with antibody or immunotherapy could lead to a 30% increase in MRD-negative rate, a 20% upgrade of increase at 6 months post-randomisation when compared to continuous R treatment, as good as therapy combinations containing an alternative proteasome inhibitor.[33]

Following the strategy of Bratton and colleagues [36] for a multi-arm multi-stage trial with:

- N=2 stages (Stage 1 (activity) and Stage 2 (efficacy)) and

For peer review only - http://bmjopen.bmj.com/site/about/guidelines.xhtml

- J=4 experimental arms (RBorD+R, R+Isa, RBorIsaD + RIsa and TBC) and a single control arm (R)
- Where allocation is equal across each arms (1:1:1:1:1) and
- The power in Stage 1 and Stage 2 is 95% and 85%, respectively,
- the one-sided significance level in Stage 1 and Stage 2 is 25% and 1%, respectively,
- 10 participants per month are entered at R2, Stage 1 (activity) analysis and reporting requires 1 month and 5% of participants drop-out.

This equates to 40 participants in each arm at Stage I and 50 further participants in each arm at Stage 2. This will require a maximum of 46 months of recruitment plus a further 6 months of follow-up for the final participant. Overall, this design has a power of 83.4% and a one-sided family-wise error rate (FWER) of ~3%.

Randomisation 2 has been powered assuming four experimental arms and that all experimental arms will continue to stage 2. In the case that only three experimental arms are implemented or at least one experimental arm is concluded to show a lack of activity after stage 1, the trial will have a higher power than stated above.

Randomisation 3

Each arm of R3 will take the form of one-stage treatment design proposed by Simon, Wittes and Ellenberg [37] where the A'Hern design [38] is first implemented to determine treatment activity in both arms. Treatment activity will be determined by considering the PFS rate at 28 months. Data from Myeloma XI demonstrated a median PFS for high-risk participants in the intensive pathway of approximately 19 months (unpublished data). It is thought that for the treatments proposed to be investigated further in a Phase III trial they should increase the median PFS from 19 months to at least 28 months (9 month increase), i.e. increasing the PFS rate at 28 months from 34.7% to 50%.

In order to have an 80% power of demonstrating that the one-sided 95% confidence interval of the PFS rate at 28 months excludes 34.7%, 81 evaluable participants are required, where a treatment arm will be deemed a "success" if at least 37 out of 81 participants are progression-free at 28 months. To account for approximately 5% of participants being unevaluable at 28 months post-R3, 86 participants will be randomised to each treatment arm i.e. 172 participants are required to be entered into R3.

Total Study Sample Size

Accounting for those participants who will be lost-to-follow-up R1, R2 and R3 require 500, 450, and 172 participants to enter each randomisation respectively.

Considering R1 and R2, it is assumed that 50 participants will lose their MRD-negative status during the 12 months of continuous treatment (Myeloma XI, unpublished data). Therefore 550 participants are required to enter the MRD-negative part of the standard-risk pathway. It is assumed that 55% of standard-risk participants will be MRD-negative at the point of assessment therefore approximately 1000 participants are required to reach the MRD assessment stage of the standard-risk pathway [35, 39] It is assumed that 90% of all those recruited will reach this point therefore 1112 participants are required to enter the standard-risk pathway[5] . It is anticipated that 80% of all those recruited will be standard-risk (Myeloma XI, unpublished data) therefore at least 1390 participants are required to be recruited to the trial. Rounding this we obtain an overall sample size of 1400.

For peer review only - http://bmjopen.bmj.com/site/about/guidelines.xhtml

This gives 280 participants who are expected to enter the high-risk pathway, using the same logic as in the standard-risk pathway if 90% of participants reach R3 then there will be approximately 252 participants recruited to R3. Given the calculations above it is plausible that another arm could be added to the high-risk pathway at a later date.

Recruitment:

It is planned that 1400 participants will be recruited for a total of 36 months. Once all centres are open, the recruitment target is 38 participants a month (equating to approximately 30 standard risk and 8 high-risk participants).

In order to ensure the trial will meet the target sample size within the recruitment period, site set-up was prioritised whilst the trial was preparing to open to recruitment. In addition, the trial team are actively engaging with Principal Investigators at sites who support the trial to ensure that those sites open quickly and are maintaining regular communication with open sites to ensure that they continue to recruit to the trial.

The trial opened to recruitment on 11/05/2021.

Assignment of treatment allocations:

Each of the registration and randomisation procedures will be conducted centrally using the University of Leeds Clinical Trial Research Unit (CTRU) automated 24-hour web base and telephone system.

Registration

If a patient is suspected to have myeloma they will enter the trial at the time of routine diagnostic tests, through bone marrow registration. Once diagnosis is confirmed locally and the research team consider the patient potentially eligible for the trial, patients will be provided with full verbal explanation of the trial and full participant information sheet and informed consent documents to consider. Once the patient has provided informed consent to the first stage of the trial they will be registered into the trial at Bone Marrow Registration.

Patients who have already had Myeloma confirmed prior to being approached for the trial, and those who decline consent at bone marrow registration, will enter at main trial registration.

Data Collection

Data collection will primarily be via Remote Data Entry (RDE) on electronic case report forms (eCRFs), provided by the CTRU at the University of Leeds, and will be entered by staff at the research site. Access to the live RADAR database will be provided by the CTRU following sites being authorised to open to recruitment; guidance on RDE and completing eCRFs will be provided. Data regarding informed consent, QoL, Healthcare Resource Use questionnaires and in-patient stay forms will be collected via paper case report forms.

For peer review only - http://bmjopen.bmj.com/site/about/guidelines.xhtml

For peer review only

QoL and Healthcare Resource Use questionnaires will be completed by the participant in clinic or at home and sealed in an envelope before handing back to the site research team who will return them to the CTRU via post.

Data Management

All information collected during the course of the trial will be kept strictly confidential. Information will be held securely on paper and electronically at the CTRU. The CTRU will comply with all aspects of the Data Protection Act 2018.

Data will be monitored for quality and completeness by the CTRU. Missing data will be chased until it is received, confirmed as not available or the trial is at analysis. The CTRU/Sponsor will reserve the right to intermittently conduct source data verification exercises on a sample of participants, which will be carried out by staff from the CTRU/Sponsor. Source data verification will involve direct access to participant notes at the participating hospital sites and the ongoing central collection of copies of Informed Consent Documents and other relevant investigation reports.

Statistical methods

Statistical analysis is the responsibility of the CTRU statisticians, with the exception of the cost-effectiveness analysis, which will be undertaken by the health economists at the University of Leeds. A full statistical analysis plan (SAP) and Health Economics Analysis Plan (HEAP) will be written and approved before any analyses are undertaken.

The analysis population will depend on the randomisation. Separate intention to treat (ITT), per protocol (PP) and safety populations will be defined for each randomisation. In either case the ITT population will include participants according to their randomisation allocation, regardless of eligibility whether they prematurely discontinued the treatment, or did not comply with the regimen. The PP population will exclude individuals who do not receive their randomisation allocation or who are found to be ineligible following randomisation. The safety population will consist of all participants who receive at least one dose of the trial treatments and analyse participants according to what they received rather than what they were randomised to.

With the exception of two interim analyses, no other formal analysis is planned until participants have attained the primary endpoint for each of the randomisations.

Primary endpoint analyses

Randomisation 1

The primary endpoint for R1 will be attained 2 years after the final participant has been randomised to R1.

As R1 is a non-inferiority trial, both PP and ITT population analyses will be used as co-primary analyses and both are required to demonstrate statistical significance of non-inferiority where any discrepancies will be reported.

For peer review only - http://bmjopen.bmj.com/site/about/guidelines.xhtml

PFS at R1 will be assessed using Cox proportional hazards regression to adjust for the stratification factors of R1 excluding centre, and presented using Kaplan-Meier estimates of the survival function. The treatment cessation strategy will be declared non-inferior to the continuous treatment strategy if the upper bound of the one-sided 95% confidence interval for the hazard ratio is less than 1.38. Note no adjustment will be made for interim analysis due to it being a test for inferiority (see below) and not non-inferiority.

Randomisation 2

The primary endpoint for R2 will be attained 6 months after the final participant has been randomised to R2. Primary analysis will be undertaken on the ITT population.

An overall one-sided approximately 3% FWER will be applied accounting for each pair-wise comparison over each stage and all comparisons. This equates to a 25% and 1% one sided significance level at interim and final analysis. MRD-negative rate will be compared between each pair-wise comparison (experimental vs control) in turn using logistic regression and adjusted for the minimisation factors of R2 excluding centre.

Randomisation 3

The primary endpoint for R3 will be attained 28 months after the final participant has been randomised to R3.

Primary analysis will be undertaken on the ITT population. The number of participants who are alive and progression-free at 28 months post R3 will be summarised separately within each treatment arm and exact one sided 95% confidence intervals calculated. The null hypothesis is that the percentage of participants alive and progression-free at 28 months post R3 is 34.7%. A treatment arm is deemed a "success" if the one sided 95% confidence intervals of the PFS rate at 28 months excludes 34.71%. The treatment will then be proposed to be investigated further in a phase III trial.

No multiple imputation is planned for the primary endpoints.

Secondary endpoint analyses

Overall survival and other time-to-event endpoints will be analysed using methods similar to those described for progression-free survival. All binary endpoints will be analysed using similar methods to those described for the attainment of MRD negativity endpoint.

For both R1 and R3, each experimental arm will be independently compared to historical control data using molecularly matched individual participant data (IPD) from Myeloma XI+(TE) and assessed for superiority in terms of progression-free survival. Further details of the molecular matching of IPD will be described in the statistical analysis plan, but it is anticipated that propensity score matching may be an appropriate technique. In each case a one-sided 10% significance level will be applied for each comparison with historical data.

The number and proportion of participants in each response category will be summarised by allocated treatment and exact 95% two-sided confidence intervals will be calculated. The difference in

For peer review only - http://bmjopen.bmj.com/site/about/guidelines.xhtml

proportions for each response category will be presented with corresponding 95% two-sided confidence intervals.

Quality of life will be summarised using mean scores adjusted for baseline and two-sided 95% CI for each EORTC QLQ-C30 and EORTC QLQ-MY20 module symptom, role and functioning domain at each assessment time-point. Similar summaries will be produced for quality-adjusted life years (QALYs) as scored by the EQ-5D 3 Level (EQ-5D-3L) questionnaire. For the key secondary analysis of R1, the Global Health Status / Quality of Life subscale at two years post-R1, will be compared between the arms using a multivariable linear regression model, adjusting for the R1 stratification factors.

Exploratory and subgroup analyses

A series of subgroup and exploratory analyses will be undertaken. Subgroup analyses will follow the same structure as the main analysis of the respective endpoint. Where appropriate interaction terms will be added to the regression models to account for the subgroup being investigated. Subgroup and exploratory analyses may, by chance, generate false negative or positive results. Those carried out will be interpreted with caution.

The analysis for the exploratory outcomes will be detailed in the SAP.

Health economics

The economic evaluation will take a decision modelling approach to assess the cost-effectiveness of treatment options for risk stratified treatment vs standard non-stratified treatment over participants' lifetime. This will allow several research questions to be incorporated within one model. The model will be developed using best practice[40] and the model structure, health states and parameter values will be derived from the trial outputs, published literature, and expert clinical opinion. Data to model stratified treatment options will be drawn from the RADAR trial results while parameters for standard non-stratified treatment will be obtained from the wider literature.

Reflecting the main trial analysis of effectiveness, the economic evaluation will assess the cost-effectiveness of treatment options at each of the randomisations in RADAR. For standard risk participants at R1, the cost-effectiveness analysis will compare treatment with isatuximab for 12 cycles only to long-term isatuximab treatment, continued until disease progression. For standard risk participants who are MRD-positive at 100 days post-ASCT in terms of conversion to MRD negativity at R2, the cost-effectiveness of post-ASCT consolidation and maintenance treatments in the treatment of standard-risk participants will be evaluated. For high-risk participants at R3, the cost-effectiveness of post-ASCT consolidation and maintenance treatment strategies containing lenalidomide, bortezomib, dexamethasone and isatuximab separately will be evaluated. In addition, the cost-effectiveness of stratified treatment pathways will be compared with standard non-stratified treatments.

All analyses will be conducted following NICE reference case guidelines[41]. As such, outcomes will be measured in QALYs based on EQ-5D [42] and EORTC QLQ-C30 [43] utilities and costs will be calculated from a health and personal social services perspective. Cost-effectiveness will be determined in comparison to the NICE cost-effectiveness threshold of £20,000 per QALY gained. Deterministic and probabilistic (using Monte Carlo simulations) sensitivity analyses will be conducted to explore the

For peer review only - http://bmjopen.bmj.com/site/about/guidelines.xhtml

impact of parameter value changes on estimates of cost-effectiveness and to determine the level of uncertainty around the results of the lifetime analysis. In addition, value of information analysis will be conducted to estimate the value of further research.

Trial Oversight

The TMG includes the CI, CTRU team and co-investigators that are assigned responsibility for the clinical set up, ongoing management, promotion of the trial and interpretation of the results. The trial steering committee (TSC), consisting of independent clinicians and statisticians, will provide overall supervision on the trial, including trial progress, adherence to protocol, participant safety and consideration of new information.

Data Monitoring

An independent data monitoring and ethic committee (DMEC) will review the safety and ethics of the trial. Detailed reports will be prepared by the CTRU for the DMEC meetings which will take place at approximately yearly intervals. Additional reports will also be provided at intervals between these meetings. The committee will also review cumulative safety data by arm on an ongoing basis along with individual SAE/SAR listings. After each annual review, the DMEC will make their recommendations to the TSC about the continuation of the trial.

Interim Analyses

Full interim reports will be presented to the DMEC in confidence annually and following the planned interim analyses which are expected to take place when half the number of events (119) have been observed in the treatment de-escalation pathway (R1) and at the end of the activity stage 1 treatment escalation pathway (R2), approximately 6 months after the 40th participant has been randomised.

The interim analysis of R1 will be a one-sided test for inferiority conducted at the 5% significance level. PFS at R1 will be assessed using Cox proportional hazards regression to adjust for the minimisation factors (excluding centre), and presented using Kaplan-Meier estimates of the survival function.

The interim analysis for R2 will be conducted at the end of Stage I (the phase II part) of the randomisation which is due 6 months following the randomisation of the 40th participant. This analysis will compare each experimental arm with the control arm in separate tests of superiority using a one-sided 25% significance level and 95% power to determine whether any of the experimental arms show lack of activity i.e. the null hypothesis in the superiority test is not rejected. Should an arm show lack of activity it will not be continued to the second stage of the randomisation (the phase III part). To account for the interim analysis and to give an overall significance level of 1% and power of 80% for each individual comparison the final analysis will be conducted using a one-sided 1% significance level and 85% power. Recruitment will continue seamlessly between activity and efficacy in these scenarios. Formal powered pair-wise comparisons are not undertaken comparing experimental arms at either stage.

The DMEC committee members, in light of the interim analysis, will make their recommendations to the TSC about the continuation of the different aspects of the trial.

For peer review only - http://bmjopen.bmj.com/site/about/guidelines.xhtml

For peer review only

Harms

Adverse events

Adverse events are any untoward medical occurrence in a participant or clinical trial subject administered a medicinal product and which does not necessarily have a causal relationship with this treatment. AEs can be defined as any unintentional, unfavourable clinical signs or symptoms; any new illness or disease, or the deterioration of existing disease or illness; or clinically relevant deterioration in any laboratory assessments or clinical test. Due to the nature of multiple myeloma and its treatment, participants are likely to experience several adverse events throughout the course of the disease.

All AEs, both related and unrelated to myeloma treatment will be collected on the relevant eCRF from trial registration until 60 days after the last dose of protocol treatment and will be evaluated in accordance with the National Cancer Institute Common Terminology Criteria for Adverse Events 5.0 (NCI-CTCAE). AEs and ARs should continue to be collected for participants who are randomised to 'stop isatuximab' at R1 (and until 60 days after last on-trial assessment), even though they will not be receiving any trial drugs during this period.

Adverse Events of special interest

An AE of special interest (serious or non-serious) is one of scientific and medical concern specific to isatuximab, for which ongoing monitoring and rapid communication by the investigator to the sponsor is considered to be appropriate. Isatuximab AEs of special interest include; ≥Grade 3 infusion reactions (IRs), Pregnancies, Symptomatic overdoses with IMP, and Second Primary Malignancies (SPMs).

Serious adverse events

Serious Adverse Events (SAEs) are defined as any untoward medical occurrence or effect that at any dose results in death; or are life threatening (at the time of the event); or require in-patient hospitalisation or prolongation of existing hospitalisation; or result in persistent or significant disability or incapacity; or result in a congenital anomaly or birth defect; or any other important medical event. All SAEs will be reported from registration until 60 days post the last dose of the trial drug. SAEs should continue to be collected for participants who are randomised to 'stop isatuximab' at R1 (and until 60 days after last on-study assessment), even though they will not be receiving any study drugs during this period.

Serious Adverse Reactions (SARs) are SAEs that are deemed possibly related to any dose administered of any trial treatment. Suspected unexpected serious adverse events (SUSARs) are SARs which are not listed in the reference safety information for that medicinal product. SARs and SUSARs will be reported from the date of first trial dose and for the duration of the trial.

Secondary malignancies

All new primary malignancies (SPMs) or suspected malignancies occurring from the date of trial registration and for the duration of the trial will be recorded. SPMs will be summarised and reviewed by an appointed member of the TMG who will determine whether trial treatment should continue.

For peer review only - http://bmjopen.bmj.com/site/about/guidelines.xhtml

Pregnancies

Participants registered into RADAR agree to follow the Celgene approved Pregnancy Prevention Programme. Pregnancies and suspected pregnancies (including a positive pregnancy test regardless of age or disease status) in a female participant must be reported throughout the study and for 12 months after the last dose of cyclophosphamide or for 5 months following cessation of protocol treatment, whichever is longest. Pregnancies and suspected pregnancies (including a positive pregnancy test regardless of age or disease status) in a male participant's partner must be reported throughout the study and for 6 months after the last dose of cyclophosphamide or for 5 months following cessation of protocol treatment, whichever is longest.

Safety Analyses

Safety analyses will summarise all SUSARs, SARs, SAEs, AEs and ARs, respective to where they occurred within the trial pathway (Induction, ASCT, Pre-R1, Post-R1, Post-R2, Post-R3). Safety data will be presented by treatment group for the safety population in addition to relationship to trial treatment.

Auditing

The CTRU and the trial sponsor have procedures in place to ensure that serious breaches of Good Clinical Practice (GCP) or the trial protocol are picked up and reported. A triggered monitoring plan will ensure that sites at risk are monitored accordingly.

Patient and Public Involvement

RADAR has been developed following extensive discussion with the UK myeloma community. Patients were first involved into the research at the grant application stage, and were incorporated into the development of the primary endpoint of R1, in which they confirmed a research question involving the stopping of treatment given there was no detectable myeloma would be of interest. The trial consent and participant information documents were reviewed for clarity by a patient representative. To ensure that a patient perspective is considered throughout the trial a PPI advisory group, including 4-8 members will feed into TMG meetings, and plan to host quarterly meetings to discuss urgent matters. It is envisioned that the PPI group, may help with writing summaries of results that can be published on patient forums and websites and maybe even shared directly with patients.

Ethics and Dissemination

Research and ethics approval

Ethics approval has been obtained from the London – Central Research Ethics Committee (20/LO/0238). In addition capacity and capability has been confirmed by the appropriate research and development department for each participating centre prior to opening to participant recruitment into the trial. This included but was not limited to; having access to the non-trial supplied IMP (Bor, Cy and D), being able to conduct FISH as SoC, capacity of staff, ability to complete all protocol required assessments/investigations, and ability to archive trials documents.

Participants will be required to provide informed consent before joining the trial.

For peer review only - http://bmjopen.bmj.com/site/about/guidelines.xhtml

Protocol Amendments

The trial opened to recruitment on 11/05/2021 using protocol version 2, dated 29th April 2020. This publication is written based from protocol version 3 dated July 2021. An amendment to protocol version 3 is anticipated Mid-2022, which should include the following:

High-Risk Pathway

The cytogenetic abnormality del(1p) will be added to the definition of high-risk. In addition, Isa will be added to induction regimen for high-risk patients following their first cycle of RCyBorD (whilst their risk status is confirmed). Furthermore, R3 will be removed and high-risk patients will receive RBorIsaD + RIsa only post-ASCT. The pathway will follow a single-arm, single stage, phase II, three outcome design, with the new primary objective to assess the activity of RCyBorD+Isa induction followed by ASCT and RBorD+Isa consolidation and RIsa maintenance in terms of PFS at 18 months post trial registration.

Other changes

- The removal of the phone registration and randomisation;
- The clarification of allowing prescribed medication to remove risks of thromboembolism before preceding onto treatment in the exclusion criteria for registration (and for starting post ASCT treatment)
- The allowance of blood support products, blood transfusions and platelet transfusions as per institutional guidelines at eligibility and pre-treatment assessments
- An update of exclusion criteria to allow for previous plasmacytomas.

Consent or assent

The PI retains overall responsibility for the informed consent of participants at their site and must ensure that any medically qualified person delegated responsibility to participate in the informed consent process is duly authorised, trained and competent to participate according to the ethically approved protocol, principles of GCP and Declaration of Helsinki. Written consent will be obtained and signed by a medically qualified member of the site research team. Informed consent must be obtained, and the participant must be registered into the trial prior to the participant undergoing procedures that are specifically for the purposes of the trial and are out-with standard routine care at the participating site. This include the confirmation of optional consent (QoL/ Healthcare resource use questionnaires and decision regarding consent for samples to be used in future research). At any stage, participants can withdraw consent without repercussion.

Confidentiality

All information collected during the course of the trial will be kept strictly confidential. Information will be held securely on paper and electronically at the Clinical Trials Research Unit (CTRU). The CTRU will comply with all aspects of the Data Protection Act 2018.

For peer review only - http://bmjopen.bmj.com/site/about/guidelines.xhtml

Access to data

Individual participant data (with any relevant supporting material, e.g. data dictionary, protocol, statistical analysis plan) for all trial participants (excluding any trial-specific participant opt-outs) will be made available for secondary research purposes at the end of the trial, i.e. usually when all primary and secondary endpoints have been met and all key analyses are complete.

Data will be shared according to a controlled access approach, based on the following principles:

- The value of the proposal will be considered in terms of the strategic priorities of the CTRU, Chief Investigator and Sponsor, the scientific value of the proposed project, and the resources necessary and available to satisfy any data release request.
- We encourage a collaborative approach to data sharing, and believe it is best practice for researchers who generated datasets to be involved in subsequent uses of those datasets.
- The timing and nature of any data release must not adversely interfere with the integrity of the trial or research project objectives, including any associated secondary and exploratory research objectives detailed in the ethically approved original research protocol. On an individual trial or research project basis, a reasonable period of exclusivity will be agreed with the trial or research project team.
- Any data release must be lawful, in line with participants' rights and must not compromise patient confidentiality. Where the purposes of the project can be achieved by using anonymised or aggregate data this will always be used. We will release individual patient data only in a form adjusted so that recipients of the data cannot identify individual participants by any reasonably likely means. We will also only share data when there is a binding agreement in place stating that data recipients will not attempt to re-identify any individual participants.
- Any data release must be in line with any contractual obligations to which the CTRU is subject.
- The research must be carried out by a bone fide researcher with the necessary skills and resources to conduct the research project.
- The research project must have clear objectives and use appropriate research methods.
- The research must be carried out on behalf of a reputable organisation that can demonstrate appropriate IT security standards to ensure the data is protected and to minimise the risk of unauthorised disclosure.

Data will only be shared for participants who have given consent to use of their data for secondary research.

Requests to access trial data should be made to CTRU-DataAccess@leeds.ac.uk in the first instance. Requests will be reviewed (based on the above principles) by relevant stakeholders. No data will be released before an appropriate agreement is in place setting out the conditions of release. The agreement will govern data retention requirements, which will usually stipulate that data recipients must delete their copy of the data at the end of the planned project.

Ancillary and post-trial agreements

Participants who stop trial treatment due to progression or any point prior to the end of trial will be treated off-trial at the discretion of their treating clinician. Those who stop prior to disease progression will be followed up 2 monthly until disease progression, death or the end of the trial. Following disease progression all participants will be followed up annually until death, or until the end of the trial for post progression endpoints.

Participants in the R1 pathway who are MRD-positive at 12 months post 12 cycles of isatuximab and therefore aren't eligible for randomisation 1 will continue to receive isatuximab maintenance, these

For peer review only - http://bmjopen.bmj.com/site/about/guidelines.xhtml

participants would continue to be monitored, and data collected, as previously but trial samples would not be collected.

Dissemination policy

Authorship will be in keeping with the UK-MRA publication policy and due acknowledgement to participants, local investigators, funders and NCRI Haematological Oncology Study Group support made. The success of the trial depends upon the collaboration of all participants. For this reason, credit for the main results will be given to all those who have collaborated in the trial, through authorship and contributor-ship. Uniform requirements for authorship for manuscripts submitted to medical journals will guide authorship decisions alongside the guidance of the UK Myeloma Research Alliance Authorship Policy.

To maintain the scientific integrity of the trial, data will not be released prior to the end of the trial or a primary endpoint being reached, either for trial publication or oral presentation purposes, without the permission of the Trial Steering Committee and the (Co-)Chief Investigators. In addition, individual collaborators must not publish data concerning their participants that is directly relevant to the questions posed in the trial until the main results of the trial have been published and following written consent from the Sponsor.

For peer review only

Appendices

Informed consent material

The consent forms which are to be completed by the participant at Bone Marrow Registration and/or Trial Registration and prior to each randomisation pathways are included in supplementary material.

Biological specimens

The collection of central samples for laboratory analysis is summarised in Figures 3 and 4. The analysis to be conducted for trial purposes is stated in the supplementary material. Additional analysis may be carried out by each central laboratory provided the appropriate consent for sample use in future research has been provided by the participant at trial entry.

Author Contributions

The RADAR trial was conceived by KY and MC and designed by them in collaboration with DAC. All authors (KLR, ABC, KR, DAC, AH, SQ, MD, MK, RO, HWA, GC, DM, CO, LB, RL, AP, BD, MC, GP, RP, GJ, CB, JS, RT, AC, CP, MC, SA, and KY) inputted into the development of the protocol and participant information sheet. The first draft of the protocol paper was written by KLR and ABC. All authors (KLR, ABC, KR, DAC, AH, SQ, MD, MK, RO, HWA, GC, DM, CO, LB, RL, AP, BD, MC, GP, RP, GJ, CB, JS, RT, AC, CP, MC, SA, and KY) reviewed and approved the final manuscript.

Funding

This trial is funded by Cancer Research UK (C9203/A24078), Sanofi Genzyme and Celgene: A BMS Company. The funders had no role in the design, collection, analysis or collection of data; in writing

For peer review only - http://bmjopen.bmj.com/site/about/guidelines.xhtml

the manuscript; or in the decision to submit the manuscript for publication. This work was also supported by Core Clinical Trials Unit Infrastructure from Cancer Research UK (C7852/A25447).

The trial sponsor is responsible for the overall conduct of the trial as defined by Directive 2001/20/EC is University of Leeds, UoL/LTHT Joint Sponsor QA office (CTIMPs); Research & Innovation Centre/Faculty of Medicine & Health; Leeds Teaching Hospitals NHS Trust/University of Leeds; St James University Hospital; Leeds LS9 7TF.

Competing Interest Statement

ABC, KLR, DAC, AH, CO, LBai, LBar, AP, RL report grants and non-financial support from BMS/Celgene, grants and non-financial support from Merck Sharpe & Dohme, grants and non-financial support from Amgen, grants and non-financial support from Takeda, during the conduct of the trial. DAC also reports travel support from Celgene Corporation.

KY, RDT, CB, GJ, GP, MC, BD, DM, GC, HA, KR,SA, AC have no conflicting interests to declare. MC declares, Bristol Myers Squibb- employee, Honoraria/travel support in the last 3 years from, Amgen ,BMS/Celgene, Janssen, Takeda, Abbvie. CP declares BMS/Celgene Ad boards and speaker fees, Sanotif – Ad board, speaker fees, conference registration fees. JS reports Carrying out consultancy work (Advisory Board) for Sanofi. And an educational speaking engagement for Celgene/BMS RP declares; Honoraria – Jannsen, BMS, Abbvie, GSK. Consultancy: GSK, Janssen. Meeting support: Janssen, Takeda, BMS. RO declares- Janssen - advisory board, honoraria, Celegene – honoraria, Beigene - advisory board, honoraria, Astra Zeneca – honoraria. MK declares inter-relationships: AbbVie: consultancy; Amgen: honoraria; BMS/Celgene: consultancy, research funding (institution); GSK: consultancy; Janssen: consultancy, research funding (institution); Karyopharm: consultancy; Pfizer: consultancy; SeattleGenetics: consultancy; Takeda: consultancy; Sanofi: honoraria. MD reports owning stock in Abingdon Health. SQ is the founder and CSO of Achilles therapeutics a company developing T cell therapies for solid tumours.

For peer review only

References

1. Cancer Research UK. Myeloma Incidence Statistics 2015 [Available from: http://www.cancerresearchuk.org/health-professional/cancer-statistics/statistics-by-cancer-type/myeloma/incidence accessed 21st October 2018 2018.
2. Kristinsson SY, Anderson WF, Landgren O. Improved long-term survival in multiple myeloma up to the age of 80 years. *Leukemia* 2014;28(6):1346-8. doi: 10.1038/leu.2014.23
3. Child JA, Morgan GJ, Davies FE, et al. High-dose chemotherapy with hematopoietic stem-cell rescue for multiple myeloma. *N Engl J Med* 2003;348(19):1875-83. doi: 10.1056/NEJMoa022340
4. Attal M, Harousseau JL, Stoppa AM, et al. A prospective, randomized trial of autologous bone marrow transplantation and chemotherapy in multiple myeloma. Intergroupe Francais du Myelome. *N Engl J Med* 1996;335(2):91-7. doi: 10.1056/NEJM199607113350204
5. Attal M, Lauwers-Cances V, Hulin C, et al. Lenalidomide, Bortezomib, and Dexamethasone with Transplantation for Myeloma. *N Engl J Med* 2017;376(14):1311-20. doi: 10.1056/NEJMoa1611750
6. Weber DM, Chen C, Niesvizky R, et al. Lenalidomide plus dexamethasone for relapsed multiple myeloma in North America. *N Engl J Med* 2007;357(21):2133-42. doi: 10.1056/NEJMoa070596
7. McCarthy PL, Holstein SA, Petrucci MT, et al. Lenalidomide Maintenance After Autologous Stem-Cell Transplantation in Newly Diagnosed Multiple Myeloma: A Meta-Analysis. *Journal of clinical oncology : official journal of the American Society of Clinical Oncology* 2017;35(29):3279-89. doi: 10.1200/JCO.2017.72.6679
8. Delforge M, Minuk L, Eisenmann JC, et al. Health-related quality-of-life in patients with newly diagnosed multiple myeloma in the FIRST trial: lenalidomide plus low-dose dexamethasone versus melphalan, prednisone, thalidomide. *Haematologica* 2015;100(6):826-33. doi: 10.3324/haematol.2014.120121
9. Kumar S, Flinn I, Richardson PG, et al. Randomized, multicenter, phase 2 study (EVOLUTION) of combinations of bortezomib, dexamethasone, cyclophosphamide, and lenalidomide in previously untreated multiple myeloma. *Blood* 2012;119(19):4375-82. doi: 10.1182/blood-2011-11-395749
10. Sonneveld P, Avet-Loiseau H, Lonial S, et al. Treatment of multiple myeloma with high-risk cytogenetics: a consensus of the International Myeloma Working Group. *Blood* 2016;127(24):2955-62. doi: 10.1182/blood-2016-01-631200
11. Joseph NS, Gentili S, Kaufman JL, et al. High-risk Multiple Myeloma: Definition and Management. *Clin Lymphoma Myeloma Leuk* 2017;17S:S80-S87. doi: 10.1016/j.clml.2017.02.018
12. Chng WJ, Dispenzieri A, Chim CS, et al. IMWG consensus on risk stratification in multiple myeloma. *Leukemia* 2014;28(2):269-77. doi: 10.1038/leu.2013.247
13. Shah V, Sherborne AL, Walker BA, et al. Prediction of outcome in newly diagnosed myeloma: a meta-analysis of the molecular profiles of 1905 trial patients. *Leukemia* 2018;32(1):102-10. doi: 10.1038/leu.2017.179
14. Boyd KD, Ross FM, Chiecchio L, et al. A novel prognostic model in myeloma based on co-segregating adverse FISH lesions and the ISS: analysis of patients treated in the MRC Myeloma IX trial. *Leukemia* 2012;26(2):349-55. doi: 10.1038/leu.2011.204
15. Kuiper R, Broyl A, de Knegt Y, et al. A gene expression signature for high-risk multiple myeloma. *Leukemia* 2012;26(11):2406-13. doi: 10.1038/leu.2012.127
16. Palumbo A, Avet-Loiseau H, Oliva S, et al. Revised International Staging System for Multiple Myeloma: A Report From International Myeloma Working Group. *Journal of clinical oncology : official journal of the American Society of Clinical Oncology* 2015;33(26):2863-9. doi: 10.1200/JCO.2015.61.2267
17. Paiva B, Vidriales MB, Cervero J, et al. Multiparameter flow cytometric remission is the most relevant prognostic factor for multiple myeloma patients who undergo autologous stem cell transplantation. *Blood* 2008;112(10):4017-23. doi: 10.1182/blood-2008-05-159624

For peer review only - http://bmjopen.bmj.com/site/about/guidelines.xhtml

18. Rawstron AC, Child JA, de Tute RM, et al. Minimal residual disease assessed by multiparameter flow cytometry in multiple myeloma: impact on outcome in the Medical Research Council Myeloma IX Study. *Journal of clinical oncology : official journal of the American Society of Clinical Oncology* 2013;31(20):2540-7. doi: 10.1200/JCO.2012.46.2119
19. Paiva B, van Dongen JJM, Orfao A. New criteria for response assessment: role of minimal residual disease in multiple myeloma. *Blood* 2015;125(20):3059-68. doi: 10.1182/blood-2014-11-568907
20. Sidiqi MH, Aljama MA, Bin Riaz I, et al. Bortezomib, lenalidomide, and dexamethasone (VRd) followed by autologous stem cell transplant for multiple myeloma. *Blood Cancer Journal* 2018;8(11):106. doi: 10.1038/s41408-018-0147-7
21. Facon T, Kumar S, Plesner T, et al. Daratumumab plus Lenalidomide and Dexamethasone for Untreated Myeloma. *The New England journal of medicine* 2019;380(22):2104-15. doi: 10.1056/NEJMoa1817249
22. Attal M, Richardson PG, Rajkumar SV, et al. Isatuximab plus pomalidomide and low-dose dexamethasone versus pomalidomide and low-dose dexamethasone in patients with relapsed and refractory multiple myeloma (ICARIA-MM): a randomised, multicentre, open-label, phase 3 study. *The Lancet (British edition)* 2019;394(10214):2096-107. doi: 10.1016/S0140-6736(19)32556-5
23. Deckert J, Wetzel MC, Bartle LM, et al. SAR650984, a novel humanized CD38-targeting antibody, demonstrates potent antitumor activity in models of multiple myeloma and other CD38+ hematologic malignancies. *Clin Cancer Res* 2014;20(17):4574-83. doi: 10.1158/1078-0432.CCR-14-0695
24. Kumar S, Paiva B, Anderson KC, et al. International Myeloma Working Group consensus criteria for response and minimal residual disease assessment in multiple myeloma. *The Lancet Oncology* 2016;17(8):e328-46. doi: 10.1016/S1470-2045(16)30206-6
25. Lahuerta JJ, Paiva B, Vidriales MB, et al. Depth of Response in Multiple Myeloma: A Pooled Analysis of Three PETHEMA/GEM Clinical Trials. *Journal of clinical oncology : official journal of the American Society of Clinical Oncology* 2017;35(25):2900-10. doi: 10.1200/JCO.2016.69.2517
26. Munshi NC, Avet-Loiseau H, Rawstron AC, et al. Association of Minimal Residual Disease With Superior Survival Outcomes in Patients With Multiple Myeloma: A Meta-analysis. *JAMA Oncol* 2017;3(1):28-35. doi: 10.1001/jamaoncol.2016.3160
27. Avet-Loiseau H, Bahlis NJ, Chng WJ, et al. Ixazomib significantly prolongs progression-free survival in high-risk relapsed/refractory myeloma patients. *Blood* 2017;130(24):2610-18. doi: 10.1182/blood-2017-06-791228
28. Chan A-W, Tetzlaff JM, Gøtzsche PC, et al. SPIRIT 2013 explanation and elaboration: guidance for protocols of clinical trials. *BMJ (Online)* 2013;346(jan08 15):e7586-e86. doi: 10.1136/bmj.e7586
29. Blade J, Samson D, Reece D, et al. Criteria for evaluating disease response and progression in patients with multiple myeloma treated by high-dose therapy and haemopoietic stem cell transplantation. Myeloma Subcommittee of the EBMT. European Group for Blood and Marrow Transplant. *Br J Haematol* 1998;102(5):1115-23.
30. Durie BG, Harousseau JL, Miguel JS, et al. International uniform response criteria for multiple myeloma. *Leukemia* 2006;20(9):1467-73. doi: 10.1038/sj.leu.2404284
31. Rajkumar SV, Harousseau JL, Durie B, et al. Consensus recommendations for the uniform reporting of clinical trials: report of the International Myeloma Workshop Consensus Panel 1. *Blood* 2011;117(18):4691-5. doi: 10.1182/blood-2010-10-299487
32. Jackson GH, Davies FE, Pawlyn C, et al. Lenalidomide Is a Highly Effective Maintenance Therapy in Myeloma Patients of All Ages; Results of the Phase III Myeloma XI Study. *Blood* 2016;128(22):1143-43.
33. Cocks K, King MT, Velikova G, et al. Evidence-based guidelines for determination of sample size and interpretation of the European Organisation for the Research and Treatment of Cancer

For peer review only - http://bmjopen.bmj.com/site/about/guidelines.xhtml

Quality of Life Questionnaire Core 30. *Journal of clinical oncology : official journal of the American Society of Clinical Oncology* 2011;29(1):89-96. doi: 10.1200/JCO.2010.28.0107
34. Royle KL, Gregory WM, Cairns DA, et al. Quality of life during and following sequential treatment of previously untreated patients with multiple myeloma: findings of the Medical Research Council Myeloma IX randomised study. *Br J Haematol* 2018;182(6):816-29. doi: 10.1111/bjh.15459
35. Avet-Loiseau H, Corre J, Lauwers-Cances V, et al. Evaluation of Minimal Residual Disease (MRD) By Next Generation Sequencing (NGS) Is Highly Predictive of Progression Free Survival in the IFM/DFCI 2009 Trial. American Society of Haematology (57th Annual Meeting and Exposition). Orlando, FL, 2015.
36. Bratton DJ, Phillips PP, Parmar MK. A multi-arm multi-stage clinical trial design for binary outcomes with application to tuberculosis. *BMC Med Res Methodol* 2013;13:139. doi: 10.1186/1471-2288-13-139
37. Simon R, Wittes RE, Ellenberg SS. Randomized phase II clinical trials. *Cancer Treat Rep* 1985;69(12):1375-81.
38. A'Hern RP. Sample size tables for exact single-stage phase II designs. *Stat Med* 2001;20(6):859-66. doi: 10.1002/sim.721
39. Reeder CB, Reece DE, Kukreti V, et al. Once- versus twice-weekly bortezomib induction therapy with CyBorD in newly diagnosed multiple myeloma. *Blood* 2010;115(16):3416-7. doi: 10.1182/blood-2010-02-271676
40. Caro JJ, Briggs AH, Siebert U, et al. Modeling good research practices--overview: a report of the ISPOR-SMDM Modeling Good Research Practices Task Force-1. *Med Decis Making* 2012;32(5):667-77. doi: 10.1177/0272989X12454577
41. Guide to the methods of technology appraisal 2013. London: NICE 2013.
42. Dolan P, Roberts J. Modelling valuations for Eq-5d health states: an alternative model using differences in valuations. *Med Care* 2002;40(5):442-6.
43. Rowen D, Brazier J, Young T, et al. Deriving a preference-based measure for cancer using the EORTC QLQ-C30. *Value Health* 2011;14(5):721-31. doi: 10.1016/j.jval.2011.01.004

For peer review only

For peer review only - http://bmjopen.bmj.com/site/about/guidelines.xhtml

Figure Legends/ Captions

Figure 1: Flow chart of participant's timelines in the RADAR study

Figure 2: Schedule of local investigations

Figure 3: Central Investigations from diagnosis up to 100 days post-ASCT

Figure 4: Central Investigations for the R1, R2 and R3 pathways

For peer review only

For peer review only - http://bmjopen.bmj.com/site/about/guidelines.xhtml

Figure 1: Flowchart of Participants timelines in the RADAR Study

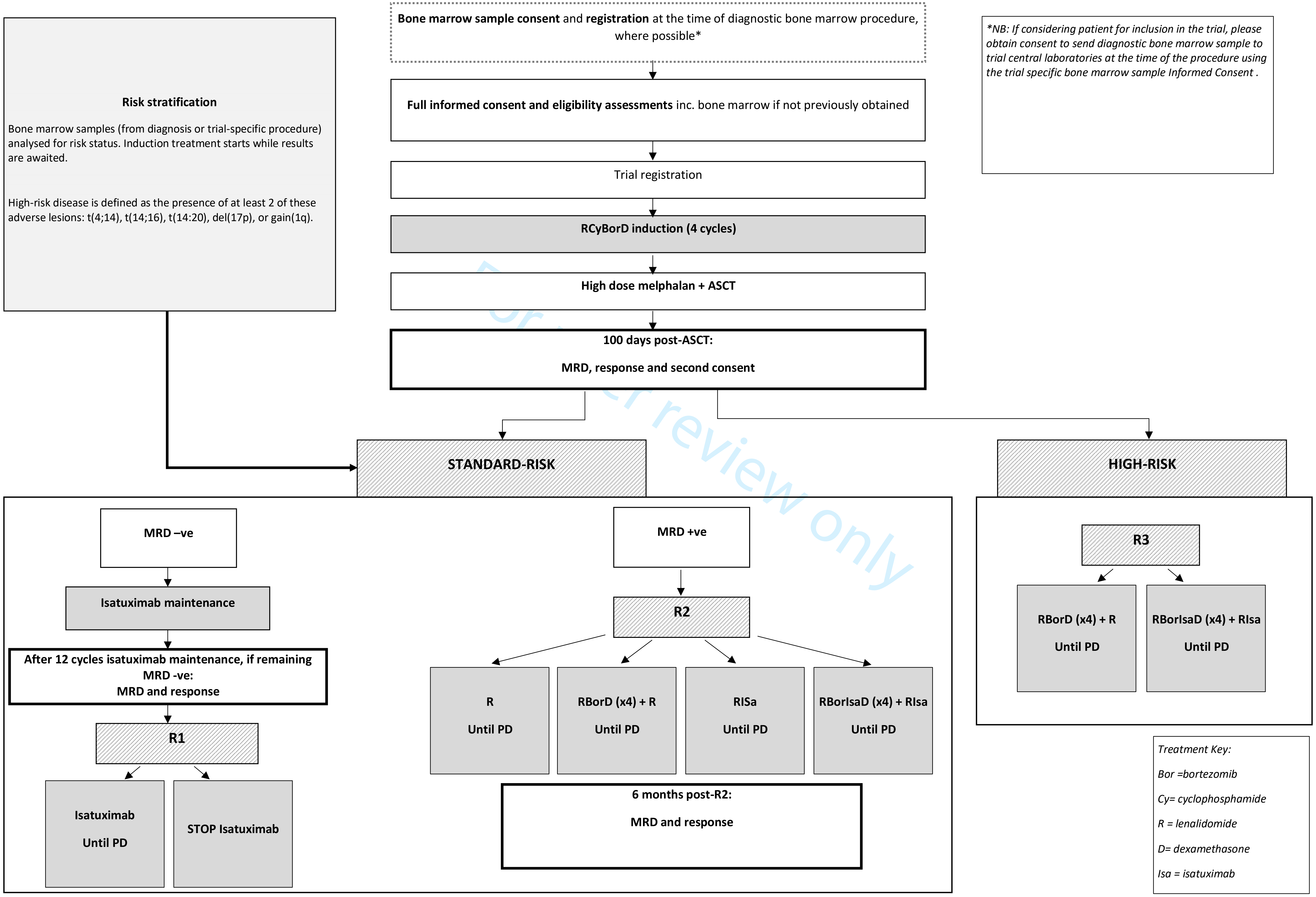

For peer review only

Figure 2: Schedule of Local Investigations

	RCyBorD Induction – All participants				R1 treatment pathway			R2 and R3 treatment pathway R2 participants on R or RIsa arm: After 3,6,12 and 18 cycles**		
	**Pre-registration/baseline** (within 14 days prior to registration, unless otherwise stated)	**Day 1 (or day -1) of each cycle during RCyBorD induction††**	**End of RCyBorD induction**	**100 days post-ASCT** (within 14 days prior to R2, start of Isatuximab maintenance or R3)	**Day 1 of each cycle of isatuximab treatment††**	**On the day of isatuximab dose††**	**Follow up for participants coming off study during R1 treatment pathway: every 2 months**	**Day 1 of each cycle of treatment††**	**On day of (and prior to) every isatuximab dose††**	**Follow up for participants coming off study during R2/R3 treatment pathway: every 2 months**
Written informed consent	✓ Consent to be taken prior to all assessments, permitted outside the 14-day window			✓ (permitted outside the 14-day window)						
Physical examination	✓	✓	✓	✓	✓			✓		
ECOG	✓	✓	✓	✓	✓			✓		
Covid vaccination status	✓	✓	✓	✓	✓		✓	✓		✓
Medical history (including review for prior cancers and history of Covid infection)	✓		✓	✓						
Assessment of cardiac risk*	✓(results from formal measurements within 28 days prior to registration)	If clinically indicated	✓	If clinically indicated	If clinically indicated			If clinically indicated		
ECG					Every 3 months in participants receiving Isatuximab			Every 3 months in participants receiving Isatuximab		
Thyroid function monitoring*	✓(within 28 days prior to registration)	If clinically indicated	✓	If clinically indicated	If clinically indicated			If clinically indicated		
FBC, LFTs, albumin, LDH, U&Es, calcium, and creatinine¶	✓	✓	✓	✓	✓	✓ FBC, U&E and creatinine only		✓	✓ FBC, U&E and creatinine only	
Hepatitis B, (surface antigen and core antibody), ,hepatitis C and HIV	⍰(within 28 days prior to registration)									
β2-microglobulin (ß2M)	✓									
C-Reactive protein (CRP)	✓									
Calculated creatinine clearance (using Cockcroft-Gault formula)	✓	✓	✓	✓	✓			✓		
Serum paraprotein and immunofixation, serum free light chains and urinary free light chain detection	✓	✓	✓	✓	✓		✓ (if performed as part of standard care)	✓		✓ (If performed as standard of care)
Bone marrow (aspirate and trephine) *See Table below for details of central investigations*	✓ (within 6 weeks prior to registration)	✓(recommended only if a first occurrence of CR or sCR is suspected)	✓	✓	✓(recommended only if a first occurrence of CR or sCR is suspected)		✓ (if performed as part of standard care)	✓ (recommended only if first occurrence of CR/sCR is suspected)		✓ (If performed as standard of care)
Pregnancy prevention counselling	✓	At least every 28 days during lenalidomide treatment								
Pregnancy test† (for females of childbearing potential)	✓	✓¥			✓			✓¥		
Cross-sectional imaging according to local practice, to include the thorax (low dose whole body CT recommended)	Within 3 months before registration§	If clinically indicated. ‡			If clinically indicated			If clinically indicated		
Assessment of adverse events (including adverse events and second primary malignancy)		Monitor throughout study and report on relevant treatment eCRFs (from first treatment dose until 60 days post last treatment dose)			Monitor throughout study and report on relevant treatment eCRFs (from first treatment dose until 60 days post last treatment dose) . AEs should continue to be monitored for participants randomised to 'stop Isatuximab' at R1 (and until 60 days after last on-study assessment).			Monitor throughout study and report on relevant treatment eCRFs (from first treatment dose until 60 days post last treatment dose)		

**Performed as part of standard of care.*
*** ECGs should be scheduled to align with other required face-to-face visits*
¶ The FBC should be repeated mid-cycle 1 (Day 14 +/-3 days) or more frequently and during subsequent cycles if there is a concern about cytopenias
† Females of childbearing potential (FCBP) must have a negative pregnancy test performed by a healthcare professional in accordance with the Celgene Lenalidomide Pregnancy Prevention Plan
†† May be performed within the 48 hours prior to the specified day
*** In addition to standard Day 1 assessments*
¥ All FCBP: The first pregnancy test must be performed within 10 to 14 days prior to the start of lenalidomide and the second pregnancy test must be performed within 24 hours prior to the start of lenalidomide.
FCBP with regular or no menstrual cycles: within 24 hours before start of lenalidomide treatment; weekly for the first 28 days of study participation; 4-weekly as a minimum during lenalidomide treatment; at study discontinuation; and 4 weeks after last dose of lenalidomide
FCBP with irregular menstrual cycles: within 24 hours before start of lenalidomide treatment; weekly for the first 28 days of study participation; 2-weekly as a minimum during lenalidomide treatment; at study discontinuation; and at 2 and 4 weeks after last dose of lenalidomide

N.B. During the COVID-19 pandemic, face-to-face assessments must be carried out at least at the beginning of cycles 1 and 2 and at the end of consolidation. Subsequently, participants must be seen by a clinician at least every 3 cycles (and more frequently if clinically indicated or there are concerns).

Figure 3: Central Investigations from diagnosis up to 100 days post-ASCT

Sample	Investigation	Diagnosis<br><br>(if patient consented on NHS form and trial bone marrow Informed Consent Document)[1]		Baseline (post trial consent and before the start of treatment)	Day 1 of Cycle 1 RCyBorD induction	End of induction cycle 1	End of induction cycle 2	End of RCyBorD induction	100 days post-ASCT and prior to consolidation/ maintenance
0.5-1 mL EDTA bone marrow aspirate<br><br>**SEND TO HMDS, LEEDS**	Minimal Residual Disease	✓	**Trial consent**<br><br>**Consented to Myeloma XV trial and central laboratory investigations**	✓<br><br>(if not sent previously)				✓	✓
10 mL clotted peripheral blood<br><br>Random urine sample<br><br>**SEND TO BIRMINGHAM**	Disease parameters (including paraproteins) for response assessments and covid-19 antibody response<br><br>M-protein intereference assay			✓	✓<br><br>**Only if baseline sample not within 14 days**	✓	✓	✓	✓
10 mL EDTA bone marrow aspirate<br><br>10 mL blood<br><br>**SEND TO UCL, LONDON**	Tumour DNA for neoantigens<br><br>cfDNA<br><br>Immune profiling	✓<br><br>Bone marrow only		✓<br><br>BMA (if not sent previously) & blood	✓<br><br>**Cycle 1 only**<br><br>10 mL blood (**only if baseline sample is not from within 14 days prior to cycle 1 day 1**)				✓
Bone marrow trephine (6 sections)<br><br>**SEND TO UCL, LONDON**	Immunostaining for immune function	✓		✓<br><br>(if not sent previously					✓

[1] Baseline bone marrow samples may be taken before trial consent, provided that patients have consented to these samples being taken using the standard NHS consent form. The patient must have provided written informed trial consent on the trial bone marrow Informed Consent Document before these samples can be sent to the central laboratories.

For peer review only

Figure 4: Central Investigations for the R1, R2 and R3 pathways

Sample	Investigation	Standard-risk MRD-negative participants (R1 pathway)	Standard-risk MRD-positive participants on R or RIsa arm (R2 pathway)			Standard-risk MRD-positive participants on RBorD+R or RBorIsaD+RIsa arm (R2 pathway)			High-risk participants (R3 pathway)			All participants
		After cycles 6, 12 and 24 of maintenance *(or at equivalent time-points if randomised to stop receiving Isa)*	After cycle 3	After cycles 6, 12 and 18	After cycle 24	After consolidation cycle 4	After maintenance cycles 3, 9 and 15	After maintenance cycle 21	After consolidation cycle 4	After maintenance cycles 3, 9 and 15	After maintenance cycle 21	Disease progression
**0.5-1 mL EDTA bone marrow aspirate**<br>SEND TO HMDS, LEEDS	Minimal Residual Disease	✓	✓	✓		✓	✓		✓	✓		✓
**10 mL clotted peripheral blood**<br>**Random urine sample**<br>SEND TO BIRMINGHAM	Disease parameters (including paraproteins) for response assessments and covid-19 antibody response<br>M-protein interference assay	✓	✓	✓	✓(blood only)	✓	✓	✓(blood only)	✓	✓	✓(blood only)	✓
**10 mL EDTA bone marrow aspirate**<br>**10 mL blood**<br>SEND TO UCL, LONDON	Tumour DNA for neoantigens<br>cfDNA<br>Immune profiling	✓ (only after 6 cycles)	✓			✓			✓			✓
**Bone marrow trephine**<br>SEND TO UCL, LONDON	Immuno-chemistry for immune function		✓			✓			✓			✓

For peer review only

RADAR: Protocol Paper Attachments

Contents

1 Glossary of Terms 2
2 List of Sites in the RADAR Trial 4
3 Spirit Checklist: Reporting checklist for protocol of a clinical trial. 5
4 Excluded Concomitant Medication: 11
5 Exploratory analysis 13
6 Subgroup analysis 14
7 Informed Consent Material 16
Bone Marrow Consent 16
Part A: Initial Treatment and Further Study Consent 19
Part B: Randomisation 1 Treatment Group 22
Part C: Randomisation 2 Treatment Group 24
Part D: Randomisation 3 Treatment Group 26
8 Central Sample Analysis 28
9 References 31

For peer review only

1 Glossary of Terms

AE	Adverse event
ALT	Alanine aminotransferase
ANC	Absolute neutrophil count
AR	Adverse reaction
ASCT	Autologous stem cell transplantation
β2M	Beta2-microglobulin
BD	Twice daily
CI	Chief Investigator
CR	Complete response
CRF	Case report form
CRUK	Cancer Research United Kingdom
CTCAE	Common Terminology Criteria for Adverse Events
CTRU	Clinical Trials Research Unit
Cy	Cyclophosphamide
D	Dexamethasone
DMEC	Data Monitoring and Ethics Committee
DSUR	Developmental Safety Update Report
ECOG	Eastern Cooperative Oncology Group
eCRF	Electronic Case Report Form
EORTC	European Organisation for Research and Treatment of Cancer
FISH	Fluorescence in situ hybridization test
FWER	Family-wise error rate
GCP	Good Clinical Practice
HDM	High-dose melphalan
HMDS	Haematological Malignancy Diagnostic Service
IMiD®	Immunomodulatory Drug
IMP	Investigational medicinal product
IMWG	International Myeloma Working Group
IPD	Individual participant data
Isa	Isatuximab
ISS	International Staging System
ITT	Intention to treat
LFT	Liver function test
MedDRA	Medical Dictionary for Regulatory Activities
mg	Milligram
MGUS	Monoclonal gammopathy of undetermined significance
MHRA	Medicines and Healthcare Products Regulatory Agency
MM	Multiple myeloma
MR	Minimal response
MRD	Minimal Residual Disease
NCRI	National Cancer Research Institute
NDMM	Newly diagnosed multiple myeloma
NICE	National Institute for Health and Care Excellence
OS	Overall survival
PD	Progressive disease
PFS	Progression-free survival

For peer review only

PFS2	Time to second progression
PI	Principal Investigator
PIS	Patient Information Sheet
PP	Paraprotein
PR	Partial response
PS	Performance status
QoL	Quality of life
R	Lenalidomide
R1-3	Randomisation 1-3
RCT	Randomised controlled trials
RDE	Remote Data Entry
REC	Research Ethics Committee
R-ISS	Revised International Staging System
RSI	Reference Safety Information
SC	Sub-cutaneous
SD	Stable disease
SAE	Serious adverse event
SAP	Statistical analysis plan
SAR	Serious adverse reaction
SmPC	Summary of Product Characteristics
SOP	Standard operating procedure
SPM	Secondary Primary Malignancy
SSOP	Study site operating procedure
SUSAR	Suspected unexpected serious adverse reaction
TE	Transplant eligible
TMG	Trial Management Group
TNE	Transplant non-eligible
TSC	Trial Steering Committee
U&Es	Urea & electrolytes
UK-MRA	United Kingdom Myeloma Research Alliance
ULN	Upper limit of normal
VGPR	Very good partial response

For peer review only

2 List of Sites in the RADAR Trial

Aberdeen Royal Infirmary	Royal Bournemouth Hospital	University Hospital Wishaw
Royal Sussex County Hospital	Poole General Hospital	St Richard's Hospital
Chesterfield Royal Hospital	Salisbury District Hospital	Worthing Hospital
Derriford Hospital	Sunderland Royal Hospital	The Christie
Kent & Canterbury Hospital, Canterbury	South Tyneside District Hospital	Cheltenham General Hospital
Queen Elizabeth the Queen Mother Hospital	Southmead Hospital Bristol	Gloucestershire Royal Hospital
William Harvey Hospital	St. Helens	The Clatterbridge Centre
Freeman Hospital	Whiston Hospital	
Queen Elizabeth Hospital Gateshead	St George's Hospital (Tooting)	
Hammersmith Hospital	Torbay Hospital	
Manchester Royal Infirmary	University College London Hospital	
Wythenshawe Hospital	Leicester Royal Infirmary	
Milton Keynes University Hospital	Worcestershire Royal Hospital	
Perth Royal Infirmary	Alexandra Hospital, Redditch	
Ninewells Hospital	York Hospital	
Northampton General Hospital	University Hospital of Wales, Cardiff	
Churchill Hospital	Stratford Hospital	
Horton General Hospital	Warwick Hospital	
Queen Alexandra Hospital, Portsmouth	New Cross Hospital	
Queen Elizabeth Hospital, Birmingham	University Hospital Coventry	
Raigmore Hospital, Inverness	University Hospital Monklands	
Rotherham General Hospital	University Hospital Hairmyres	

For peer review only

3 Spirit Checklist: Reporting checklist for protocol of a clinical trial.

Based on the SPIRIT guidelines.

Chan A-W, Tetzlaff JM, Gøtzsche PC, Altman DG, Mann H, Berlin J, Dickersin K, Hróbjartsson A, Schulz KF, Parulekar WR, Krleža-Jerić K, Laupacis A, Moher D. SPIRIT 2013 Explanation and Elaboration: Guidance for protocols of clinical trials. BMJ. 2013;346:e7586

		Reporting Item	Page Number
**Administrative information**			
Title	#1	Descriptive title identifying the study design, population, interventions, and, if applicable, trial acronym	1
Trial registration	#2a	Trial identifier and registry name. If not yet registered, name of intended registry	1,2,3
Trial registration: data set	#2b	All items from the World Health Organization Trial Registration Data Set	NA
Protocol version	#3	Date and version identifier	29
Funding	#4	Sources and types of financial, material, and other support	32
Roles and responsibilities: contributorship	#5a	Names, affiliations, and roles of protocol contributors	1, 31
Roles and responsibilities: sponsor contact information	#5b	Name and contact information for the trial sponsor	31,32
Roles and responsibilities: sponsor and funder	#5c	Role of study sponsor and funders, if any, in study design; collection, management, analysis, and interpretation of data; writing of the report; and the decision to submit the report for publication, including whether they will have ultimate authority over any of these activities	32
Roles and responsibilities: committees	#5d	Composition, roles, and responsibilities of the coordinating centre, steering committee, endpoint adjudication committee, data management team, and other individuals or groups overseeing the trial, if applicable (see Item 21a for data monitoring committee)	26,27
**Introduction**			
Background and rationale	#6a	Description of research question and justification for undertaking the trial, including summary of relevant	6,7

For peer review only

		studies (published and unpublished) examining benefits and harms for each intervention	
Background and rationale: choice of comparators	#6b	Explanation for choice of comparators	6,7
Objectives	#7	Specific objectives or hypotheses	7, 18
Trial design	#8	Description of trial design including type of trial (eg, parallel group, crossover, factorial, single group), allocation ratio, and framework (eg, superiority, equivalence, non-inferiority, exploratory)	8
**Methods: Participants, interventions, and outcomes**			
Study setting	#9	Description of study settings (eg, community clinic, academic hospital) and list of countries where data will be collected. Reference to where list of study sites can be obtained	8
Eligibility criteria	#10	Inclusion and exclusion criteria for participants. If applicable, eligibility criteria for study centres and individuals who will perform the interventions (eg, surgeons, psychotherapists)	8-14
Interventions: description	#11a	Interventions for each group with sufficient detail to allow replication, including how and when they will be administered	15-17
Interventions: modifications	#11b	Criteria for discontinuing or modifying allocated interventions for a given trial participant (eg, drug dose change in response to harms, participant request, or improving / worsening disease)	17
Interventions: adherence	#11c	Strategies to improve adherence to intervention protocols, and any procedures for monitoring adherence (eg, drug tablet return; laboratory tests)	17
Interventions: concomitant care	#11d	Relevant concomitant care and interventions that are permitted or prohibited during the trial	17
Outcomes	#12	Primary, secondary, and other outcomes, including the specific measurement variable (eg, systolic blood pressure), analysis metric (eg, change from baseline, final value, time to event), method of aggregation (eg, median, proportion), and time point for each outcome. Explanation of the clinical relevance of chosen efficacy and harm outcomes is strongly recommended	18

For peer review only

Participant timeline	#13	Time schedule of enrolment, interventions (including any run-ins and washouts), assessments, and visits for participants. A schematic diagram is highly recommended (see Figure)	18,19
Sample size	#14	Estimated number of participants needed to achieve study objectives and how it was determined, including clinical and statistical assumptions supporting any sample size calculations	20
Recruitment	#15	Strategies for achieving adequate participant enrolment to reach target sample size	23
**Methods: Assignment of interventions (for controlled trials)**			
Allocation: sequence generation	#16a	Method of generating the allocation sequence (eg, computer-generated random numbers), and list of any factors for stratification. To reduce predictability of a random sequence, details of any planned restriction (eg, blocking) should be provided in a separate document that is unavailable to those who enrol participants or assign interventions	23
Allocation concealment mechanism	#16b	Mechanism of implementing the allocation sequence (eg, central telephone; sequentially numbered, opaque, sealed envelopes), describing any steps to conceal the sequence until interventions are assigned	23
Allocation: implementation	#16c	Who will generate the allocation sequence, who will enrol participants, and who will assign participants to interventions	23
Blinding (masking)	#17a	Who will be blinded after assignment to interventions (eg, trial participants, care providers, outcome assessors, data analysts), and how	NA
Blinding (masking): emergency unblinding	#17b	If blinded, circumstances under which unblinding is permissible, and procedure for revealing a participant's allocated intervention during the trial	NA
**Methods: Data collection, management, and analysis**			
Data collection plan	#18a	Plans for assessment and collection of outcome, baseline, and other trial data, including any related processes to promote data quality (eg, duplicate measurements, training of assessors) and a description of study instruments (eg, questionnaires,	23

For peer review only

		laboratory tests) along with their reliability and validity, if known. Reference to where data collection forms can be found, if not in the protocol	
Data collection plan: retention	#18b	Plans to promote participant retention and complete follow-up, including list of any outcome data to be collected for participants who discontinue or deviate from intervention protocols	23
Data management	#19	Plans for data entry, coding, security, and storage, including any related processes to promote data quality (eg, double data entry; range checks for data values). Reference to where details of data management procedures can be found, if not in the protocol	23
Statistics: outcomes	#20a	Statistical methods for analysing primary and secondary outcomes. Reference to where other details of the statistical analysis plan can be found, if not in the protocol	18
Statistics: additional analyses	#20b	Methods for any additional analyses (eg, subgroup and adjusted analyses)	24
Statistics: analysis population and missing data	#20c	Definition of analysis population relating to protocol non-adherence (eg, as randomised analysis), and any statistical methods to handle missing data (eg, multiple imputation)	24
**Methods: Monitoring**			
Data monitoring: formal committee	#21a	Composition of data monitoring committee (DMC); summary of its role and reporting structure; statement of whether it is independent from the sponsor and competing interests; and reference to where further details about its charter can be found, if not in the protocol. Alternatively, an explanation of why a DMC is not needed	27
Data monitoring: interim analysis	#21b	Description of any interim analyses and stopping guidelines, including who will have access to these interim results and make the final decision to terminate the trial	27
Harms	#22	Plans for collecting, assessing, reporting, and managing solicited and spontaneously reported adverse events and other unintended effects of trial interventions or trial conduct	27
Auditing	#23	Frequency and procedures for auditing trial conduct, if any, and whether the process will be independent from investigators and the sponsor	29

For peer review only

Ethics and dissemination

Research ethics approval	#24	Plans for seeking research ethics committee / institutional review board (REC / IRB) approval	29
Protocol amendments	#25	Plans for communicating important protocol modifications (eg, changes to eligibility criteria, outcomes, analyses) to relevant parties (eg, investigators, REC / IRBs, trial participants, trial registries, journals, regulators)	29
Consent or assent	#26a	Who will obtain informed consent or assent from potential trial participants or authorised surrogates, and how (see Item 32)	30
Consent or assent: ancillary studies	#26b	Additional consent provisions for collection and use of participant data and biological specimens in ancillary studies, if applicable	30,31
Confidentiality	#27	How personal information about potential and enrolled participants will be collected, shared, and maintained in order to protect confidentiality before, during, and after the trial	30
Declaration of interests	#28	Financial and other competing interests for principal investigators for the overall trial and each study site	32
Data access	#29	Statement of who will have access to the final trial dataset, and disclosure of contractual agreements that limit such access for investigators	30
Ancillary and post trial care	#30	Provisions, if any, for ancillary and post-trial care, and for compensation to those who suffer harm from trial participation	32
Dissemination policy: trial results	#31a	Plans for investigators and sponsor to communicate trial results to participants, healthcare professionals, the public, and other relevant groups (eg, via publication, reporting in results databases, or other data sharing arrangements), including any publication restrictions	31
Dissemination policy: authorship	#31b	Authorship eligibility guidelines and any intended use of professional writers	31
Dissemination policy: reproducible research	#31c	Plans, if any, for granting public access to the full protocol, participant-level dataset, and statistical code	31

Appendices

Informed consent materials	#32	Model consent form and other related documentation given to participants and authorised surrogates	(Supplementary Material)

For peer review only

Biological specimens	#33	Plans for collection, laboratory evaluation, and storage of biological specimens for genetic or molecular analysis in the current trial and for future use in ancillary studies, if applicable	(Supplementary Material)

None The SPIRIT Explanation and Elaboration paper is distributed under the terms of the Creative Commons Attribution License CC-BY-NC. This checklist can be completed online using https://www.goodreports.org/, a tool made by the EQUATOR Network in collaboration with Penelope.ai

For peer review only

4 Excluded Concomitant Medication:

Excluded concomitant Medication:
1. Systemic treatment with any of the following metabolising enzyme inducers and inhibitors should be avoided, unless there is no appropriate alternative medication for the participant's use (if there were to be a drug-drug interaction with an inducer, cyclophosphamide and bortezomib metabolite exposure would be increased):<br>a. Strong CYP3A inducers: rifampin, rifapentine, rifabutin, carbamazepine, phenytoin, and phenobarbital<br>b. Strong CYP3A inhibitors<br><br>2. Excluded foods and dietary supplements include St. John's Wort, green tea and supplements containing green tea extracts<br><br>3. The following medicinal products and procedures are **prohibited** during this study:<br>a. Any antineoplastic treatment with activity against MM, other than study drugs<br>b. Platelet transfusions within 3 days prior to study drug dosing for any dosing day, or prior to randomisation in order to meet eligibility criteria.<br>c. Additional steroid therapy (not as trial IMP) (except before commencing trial treatment up to a maximum of 160mg dexamethasone equivalent as per inclusion criteria or steroids used to manage hypersensitivity to trial medication)<br>d. Radiotherapy for the treatment of symptoms related to disease progression.<br>e. Continuous use of clarithromycin (short courses of 5-10 days are permitted for treatment of infective episodes when indicated)<br>f. Hormone replacement therapy and erythropoietic agents and other agents that increase the risk of thrombosis (applicable during treatment with lenalidomide)

For peer review only

Quality of Life and Health Economics questionnaires

Timepoints for all participants up to 100 days post-ASCT

- Baseline
- End of induction
- 100 days post-ASCT

***R1 pathway participants**

- After cycles 6, 12, 24 and 36 (for participants randomised to 'stop isatuximab' at R1, the cycle 24 and 36 questionnaires should still be completed at equivalent timepoints)

***Timepoints for R2 and R3 pathway participants receiving R or RIsa**

- After cycles 6, 12 and 24 of maintenance

***Timepoints for R2 and R3 pathway participants receiving RBorD+R or RBorIsaD+RIsa**

- After cycles 3, 9 and 21 of maintenance.

**Note that participants who stop trial treatment should complete QoL at the equivalent time points (unless the participant wishes to withdraw consent for any further data collection)*

For peer review only

5 Exploratory analysis

Exploratory analysis will consider; the effect of post-ASCT consolidation regimens utilising combinations of lenalidomide, bortezomib, dexamethasone, and isatuximab on immune function in the bone marrow of participants following ASCT, using high dimensional characterisation of the immune microenvironment, immune subsets, phenotype and transcriptome; T cell receptor (TCR) repertoire in the bone marrow of participants following ASCT and consolidation and/or maintenance; correlation of baseline immune profile, TCR diversity and function with disease response, including MRD status and timing of disease relapse; ex-vivo functional studies with participant derived samples used to generate explants or immune-competent patient derived xenografts (icPDX); and to review infection and re-infection rates of COVID-19 in participants during induction, post-ASCT and during post-ASCT consolidation and maintenance treatment. All exploratory analysis to be conducted by the trial statisticians will be pre-specified in the statistical analysis plan.

For peer review only

6 Subgroup analysis

Subgroup analysis will be conducted to determine whether a selection of patient characteristics, haematology / local serological results, biochemistry results and cytogenetic/molecular results as well as response assessments (full list below) are prognostic of the endpoints; PFS, PFS2, OS, attainment of ≥VGPR and attainment of MRD negativity, as defined for all randomisations. Note that the subgroups listed under the Response subheading will not be analysed for the last two endpoints.

Patient Characteristics

- Age (years) at trial registration (≤65, >65)
- Sex (Male / Female)
- ECOG performance status at trial registration (0, 1, ≥2)
- ISS at trial registration (Stage I, Stage II, Stage III)
- R-ISS at trial registration (Stage I, Stage II, Stage III)

Haematology / Local Serological Results

- Haemoglobin concentration at trial registration (<100, ≥100 g/L)
- Platelets at trial registration (<150, ≥150 x10^9/L)
- White blood cells at trial registration (< LLN (Lower limit of normal), ≥LLN)
- Neutrophil count at trial registration (<LLN, ≥LLN)
- Lymphocyte count at trial registration (<LLN, ≥LLN)
- Plasma cells (%) in bone marrow at trial registration (<60%, ≥60%)

Biochemistry Results

- β-2 microglobulin concentration at trial registration (<3.5, 3.5–<5.5, ≥5.5 mg/L)
- Serum creatinine concentration at trial registration (<175, ≥175 µmol/L)
- Corrected serum calcium concentration at trial registration (<2.75, ≥2.75 mmol/L)
- Serum albumin at trial registration (<ULN (Upper limit of normal) , ≥ULN)
- Lactate dehydrogenase at trial registration (<ULN, ≥ULN)
- C-Reactive protein at trial registration (<ULN, ≥ULN)
- Serum bilirubin at trial registration (<ULN, ≥ULN)
- ALT/AST at trial registration (<ULN, ≥ULN)

Cytogenetic/molecular Results

- t(4,14) at trial registration (detected, not detected)
- t(14,16) at trial registration (detected, not detected)
- t(14,20) at trial registration (detected, not detected)
- del(17p) at trial registration (detected, not detected)
- 1q gain at trial registration (detected, not detected)
- Hyperdiploidy at trial registration (detected, not detected)
- The number of high-risk adverse lesions at trial registration (R1 and R2: 0, 1 unable to determine, R3: 2, ≥3, or unable to determine)

Response

For peer review only

- Response to RCyBorD induction (<VGPR, ≥ VGPR)
- Response at 100 days post-ASCT (<VGPR, ≥ VGPR)
- Response at the end of consolidation (<VGPR, ≥ VGPR)
- Response to 12m post-ASCT treatment (<VGPR, ≥ VGPR)
- MRD response to RCyBorD induction (R3 Only: MRD positive, MRD Negative)
- MRD at 100 days post-ASCT (R3 Only: MRD positive, MRD negative)
- MRD response to 6m post-ASCT treatment (R3 Only: MRD positive, MRD Negative)
- MRD response to 12m post-ASCT treatment (R3 Only MRD positive, MRD Negative)

Further subgroup analyses may be undertaken, as appropriate and all the subgroups will be fully defined within the statistical analysis plan according to the current criteria [1-3].

For peer review only

7 Informed Consent Material

Bone Marrow Consent

Delete this line, then print on Trust/Hospital headed paper

Participant ID:	Initials:
Date of Birth:	NHS or CHI number for Scotland/Hospital Number :
EudraCT Number:	Principal Investigator:

CANCER RESEARCH UK

RADAR (UK-MRA Myeloma XV)

INFORMED CONSENT DOCUMENT FOR BONE MARROW AND BLOOD SAMPLE

Please initial each box

1. I confirm that I have read and understand the information sheet for the bone marrow sample request and have had the opportunity to ask questions. ☐

2. I understand that my providing bone marrow samples is voluntary and that I am free to withdraw at any time without my medical care or legal rights being affected. ☐

3. I understand that the samples will be sent along with information about me, which will include NHS number or CHI number for Scotland and may include my name. ☐

4. I agree to these samples being stored and used for research investigations that form part of the RADAR study, and understand that this will include genetic research (for example, analysis of DNA). ☐

For peer review only

5. I understand that a unique reference number will be allocated to the samples which may allow them to be linked back to me in future for research purposes.

6. I understand that my data may be shared on a collaborative basis with researchers in the UK and potentially, centres abroad.

7. I agree to allow any information provided to be used for healthcare and/or further medical research upon the understanding that my identity will remain anonymous wherever possible.

8. I understand that results of my laboratory tests may be shared with other research teams if I am taking part in another clinical study being run by the University of Leeds.

9. I agree to a copy of this Consent Form being sent to the Clinical Trials Research Unit.

10. I agree for my details (which will include date of birth and NHS number or CHI number for Scotland) to be submitted to NHS Digital or other central UK NHS bodies so that information about my health, and mortality should this become available, may be obtained by the CTRU if necessary.

11. I agree to provide bone marrow samples.

Please indicate your wishes in the below scenarios:

Please initial

A) If I am diagnosed with a plasma cell dyscrasia, other than myeloma, I give permission for the samples sent to the central laboratories to be stored and used in future research that receives ethical approval. I understand that the samples and data collected from them may be shared with researchers, possibly in other countries.

Yes No

B) If I am diagnosed with myeloma but do not take part in the RADAR study, I give permission for the samples sent to the central laboratories to be stored and used in future research that receives ethical approval. I understand that the samples and data collected from them may be shared with researchers, possibly outside the EEA.

Yes No

Patient (to be completed by the patient):

Signature…….

Name (block capitals)……………………………………………………………………………………………………….

Date……..

Investigator:

For peer review only

I have explained the request to the above named patient and he/she has indicated his/her willingness for samples of bone marrow to be sent to the RADAR laboratories.

Signature…………………………………………….……………………………………………………………………

Name (block capitals)……………………………………….………………………………………………………….

Date………………………………………………….………………………………………………………………………

(If used)Translator:

Signature…………………………………………….……………………………………………………………………

Name (block capitals)……………………………………….………………………………………………………….

Date………………………………………………….………………………………………………………………………

(1 copy for patient; 1 for the CTRU; 1 held in patient notes, original stored in Investigator Site File)

For peer review only

Part A: Initial Treatment and Further Study Consent

Delete this line, then print on Trust/Hospital headed paper

Participant ID:	Initials:
Date of Birth:	NHS or CHI number for Scotland/Hospital Number:
EudraCT Number:	Principal Investigator:

RADAR (UK-MRA Myeloma XV)

INFORMED CONSENT DOCUMENT PART A

Initial Treatment and Further Study Information

PLEASE INITIAL EACH BOX

12. I confirm that I have read and understand the Part A information document for the RADAR study and have had the opportunity to ask questions.

13. I understand that my participation in this study is voluntary and that I am free to withdraw at any time without my medical care or legal rights being affected. I understand that even if I withdraw from the above study, the data and samples collected from me will be used in analysing the results of the study, and in some cases further information about any unwanted effects of my treatment may need to be collected by the study team.

14. I understand that my healthcare records may be looked at by authorised individuals from the study team, regulatory bodies or Sponsor in order to check that the study is being carried out correctly.

15. I am aware that I am required to have samples taken and sent to laboratories and I understand that this will include genetic research (for example, analysis of DNA). I understand that the samples will be sent along with information about me, which will include NHS number, or CHI number for Scotland, and may include my name.

For peer review only

16. I understand that results or reports from imaging scans, blood tests or other investigations (including for additional cancers) may be sent to the Clinical Trials Research Unit for central review by the research team.

17. I agree to allow any information or results arising from this study to be used for healthcare and/or further medical research upon the understanding that my identity will remain anonymous wherever possible.

18. I understand that my data and results of my laboratory tests (blood, urine and bone marrow sample collections) may be shared with other research teams if I am taking part in another clinical study being run by the University of Leeds.

19. I agree for my details (which will include date of birth and NHS number or CHI number for Scotland) to be submitted to NHS Digital or other central UK NHS bodies so that information about my health may be obtained by the CTRU if necessary.

20. I understand that my GP, or any other doctor treating me, will be notified of my participation in this study.

21. I understand that some of my data may be passed to other organisations, such as the pharmaceutical companies Sanofi and Celgene, (possibly in other countries where the data protection standards and laws may be different from the UK) to monitor the safety of the treatment(s) that I am receiving. I understand that my identity will remain anonymous.

22. I agree to a copy of this Consent Form being sent to the Clinical Trials Research Unit.

23. I agree to take part in the study.

The following parts of the study are OPTIONAL (see section 3 of the Participant Information Sheet). Even if you agree to take part in the RADAR study, you do not have to agree to these optional parts. Please initial yes or no.

Please initial

A) I agree to take part in the Quality of Life and Healthcare Resource Use studies by completing questionnaires at timepoints throughout the study.

Yes No

B) I give permission for samples that I provide to be used in future research that receives ethical approval, and I understand that this includes the bone marrow samples provided at the time of my diagnosis (if I consented separately to collection of the diagnostic bone marrow sample in anticipation of participation in this study). I understand that my samples and data collected from them may be shared on a collaborative basis with researchers in the UK and, potentially, centres abroad.

Yes No

For peer review only

Patient (to be completed by the patient):

I agree to take part in this study.

Signature…….

Name (block capitals)……………………………………………………………………………………………………….

Date……....

Investigator:

I have explained the study to the above named patient, and he/she has indicated his/her willingness to participate in this study.

Signature…….

Name (block capitals)……………………………………………………………………………………………………….

Date……….

(If used) Translator:

Signature…….

Name (block capitals)……………………………………………………………………………………………………….

Date……….

(1 copy for patient; 1 for the CTRU; 1 held in patient notes, original stored in Investigator Site File)

For peer review only

Part B: Randomisation 1 Treatment Group

Delete this line, then print on Trust/Hospital headed paper

Participant ID:	Initials:
Date of Birth:	NHS or CHI number for Scotland/Hospital Number:
EudraCT Number:	Principal Investigator:

CANCER RESEARCH UK

RADAR (UK-MRA Myeloma XV)

INFORMED CONSENT DOCUMENT PART B

R1 Treatment Group

PLEASE INITIAL EACH BOX

24. I confirm that I have read and understand the Part B information document for the RADAR study and have had the opportunity to ask questions. ☐

25. I agree to continue my participation in the study as part of the R1 Treatment Group. ☐

Patient (to be completed by the patient):

I agree to take part in this study.

Signature………..

Name (block capitals)……………………………………………………………………………………………………….

Date……..

Investigator:

I have explained the study to the above named patient, and he/she has indicated his/her willingness to participate in this study.

Signature………..

Name (block capitals)……………………………………………………………………………………………………….

Date……..

For peer review only

(If used) Translator:

Signature………

Name (block capitals)………………………………………………………………………………………………….

Date……

(1 copy for patient; 1 for the CTRU; 1 held in patient notes, original stored in Investigator Site File)

For peer review only

Part C: Randomisation 2 Treatment Group

Delete this line, then print on Trust/Hospital headed paper

Participant ID:	Initials:
Date of Birth:	NHS or CHI number for Scotland/Hospital Number:
EudraCT Number:	Principal Investigator:

CANCER RESEARCH UK

RADAR (UK-MRA Myeloma XV)

INFORMED CONSENT DOCUMENT PART C

R2 Treatment Group

PLEASE INITIAL EACH BOX

26. I confirm that I have read and understand the Part C information sheet for the RADAR study and have had the opportunity to ask questions. ☐

27. I agree to continue my participation in the study as part of the R2 Treatment Group. ☐

Patient (to be completed by the patient):

I agree to take part in this study.

Signature…….

Name (block capitals)……………………………………………………………………………………………….

Date……....

Investigator:

I have explained the study to the above named patient, and he/she has indicated his/her willingness to participate in this study.

Signature…….

Name (block capitals)……………………………………………………………………………………………….

For peer review only

Date………

(If used) Translator:

Signature……

Name (block capitals)……………………………………………………………………………………………………..

Date………

(1 copy for patient; 1 for the CTRU; 1 held in patient notes, original stored in Investigator Site File)

For peer review only

Part D: Randomisation 3 Treatment Group

Delete this line, then print on Trust/Hospital headed paper

Participant ID:	Initials:
Date of Birth:	NHS or CHI number for Scotland/Hospital Number:
EudraCT Number:	Principal Investigator:

CANCER RESEARCH UK

RADAR (UK-MRA Myeloma XV)

INFORMED CONSENT DOCUMENT PART D

R3 Treatment Group

PLEASE INITIAL EACH BOX

28. I confirm that I have read and understand the Part D information sheet for the RADAR study and have had the opportunity to ask questions. ☐

29. I agree to continue my participation in the study as part of the R3 Treatment Group. ☐

Patient (to be completed by the patient):

I agree to take part in this study.

Signature………

Name (block capitals)……………………………………………………………………………………………

Date………

Investigator:

I have explained the study to the above named patient, and he/she has indicated his/her willingness to participate in this study.

Signature………

Name (block capitals)……………………………………………………………………………………………

Date………

For peer review only

(If used) Translator:

Signature……………………………………………..………………………………………………………………………

Name (block capitals)……………………………………………..………………………………………………….

Date……………………………………………..………………………………………………………………………

(1 copy for patient; 1 for the CTRU; 1 held in patient notes, original stored in Investigator Site File)

For peer review only

8 Central Sample Analysis

The trial has 3 central laboratories receiving samples for trial purposes. The time points and the reason for analysis for each central sample can be found in Figure 2. Details on the analysis of each sample is below. Note that participants can consent for their samples to be used in future research and hence each laboratory can store their trial samples for use in future studies. The storage details are also included below.

Birmingham

The Birmingham University Clinical Immunology Laboratory holds UKAS ISO 15189:2012 - Medical Laboratories accreditation. The Trial-related Laboratory Work will be performed using standard assays to the same quality standard as during routine clinical care. The Laboratory will be responsible for the analysis of whole clotted blood, serum and urine collected from participants of the Research Project in accordance with the Protocol. The laboratory will measure disease response and Disease progression, defined according to the IMWG 2016 response criteria for Multiple Myeloma [21 26-28]. This will include assessment of prognostic markers, markers of disease activity and M-protein levels. Analyses will include serum levels of beta-2 microglobulin, immunoglobulins IgG, IgA and IgM, kappa and lambda free immunoglobulin light chains. Serum electrophoresis and immunofixation, M-protein quantitation including total protein and densitometry. Urine immunofixation and free light chain quantitation will be performed where appropriate. As the trial proceeds new biomarkers of myeloma disease activity are likely to become available and will also be measured. The Laboratory will ensure that the Materials are received, handled, analysed, stored and destroyed in line with the individual consent provided.

HMDS

The presence of minimal residual disease will be assessed in bone marrow aspirates using a validated flow cytometry assay (sensitivity ⩽0.001%) performed at a single central laboratory (HMDS, Leeds Teaching Hospitals Trust). A minimum of 2,500,000 cells will be evaluated with an eight-colour antibody combination of CD138, CD38, CD45, CD19, CD56, CD27, CD81 and CD117. Additional antigens for detection of CD38-negative plasma cells will be utilised as required. % neoplastic plasma cells and % normal plasma cells as a percentage of leucocytes will be reported. Limit of detection (LoD) and limit of quantification (LoQ) will also be calculated for each sample.
Samples will be used for trial purposes only and will not be stored for future research.

For peer review only

UCL

Bone marrow aspirates, peripheral blood and bone marrow trephine processing within UCL Myeloma Lab for RADAR clinical trial

OBJECTIVES

To provide molecular and genetic data on bone marrow immune profiles of newly diagnosed myeloma patients prior and post autologous stem cell transplantation. This will also be analysed between study arms to compare how different treatment regimes alert immune landscape in patients in particular anti-CD38 mAb Isatuximab.

METHODS

BMA and PB:

1. Sites are requested to collect samples and ship as per Lab Manual together with completed sample request form.

2. All samples will be processed in Myeloma Lab at UCL Cancer Institute.

3. UCL ECMC GCLP Facility will serve as storage facility for the following materials: PB plasma, BM serum, pellets (BM, PB, CD138+ and CD138-), protein lysates, extracted RNA and DNA. All material to be stored at -80˚C.

4. Myeloma Lab will serve as processing facility for all samples and storing of the following materials: BM MNCs (mononuclear cells), PB MNCs, CD138+ cells and CD138- cells. All material to be stored in liquid nitrogen.

5. Both -80˚C freezers and liquid nitrogen tanks are temperature monitored with systems that alert when temperature is out of acceptable range.

6. All samples and material extracted will be logged into the trial specific lab book and database with a unique trial ID, lab ID and time-point.

7. Upon arrival bone marrow aspirates and peripheral blood will be centrifuged. Serum (from BMA) and plasma (from PB) will be collected, aliquoted into 1-1.5ml aliquots and frozen at -80˚C.

8. BMA and PB mononuclear cells will then be isolated and enriched using Ficoll density gradient centrifugation.

9. PB MNCs will then be frozen as pellets and/or cryopreserved.

10. Depending on the tumour burden in the BM MNCs samples will be either frozen as MNCs pellets and/or cryopreserved or tumour cells will be isolated.

11. Tumour will be isolated via column-based MACS cell positive selection using CD138 magnetic beads (MicroBeads).

12. Depending on the number of cells isolated, CD138+ plasma cells will be frozen and stored as pellets and/or protein lysates and/or cryopreserved.

13. CD138- cells will be frozen and stored as pellets and/or cryopreserved.

Bone marrow trephine:

1. Sites are requested to collect, process and ship samples as per Lab Manual together with completed sample request form.

2. Myeloma lab will serve as storage facility for bone marrow trephine slides.

3. Upon arrival all samples will be logged into trial specific database with a unique trial ID, lab ID and time-point.

4. Upon arrival slides will be labelled and stored in the air-tight slide storage box.

ANALYSIS

1. High-dimensional flow cytometry will be used to profile immune context of patient bone marrow mononuclear cells prior and post-transplant.

2. Selected samples will be analysed further using transcriptomic profiling, T cell receptor (TCR) sequencing or single cell sequencing and multiplex immunofluorescence/immunohistochemistry on bone marrow trephine biopsies.

For peer review only

9 References

1. Oxman, A.D., *Subgroup analyses.* BMJ, 2012. **344**.
2. Sun, X., et al., *Is a subgroup effect believable? Updating criteria to evaluate* the *credibility of subgroup analyses.* BMJ, 2010. **340**.
3. Sun, X., et al., *Credibility of claims of subgroup effects in randomised controlled trials: systematic review.* BMJ, 2012. **344**.

For peer review only

BMJ Open

Risk and response adapted therapy following autologous stem cell transplant in patients with newly diagnosed multiple myeloma (RADAR (UK-MRA Myeloma XV Trial): study protocol for a Phase II/III randomised controlled trial

Journal:	*BMJ Open*
Manuscript ID	bmjopen-2022-063037.R2
Article Type:	Protocol
Date Submitted by the Author:	26-Sep-2022
Complete List of Authors:	Royle, Kara-Louise; University of Leeds Clinical Trials Research Unit<br>Coulson, Amy; University of Leeds Clinical Trials Research Unit<br>Ramasamy, Karthik; Oxford University Hospitals NHS Trust, Haematology; Oxford University, Radcliffe Department of Medicine<br>Cairns, David; University of Leeds Clinical Trials Research Unit<br>Hockaday, Anna; University of Leeds Clinical Trials Research Unit<br>Quezada, Sergio; UCL Cancer Institute<br>Drayson, Mark ; University of Birmingham<br>Kaiser, Martin; Institute of Cancer Research<br>Owen, Roger; St James's University Hospital, HMDS<br>Auner, Holger; Imperial College London, Department of Immunology and Inflammation; Imperial College London, Langmuir Centre for Myeloma Research<br>Cook, Gordon; University of Leeds Clinical Trials Research Unit; St James's University Hospital, Leeds Cancer Centre<br>Meads, David; University of Leeds, Leeds Institute of Health Sciences<br>Olivier, Catherine; University of Leeds Clinical Trials Research Unit<br>Barnard, Lorna; University of Leeds Clinical Trials Research Unit<br>Lambkin, Rhiannon; University of Leeds Clinical Trials Research Unit<br>Paterson, Andrea; University of Leeds Clinical Trials Research Unit<br>Dawkins, Bryony ; University of Leeds Leeds Institute of Health Sciences, Academic Unit of Health Economics<br>Chapman, Mike; University of Cambridge, Department of Haematology<br>Pratt, Guy; Queen Elizabeth Hospital<br>Popat, Rakesh; University College London Hospitals NHS Foundation Trust<br>Jackson, Graham; Leeds Teaching Hospitals NHS Trust, Department of Haematology<br>Bygrave, Ceri; University Hospital of Wales<br>Sive, Jonathan; University College London Hospitals NHS Foundation Trust<br>de Tute, Ruth; St James's University Hospital, HMDS<br>Chantry, Andrew; Royal Hallamshire Hospital, Department of Haematology<br>Parrish, Christopher; Leeds Teaching Hospitals NHS Trust, Haematology<br>Cook, Mark; University of Birmingham Institute of Cancer and Genomic Sciences; Bristol Myers Squibb

Asher, Samir; UCL Cancer Institute
Yong, Kwee; UCL Cancer Institute

**Primary Subject Heading**:	Oncology
Secondary Subject Heading:	Haematology (incl blood transfusion)
Keywords:	Myeloma < ONCOLOGY, Myeloma < HAEMATOLOGY, Clinical trials < THERAPEUTICS, Cancer genetics < GENETICS, STATISTICS & RESEARCH METHODS, Protocols & guidelines < HEALTH SERVICES ADMINISTRATION & MANAGEMENT

SCHOLARONE™ Manuscripts

Risk and response adapted therapy following autologous stem cell transplant in patients with newly diagnosed multiple myeloma (RADAR (UK-MRA Myeloma XV Trial): study protocol for a Phase II/III randomised controlled trial

Kara-Louise Royle[1], Amy Beth Coulson[1], Karthik Ramasamy[2], David A Cairns[1], Anna Hockaday[1], Sergio Quezada[3], Mark Drayson[4], Martin Kaiser[5], Roger Owen[6], Holger W. Auner[7], Gordon Cook[1,8], David Meads[19], Catherine Olivier[1], Lorna Barnard[1], Rhiannon Lambkin[1], Andrea Paterson[1], Bryony Dawkins[19], Mike Chapman[9], Guy Pratt[10], Rakesh Popat[11], Graham Jackson[12], Ceri Bygrave[13], Jonathan Sive[11], Ruth de Tute[6], Andrew Chantry[14], Christopher Parrish[1,16,] Mark Cook[16,17], Samir Asher[18], Kwee Yong [18]

1. Clinical Trials Research Unit, Leeds Institute of Clinical Trials Research, University of Leeds, Leeds, United Kingdom
2. Oxford University Hospitals NHS Foundation Trust, Radcliffe Department of Medicine, Oxford University
3. Cancer Immunotherapy Group, UCL Cancer Institute, 72 Huntley Street, London WC1E 6BT
4. Clinical Immunology Service, Institute of Immunology and Immunotherapy, Medical School, University of Birmingham, Edgbaston, Birmingham, B15 2TT
5. The Institute of Cancer Research (ICR), Centre for Myeloma Research, Division of Molecular Pathology, Brookes Lawley Building, 15 Cotswold Road, London, Surrey, SM2 5NG
6. HMDS, St James's University Hospital, Leeds, LS9 7TF
7. Centre for Haematology, Imperial College London, London, W12 0NN
8. Leeds Cancer Centre, St James's University Hospital, Leeds, United Kingdom
9. Department of Haematology, University of Cambridge, Cambridge, CB2 0XY
10. Department of Haematology; Queen Elizabeth Hospital, Birmingham, B15 2TH
11. University College London Hospitals NHS Foundation Trust, NW1 2BU
12. Northern Centre for Cancer Care, Freeman Hospital, Newcastle-Upon-Tyne, NE7 7DN
13. University Hospital of Wales, Heath Park, Cardiff, CF14 4XW
14. Department of Haematology, Royal Hallamshire Hospital, Sheffield, S10 2RX
15. Leeds Teaching Hospitals NHS Trust, St James's Institute of Oncology, Beckett Street, Leeds, LS9 7TF, UK
16. University of Birmingham Institute of Cancer and Genomic Sciences
17. Bristol Myers Squibb, Boudry Switzerland
18. UCL Cancer Institute, London, WC1E 6BT
19. Academic Unit of Health Economics, Leeds Institute of Health Sciences, University of Leeds, Leeds, United Kingdom

*corresponding author
Miss Kara-Louise Royle,
Clinical Trials Research Unit, Leeds Institute of Clinical Trials Research, University of Leeds, Leeds, United Kingdom, LS2 9JT

Tel: 0113 343 8366

For peer review only

Email: k.l.royle@leeds.ac.uk

Word count: 8343

For peer review only - http://bmjopen.bmj.com/site/about/guidelines.xhtml

Abstract

Introduction

Multiple myeloma is a plasma cell malignancy that accounts for 1-2% of newly diagnosed cancers. At diagnosis, approximately 20% of patients can be identified, using cytogenetics, to have inferior survival (high-risk). Additionally, standard-risk patients, with detectable disease (minimal residual disease (MRD)-positive) post-autologus stem cell transplant (ASCT), fare worse compared to those who do not (MRD-negative). Research is required to determine whether a risk-adapted approach post-ASCT could further improve patient outcomes.

Methods

RADAR is a UK, multi-centre, risk-adapted, response-guided, open-label, randomised controlled trial for transplant-eligible newly diagnosed multiple myeloma patients, using combinations of lenalidomide (R), cyclophosphamide (Cy), bortezomib (Bor), dexamethasone (D) and isatuximab (Isa).

Participants receive RCyBorD(x4) induction therapy, followed by high-dose melphalan and ASCT. Post-ASCT, there are three pathways.

1. A phase III discontinuation design to assess de-escalating therapy in standard-risk MRD-negative patients. Participants receive 12 cycles of Isa maintenance. Those who remain MRD-negative are randomised to either continue or stop treatment.
2. A phase II/III multi-arm multi-stage design to test treatment strategies for treatment escalation in standard-risk MRD-positive patients. Participants are randomised to either; R, RBorD(x4) +R, RIsa, or RBorIsaD(x4) + RIsa.
3. A phase II design to assess the activity of intensive treatment strategies in high-risk patients. Participants are randomised to RBorD(x4) +R or RBorIsaD(x4) + RIsa.

1400 participants will be registered to allow for 500, 450 and 172 participants in each pathway. Randomisations are equal and treatment is given until disease progression or intolerance.

Ethics and dissemination

Ethical approval was granted by the London–Central Research Ethics Committee (20/LO/0238) and capacity and capability confirmed by the appropriate local research and development department for each participating centre prior to opening recruitment. Participant informed consent is required before trial registration and re-confirmed post-ASCT. Results will be disseminated by conference presentations and peer-reviewed publications.

Trial registration

ISCRTN 46841867

Key Words: Newly Diagnosed, Multiple Myeloma, Transplant-eligible, Risk-adapted, Treatment escalation, Treatment de-escalation

For peer review only

Article Summary

Strengths and limitations of the study

Strengths

- RADAR trial treatment is personalised through the utilisation of baseline cytogenetic and dynamic MRD-based stratification within its design which includes a de-escalation strategy to determine whether patients responding to treatment need to continue treatment until progression.
- RADAR is adaptive, new experimental treatment arms are expected to be added as new evidence emerges.

Limitations

- Patients achieving <PR to induction therapy will not proceed with on-study ASCT and will be treated off -trial, there is no trial pathway for these participants.
- RADAR excludes patients with impaired renal impairment (defined as CrCl <30 ml/min)

For peer review only

For peer review only - http://bmjopen.bmj.com/site/about/guidelines.xhtml

Introduction

Multiple Myeloma

Multiple myeloma (MM) is the second most common haematological malignancy with over 5500 patients diagnosed in the UK each year.[1] Whilst there is no established cure, survival has extended markedly in the last 15 years such that patients diagnosed today can expect to live a median of 6 years, and 30% will live for 10 years or longer.[1, 2]

These advances have been made possible due to the now standard use of “novel agents” including proteasome inhibitors and immunomodulatory agents (IMiD® agents) in conjunction with high-dose melphalan (HDM) supported by autologous stem cell transplantation (ASCT). Whilst MM is predominantly a disease of older people, around 40% of patients are young and fit enough at diagnosis to be eligible for ASCT, a strategy shown in several randomised trials (RCT) to extend progression-free survival (PFS).[2-4]

Improved response rates and increased survival poses challenges for the evaluation of new regimens in a timely manner. Recent studies re-evaluating the role of ASCT in the era of novel agents have confirmed superior PFS in patients allocated to ASCT compared to those allocated to chemotherapy with deferred ASCT.[5] No overall survival (OS) benefit has been seen yet, although this probably reflects the frequent use of salvage ASCT at second remission in most trial patients. Such studies utilise post-ASCT continuous therapy (lenalidomide maintenance) [6] that can extend PFS and OS,[7] but may also impact on quality of life (partly due to treatment-related toxicity), and are not without long-term risk.[8] It is thus imperative to identify those patients who are likely to benefit from extended maintenance therapy, in order to reduce health care costs and minimise toxicity for those who do not benefit.

Existing Evidence: Induction Therapy

Treatment with lenalidomide (R), cyclophosophamide (Cy), bortezomib (Bor) and dexamethasone (D) (RCyBorD) was studied as first line therapy in the Phase 2 EVOLUTION study, where it produced high response rates and deep responses compared to CyBorD or LenBorD.[9]

Existing Evidence: High-Risk Patients

At diagnosis, a small subgroup of patients (approximately 20%) can be identified on the basis of genetic features to have inferior survival regardless of treatment regimen (high-risk patients).[10-12] For such patients, and for those patients with refractory disease (no response to first line therapy), outcomes have remained poor despite the introduction of novel agents[13-15]. Whilst high-risk patients often show a good response to induction therapy, the durability of the response is inferior to standard-risk patients. Using the R-ISS which incorporates high risk cytogenetic features into the the International Staging System (ISS), PFS time is 66 months for R-ISS stage I, 42 months for R-ISS stage II, and 29 months for R-ISS stage III)[16]. The attainment of MRD-negative disease is also critical.[17-19] The activity of the RBorD regimen in patients with high-risk disease looks promising[20], and there is a question of whether an additional benefit may be gained through the use of anti-CD38 immunotherapy. Anti-CD38[21, 22] antibodies have single agent activity, but also impressive efficacy in combination, particulary with IMiD agents, lenalidomide and pomalidomide. In the RADAR trial the anti-CD38 antibody isatuximab (Isa) (SAR650984) will be used. Isatuximab is a naked immunoglobulin

For peer review only - http://bmjopen.bmj.com/site/about/guidelines.xhtml

(Ig) G1 derived monoclonal antibody (mAb) that binds selectively to a unqiue epitope on the human surface antigen called CD38 [23].

Existing Evidence: Standard-Risk Patients

With increasingly effective multi-drug regimens being used for induction treatment, and responses further deepened following high-dose therapy and ASCT, the major challenge for standard-risk participants is how to maintain their disease control for as long as possible. The benefit of post-ASCT consolidation approaches remains controversial, with inconsistent evidence identifying both appropriate patient groups and treatment strategies.

Maintenance lenalidomide (R) has been studied within several large studies and has consistently been shown to increase PFS, confimed within a recent meta-analysis.[7] The impact of long-term lenalidomide therapy on bone marrow and immune function, and general wellbeing are not completely understood. Thus there is an urgent need to identify those patients who benefit most from this approach, as well as an evaluation of other agents that utilise the host immune system to improve outcomes, such as antibodies. It is also of benefit to explore the option and effect of stopping maintenance therapy in patients who have ahcieved and maintained the deepest response

Existing Evidence: Use of MRD for stratifying treatment in standard-risk patients

MRD status is a clinically relevant biomarker, and an emerging clinical trial endpoint with recent guidance published by the International Myeloma Working Group (IMWG).[19, 24]

Several studies have shown that patients who have detectable disease in their bone marrow (MRD-positive) following ASCT suffer earlier relapses and shorter survival when compared with those who are MRD-negative.[25, 26] These results have been combined in a recent meta-analysis.[26] Hence these patients should be the focus of investigative strategies aimed at deepening disease response and converting them from MRD-positive to MRD-negative.

Patients without detectable disease in their bone marrow (MRD-negative), have longer PFS and OS .[27] In the IFM2009 study that randomised patients to receive ASCT upfront or to continue chemotherapy, patients who were MRD-negative fared equally well, regardless of whether they had received ASCT, or chemotherapy alone. This would suggest that those who have achieved MRD-negative response may not need to undergo intensive consolidation therapy, and instead may benefit from a de-escalation of therapy.

Aims and objectives

RADAR aims to address the most important contemporary therapeutic questions for newly diagnosed multiple myeloma transplant eligible (NDMM TE) patients post-ASCT by considering three different post-ASCT treatment pathways:

1. A de-escalation treatment strategy is used to determine whether ceasing Isa maintenance treatment, after 12 cycles of continuous treatment, is non-inferior to long-term Isa treatment, in terms of progression-free survival (PFS) in participants who are standard-risk at diagnosis (participants who have ≤1 high-risk cytogenetic lesion) and are MRD-negative at 100 days post-ASCT and 12 months (cycles) after.

For peer review only - http://bmjopen.bmj.com/site/about/guidelines.xhtml

2. A treatment escalation strategy is used for participants who are standard-risk at diagnosis (participants who have ≤1 high-risk cytogenetic lesion) and MRD-positive at 100 days post ASCT. The trial aims to compare the activity and efficacy of post-ASCT consolidation and maintenance treatment between combinations of R, Bor, D and Isa to R alone in terms of conversion to MRD-negativity.
3. Dose-intensive consolidation and maintenance strategies will be studied for participants who are high-risk at diagnosis (participants who have >1 high-risk cytogenetic lesion). The activity of post-ASCT strategies containing R, Bor, D and Isa will be assessed in terms of PFS rate.

Trial Design

RADAR is a national, multi-centre, risk-adapted, response-guided open-label, randomised controlled trial for NDMM TE patients: phase II/III for standard-risk, MRD-Positive participants, phase III randomised discontinuation design for standard-risk, MRD-Negative participants, phase II randomised for high-risk participants. The following report details the trial protocol and follows the structure of the SPIRIT statement.[28] The SPIRIT checklist can be found within the supplementary material.

Methods:

Setting

The RADAR trial will be conducted at 70 centres in the United Kingdom (See supplementary material), as identified via a feasibility assessment to determine those most appropriate to participate in the trial.

Potential participants will be identified by the research team at the time they are referred to the haematology outpatient department with suspected multiple myeloma. A smaller number of participants may be identified during inpatient admissions. Invitation to participate in the trial and provision of information will be made either during their first consultation, when routine diagnostic tests will be performed and potential treatment options discussed, or at the time they receive their diagnostic test results.

Eligibility criteria

Adults (18 years and older) with previously untreated multiple myeloma requiring therapy, as defined by IMWG diagnostic criteria, who are eligible for stem cell transplant and capable of giving informed consent will be assessed for eligibility. Eligibility will be confirmed prior to; registration, the start of isatuximab maintenance treatment in the Randomisation 1 pathway and each randomisation by the principal investigator (PI) or an appropriately authorised medically trained delegate and will be recorded in the participants' medical records and on the relevant electronic case report form (eCRF).

To be eligible for each point in the trial the participants must meet all the inclusion criteria and none of the exclusion criteria presented in Table 1 for trial registration, Table 2 for isatuximab treatment in the R1 pathway, Table 3 for Randomisation 1 (R1), Table 4 for Randomisation 2 (R2), Table 5 for Randomisation 3 (R3).

For peer review only - http://bmjopen.bmj.com/site/about/guidelines.xhtml

Table 1: Inclusion and Exclusion Criteria for Main Trial Registration

Inclusion Criteria:

1. Previously untreated patients with multiple myeloma requiring therapy, defined as having myeloma defining events or with biomarkers of malignancy according to IMWG diagnostic criteria
2. Eligible for stem cell transplant
3. Eastern Cooperative Oncology Group (ECOG) performance status 0–2 (except in cases where ECOG >2 is due to effects of myeloma eg spinal cord compression);
4. Total bilirubin ≤ 1.5 x upper limit of normal (ULN)
5. Alanine aminotransferase (ALT) and/or aspartate aminotransferase (AST) ≤ 3 x ULN (if ALT **and** AST are tested, both must meet this criteria)
6. Adequate marrow function:
    - neutrophils ≥1.0 × 10^9/L (unless the participant has a known/suspected diagnosis of familial or racial neutropenia in which case an ANC ≥0.75 x 10^9/L is allowed),
    - haemaglobin (Hb) ≥ 80g/L. Blood *transfusions within 3 days prior to eligibility assessments are* ***not*** *permitted*,
    - platelets ≥ 75 × 10^9/L (in the case of heavy bone marrow infiltration (>50%) which is, in the opinion of the investigator, the cause of the thrombocytopaenia and provided appropriate supportive measures and patient monitoring are in place, a platelet count of ≥50 × 10^9/L is permitted. *Platelet transfusions within 3 days prior to eligibility assessments are* ***not*** *permitted.*
7. Creatinine clearance (CrCl) ≥ 30 mL/minute, according to the Cockcroft-Gault formula, following correction of reversible causes (e.g. dehydration, hypercalcaemia, sepsis)
8. Able to swallow oral medication
9. Aged at least 18 years
10. Agree to follow the pregnancy prevention guidelines:

    Female participants who:

    a) Are not of childbearing potential, OR

    b) If they are of childbearing potential, agree to practice 2 effective methods of contraception, at the same time, from the time of signing the informed consent form until 12 months after the last dose of cyclophosphamide or 5 months after the last dose of study drug, whichever is longest, OR

    c) Agree to practice true abstinence when this is in line with the preferred and usual lifestyle of the subject from the time of signing the informed consent form until 12 months after the last dose of cyclophosphamide or 5 months after the last dose of study drug, whichever is longest. (Periodic abstinence [e.g. calendar, ovulation, symptothermal, post-ovulation methods] and withdrawal are not acceptable methods of contraception.)

    d) Agree to not donate oocytes during the entire study treatment period and until 12 months after the last dose of cyclophosphamide or 5 months after the last dose of study drug, whichever is longest.

    Male participants, even if surgically sterilised (i.e. status post-vasectomy), must agree to one of the following:

    a) Agree to practice effective barrier contraception during the entire study treatment period and until 6 months after the last dose of cyclophosphamide or 5 months after the last dose of study drug, whichever is longest OR

    b) Agree to practice true abstinence when this is in line with the preferred and usual lifestyle of the subject, during the entire study treatment period and until 6 months after the last dose of cyclophosphamide or 5 months after the last dose of study drug, whichever is longest. (Periodic abstinence (e.g. calendar, ovulation, symptothermal, post-ovulation methods] and withdrawal are not acceptable methods of contraception).

    c) Agree to not donate sperm during the entire study treatment period and until 6 months after the last dose of cyclophosphamide or 5 months after the last dose of study drug, whichever is longest.

    Contraception for female and male participants must be in accordance with (and participants must consent to) the Celgene Pregnancy Prevention Plan.

    If female and of childbearing potential, they must have a negative pregnancy test performed by a healthcare professional within 14 days prior to registration.
11. Able to provide written informed consent.

Exclusion Criteria

1. Asymptomatic (smouldering) MM, monoclonal gammopathy of undetermined significance (MGUS), solitary plasmacytoma of bone, or extramedullary plasmacytoma (without evidence of MM)

For peer review only - http://bmjopen.bmj.com/site/about/guidelines.xhtml

2. Received previous treatment for MM, with the exception of local radiotherapy to relieve bone pain or spinal cord compression, prior bisphosphonate treatment, or corticosteroids as long as the total dose does not exceed the equivalent of 160mg dexamethasone. *This criteria is not applicable at R1, R2 and R3 when participants will have received previous treatment for MM as part of this trial.*<br>3. Unstable angina or myocardial infarction within 4 months prior to registration (or at any time since registration for participants starting isatuximab maintenance, R1, R2 and R3), NYHA Class III or IV heart failure, uncontrolled angina, history of severe coronary artery disease, severe uncontrolled ventricular arrhythmias, sick sinus syndrome, or electrocardiographic evidence of acute ischemia or Grade 3 conduction system abnormalities unless subject has a pacemaker<br>4. Cardiac disorder identified according to local practice (eg left ventricular ejection fraction, LVEF; results from formal measurements acceptable within 28 days prior to registration)<br>5. Significant neuropathy (Grade ≥3, or Grade 2 with pain)<br>6. Prior malignancy that required treatment or has shown evidence of recurrence (except for non-melanoma skin cancer or adequately treated cervical carcinoma in situ) during the 5 years prior to registration. Cancer treated with curative intent for >5 years previously and without evidence of recurrence will be allowed.<br>7. Pregnant, lactating or breastfeeding female participants<br>8. Known resistance, intolerance or hypersensitivity to any component of the planned therapies, except in the case of hypersensitivity which is amenable to premedication with steroids or H2 blocker. Intolerance includes hereditary problems of galactose intolerance, the Lapp lactase deficiency or glucose-galactose malabsorption.<br>9. Major surgery within 14 days before registration (or starting isatuximab maintenance, R1, R2 and R3). This would include surgical intervention for relief of cord compression but does not include vertebroplasty or kyphoplasty.<br>10. Known gastrointestinal (GI) disease or GI procedure that could interfere with the oral absorption or tolerance of trial treatment, including difficulty swallowing.<br>11. Active systemic infection<br>12. Participant is hepatitis B surface antigen positive, hepatitis C antibody positive or HIV positive (participants who are hepatitis C core antibody positive but have been successfully treated for the disease may still be eligible – please consult the CTRU/CI). Participants must have hepatitis and HIV screening conducted within 28 days prior to registration.<br>13. Any other medical or psychiatric condition which, in the opinion of the investigator, contraindicates the participant's participation in this study.<br>14. Receipt of live vaccination within 30 days prior to registration, for the duration of the study and for 3 months after the last dose of study drug.<br>15. Participant has risk factors for thromboembolism including the use of agents which may increase their risk of thrombosis, such as hormone replacement therapy (*this exclusion criteria is applicable only at registration and when starting R2 or R3 pathway*)<br>16. Participant has risk factors for seizures (*this exclusion criteria is applicable only at registration and when starting R2 or R3 pathway*

For peer review only

For peer review only - http://bmjopen.bmj.com/site/about/guidelines.xhtml

Table 2: Inclusion and Exclusion criteria for Isatuximab treatment (R1 Pathway)

Inclusion Criteria:

1. Standard-risk (participant is not **confirmed** to have at least two of these genetically adverse lesions: t(4;14), t(14;16), t(14;20), del(17p), gain(1q), as confirmed by the CTRU.
2. 4 cycles of RCyBorD received
3. MRD-negative (proportion of malignant cells in the bone marrow is < 1 in 100,000, confirmed by HMDS central lab) at 100 days post-ASCT
4. Received ≥100mg/m^2 high-dose melphalan and ASCT
5. Eastern Cooperative Oncology Group (ECOG) performance status 0–2 (except in cases where ECOG >2 is due to effects of myeloma eg spinal cord compression)
6. Total bilirubin <= 1.5 x upper limit of normal (ULN)
7. Alanine aminotransferase (ALT) and/or aspartate aminotransferase (AST) ≤ 3 x ULN (if ALT **and** AST are tested, both must meet this criteria)
8. Adequate marrow function:
   a) neutrophils ≥1.0 × 10^9/L (unless the participant has a known/suspected diagnosis of familial or racial neutropenia in which case an ANC ≥0.75 x 10^9/L is allowed),
   b) Hb≥ 80g/L. Blood transfusions within 3 days prior to eligibility assessments are not permitted,
   c) platelets ≥ 75 × 10^9/L. Platelet transfusions within 3 days prior to eligibility assessments are not permitted
9. Creatinine clearance (CrCl) ≥ 30 mL/minute, according to the Cockcroft-Gault formula, following correction of reversible causes (e.g. dehydration, hypercalcaemia, sepsis)
10. Agree to follow the pregnancy prevention guidelines:
    Female participants who:
    a) Are not of childbearing potential, OR
    b) If they are of childbearing potential, agree to practice 2 effective methods of contraception, at the same time, from the time of signing the informed consent form until 12 months after the last dose of cyclophosphamide or until 5 months after the last dose of study drug, whichever is longest OR
    c) Agree to practice true abstinence when this is in line with the preferred and usual lifestyle of the subject, from the time of signing the informed consent form until 12 months after the last dose of cyclophosphamide or until 5 months after the last dose of study drug, whichever is longest. (Periodic abstinence [e.g. calendar, ovulation, symptothermal, post-ovulation methods] and withdrawal are not acceptable methods of contraception.)
    d) Agree to not donate oocytes during the entire study treatment period until 12 months after the last dose of cyclophosphamide or 5 months after the last dose of study drug, whichever is longest.
    Male participants, even if surgically sterilised (i.e. status post-vasectomy), must agree to one of the following:
    e) Agree to practice effective barrier contraception during the entire study treatment period and until 6 months after the last dose of cyclophosphamide or until 5 months after the last dose of study drug, whichever is longest OR
    f) Agree to practice true abstinence when this is in line with the preferred and usual lifestyle of the subject during the entire study treatment period and until 6 months after the last dose of cyclophosphamide or until 5 months after the last dose of study drug, whichever is longest.. (Periodic abstinence [e.g. calendar, ovulation, symptothermal, post-ovulation methods] and withdrawal are not acceptable methods of contraception).
    g) Agree to not donate sperm during the entire study treatment period until 6 months after the last dose of cyclophosphamide or 5 months after the last dose of study drug, whichever is longest.
11. Signed the Informed Consent Document for the R1 treatment pathway.
    a)

Exclusion Criteria:

1. Disease progression (according to IMWG criteria)
2. MRD-positive (proportion of malignant cells in the bone marrow is ≥ 1 in 100,000, confirmed by HMDS central lab) at 100 days post-ASCT
3. Registration exclusion criteria (cardiac risk assessment, HIV and Hepatitis B and C testing do not need to be repeated)

For peer review only

For peer review only - http://bmjopen.bmj.com/site/about/guidelines.xhtml

Table 3: Inclusion and Exclusion criteria for Randomisation 1

Inclusion Criteria:

1. Standard-risk (participant is not **confirmed** to have at least two of these genetically adverse lesions: t(4;14), t(14;16), t(14;20), del(17p), gain(1q), as confirmed by the CTRU.
2. 12 cycles of isatuximab maintenance received
3. MRD-negative (proportion of malignant cells in the bone marrow is < 1 in 100,000, confirmed by HMDS central lab) at 100 days post-ASCT
4. MRD-negative (proportion of malignant cells in the bone marrow is < 1 in 100,000, confirmed by HMDS central lab) after 12 cycles of isatuximab
5. Received ≥100mg/m^2 high-dose melphalan and ASCT
6. Eastern Cooperative Oncology Group (ECOG) performance status 0–2 (except in cases where ECOG >2 is due to effects of myeloma eg spinal cord compression)
7. Total bilirubin <= 1.5 x upper limit of normal (ULN)
8. Alanine aminotransferase (ALT) and/or aspartate aminotransferase (AST) ≤ 3 x ULN (if ALT and AST are tested, both must meet this criteria)
9. Adequate marrow function:
   - neutrophils ≥1.0 × 10^9/L (unless the participant has a known/suspected diagnosis of familial or racial neutropenia in which case an ANC ≥0.75 x 10^9/L is allowed),
   - Hb≥ 80g/L. Blood transfusions within 3 days prior to eligibility assessments are not permitted,
   - platelets ≥ 75 × 10^9/L. Platelet transfusions within 3 days prior to eligibility assessments are not permitted
10. Creatinine clearance (CrCl) ≥ 30 mL/minute, according to the Cockcroft-Gault formula, following correction of reversible causes (e.g. dehydration, hypercalcaemia, sepsis)
11. Agree to follow the pregnancy prevention guidelines:

    Female participants who:

    a) Are not of childbearing potential, OR
    b) If they are of childbearing potential, agree to practice 2 effective methods of contraception, at the same time, from the time of signing the informed consent form until 5 months after the last dose of study drug, OR
    c) Agree to practice true abstinence when this is in line with the preferred and usual lifestyle of the subject from the time of signing the informed consent form until 5 months after the last dose of study drug. (Periodic abstinence [e.g. calendar, ovulation, symptothermal, post-ovulation methods] and withdrawal are not acceptable methods of contraception.)
    d) Agree to not donate oocytes during the entire study treatment period and until 5 months after the last dose of study drug.

    Male participants, even if surgically sterilised (i.e. status post-vasectomy), must agree to one of the following:

    a) Agree to practice effective barrier contraception during the entire study treatment period and until 5 months after the last dose of study drug, OR
    b) Agree to practice true abstinence when this is in line with the preferred and usual lifestyle of the subject during the entire study treatment period and until 5 months after the last dose of study drug. (Periodic abstinence [e.g. calendar, ovulation, symptothermal, post-ovulation methods] and withdrawal are not acceptable methods of contraception).
    c) Agree to not donate sperm during the entire study treatment period and until 5 months after the last dose of study drug.
12. Signed the Informed Consent Document for the R1 treatment pathway.

Exclusion Criteria

1. Disease progression (according to IMWG criteria)
2. MRD-positive (proportion of malignant cells in the bone marrow is ≥ 1 in 100,000, confirmed by HMDS central lab) at 100 days post-ASCT or after 12 cycles of isatuximab
3. Registration exclusion criteria (cardiac risk assessment, HIV and Hepatitis B and C testing do not need to be repeated)

For peer review only - http://bmjopen.bmj.com/site/about/guidelines.xhtml

Table 4: Inclusion and Exclusion Criteria for Randomisation 2

Inclusion Criteria:

1. Standard-risk (participant is not **confirmed** to have at least two of these genetically adverse lesions: t(4;14), t(14;16), t(14;20), del(17p), gain(1q) as confirmed by CTRU.
2. 4 cycles of RCyBorD received
3. MRD-positive (proportion of malignant cells in the bone marrow is ≥ 1 in 100,000, confirmed by HMDS central lab) at 100 days post-ASCT
4. Received ≥100mg/m^2 high-dose melphalan and ASCT
5. Eastern Cooperative Oncology Group (ECOG) performance status 0–2 (except in cases where ECOG >2 is due to effects of myeloma eg spinal cord compression)
6. Total bilirubin <= 1.5 x upper limit of normal (ULN)
7. Alanine aminotransferase (ALT) and/or aspartate aminotransferase (AST) ≤ 3 x ULN (if ALT and AST are tested, both must meet this criteria)
8. Adequate marrow function:
   - neutrophils ≥1.0 × 10^9/L (unless the participant has a known/suspected diagnosis of familial or racial neutropenia in which case an ANC ≥0.75 x 10^9/L is allowed),
   - Hb≥ 80g/L
   - platelets ≥ 75 × 10^9/L. Platelet transfusions within 3 days prior to eligibility assessments are not permitted
9. Creatinine clearance (CrCl) ≥ 30 mL/minute, according to the Cockcroft-Gault formula, following correction of reversible causes (e.g. dehydration, hypercalcaemia, sepsis)
10. Agree to follow the pregnancy prevention guidelines:

    Female participants who:

    a) Are not of childbearing potential, OR
    b) If they are of childbearing potential, agree to practice 2 effective methods of contraception, at the same time, from the time of signing the informed consent form until 12 months after the last dose of cyclophosphamide or 5 months after the last dose of study drug, whichever is longest OR
    c) Agree to practice true abstinence when this is in line with the preferred and usual lifestyle of the subject, from the time of signing the informed consent form until 12 months after the last dose of cyclophosphamide or 5 months after the last dose of study drug, whichever is longest. (Periodic abstinence [e.g. calendar, ovulation, symptothermal, post-ovulation methods] and withdrawal are not acceptable methods of contraception.)
    d) Agree to not donate oocytes during the entire study treatment period and until 12 months after the last dose of cyclophosphamide or 5 months after the last dose of study drug, whichever is longest.

    Male participants, even if surgically sterilised (i.e. status post-vasectomy), must agree to one of the following:

    a) Agree to practice effective barrier contraception during the entire study treatment period and until 6 months after the last dose of cyclophosphamide or 5 months after the last dose of study drug, whichever is longest OR
    b) Agree to practice true abstinence when this is in line with the preferred and usual lifestyle of the subject during the entire study treatment period and until 6 months after the last dose of cyclophosphamide or 5 months after the last dose of study drug, whichever is longest. (Periodic abstinence [e.g. calendar, ovulation, symptothermal, post-ovulation methods] and withdrawal are not acceptable methods of contraception).
    c) Agree to not donate sperm during the entire study treatment period and until until 6 months after the last dose of cyclophosphamide or 5 months after the last dose of study drug, whichever is longest.
11. Signed the Informed Consent Document for the R2 treatment pathway.

Exclusion Criteria:

1. Disease progression (according to IMWG criteria)
2. Registration exclusion criteria (cardiac risk assessment, HIV and Hepatitis B and C testing do not need to be repeated)

For peer review only - http://bmjopen.bmj.com/site/about/guidelines.xhtml

Table 5: Inclusion and Exclusion criteria for Randomisation 3

Inclusion Criteria:

1. High-risk (participant is **confirmed** to have at least two of these genetically adverse lesions: t(4;14), t(14;16), t(14;20), del(17p), gain(1q)) as confirmed by CTRU.
2. 4 cycles of RCyBord received
3. Received ≥100mg/m^2 high-dose melphalan and ASCT
4. Eastern Cooperative Oncology Group (ECOG) performance status 0–2 (except in cases where ECOG >2 is due to effects of myeloma eg spinal cord compression)
5. Total bilirubin <= 1.5 x upper limit of normal (ULN)
6. Alanine aminotransferase (ALT) and/or aspartate aminotransferase (AST) ≤ 3 x ULN (if ALT and AST are tested, both must meet this criteria)
7. Adequate marrow function:
   - neutrophils ≥1.0 × 10^9/L (unless the participant has a known/suspected diagnosis of familial or racial neutropenia in which case an ANC ≥0.75 x 109/L is allowed),
   - Hb≥ 80g/L
   - platelets ≥ 75 × 10^9/L. Platelet transfusions within 3 days prior to eligibility assessments are not permitted
   - Creatinine clearance (CrCl) ≥ 30 mL/minute, according to the Cockcroft-Gault formula, following correction of reversible causes (e.g. dehydration, hypercalcaemia, sepsis)
8. Creatinine clearance (CrCl) ≥ 30 mL/minute, according to the Cockcroft-Gault formula, following correction of reversible causes (e.g. dehydration, hypercalcaemia, sepsis)
9. Agree to follow the pregnancy prevention guidelines:

   Female participants who:

   a) Are not of childbearing potential, OR
   b) If they are of childbearing potential, agree to practice 2 effective methods of contraception, at the same time, from the time of signing the informed consent form until 12 months after the last dose of cyclophosphamide or 5 months after the last dose of study drug, whichever is longest OR
   c) Agree to practice true abstinence when this is in line with the preferred and usual lifestyle of the subject from the time of signing the informed consent form until 12 months after the last dose of cyclophosphamide or 5 months after the last dose of study drug, whichever is longest. (Periodic abstinence [e.g. calendar, ovulation, symptothermal, post-ovulation methods] and withdrawal are not acceptable methods of contraception.)
   d) Agree to not donate oocytes during the entire study treatment period and until 12 months after the last dose of cyclophosphamide or 5 months after the last dose of study drug, whichever is longest.

   Male participants, even if surgically sterilised (i.e. status post-vasectomy), must agree to one of the following:

   a) Agree to practice effective barrier contraception during the entire study treatment period and until 6 months after the last dose of cyclophosphamide or 5 months after the last dose of study drug, whichever is longest OR
   b) Agree to practice true abstinence when this is in line with the preferred and usual lifestyle of the subject during the entire study treatment period and until 6 months after the last dose of cyclophosphamide or 5 months after the last dose of study drug, whichever is longest. (Periodic abstinence [e.g. calendar, ovulation, symptothermal, post-ovulation methods] and withdrawal are not acceptable methods of contraception).
   c) Agree to not donate sperm during the entire study treatment period and until 6 months after the last dose of cyclophosphamide or 5 months after the last dose of study drug, whichever is longest.
10. Signed the Informed Consent Document for the R3 treatment pathway.

Exclusion Criteria

1. Disease progression (according to IMWG criteria)
2. Registration exclusion criteria (cardiac risk assessment, HIV and Hepatitis B and C testing do not need to be repeated)

For peer review only - http://bmjopen.bmj.com/site/about/guidelines.xhtml

Interventions and Dosing

Intervention Schedule

All participants will receive four, 21 day, cycles of induction treatment with RCyBorD. During this time, the genetics risk status of the participant will be analysed and determined from bone marrow samples prior induction treatment (from diagnosis or trial-specific procedures). Participants will either be allocated as high risk (defined as the presence of at least 2 of these adverse lesions: t(4,14), t(14,16(, t(14,20), del(17p) or gain(1q)) or standard risk. At the end of the induction cycle participants will be assessed for response In the absence of disease progression or intolerance, participants with at least a partial response (PR) to will proceed to HDM and ASCT which will be carried out according to local practice. At 100 days post-ASCT participants will be assessed for MRD and response and will follow one of three pathways of post ASCT treatment, provided they meet the eligibility criteria:

1. Participants identified as standard risk and who are MRD-negative post-ASCT will be treated according to the R1 pathway. All participants will receive 12 cycles of Isa maintenance treatment. Those who remain MRD-negative following cycle 12, will be randomised to continue or stop Isa maintenance treatment.
2. Participants identified as standard-risk and who are MRD-positive post-ASCT and have at least a minimal response (MR) at 100 days post ASCT, will proceed to R2. Eligible participants will be randomised between: R maintenance; RBorD consolidation and R maintenance (RBorD + R); RISa maintenance; or RBorIsaD consolidation and RIsa maintenance (RBorIsaD + RIsa).
3. Participants identified as high-risk and have at least a partial response (PR) at 100 days post ASCT will proceed to R3. These participants will be randomised to receive either RBorD consolidation, followed by R maintenance; or RBorIsaD consolidation followed by RIsa maintenance.

All randomisations are balanced across the treatment arms. A computer generated minimisation programme that incorporates a random element will be used to ensure treatment groups are well balanced for the stratification factors of each randomisation (See Table 6).

Table 6: Stratification Factors

Randomisation:	Stratification factors:
1	• Centre<br>• Number of high risk lesions at trial registration (0 or 1 or unable to determine)
2	• Centre<br>• Number of high risk lesions at trial registration (0 or 1 or unable to determine)<br>• Response at 100 days post-ASCT ($< VGPR, \geq VGPR$)
3	• Centre<br>• Number of high risk lesions at trial registration (2, or ≥ 3 , or unable to determine)<br>• Response at 100 days post-ASCT ($< VGPR, \geq VGPR$)

For peer review only - http://bmjopen.bmj.com/site/about/guidelines.xhtml

Table 7 summarises the starting doses for the induction treatment and each post-ASCT treatment pathway. Following ASCT all treatments will continue until disease progression or intolerance. Response will be assessed at the end of each cycle according to the IMWG 2016 response criteria[24, 29-31]. Treatment intolerance will be assessed throughout each treatment cycle, according to the national cancer institute (NCI) common terminology criteria for AE (CTCAE) Version 5. All consolidation cycles are 21 days in length, maintenance cycles are 28 days in length.

Table 7: Dosing schedule for RCyBorD induction and each randomisation pathways

**Trial Registration- Induction (Each cycle = 21 days)**		
**Treatment (Route)**	**Induction – All participants (Starting Dose)**	**Days**
**Bortezomib (SC)**	1.3mg/m^2	1, 8, 15
**Cyclophosphamide (PO)**	500mg	1, 8
**Lenalidomide (PO)**	25mg	1-14
**Dexamethasone (PO)**	40mg	1, 8, 15
**Isatuximab Pre-Randomisation 1 (Each cycle = 28 day)**		
**Treatment (Route)**	**R1 pathway participants Only (Starting Dose)**	**Days**
**Isatuximab (IV)**	10mg/kg	1, 8, 15 and 22 cycle 1 then days 1 and 15 from cycle 2 onwards
**Randomisation 1: Isatuximab maintenance (Each Cycle = 28 days)**		
**Treatment**	**Isatuximab (Days)**	**"Stop Isatuximab" (Days)**
**Isatuximab (IV)**	10mg/kg (Day 1 Only)	None
**Randomisation 2: MRD-positive treatment escalation pathway**		
**Treatment Arm 1 (Control) : Lenalidomide (R) maintenance**		
**Treatment (Route)**	**Starting Dose (Days)**	
**Lenalidomide (PO)**	10mg/kg* (1-21)	
**Treatment Arm 2: RBorD consolidation + R Maintenance**		
**Treatment**	**Consolidation: Cycle 1-4 (each cycle = 21 days)**	**Maintenance (each Cycle = 28 days)**
**Bortezomib (SC)**	1.3mg/m^2 (1, 8, 15)	NA
**Lenalidomide (PO)**	15mg* (1-14)	10mg* (1-21)
**Dexamethasone (PO)**	20mg (1, 8, 15)	NA
**Treatment Arm 3:RISa Maintenance**		
**Treatment**	**Maintenance (Days)**	
**Lenalidomide (PO)**	10mg* (Day 1-21)	
**Isatuximab (IV)**	10mg/kg (1, 8, 15 and 22 cycle 1 then days 1 and 15 from cycle 2 onwards)	
**Treatment Arm 4: RBorIsaD + RIsa**		
Treatment	Consolidation Cycle 1-4: Each cycle = 21 days (Days)	Maintenance: Each cycle = 28 days (Days)
**Bortezomib (SC)**	1.3mg/m^2 (1, 8, 15)	NA
**Lenalidomide (PO)**	15mg* (1-14)	10mg* (1-21)
**Isatuximab (IV)**	10mg/kg (1, 8, 15 cycle 1 then days 1 and 8 cycle 2, then days 1 and 15 cycle 3 onwards)	10mg/kg (1 and 15)
**Dexamethasone (PO)**	20mg (1, 8, 15)	NA
**Randomisation 3: High Risk Treatment Pathway**		
**Treatment Arm 1: RBorD Consolidation and R maintenance**		
**Treatment**	**Consolidation: Cycle 1-4 (each cycle = 21 days)**	**Maintenance (each Cycle = 28 days)**
**Bortezomib (SC)**	1.3mg/m^2 (1, 8, 15)	NA
**Lenalidomide (PO)**	15mg* (1-14)	10mg* (1-21)

For peer review only

For peer review only - http://bmjopen.bmj.com/site/about/guidelines.xhtml

**Dexamethasone (PO)**	20mg (1, 8, 15)	NA
**Treatment Arm 2: RBorIsaD consolidation + RIsa Maintenance**		
**Treatment**	**Consolidation Cycle 1-4: Each cycle = 21 days (Days)**	**Maintenance: Each cycle = 28 days (Days)**
**Bortezomib (SC)**	1.3mg/m^2 (1, 8, 15)	NA
**Lenalidomide (PO)**	15mg* (1-14)	10mg* (1-21)
**Isatuximab (IV)**	10mg/kg (1, 8, 15 cycle 1 then days 1 and 8 cycle 2, then days 1 and 15 cycle 3 onwards)	10mg/kg (1 and 15)
**Dexamethasone (PO)**	20mg (1, 8, 15)	NA

*or final dose administrated at the end of induction or consolidation treatment if lower

Intervention Adherence

Throughout the trial R will be taken orally and swallowed whole at the same time on the scheduled days. D and Cy will be taken orally, whilst Bor will be administrated via a subcutaneous injection. Bortezomib may also be administered at home if this is in line with local policies and if appropriate procedures are in place. Isa will be given as an intravenous infusion and at a facility capable of managing hypersensitivity reactions.

To monitor adherence to the oral trial medicines, participants will complete a participant diary card which will be reviewed at each trial visit. Non-compliance will be reported to the CTRU and any unused capsules will be returned to the pharmacy.

Dose Modifications and Discontinuations

With the exception of Isa, which can only be interrupted and resumed, dose modifications are permitted in the management of toxicities. Upfront dose modifications in response to liver and/or renal impairment are also permitted within the protocol. When the dose of any drug is reduced the dose cannot be re-escalated in this trial.

If one drug in a combination is stopped, treatment with the other drugs in combination can continue. Scenarios in which the participant may remain on trial following consultation with the Chief Investigator (CI) are if they: discontinue two or more drugs during combination therapy; cease treatment for more than three weeks in between any treatment cycle or are delayed in starting maintenance of consolidation beyond 120 days post-ASCT. Note that if Bor, R or D is stopped during the pre-ASCT induction treatment due to toxicity, the participant will only be able to be treated on the standard-risk-MRD-negative pathway (assuming they meet the relevant inclusion criteria).

If treatment is discontinued early, the participant will be treated off trial at the discretion of their treating clinician.

Concomitant medication:

Local support care protocols, including anti-emetic schedules, tumour lysis syndrome prevention, venous thromboembolism prophylaxis and prophylactic antimicrobial therapy should be followed. The excluded concomitant medications and procedures whilst receiving trial treatment are listed in supplementary material.

For peer review only - http://bmjopen.bmj.com/site/about/guidelines.xhtml

Concomitant medication, disease and other malignancies will be recorded at eligibility.

Outcomes:

Primary outcomes

PFS-R1 is defined as the time from R1 to the time of first documented evidence of disease progression or death from any cause. Individuals who are lost to follow-up or progression-free at the time of analysis will be censored at their last known date to be alive and progression-free.

Attainment of MRD-negativity is defined as a binary endpoint. MRD-negativity will be determined at 6 months post-R2 (end of cycle 6 post-ASCT treatment for participants allocated to maintenance only strategies and end of cycle 7 post-ASCT treatment for participants allocated to maintenance and consolidation strategies) according to the IMWG 2016 response criteria.

The PFS rate is defined as the proportion of participants who are alive and progression-free 28 months post-R3.

Disease progression is defined according to the IMWG 2016 response criteria for Multiple Myeloma [24, 29-31].

Secondary outcomes:

The secondary outcomes of this trial are to assess; PFS for R2 and R3, Time to progression, Time to second PFS event (PFS2), Overall survival (OS), Survival after progression, Objective response rate (ORR), Attainment of Very Good Partial Response (≥ VGPR), Attainment of MRD negativity, Duration of MRD Negativity, Time to improved response, Time to next treatment, Treatment compliance and total amount of therapy delivered, Toxicity and Safety, including the incidence of second malignancies, Quality of life (QoL) including EORTC-QLQ-C30, EORTC-QLQ-MY20 and EQ-5D-3L and Cost effectiveness.

Exploratory and subgroup analyses

An overview of the planned exploratory and subgroup analysis can be found in the supplementary material. These include genetic and molecular analysis of participant samples conducted by the respective central laboratories, as well as additional analysis to review the infection and re-infection rates of covid-19 in participants during induction, post-ASCT and during post ASCT consolidation.

Participant's timelines

The full trial schema can be seen in Figure 1. The schedule of assessments at each time point are presented in Figure 2.

Trial Entry

Participants will enter into the trial at one of two points in their pathway, this will either be at bone marrow registration or at main trial registration.

For peer review only - http://bmjopen.bmj.com/site/about/guidelines.xhtml

Trial Consent

Participants who enter the trial at bone marrow registration will provide consent to have bone marrow samples taken and sent to central laboratories for analysis. If the participant does not have myeloma or decides to not take part in RADAR, they will have the option of consenting to their samples being used in future research.

All participants will be required to provide written informed consent for the trial prior to trial registration (for initial trial treatment and further trial information) and prior to starting their post-ASCT treatment pathway once their post-ASCT assessments have been completed (for their pathway specific treatment). Optional consent regarding QoL and healthcare resource use questionnaires and the use of samples for future research will also be obtained within the first consent process.

Consent forms are included in supplementary material.

Trial Registration

Following initial consent, participant eligibility for trial registration will be assessed. Trial samples for blood and urine will be taken for all participants and bone marrow samples will be taken for those who did not enter the trial through bone marrow registration.

Genetic Risk

Bone marrow samples from diagnosis will be analysed at time of diagnosis at local cytogenetics laboratories for FISH-defined high-risk makers. Original/copy of the FISH testing report will also be sent to CTRU where they will be reviewed by the CI/Trial management team for risk stratification. Participants will either be categorised high-risk (defined as the presence of at least 2 of the following adverse lesions: t(4;14), t(4;16), t(14:20), del(17p) or gain(1q)) or standard-risk.

Participants will commence RCyBorD treatment once a diagnosis of symptomatic myeloma has been confirmed by the treating physician, and ideally when it is known that the FISH testing has been successful.

Where risk status can be determined from the first sample participants can continue on the trial. If status cannot be confirmed and the FISH report shows a full test failure i.e. no information relating to adverse lesions is available, a second attempt will be made to collect a bone marrow sample prior to treatment starting. If partial results are obtained from the FISH sample, it may be possible that the CI/ trial management group (TMG) will be able to determine risk status using the assumption that translocations are mutually exclusive and that some markers are rare. If risk status can be determined a second bone marrow sample is not required. However, this must be confirmed by the CTRU.

If, after two bone marrow samples, the FISH results for some or all the adverse lesions are inconclusive participants risk will be determined using a combination of the results from the two samples. The most appropriate pathway will be determined by the CI. Note this may result in the participants stratification factor being 'unable to determine' at randomisation. If a second bone marrow has been requested by CTRU but the collection was not attempted, the participants will be taken off the study (after ASCT, if induction treatment was already started)

For peer review only - http://bmjopen.bmj.com/site/about/guidelines.xhtml

Induction and ASCT

Following trial registration, once all central samples have been taken and risk status confirmed, participants will receive induction treatment as described in the intervention section. In the absence of disease progression or treatment intolerance participants with at least a partial response (PR) to will proceed to HDM and ASCT.

Consenting participants will complete QoL and resource use questionnaires at the end of RCyBorD induction treatment.

All participants who go through ASCT will have a bone marrow sample taken at 100 days (+/- 14) post-ASCT. If an MRD result cannot be obtained at the first attempt, the bone marrow will be repeated once (within 28 days after 100 days post-ASCT time point). If the second attempt fails, the participant will be considered MRD-positive.

Post-ASCT Treatment

Participants who receive all 4 cycles of induction treatment followed by ASCT will be assessed for eligibility into their assigned randomisation pathways determined by genetic risk and MRD results and treated as described in the intervention schedule.

Consenting participants in the R1 pathway will complete QoL and resource use questionnaires after cycle 6, 12, 24 and 36 (participants randomised to “stop Isa” will complete the cycle 24 and 36 QoL and resource use questionnaires at equivalent time points).

Similarly, consenting participants to receive R or RIsa within pathway R2 and R3 will complete QoL and resource use questionnaires after cycles 6, 12 and 24 of maintenance. Those randomised to receive RBorIsa+R or RBorIsaD+RIsa will complete QoL and resource use questionnaires after cycles 3, 9 and 21 of maintenance.

Post-ASCT treatment (including observation only in the R1 pathway) is continued until disease progression, participant or clinician withdrawal or death. After treatment discontinuation, the participant is treated off trial by their local clinician and followed-up as per the trial follow-up schedule.

Trial Follow-up

Participants who discontinue during induction and until 100 days post-ASCT will be followed up for data pertaining to safety, details of salvage treatment, progression (including second progression), and survival at standard care visits. Follow ups will be conducted every 2 months up until disease progression, death or the end of trial. Similar follow-up will be completed for participants who discontinue their allocated trial treatment, which started post-ASCT. In addition consenting participants will still complete QoL and resource use questionnaires at appropriate time points.

All types of adverse event/reaction will be collected until 60 days after the last dose of protocol treatment.

For peer review only - http://bmjopen.bmj.com/site/about/guidelines.xhtml

Following disease progression all participants will be followed up annually until death, or until the end of the trial.

Sample size

In total 1400 participants will be recruited into the trial. Accounting for participants who will be lost to follow-up, this allows for 500, 450 and 172 participants to enter each randomisation, respectively.

Randomisation 1

Data from our previous trial Myeloma XI, suggests that approximately 50 participants will lose their MRD-negative status during the first 12 cycles of Isa maintenance. In addition, Myeloma XI estimated median PFS with continuous R to be 50 months amongst TE participants.[32] Under these assumptions and allowing for our randomisation being 12 months later, 500 participants allows for 80% power to test a non-inferiority margin of 10% at 2 years post-R1 (this equates to a hazard ratio of 1.38) with a one-sided 5% significance level, allowing for approximately 20% further drop-out after R1 amongst participants unwilling to cease treatment or being lost to follow-up.

A power of 80% will be attained when 238 PFS events have been observed. These calculations assume that PFS follow an exponential distribution and that there will be 3 years of recruitment to R1 and 2 further years of follow-up (in order that all participants can have PFS at 2 years post-R1 assessed).

Global Health Status/Quality of Life is considered a key secondary endpoint of R1 as the conclusion that the cessation of continuous treatment is non-inferior should not come at the expense of quality of life. Ignoring those required to account for participants lost to follow-up, the 406 participants recruited above give >95% power to detect a difference of 10 points at 2 years post-R1, where 10 points represents an accepted medium magnitude clinically relevant difference.[33] The calculation uses data collected from the intensive pathway of MRC Myeloma IX to estimate that the standard deviation in the Global Health Status/QoL subscale of EORTC-QLQ-C30 as 27.54 along with a two-sided 5% significance level.[34]

Randomisation 2

R2 is a multi-arm, multi-stage drug/drug-combination assessment platform with seamless phase II (activity) and phase III (efficacy) stages. Each experimental arm is compared with a designated control arm (R maintenance).

Early data from Myeloma XI and similar data from IFM/DFCI 2009 [35] suggest that a 10% upgrade in MRD-negative rate after 6 months continuous R treatment is achievable. It is thought that RBorD consolidation followed by attenuated continuous R or continuous R combined with antibody or immunotherapy could lead to a 30% increase in MRD-negative rate, a 20% upgrade of increase at 6 months post-randomisation when compared to continuous R treatment, as good as therapy combinations containing an alternative proteasome inhibitor.[33]

Following the strategy of Bratton and colleagues [36] for a multi-arm multi-stage trial with:

- N=2 stages (Stage 1 (activity) and Stage 2 (efficacy)) and

For peer review only - http://bmjopen.bmj.com/site/about/guidelines.xhtml

- J=4 experimental arms (RBorD+R, R+Isa, RBorIsaD + RIsa and TBC) and a single control arm (R)
- Where allocation is equal across each arms (1:1:1:1:1) and
- The power in Stage 1 and Stage 2 is 95% and 85%, respectively,
- the one-sided significance level in Stage 1 and Stage 2 is 25% and 1%, respectively,
- 10 participants per month are entered at R2, Stage 1 (activity) analysis and reporting requires 1 month and 5% of participants drop-out.

This equates to 40 participants in each arm at Stage I and 50 further participants in each arm at Stage 2. This will require a maximum of 46 months of recruitment plus a further 6 months of follow-up for the final participant. Overall, this design has a power of 83.4% and a one-sided family-wise error rate (FWER) of ~3%.

Randomisation 2 has been powered assuming four experimental arms and that all experimental arms will continue to stage 2. In the case that only three experimental arms are implemented or at least one experimental arm is concluded to show a lack of activity after stage 1, the trial will have a higher power than stated above.

Randomisation 3

Each arm of R3 will take the form of one-stage treatment design proposed by Simon, Wittes and Ellenberg [37] where the A'Hern design [38] is first implemented to determine treatment activity in both arms. Treatment activity will be determined by considering the PFS rate at 28 months. Data from Myeloma XI demonstrated a median PFS for high-risk participants in the intensive pathway of approximately 19 months (unpublished data). It is thought that for the treatments proposed to be investigated further in a Phase III trial they should increase the median PFS from 19 months to at least 28 months (9 month increase), i.e. increasing the PFS rate at 28 months from 34.7% to 50%.

In order to have an 80% power of demonstrating that the one-sided 95% confidence interval of the PFS rate at 28 months excludes 34.7%, 81 evaluable participants are required, where a treatment arm will be deemed a "success" if at least 37 out of 81 participants are progression-free at 28 months. To account for approximately 5% of participants being unevaluable at 28 months post-R3, 86 participants will be randomised to each treatment arm i.e. 172 participants are required to be entered into R3.

Total Study Sample Size

Accounting for those participants who will be lost-to-follow-up R1, R2 and R3 require 500, 450, and 172 participants to enter each randomisation respectively.

Considering R1 and R2, it is assumed that 50 participants will lose their MRD-negative status during the 12 months of continuous treatment (Myeloma XI, unpublished data). Therefore 550 participants are required to enter the MRD-negative part of the standard-risk pathway. It is assumed that 55% of standard-risk participants will be MRD-negative at the point of assessment therefore approximately 1000 participants are required to reach the MRD assessment stage of the standard-risk pathway [35, 39] It is assumed that 90% of all those recruited will reach this point therefore 1112 participants are required to enter the standard-risk pathway[5] . It is anticipated that 80% of all those recruited will be standard-risk (Myeloma XI, unpublished data) therefore at least 1390 participants are required to be recruited to the trial. Rounding this we obtain an overall sample size of 1400.

For peer review only

This gives 280 participants who are expected to enter the high-risk pathway, using the same logic as in the standard-risk pathway if 90% of participants reach R3 then there will be approximately 252 participants recruited to R3. Given the calculations above it is plausible that another arm could be added to the high-risk pathway at a later date.

Recruitment:

It is planned that 1400 participants will be recruited for a total of 36 months. Once all centres are open, the recruitment target is 38 participants a month (equating to approximately 30 standard risk and 8 high-risk participants).

In order to ensure the trial will meet the target sample size within the recruitment period, site set-up was prioritised whilst the trial was preparing to open to recruitment. In addition, the trial team are actively engaging with Principal Investigators at sites who support the trial to ensure that those sites open quickly and are maintaining regular communication with open sites to ensure that they continue to recruit to the trial.

The trial opened to recruitment on 11/05/2021.

Assignment of treatment allocations:

Each of the registration and randomisation procedures will be conducted centrally using the University of Leeds Clinical Trial Research Unit (CTRU) automated 24-hour web base and telephone system.

Registration

If a patient is suspected to have myeloma they will enter the trial at the time of routine diagnostic tests, through bone marrow registration. Once diagnosis is confirmed locally and the research team consider the patient potentially eligible for the trial, patients will be provided with full verbal explanation of the trial and full participant information sheet and informed consent documents to consider. Once the patient has provided informed consent to the first stage of the trial they will be registered into the trial at Bone Marrow Registration.

Patients who have already had Myeloma confirmed prior to being approached for the trial, and those who decline consent at bone marrow registration, will enter at main trial registration.

Data Collection

Data collection will primarily be via Remote Data Entry (RDE) on electronic case report forms (eCRFs), provided by the CTRU at the University of Leeds, and will be entered by staff at the research site. Access to the live RADAR database will be provided by the CTRU following sites being authorised to open to recruitment; guidance on RDE and completing eCRFs will be provided. Data regarding informed consent, QoL, Healthcare Resource Use questionnaires and in-patient stay forms will be collected via paper case report forms.

For peer review only - http://bmjopen.bmj.com/site/about/guidelines.xhtml

QoL and Healthcare Resource Use questionnaires will be completed by the participant in clinic or at home and sealed in an envelope before handing back to the site research team who will return them to the CTRU via post.

Data Management

All information collected during the course of the trial will be kept strictly confidential. Information will be held securely on paper and electronically at the CTRU. The CTRU will comply with all aspects of the Data Protection Act 2018.

Data will be monitored for quality and completeness by the CTRU. Missing data will be chased until it is received, confirmed as not available or the trial is at analysis. The CTRU/Sponsor will reserve the right to intermittently conduct source data verification exercises on a sample of participants, which will be carried out by staff from the CTRU/Sponsor. Source data verification will involve direct access to participant notes at the participating hospital sites and the ongoing central collection of copies of Informed Consent Documents and other relevant investigation reports.

Statistical methods

Statistical analysis is the responsibility of the CTRU statisticians, with the exception of the cost-effectiveness analysis, which will be undertaken by the health economists at the University of Leeds. A full statistical analysis plan (SAP) and Health Economics Analysis Plan (HEAP) will be written and approved before any analyses are undertaken.

The analysis population will depend on the randomisation. Separate intention to treat (ITT), per protocol (PP) and safety populations will be defined for each randomisation. In either case the ITT population will include participants according to their randomisation allocation, regardless of eligibility whether they prematurely discontinued the treatment, or did not comply with the regimen. The PP population will exclude individuals who do not receive their randomisation allocation or who are found to be ineligible following randomisation. The safety population will consist of all participants who receive at least one dose of the trial treatments and analyse participants according to what they received rather than what they were randomised to.

With the exception of two interim analyses, no other formal analysis is planned until participants have attained the primary endpoint for each of the randomisations.

Primary endpoint analyses

Randomisation 1

The primary endpoint for R1 will be attained 2 years after the final participant has been randomised to R1.

As R1 is a non-inferiority trial, both PP and ITT population analyses will be used as co-primary analyses and both are required to demonstrate statistical significance of non-inferiority where any discrepancies will be reported.

For peer review only - http://bmjopen.bmj.com/site/about/guidelines.xhtml

PFS at R1 will be assessed using Cox proportional hazards regression to adjust for the stratification factors of R1 excluding centre, and presented using Kaplan-Meier estimates of the survival function. The treatment cessation strategy will be declared non-inferior to the continuous treatment strategy if the upper bound of the one-sided 95% confidence interval for the hazard ratio is less than 1.38. Note no adjustment will be made for interim analysis due to it being a test for inferiority (see below) and not non-inferiority.

<u>Randomisation 2</u>

The primary endpoint for R2 will be attained 6 months after the final participant has been randomised to R2. Primary analysis will be undertaken on the ITT population.

An overall one-sided approximately 3% FWER will be applied accounting for each pair-wise comparison over each stage and all comparisons. This equates to a 25% and 1% one sided significance level at interim and final analysis. MRD-negative rate will be compared between each pair-wise comparison (experimental vs control) in turn using logistic regression and adjusted for the minimisation factors of R2 excluding centre.

<u>Randomisation 3</u>

The primary endpoint for R3 will be attained 28 months after the final participant has been randomised to R3.

Primary analysis will be undertaken on the ITT population. The number of participants who are alive and progression-free at 28 months post R3 will be summarised separately within each treatment arm and exact one sided 95% confidence intervals calculated. The null hypothesis is that the percentage of participants alive and progression-free at 28 months post R3 is 34.7%. A treatment arm is deemed a "success" if the one sided 95% confidence intervals of the PFS rate at 28 months excludes 34.71%. The treatment will then be proposed to be investigated further in a phase III trial.

No multiple imputation is planned for the primary endpoints.

Secondary endpoint analyses

Overall survival and other time-to-event endpoints will be analysed using methods similar to those described for progression-free survival. All binary endpoints will be analysed using similar methods to those described for the attainment of MRD negativity endpoint.

For both R1 and R3, each experimental arm will be independently compared to historical control data using molecularly matched individual participant data (IPD) from Myeloma XI+(TE) and assessed for superiority in terms of progression-free survival. Further details of the molecular matching of IPD will be described in the statistical analysis plan, but it is anticipated that propensity score matching may be an appropriate technique. In each case a one-sided 10% significance level will be applied for each comparison with historical data.

The number and proportion of participants in each response category will be summarised by allocated treatment and exact 95% two-sided confidence intervals will be calculated. The difference in

For peer review only - http://bmjopen.bmj.com/site/about/guidelines.xhtml

proportions for each response category will be presented with corresponding 95% two-sided confidence intervals.

Quality of life will be summarised using mean scores adjusted for baseline and two-sided 95% CI for each EORTC QLQ-C30 and EORTC QLQ-MY20 module symptom, role and functioning domain at each assessment time-point. Similar summaries will be produced for quality-adjusted life years (QALYs) as scored by the EQ-5D 3 Level (EQ-5D-3L) questionnaire. For the key secondary analysis of R1, the Global Health Status / Quality of Life subscale at two years post-R1, will be compared between the arms using a multivariable linear regression model, adjusting for the R1 stratification factors.

Exploratory and subgroup analyses

A series of subgroup and exploratory analyses will be undertaken. Subgroup analyses will follow the same structure as the main analysis of the respective endpoint. Where appropriate interaction terms will be added to the regression models to account for the subgroup being investigated. Subgroup and exploratory analyses may, by chance, generate false negative or positive results. Those carried out will be interpreted with caution.

The analysis for the exploratory outcomes will be detailed in the SAP.

Health economics

The economic evaluation will take a decision modelling approach to assess the cost-effectiveness of treatment options for risk stratified treatment vs standard non-stratified treatment over participants' lifetime. This will allow several research questions to be incorporated within one model. The model will be developed using best practice[40] and the model structure, health states and parameter values will be derived from the trial outputs, published literature, and expert clinical opinion. Data to model stratified treatment options will be drawn from the RADAR trial results while parameters for standard non-stratified treatment will be obtained from the wider literature.

Reflecting the main trial analysis of effectiveness, the economic evaluation will assess the cost-effectiveness of treatment options at each of the randomisations in RADAR. For standard risk participants at R1, the cost-effectiveness analysis will compare treatment with isatuximab for 12 cycles only to long-term isatuximab treatment, continued until disease progression. For standard risk participants who are MRD-positive at 100 days post-ASCT in terms of conversion to MRD negativity at R2, the cost-effectiveness of post-ASCT consolidation and maintenance treatments in the treatment of standard-risk participants will be evaluated. For high-risk participants at R3, the cost-effectiveness of post-ASCT consolidation and maintenance treatment strategies containing lenalidomide, bortezomib, dexamethasone and isatuximab separately will be evaluated. In addition, the cost-effectiveness of stratified treatment pathways will be compared with standard non-stratified treatments.

All analyses will be conducted following NICE reference case guidelines[41]. As such, outcomes will be measured in QALYs based on EQ-5D [42] and EORTC QLQ-C30 [43] utilities and costs will be calculated from a health and personal social services perspective. Cost-effectiveness will be determined in comparison to the NICE cost-effectiveness threshold of £20,000 per QALY gained. Deterministic and probabilistic (using Monte Carlo simulations) sensitivity analyses will be conducted to explore the

For peer review only - http://bmjopen.bmj.com/site/about/guidelines.xhtml

impact of parameter value changes on estimates of cost-effectiveness and to determine the level of uncertainty around the results of the lifetime analysis. In addition, value of information analysis will be conducted to estimate the value of further research.

Trial Oversight

The TMG includes the CI, CTRU team and co-investigators that are assigned responsibility for the clinical set up, ongoing management, promotion of the trial and interpretation of the results. The trial steering committee (TSC), consisting of independent clinicians and statisticians, will provide overall supervision on the trial, including trial progress, adherence to protocol, participant safety and consideration of new information.

Data Monitoring

An independent data monitoring and ethic committee (DMEC) will review the safety and ethics of the trial. Detailed reports will be prepared by the CTRU for the DMEC meetings which will take place at approximately yearly intervals. Additional reports will also be provided at intervals between these meetings. The committee will also review cumulative safety data by arm on an ongoing basis along with individual SAE/SAR listings. After each annual review, the DMEC will make their recommendations to the TSC about the continuation of the trial.

Interim Analyses

Full interim reports will be presented to the DMEC in confidence annually and following the planned interim analyses which are expected to take place when half the number of events (119) have been observed in the treatment de-escalation pathway (R1) and at the end of the activity stage 1 treatment escalation pathway (R2), approximately 6 months after the 40th participant has been randomised.

The interim analysis of R1 will be a one-sided test for inferiority conducted at the 5% significance level. PFS at R1 will be assessed using Cox proportional hazards regression to adjust for the minimisation factors (excluding centre), and presented using Kaplan-Meier estimates of the survival function.

The interim analysis for R2 will be conducted at the end of Stage I (the phase II part) of the randomisation which is due 6 months following the randomisation of the 40th participant. This analysis will compare each experimental arm with the control arm in separate tests of superiority using a one-sided 25% significance level and 95% power to determine whether any of the experimental arms show lack of activity i.e. the null hypothesis in the superiority test is not rejected. Should an arm show lack of activity it will not be continued to the second stage of the randomisation (the phase III part). To account for the interim analysis and to give an overall significance level of 1% and power of 80% for each individual comparison the final analysis will be conducted using a one-sided 1% significance level and 85% power. Recruitment will continue seamlessly between activity and efficacy in these scenarios. Formal powered pair-wise comparisons are not undertaken comparing experimental arms at either stage.

The DMEC committee members, in light of the interim analysis, will make their recommendations to the TSC about the continuation of the different aspects of the trial.

For peer review only - http://bmjopen.bmj.com/site/about/guidelines.xhtml

Harms

Adverse events

Adverse events are any untoward medical occurrence in a participant or clinical trial subject administered a medicinal product and which does not necessarily have a causal relationship with this treatment. AEs can be defined as any unintentional, unfavourable clinical signs or symptoms; any new illness or disease, or the deterioration of existing disease or illness; or clinically relevant deterioration in any laboratory assessments or clinical test. Due to the nature of multiple myeloma and its treatment, participants are likely to experience several adverse events throughout the course of the disease.

All AEs, both related and unrelated to myeloma treatment will be collected on the relevant eCRF from trial registration until 60 days after the last dose of protocol treatment and will be evaluated in accordance with the National Cancer Institute Common Terminology Criteria for Adverse Events 5.0 (NCI-CTCAE). AEs and ARs should continue to be collected for participants who are randomised to 'stop isatuximab' at R1 (and until 60 days after last on-trial assessment), even though they will not be receiving any trial drugs during this period.

Adverse Events of special interest

An AE of special interest (serious or non-serious) is one of scientific and medical concern specific to isatuximab, for which ongoing monitoring and rapid communication by the investigator to the sponsor is considered to be appropriate. Isatuximab AEs of special interest include; ≥Grade 3 infusion reactions (IRs), Pregnancies, Symptomatic overdoses with IMP, and Second Primary Malignancies (SPMs).

Serious adverse events

Serious Adverse Events (SAEs) are defined as any untoward medical occurrence or effect that at any dose results in death; or are life threatening (at the time of the event); or require in-patient hospitalisation or prolongation of existing hospitalisation; or result in persistent or significant disability or incapacity; or result in a congenital anomaly or birth defect; or any other important medical event. All SAEs will be reported from registration until 60 days post the last dose of the trial drug. SAEs should continue to be collected for participants who are randomised to 'stop isatuximab' at R1 (and until 60 days after last on-study assessment), even though they will not be receiving any study drugs during this period.

Serious Adverse Reactions (SARs) are SAEs that are deemed possibly related to any dose administered of any trial treatment. Suspected unexpected serious adverse events (SUSARs) are SARs which are not listed in the reference safety information for that medicinal product. SARs and SUSARs will be reported from the date of first trial dose and for the duration of the trial.

Secondary malignancies

All new primary malignancies (SPMs) or suspected malignancies occurring from the date of trial registration and for the duration of the trial will be recorded. SPMs will be summarised and reviewed by an appointed member of the TMG who will determine whether trial treatment should continue.

For peer review only - http://bmjopen.bmj.com/site/about/guidelines.xhtml

Pregnancies

Participants registered into RADAR agree to follow the Celgene approved Pregnancy Prevention Programme. Pregnancies and suspected pregnancies (including a positive pregnancy test regardless of age or disease status) in a female participant must be reported throughout the study and for 12 months after the last dose of cyclophosphamide or for 5 months following cessation of protocol treatment, whichever is longest. Pregnancies and suspected pregnancies (including a positive pregnancy test regardless of age or disease status) in a male participant's partner must be reported throughout the study and for 6 months after the last dose of cyclophosphamide or for 5 months following cessation of protocol treatment, whichever is longest.

Safety Analyses

Safety analyses will summarise all SUSARs, SARs, SAEs, AEs and ARs, respective to where they occurred within the trial pathway (Induction, ASCT, Pre-R1, Post-R1, Post-R2, Post-R3). Safety data will be presented by treatment group for the safety population in addition to relationship to trial treatment.

Auditing

The CTRU and the trial sponsor have procedures in place to ensure that serious breaches of Good Clinical Practice (GCP) or the trial protocol are picked up and reported. A triggered monitoring plan will ensure that sites at risk are monitored accordingly.

Patient and Public Involvement

RADAR has been developed following extensive discussion with the UK myeloma community. Patients were first involved into the research at the grant application stage, and were incorporated into the development of the primary endpoint of R1, in which they confirmed a research question involving the stopping of treatment given there was no detectable myeloma would be of interest. The trial consent and participant information documents were reviewed for clarity by a patient representative. To ensure that a patient perspective is considered throughout the trial a PPI advisory group, including 4-8 members will feed into TMG meetings, and plan to host quarterly meetings to discuss urgent matters. It is envisioned that the PPI group, may help with writing summaries of results that can be published on patient forums and websites and maybe even shared directly with patients.

Ethics and Dissemination

Research and ethics approval

Ethics approval has been obtained from the London – Central Research Ethics Committee (20/LO/0238). In addition capacity and capability has been confirmed by the appropriate research and development department for each participating centre prior to opening to participant recruitment into the trial. This included but was not limited to; having access to the non-trial supplied IMP (Bor, Cy and D), being able to conduct FISH as SoC, capacity of staff, ability to complete all protocol required assessments/investigations, and ability to archive trials documents.

Participants will be required to provide informed consent before joining the trial.

For peer review only - http://bmjopen.bmj.com/site/about/guidelines.xhtml

Protocol Amendments

The trial opened to recruitment on 11/05/2021 using protocol version 2, dated 29th April 2020. This publication is written based from protocol version 3 dated July 2021. An amendment to protocol version 3 is anticipated Mid-2022, which should include the following:

High-Risk Pathway

The cytogenetic abnormality del(1p) will be added to the definition of high-risk. In addition, Isa will be added to induction regimen for high-risk patients following their first cycle of RCyBorD (whilst their risk status is confirmed). Furthermore, R3 will be removed and high-risk patients will receive RBorIsaD + RIsa only post-ASCT. The pathway will follow a single-arm, single stage, phase II, three outcome design, with the new primary objective to assess the activity of RCyBorD+Isa induction followed by ASCT and RBorD+Isa consolidation and RIsa maintenance in terms of PFS at 18 months post trial registration.

Other changes

- The removal of the phone registration and randomisation;
- The clarification of allowing prescribed medication to remove risks of thromboembolism before preceding onto treatment in the exclusion criteria for registration (and for starting post ASCT treatment)
- The allowance of blood support products, blood transfusions and platelet transfusions as per institutional guidelines at eligibility and pre-treatment assessments
- An update of exclusion criteria to allow for previous plasmacytomas.

Consent or assent

The PI retains overall responsibility for the informed consent of participants at their site and must ensure that any medically qualified person delegated responsibility to participate in the informed consent process is duly authorised, trained and competent to participate according to the ethically approved protocol, principles of GCP and Declaration of Helsinki. Written consent will be obtained and signed by a medically qualified member of the site research team. Informed consent must be obtained, and the participant must be registered into the trial prior to the participant undergoing procedures that are specifically for the purposes of the trial and are out-with standard routine care at the participating site. This include the confirmation of optional consent (QoL/ Healthcare resource use questionnaires and decision regarding consent for samples to be used in future research). At any stage, participants can withdraw consent without repercussion.

Confidentiality

All information collected during the course of the trial will be kept strictly confidential. Information will be held securely on paper and electronically at the Clinical Trials Research Unit (CTRU). The CTRU will comply with all aspects of the Data Protection Act 2018.

For peer review only - http://bmjopen.bmj.com/site/about/guidelines.xhtml

Access to data

Individual participant data (with any relevant supporting material, e.g. data dictionary, protocol, statistical analysis plan) for all trial participants (excluding any trial-specific participant opt-outs) will be made available for secondary research purposes at the end of the trial, i.e. usually when all primary and secondary endpoints have been met and all key analyses are complete.

Data will be shared according to a controlled access approach, based on the following principles:

- The value of the proposal will be considered in terms of the strategic priorities of the CTRU, Chief Investigator and Sponsor, the scientific value of the proposed project, and the resources necessary and available to satisfy any data release request.
- We encourage a collaborative approach to data sharing, and believe it is best practice for researchers who generated datasets to be involved in subsequent uses of those datasets.
- The timing and nature of any data release must not adversely interfere with the integrity of the trial or research project objectives, including any associated secondary and exploratory research objectives detailed in the ethically approved original research protocol. On an individual trial or research project basis, a reasonable period of exclusivity will be agreed with the trial or research project team.
- Any data release must be lawful, in line with participants' rights and must not compromise patient confidentiality. Where the purposes of the project can be achieved by using anonymised or aggregate data this will always be used. We will release individual patient data only in a form adjusted so that recipients of the data cannot identify individual participants by any reasonably likely means. We will also only share data when there is a binding agreement in place stating that data recipients will not attempt to re-identify any individual participants.
- Any data release must be in line with any contractual obligations to which the CTRU is subject.
- The research must be carried out by a bone fide researcher with the necessary skills and resources to conduct the research project.
- The research project must have clear objectives and use appropriate research methods.
- The research must be carried out on behalf of a reputable organisation that can demonstrate appropriate IT security standards to ensure the data is protected and to minimise the risk of unauthorised disclosure.

Data will only be shared for participants who have given consent to use of their data for secondary research.

Requests to access trial data should be made to CTRU-DataAccess@leeds.ac.uk in the first instance. Requests will be reviewed (based on the above principles) by relevant stakeholders. No data will be released before an appropriate agreement is in place setting out the conditions of release. The agreement will govern data retention requirements, which will usually stipulate that data recipients must delete their copy of the data at the end of the planned project.

Ancillary and post-trial agreements

Participants who stop trial treatment due to progression or any point prior to the end of trial will be treated off-trial at the discretion of their treating clinician. Those who stop prior to disease progression will be followed up 2 monthly until disease progression, death or the end of the trial. Following disease progression all participants will be followed up annually until death, or until the end of the trial for post progression endpoints.

Participants in the R1 pathway who are MRD-positive at 12 months post 12 cycles of isatuximab and therefore aren't eligible for randomisation 1 will continue to receive isatuximab maintenance, these

For peer review only - http://bmjopen.bmj.com/site/about/guidelines.xhtml

participants would continue to be monitored, and data collected, as previously but trial samples would not be collected.

Dissemination policy

Authorship will be in keeping with the UK-MRA publication policy and due acknowledgement to participants, local investigators, funders and NCRI Haematological Oncology Study Group support made. The success of the trial depends upon the collaboration of all participants. For this reason, credit for the main results will be given to all those who have collaborated in the trial, through authorship and contributor-ship. Uniform requirements for authorship for manuscripts submitted to medical journals will guide authorship decisions alongside the guidance of the UK Myeloma Research Alliance Authorship Policy.

To maintain the scientific integrity of the trial, data will not be released prior to the end of the trial or a primary endpoint being reached, either for trial publication or oral presentation purposes, without the permission of the Trial Steering Committee and the (Co-)Chief Investigators. In addition, individual collaborators must not publish data concerning their participants that is directly relevant to the questions posed in the trial until the main results of the trial have been published and following written consent from the Sponsor.

Appendices

Informed consent material

The consent forms which are to be completed by the participant at Bone Marrow Registration and/or Trial Registration and prior to each randomisation pathways are included in supplementary material.

Biological specimens

The collection of central samples for laboratory analysis is summarised in Figures 3 and 4. The analysis to be conducted for trial purposes is stated in the supplementary material. Additional analysis may be carried out by each central laboratory provided the appropriate consent for sample use in future research has been provided by the participant at trial entry.

Author Contributions

The RADAR trial was conceived by KY and MC and designed by them in collaboration with DAC. All authors (KLR, ABC, KR, DAC, AH, SQ, MD, MK, RO, HWA, GC, DM, CO, LB, RL, AP, BD, MC, GP, RP, GJ, CB, JS, RT, AC, CP, MC, SA, and KY) inputted into the development of the protocol and participant information sheet. The first draft of the protocol paper was written by KLR and ABC. All authors (KLR, ABC, KR, DAC, AH, SQ, MD, MK, RO, HWA, GC, DM, CO, LB, RL, AP, BD, MC, GP, RP, GJ, CB, JS, RT, AC, CP, MC, SA, and KY) reviewed and approved the final manuscript.

Funding

This trial is funded by Cancer Research UK (C9203/A24078), Sanofi Genzyme and Celgene: A BMS Company. The funders had no role in the design, collection, analysis or collection of data; in writing

For peer review only - http://bmjopen.bmj.com/site/about/guidelines.xhtml

the manuscript; or in the decision to submit the manuscript for publication. This work was also supported by Core Clinical Trials Unit Infrastructure from Cancer Research UK (C7852/A25447).

The trial sponsor is responsible for the overall conduct of the trial as defined by Directive 2001/20/EC is University of Leeds, UoL/LTHT Joint Sponsor QA office (CTIMPs); Research & Innovation Centre/Faculty of Medicine & Health; Leeds Teaching Hospitals NHS Trust/University of Leeds; St James University Hospital; Leeds LS9 7TF.

Competing Interest Statement

ABC, KLR, DAC, AH, CO, LBai, LBar, AP, RL report grants and non-financial support from BMS/Celgene, grants and non-financial support from Merck Sharpe & Dohme, grants and non-financial support from Amgen, grants and non-financial support from Takeda, during the conduct of the trial. DAC also reports travel support from Celgene Corporation.

KY, RDT, CB, GJ, GP, MC, BD, DM, GC, HA, KR,SA, AC have no conflicting interests to declare. MC declares, Bristol Myers Squibb- employee, Honoraria/travel support in the last 3 years from, Amgen ,BMS/Celgene, Janssen, Takeda, Abbvie. CP declares BMS/Celgene Ad boards and speaker fees, Sanotif – Ad board, speaker fees, conference registration fees. JS reports Carrying out consultancy work (Advisory Board) for Sanofi. And an educational speaking engagement for Celgene/BMS RP declares; Honoraria – Jannsen, BMS, Abbvie, GSK. Consultancy: GSK, Janssen. Meeting support: Janssen, Takeda, BMS. RO declares- Janssen - advisory board, honoraria, Celegene – honoraria, Beigene - advisory board, honoraria, Astra Zeneca – honoraria. MK declares inter-relationships: AbbVie: consultancy; Amgen: honoraria; BMS/Celgene: consultancy, research funding (institution); GSK: consultancy; Janssen: consultancy, research funding (institution); Karyopharm: consultancy; Pfizer: consultancy; SeattleGenetics: consultancy; Takeda: consultancy; Sanofi: honoraria. MD reports owning stock in Abingdon Health. SQ is the founder and CSO of Achilles therapeutics a company developing T cell therapies for solid tumours.

For peer review only

References

1. Cancer Research UK. Myeloma Incidence Statistics 2015 [Available from: http://www.cancerresearchuk.org/health-professional/cancer-statistics/statistics-by-cancer-type/myeloma/incidence accessed 21st October 2018 2018.
2. Kristinsson SY, Anderson WF, Landgren O. Improved long-term survival in multiple myeloma up to the age of 80 years. *Leukemia* 2014;28(6):1346-8. doi: 10.1038/leu.2014.23
3. Child JA, Morgan GJ, Davies FE, et al. High-dose chemotherapy with hematopoietic stem-cell rescue for multiple myeloma. *N Engl J Med* 2003;348(19):1875-83. doi: 10.1056/NEJMoa022340
4. Attal M, Harousseau JL, Stoppa AM, et al. A prospective, randomized trial of autologous bone marrow transplantation and chemotherapy in multiple myeloma. Intergroupe Francais du Myelome. *N Engl J Med* 1996;335(2):91-7. doi: 10.1056/NEJM199607113350204
5. Attal M, Lauwers-Cances V, Hulin C, et al. Lenalidomide, Bortezomib, and Dexamethasone with Transplantation for Myeloma. *N Engl J Med* 2017;376(14):1311-20. doi: 10.1056/NEJMoa1611750
6. Weber DM, Chen C, Niesvizky R, et al. Lenalidomide plus dexamethasone for relapsed multiple myeloma in North America. *N Engl J Med* 2007;357(21):2133-42. doi: 10.1056/NEJMoa070596
7. McCarthy PL, Holstein SA, Petrucci MT, et al. Lenalidomide Maintenance After Autologous Stem-Cell Transplantation in Newly Diagnosed Multiple Myeloma: A Meta-Analysis. *Journal of clinical oncology : official journal of the American Society of Clinical Oncology* 2017;35(29):3279-89. doi: 10.1200/JCO.2017.72.6679
8. Delforge M, Minuk L, Eisenmann JC, et al. Health-related quality-of-life in patients with newly diagnosed multiple myeloma in the FIRST trial: lenalidomide plus low-dose dexamethasone versus melphalan, prednisone, thalidomide. *Haematologica* 2015;100(6):826-33. doi: 10.3324/haematol.2014.120121
9. Kumar S, Flinn I, Richardson PG, et al. Randomized, multicenter, phase 2 study (EVOLUTION) of combinations of bortezomib, dexamethasone, cyclophosphamide, and lenalidomide in previously untreated multiple myeloma. *Blood* 2012;119(19):4375-82. doi: 10.1182/blood-2011-11-395749
10. Sonneveld P, Avet-Loiseau H, Lonial S, et al. Treatment of multiple myeloma with high-risk cytogenetics: a consensus of the International Myeloma Working Group. *Blood* 2016;127(24):2955-62. doi: 10.1182/blood-2016-01-631200
11. Joseph NS, Gentili S, Kaufman JL, et al. High-risk Multiple Myeloma: Definition and Management. *Clin Lymphoma Myeloma Leuk* 2017;17S:S80-S87. doi: 10.1016/j.clml.2017.02.018
12. Chng WJ, Dispenzieri A, Chim CS, et al. IMWG consensus on risk stratification in multiple myeloma. *Leukemia* 2014;28(2):269-77. doi: 10.1038/leu.2013.247
13. Shah V, Sherborne AL, Walker BA, et al. Prediction of outcome in newly diagnosed myeloma: a meta-analysis of the molecular profiles of 1905 trial patients. *Leukemia* 2018;32(1):102-10. doi: 10.1038/leu.2017.179
14. Boyd KD, Ross FM, Chiecchio L, et al. A novel prognostic model in myeloma based on co-segregating adverse FISH lesions and the ISS: analysis of patients treated in the MRC Myeloma IX trial. *Leukemia* 2012;26(2):349-55. doi: 10.1038/leu.2011.204
15. Kuiper R, Broyl A, de Knegt Y, et al. A gene expression signature for high-risk multiple myeloma. *Leukemia* 2012;26(11):2406-13. doi: 10.1038/leu.2012.127
16. Palumbo A, Avet-Loiseau H, Oliva S, et al. Revised International Staging System for Multiple Myeloma: A Report From International Myeloma Working Group. *Journal of clinical oncology : official journal of the American Society of Clinical Oncology* 2015;33(26):2863-9. doi: 10.1200/JCO.2015.61.2267
17. Paiva B, Vidriales MB, Cervero J, et al. Multiparameter flow cytometric remission is the most relevant prognostic factor for multiple myeloma patients who undergo autologous stem cell transplantation. *Blood* 2008;112(10):4017-23. doi: 10.1182/blood-2008-05-159624

For peer review only - http://bmjopen.bmj.com/site/about/guidelines.xhtml

18. Rawstron AC, Child JA, de Tute RM, et al. Minimal residual disease assessed by multiparameter flow cytometry in multiple myeloma: impact on outcome in the Medical Research Council Myeloma IX Study. *Journal of clinical oncology : official journal of the American Society of Clinical Oncology* 2013;31(20):2540-7. doi: 10.1200/JCO.2012.46.2119
19. Paiva B, van Dongen JJM, Orfao A. New criteria for response assessment: role of minimal residual disease in multiple myeloma. *Blood* 2015;125(20):3059-68. doi: 10.1182/blood-2014-11-568907
20. Sidiqi MH, Aljama MA, Bin Riaz I, et al. Bortezomib, lenalidomide, and dexamethasone (VRd) followed by autologous stem cell transplant for multiple myeloma. *Blood Cancer Journal* 2018;8(11):106. doi: 10.1038/s41408-018-0147-7
21. Facon T, Kumar S, Plesner T, et al. Daratumumab plus Lenalidomide and Dexamethasone for Untreated Myeloma. *The New England journal of medicine* 2019;380(22):2104-15. doi: 10.1056/NEJMoa1817249
22. Attal M, Richardson PG, Rajkumar SV, et al. Isatuximab plus pomalidomide and low-dose dexamethasone versus pomalidomide and low-dose dexamethasone in patients with relapsed and refractory multiple myeloma (ICARIA-MM): a randomised, multicentre, open-label, phase 3 study. *The Lancet (British edition)* 2019;394(10214):2096-107. doi: 10.1016/S0140-6736(19)32556-5
23. Deckert J, Wetzel MC, Bartle LM, et al. SAR650984, a novel humanized CD38-targeting antibody, demonstrates potent antitumor activity in models of multiple myeloma and other CD38+ hematologic malignancies. *Clin Cancer Res* 2014;20(17):4574-83. doi: 10.1158/1078-0432.CCR-14-0695
24. Kumar S, Paiva B, Anderson KC, et al. International Myeloma Working Group consensus criteria for response and minimal residual disease assessment in multiple myeloma. *The Lancet Oncology* 2016;17(8):e328-46. doi: 10.1016/S1470-2045(16)30206-6
25. Lahuerta JJ, Paiva B, Vidriales MB, et al. Depth of Response in Multiple Myeloma: A Pooled Analysis of Three PETHEMA/GEM Clinical Trials. *Journal of clinical oncology : official journal of the American Society of Clinical Oncology* 2017;35(25):2900-10. doi: 10.1200/JCO.2016.69.2517
26. Munshi NC, Avet-Loiseau H, Rawstron AC, et al. Association of Minimal Residual Disease With Superior Survival Outcomes in Patients With Multiple Myeloma: A Meta-analysis. *JAMA Oncol* 2017;3(1):28-35. doi: 10.1001/jamaoncol.2016.3160
27. Avet-Loiseau H, Bahlis NJ, Chng WJ, et al. Ixazomib significantly prolongs progression-free survival in high-risk relapsed/refractory myeloma patients. *Blood* 2017;130(24):2610-18. doi: 10.1182/blood-2017-06-791228
28. Chan A-W, Tetzlaff JM, Gøtzsche PC, et al. SPIRIT 2013 explanation and elaboration: guidance for protocols of clinical trials. *BMJ (Online)* 2013;346(jan08 15):e7586-e86. doi: 10.1136/bmj.e7586
29. Blade J, Samson D, Reece D, et al. Criteria for evaluating disease response and progression in patients with multiple myeloma treated by high-dose therapy and haemopoietic stem cell transplantation. Myeloma Subcommittee of the EBMT. European Group for Blood and Marrow Transplant. *Br J Haematol* 1998;102(5):1115-23.
30. Durie BG, Harousseau JL, Miguel JS, et al. International uniform response criteria for multiple myeloma. *Leukemia* 2006;20(9):1467-73. doi: 10.1038/sj.leu.2404284
31. Rajkumar SV, Harousseau JL, Durie B, et al. Consensus recommendations for the uniform reporting of clinical trials: report of the International Myeloma Workshop Consensus Panel 1. *Blood* 2011;117(18):4691-5. doi: 10.1182/blood-2010-10-299487
32. Jackson GH, Davies FE, Pawlyn C, et al. Lenalidomide Is a Highly Effective Maintenance Therapy in Myeloma Patients of All Ages; Results of the Phase III Myeloma XI Study. *Blood* 2016;128(22):1143-43.
33. Cocks K, King MT, Velikova G, et al. Evidence-based guidelines for determination of sample size and interpretation of the European Organisation for the Research and Treatment of Cancer

For peer review only - http://bmjopen.bmj.com/site/about/guidelines.xhtml

Quality of Life Questionnaire Core 30. *Journal of clinical oncology : official journal of the American Society of Clinical Oncology* 2011;29(1):89-96. doi: 10.1200/JCO.2010.28.0107
34. Royle KL, Gregory WM, Cairns DA, et al. Quality of life during and following sequential treatment of previously untreated patients with multiple myeloma: findings of the Medical Research Council Myeloma IX randomised study. *Br J Haematol* 2018;182(6):816-29. doi: 10.1111/bjh.15459
35. Avet-Loiseau H, Corre J, Lauwers-Cances V, et al. Evaluation of Minimal Residual Disease (MRD) By Next Generation Sequencing (NGS) Is Highly Predictive of Progression Free Survival in the IFM/DFCI 2009 Trial. American Society of Haematology (57th Annual Meeting and Exposition). Orlando, FL, 2015.
36. Bratton DJ, Phillips PP, Parmar MK. A multi-arm multi-stage clinical trial design for binary outcomes with application to tuberculosis. *BMC Med Res Methodol* 2013;13:139. doi: 10.1186/1471-2288-13-139
37. Simon R, Wittes RE, Ellenberg SS. Randomized phase II clinical trials. *Cancer Treat Rep* 1985;69(12):1375-81.
38. A'Hern RP. Sample size tables for exact single-stage phase II designs. *Stat Med* 2001;20(6):859-66. doi: 10.1002/sim.721
39. Reeder CB, Reece DE, Kukreti V, et al. Once- versus twice-weekly bortezomib induction therapy with CyBorD in newly diagnosed multiple myeloma. *Blood* 2010;115(16):3416-7. doi: 10.1182/blood-2010-02-271676
40. Caro JJ, Briggs AH, Siebert U, et al. Modeling good research practices--overview: a report of the ISPOR-SMDM Modeling Good Research Practices Task Force-1. *Med Decis Making* 2012;32(5):667-77. doi: 10.1177/0272989X12454577
41. Guide to the methods of technology appraisal 2013. London: NICE 2013.
42. Dolan P, Roberts J. Modelling valuations for Eq-5d health states: an alternative model using differences in valuations. *Med Care* 2002;40(5):442-6.
43. Rowen D, Brazier J, Young T, et al. Deriving a preference-based measure for cancer using the EORTC QLQ-C30. *Value Health* 2011;14(5):721-31. doi: 10.1016/j.jval.2011.01.004

For peer review only

For peer review only - http://bmjopen.bmj.com/site/about/guidelines.xhtml

Figure Legends/ Captions

Figure 1: Flow chart of participant's timelines in the RADAR study

Figure 2: Schedule of local investigations

Figure 3: Central Investigations from diagnosis up to 100 days post-ASCT

Figure 4: Central Investigations for the R1, R2 and R3 pathways

For peer review only

For peer review only - http://bmjopen.bmj.com/site/about/guidelines.xhtml

Figure 1: Flowchart of Participants timelines in the RADAR Study

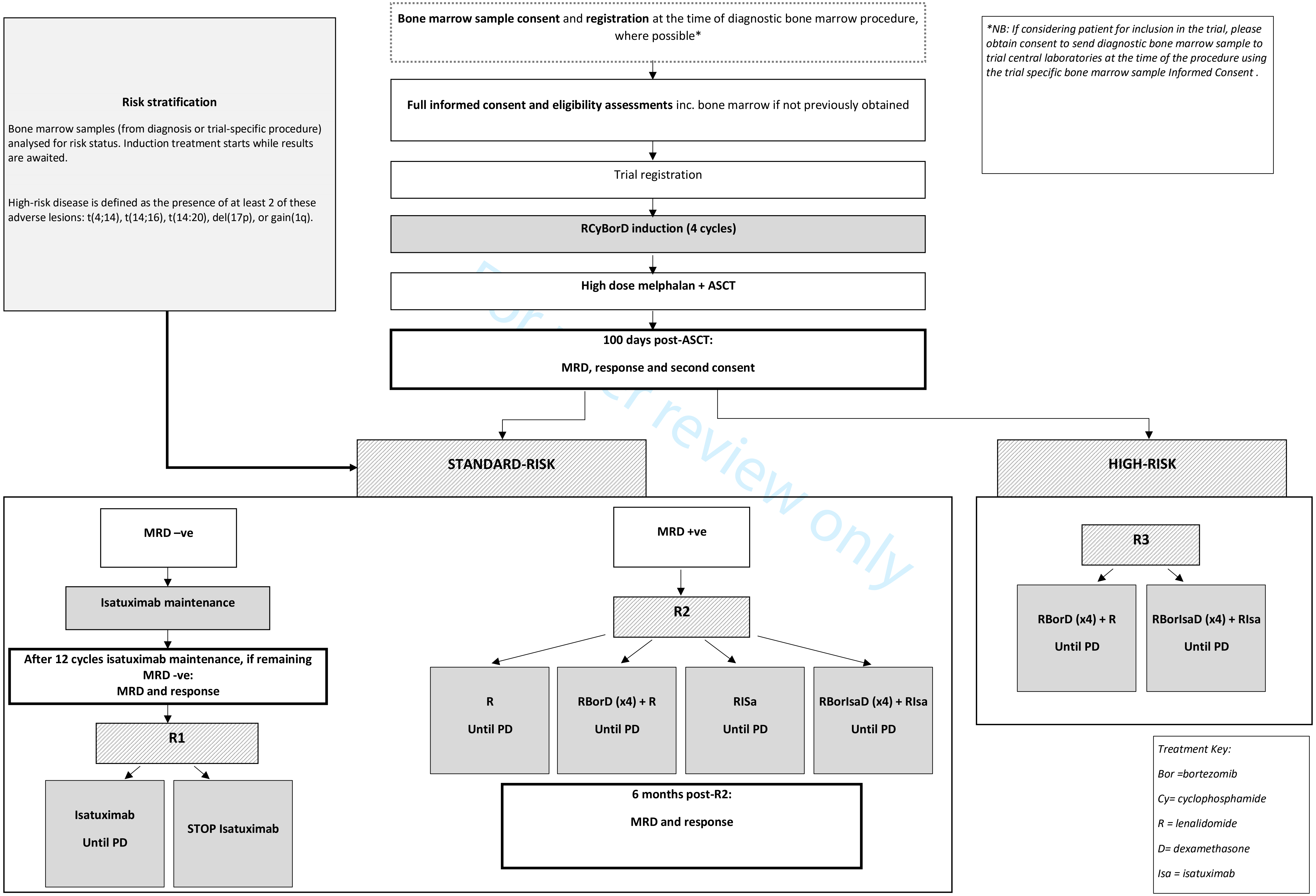

For peer review only

Figure 2: Schedule of Local Investigations

	RCyBorD Induction – All participants				R1 treatment pathway			R2 and R3 treatment pathway<br>R2 participants on R or RIsa arm: After 3,6,12 and 18 cycles**		
	**Pre-registration/baseline** (within 14 days prior to registration, unless otherwise stated)	**Day 1 (or day -1) of each cycle during RCyBorD induction††**	**End of RCyBorD induction**	**100 days post-ASCT** (within 14 days prior to R2, start of Isatuximab maintenance or R3)	**Day 1 of each cycle of isatuximab treatment††**	**On the day of isatuximab dose††**	**Follow up for participants coming off study during R1 treatment pathway: every 2 months**	**Day 1 of each cycle of treatment††**	**On day of (and prior to) every isatuximab dose††**	**Follow up for participants coming off study during R2/R3 treatment pathway: every 2 months**
Written informed consent	✓<br>Consent to be taken prior to all assessments, permitted outside the 14-day window			✓<br>(permitted outside the 14-day window)						
Physical examination	✓	✓	✓	✓	✓			✓		
ECOG	✓	✓	✓	✓	✓			✓		
Covid vaccination status	✓	✓	✓	✓	✓		✓	✓		✓
Medical history (including review for prior cancers and history of Covid infection)	✓		✓	✓						
Assessment of cardiac risk*	✓(results from formal measurements within 28 days prior to registration)	If clinically indicated	✓	If clinically indicated	If clinically indicated			If clinically indicated		
ECG					Every 3 months in participants receiving Isatuximab			Every 3 months in participants receiving Isatuximab		
Thyroid function monitoring*	✓(within 28 days prior to registration)	If clinically indicated	✓	If clinically indicated	If clinically indicated			If clinically indicated		
FBC, LFTs, albumin, LDH, U&Es, calcium, and creatinine¶	✓	✓	✓	✓	✓	✓ FBC, U&E and creatinine only		✓	✓ FBC, U&E and creatinine only	
Hepatitis B, (surface antigen and core antibody), ,hepatitis C and HIV	⍰(within 28 days prior to registration)									
β2-microglobulin (ß2M)	✓									
C-Reactive protein (CRP)	✓									
Calculated creatinine clearance (using Cockcroft-Gault formula)	✓	✓	✓	✓	✓			✓		
Serum paraprotein and immunofixation, serum free light chains and urinary free light chain detection	✓	✓	✓	✓	✓		✓ (if performed as part of standard care)	✓		✓ (If performed as standard of care)
Bone marrow (aspirate and trephine)<br>*See Table below for details of central investigations*	✓ (within 6 weeks prior to registration)	✓(recommended only if a first occurrence of CR or sCR is suspected)	✓	✓	✓(recommended only if a first occurrence of CR or sCR is suspected)		✓ (if performed as part of standard care)	✓ (recommended only if first occurrence of CR/sCR is suspected)		✓ (If performed as standard of care)
Pregnancy prevention counselling	✓	At least every 28 days during lenalidomide treatment								
Pregnancy test† (for females of childbearing potential)	✓	✓¥			✓			✓¥		
Cross-sectional imaging according to local practice, to include the thorax (low dose whole body CT recommended)	Within 3 months before registration§	If clinically indicated. ‡			If clinically indicated			If clinically indicated		
Assessment of adverse events (including adverse events and second primary malignancy)		Monitor throughout study and report on relevant treatment eCRFs (from first treatment dose until 60 days post last treatment dose)			Monitor throughout study and report on relevant treatment eCRFs (from first treatment dose until 60 days post last treatment dose) . AEs should continue to be monitored for participants randomised to 'stop Isatuximab' at R1 (and until 60 days after last on-study assessment).			Monitor throughout study and report on relevant treatment eCRFs (from first treatment dose until 60 days post last treatment dose)		

**Performed as part of standard of care.*
*** ECGs should be scheduled to align with other required face-to-face visits*
¶ The FBC should be repeated mid-cycle 1 (Day 14 +/-3 days) or more frequently and during subsequent cycles if there is a concern about cytopenias
† Females of childbearing potential (FCBP) must have a negative pregnancy test performed by a healthcare professional in accordance with the Celgene Lenalidomide Pregnancy Prevention Plan
†† May be performed within the 48 hours prior to the specified day
*** In addition to standard Day 1 assessments*
¥ All FCBP: The first pregnancy test must be performed within 10 to 14 days prior to the start of lenalidomide and the second pregnancy test must be performed within 24 hours prior to the start of lenalidomide.
FCBP with regular or no menstrual cycles: within 24 hours before start of lenalidomide treatment; weekly for the first 28 days of study participation; 4-weekly as a minimum during lenalidomide treatment; at study discontinuation; and 4 weeks after last dose of lenalidomide
FCBP with irregular menstrual cycles: within 24 hours before start of lenalidomide treatment; weekly for the first 28 days of study participation; 2-weekly as a minimum during lenalidomide treatment; at study discontinuation; and at 2 and 4 weeks after last dose of lenalidomide

N.B. During the COVID-19 pandemic, face-to-face assessments must be carried out at least at the beginning of cycles 1 and 2 and at the end of consolidation. Subsequently, participants must be seen by a clinician at least every 3 cycles (and more frequently if clinically indicated or there are concerns).

For peer review only

Figure 3: Central Investigations from diagnosis up to 100 days post-ASCT

Sample	Investigation	**Diagnosis**<br><br>**(if patient consented on NHS form and trial bone marrow Informed Consent Document)**[1]		**Baseline (post trial consent and before the start of treatment)**	**Day 1 of Cycle 1 RCyBorD induction**	**End of induction cycle 1**	**End of induction cycle 2**	**End of RCyBorD induction**	**100 days post-ASCT and prior to consolidation/ maintenance**
0.5-1 mL EDTA bone marrow aspirate<br><br>**SEND TO HMDS, LEEDS**	Minimal Residual Disease	✓	**Trial consent**<br><br>**Consented to Myeloma XV trial and central laboratory investigations**	✓<br><br>(if not sent previously)				✓	✓
10 mL clotted peripheral blood<br><br>Random urine sample<br><br>**SEND TO BIRMINGHAM**	Disease parameters (including paraproteins) for response assessments and covid-19 antibody response<br><br>M-protein intereference assay			✓	✓<br><br>**Only if baseline sample not within 14 days**	✓	✓	✓	✓
10 mL EDTA bone marrow aspirate<br><br>10 mL blood<br><br>**SEND TO UCL, LONDON**	Tumour DNA for neoantigens<br><br>cfDNA<br><br>Immune profiling	✓<br><br>Bone marrow only		✓<br><br>BMA (if not sent previously) & blood	✓<br><br>**Cycle 1 only**<br><br>10 mL blood (**only if baseline sample is not from within 14 days prior to cycle 1 day 1**)				✓
Bone marrow trephine (6 sections)<br><br>**SEND TO UCL, LONDON**	Immunostaining for immune function	✓		✓<br><br>(if not sent previously					✓

[1] Baseline bone marrow samples may be taken before trial consent, provided that patients have consented to these samples being taken using the standard NHS consent form. The patient must have provided written informed trial consent on the trial bone marrow Informed Consent Document before these samples can be sent to the central laboratories.

For peer review only

Figure 4: Central Investigations for the R1, R2 and R3 pathways

Sample	Investigation	Standard-risk MRD-negative participants (R1 pathway)	Standard-risk MRD-positive participants on R or RIsa arm (R2 pathway)			Standard-risk MRD-positive participants on RBorD+R or RBorIsaD+RIsa arm (R2 pathway)			High-risk participants (R3 pathway)			All participants
		After cycles 6, 12 and 24 of maintenance *(or at equivalent time-points if randomised to stop receiving Isa)*	After cycle 3	After cycles 6, 12 and 18	After cycle 24	After consolidation cycle 4	After maintenance cycles 3, 9 and 15	After maintenance cycle 21	After consolidation cycle 4	After maintenance cycles 3, 9 and 15	After maintenance cycle 21	Disease progression
**0.5-1 mL EDTA bone marrow aspirate**<br>SEND TO HMDS, LEEDS	Minimal Residual Disease	✓	✓	✓		✓	✓		✓	✓		✓
**10 mL clotted peripheral blood**<br>**Random urine sample**<br>SEND TO BIRMINGHAM	Disease parameters (including paraproteins) for response assessments and covid-19 antibody response<br>M-protein interference assay	✓	✓	✓	✓(blood only)	✓	✓	✓(blood only)	✓	✓	✓(blood only)	✓
**10 mL EDTA bone marrow aspirate**<br>**10 mL blood**<br>SEND TO UCL, LONDON	Tumour DNA for neoantigens<br>cfDNA<br>Immune profiling	✓ (only after 6 cycles)	✓			✓			✓			✓
**Bone marrow trephine**<br>SEND TO UCL, LONDON	Immuno-chemistry for immune function		✓			✓			✓			✓

For peer review only

RADAR: Protocol Paper Attachments

Contents

1 Glossary of Terms 2
2 List of Sites in the RADAR Trial 4
3 Spirit Checklist: Reporting checklist for protocol of a clinical trial. 5
4 Excluded Concomitant Medication: 11
5 Exploratory analysis 13
6 Subgroup analysis 14
7 Informed Consent Material 16
Bone Marrow Consent 16
Part A: Initial Treatment and Further Study Consent 19
Part B: Randomisation 1 Treatment Group 22
Part C: Randomisation 2 Treatment Group 24
Part D: Randomisation 3 Treatment Group 26
8 Central Sample Analysis 28
9 References 31

For peer review only

1 Glossary of Terms

AE	Adverse event
ALT	Alanine aminotransferase
ANC	Absolute neutrophil count
AR	Adverse reaction
ASCT	Autologous stem cell transplantation
β2M	Beta2-microglobulin
BD	Twice daily
CI	Chief Investigator
CR	Complete response
CRF	Case report form
CRUK	Cancer Research United Kingdom
CTCAE	Common Terminology Criteria for Adverse Events
CTRU	Clinical Trials Research Unit
Cy	Cyclophosphamide
D	Dexamethasone
DMEC	Data Monitoring and Ethics Committee
DSUR	Developmental Safety Update Report
ECOG	Eastern Cooperative Oncology Group
eCRF	Electronic Case Report Form
EORTC	European Organisation for Research and Treatment of Cancer
FISH	Fluorescence in situ hybridization test
FWER	Family-wise error rate
GCP	Good Clinical Practice
HDM	High-dose melphalan
HMDS	Haematological Malignancy Diagnostic Service
IMiD®	Immunomodulatory Drug
IMP	Investigational medicinal product
IMWG	International Myeloma Working Group
IPD	Individual participant data
Isa	Isatuximab
ISS	International Staging System
ITT	Intention to treat
LFT	Liver function test
MedDRA	Medical Dictionary for Regulatory Activities
mg	Milligram
MGUS	Monoclonal gammopathy of undetermined significance
MHRA	Medicines and Healthcare Products Regulatory Agency
MM	Multiple myeloma
MR	Minimal response
MRD	Minimal Residual Disease
NCRI	National Cancer Research Institute
NDMM	Newly diagnosed multiple myeloma
NICE	National Institute for Health and Care Excellence
OS	Overall survival
PD	Progressive disease
PFS	Progression-free survival

For peer review only

PFS2	Time to second progression
PI	Principal Investigator
PIS	Patient Information Sheet
PP	Paraprotein
PR	Partial response
PS	Performance status
QoL	Quality of life
R	Lenalidomide
R1-3	Randomisation 1-3
RCT	Randomised controlled trials
RDE	Remote Data Entry
REC	Research Ethics Committee
R-ISS	Revised International Staging System
RSI	Reference Safety Information
SC	Sub-cutaneous
SD	Stable disease
SAE	Serious adverse event
SAP	Statistical analysis plan
SAR	Serious adverse reaction
SmPC	Summary of Product Characteristics
SOP	Standard operating procedure
SPM	Secondary Primary Malignancy
SSOP	Study site operating procedure
SUSAR	Suspected unexpected serious adverse reaction
TE	Transplant eligible
TMG	Trial Management Group
TNE	Transplant non-eligible
TSC	Trial Steering Committee
U&Es	Urea & electrolytes
UK-MRA	United Kingdom Myeloma Research Alliance
ULN	Upper limit of normal
VGPR	Very good partial response

For peer review only

2 List of Sites in the RADAR Trial

Aberdeen Royal Infirmary	Royal Bournemouth Hospital	University Hospital Wishaw
Royal Sussex County Hospital	Poole General Hospital	St Richard's Hospital
Chesterfield Royal Hospital	Salisbury District Hospital	Worthing Hospital
Derriford Hospital	Sunderland Royal Hospital	The Christie
Kent & Canterbury Hospital, Canterbury	South Tyneside District Hospital	Cheltenham General Hospital
Queen Elizabeth the Queen Mother Hospital	Southmead Hospital Bristol	Gloucestershire Royal Hospital
William Harvey Hospital	St. Helens	The Clatterbridge Centre
Freeman Hospital	Whiston Hospital	
Queen Elizabeth Hospital Gateshead	St George's Hospital (Tooting)	
Hammersmith Hospital	Torbay Hospital	
Manchester Royal Infirmary	University College London Hospital	
Wythenshawe Hospital	Leicester Royal Infirmary	
Milton Keynes University Hospital	Worcestershire Royal Hospital	
Perth Royal Infirmary	Alexandra Hospital, Redditch	
Ninewells Hospital	York Hospital	
Northampton General Hospital	University Hospital of Wales, Cardiff	
Churchill Hospital	Stratford Hospital	
Horton General Hospital	Warwick Hospital	
Queen Alexandra Hospital, Portsmouth	New Cross Hospital	
Queen Elizabeth Hospital, Birmingham	University Hospital Coventry	
Raigmore Hospital, Inverness	University Hospital Monklands	
Rotherham General Hospital	University Hospital Hairmyres	

For peer review only

3 Spirit Checklist: Reporting checklist for protocol of a clinical trial.

Based on the SPIRIT guidelines.

Chan A-W, Tetzlaff JM, Gøtzsche PC, Altman DG, Mann H, Berlin J, Dickersin K, Hróbjartsson A, Schulz KF, Parulekar WR, Krleža-Jerić K, Laupacis A, Moher D. SPIRIT 2013 Explanation and Elaboration: Guidance for protocols of clinical trials. BMJ. 2013;346:e7586

		Reporting Item	Page Number
**Administrative information**			
Title	#1	Descriptive title identifying the study design, population, interventions, and, if applicable, trial acronym	1
Trial registration	#2a	Trial identifier and registry name. If not yet registered, name of intended registry	1,2,3
Trial registration: data set	#2b	All items from the World Health Organization Trial Registration Data Set	NA
Protocol version	#3	Date and version identifier	29
Funding	#4	Sources and types of financial, material, and other support	32
Roles and responsibilities: contributorship	#5a	Names, affiliations, and roles of protocol contributors	1, 31
Roles and responsibilities: sponsor contact information	#5b	Name and contact information for the trial sponsor	31,32
Roles and responsibilities: sponsor and funder	#5c	Role of study sponsor and funders, if any, in study design; collection, management, analysis, and interpretation of data; writing of the report; and the decision to submit the report for publication, including whether they will have ultimate authority over any of these activities	32
Roles and responsibilities: committees	#5d	Composition, roles, and responsibilities of the coordinating centre, steering committee, endpoint adjudication committee, data management team, and other individuals or groups overseeing the trial, if applicable (see Item 21a for data monitoring committee)	26,27
**Introduction**			
Background and rationale	#6a	Description of research question and justification for undertaking the trial, including summary of relevant	6,7

For peer review only

		studies (published and unpublished) examining benefits and harms for each intervention	
Background and rationale: choice of comparators	#6b	Explanation for choice of comparators	6,7
Objectives	#7	Specific objectives or hypotheses	7, 18
Trial design	#8	Description of trial design including type of trial (eg, parallel group, crossover, factorial, single group), allocation ratio, and framework (eg, superiority, equivalence, non-inferiority, exploratory)	8
**Methods: Participants, interventions, and outcomes**			
Study setting	#9	Description of study settings (eg, community clinic, academic hospital) and list of countries where data will be collected. Reference to where list of study sites can be obtained	8
Eligibility criteria	#10	Inclusion and exclusion criteria for participants. If applicable, eligibility criteria for study centres and individuals who will perform the interventions (eg, surgeons, psychotherapists)	8-14
Interventions: description	#11a	Interventions for each group with sufficient detail to allow replication, including how and when they will be administered	15-17
Interventions: modifications	#11b	Criteria for discontinuing or modifying allocated interventions for a given trial participant (eg, drug dose change in response to harms, participant request, or improving / worsening disease)	17
Interventions: adherence	#11c	Strategies to improve adherence to intervention protocols, and any procedures for monitoring adherence (eg, drug tablet return; laboratory tests)	17
Interventions: concomitant care	#11d	Relevant concomitant care and interventions that are permitted or prohibited during the trial	17
Outcomes	#12	Primary, secondary, and other outcomes, including the specific measurement variable (eg, systolic blood pressure), analysis metric (eg, change from baseline, final value, time to event), method of aggregation (eg, median, proportion), and time point for each outcome. Explanation of the clinical relevance of chosen efficacy and harm outcomes is strongly recommended	18

For peer review only

Participant timeline	#13	Time schedule of enrolment, interventions (including any run-ins and washouts), assessments, and visits for participants. A schematic diagram is highly recommended (see Figure)	18,19
Sample size	#14	Estimated number of participants needed to achieve study objectives and how it was determined, including clinical and statistical assumptions supporting any sample size calculations	20
Recruitment	#15	Strategies for achieving adequate participant enrolment to reach target sample size	23
**Methods: Assignment of interventions (for controlled trials)**			
Allocation: sequence generation	#16a	Method of generating the allocation sequence (eg, computer-generated random numbers), and list of any factors for stratification. To reduce predictability of a random sequence, details of any planned restriction (eg, blocking) should be provided in a separate document that is unavailable to those who enrol participants or assign interventions	23
Allocation concealment mechanism	#16b	Mechanism of implementing the allocation sequence (eg, central telephone; sequentially numbered, opaque, sealed envelopes), describing any steps to conceal the sequence until interventions are assigned	23
Allocation: implementation	#16c	Who will generate the allocation sequence, who will enrol participants, and who will assign participants to interventions	23
Blinding (masking)	#17a	Who will be blinded after assignment to interventions (eg, trial participants, care providers, outcome assessors, data analysts), and how	NA
Blinding (masking): emergency unblinding	#17b	If blinded, circumstances under which unblinding is permissible, and procedure for revealing a participant's allocated intervention during the trial	NA
**Methods: Data collection, management, and analysis**			
Data collection plan	#18a	Plans for assessment and collection of outcome, baseline, and other trial data, including any related processes to promote data quality (eg, duplicate measurements, training of assessors) and a description of study instruments (eg, questionnaires,	23

For peer review only

		laboratory tests) along with their reliability and validity, if known. Reference to where data collection forms can be found, if not in the protocol	
Data collection plan: retention	#18b	Plans to promote participant retention and complete follow-up, including list of any outcome data to be collected for participants who discontinue or deviate from intervention protocols	23
Data management	#19	Plans for data entry, coding, security, and storage, including any related processes to promote data quality (eg, double data entry; range checks for data values). Reference to where details of data management procedures can be found, if not in the protocol	23
Statistics: outcomes	#20a	Statistical methods for analysing primary and secondary outcomes. Reference to where other details of the statistical analysis plan can be found, if not in the protocol	18
Statistics: additional analyses	#20b	Methods for any additional analyses (eg, subgroup and adjusted analyses)	24
Statistics: analysis population and missing data	#20c	Definition of analysis population relating to protocol non-adherence (eg, as randomised analysis), and any statistical methods to handle missing data (eg, multiple imputation)	24
**Methods: Monitoring**			
Data monitoring: formal committee	#21a	Composition of data monitoring committee (DMC); summary of its role and reporting structure; statement of whether it is independent from the sponsor and competing interests; and reference to where further details about its charter can be found, if not in the protocol. Alternatively, an explanation of why a DMC is not needed	27
Data monitoring: interim analysis	#21b	Description of any interim analyses and stopping guidelines, including who will have access to these interim results and make the final decision to terminate the trial	27
Harms	#22	Plans for collecting, assessing, reporting, and managing solicited and spontaneously reported adverse events and other unintended effects of trial interventions or trial conduct	27
Auditing	#23	Frequency and procedures for auditing trial conduct, if any, and whether the process will be independent from investigators and the sponsor	29

For peer review only

Ethics and dissemination

Research ethics approval	#24	Plans for seeking research ethics committee / institutional review board (REC / IRB) approval	29
Protocol amendments	#25	Plans for communicating important protocol modifications (eg, changes to eligibility criteria, outcomes, analyses) to relevant parties (eg, investigators, REC / IRBs, trial participants, trial registries, journals, regulators)	29
Consent or assent	#26a	Who will obtain informed consent or assent from potential trial participants or authorised surrogates, and how (see Item 32)	30
Consent or assent: ancillary studies	#26b	Additional consent provisions for collection and use of participant data and biological specimens in ancillary studies, if applicable	30,31
Confidentiality	#27	How personal information about potential and enrolled participants will be collected, shared, and maintained in order to protect confidentiality before, during, and after the trial	30
Declaration of interests	#28	Financial and other competing interests for principal investigators for the overall trial and each study site	32
Data access	#29	Statement of who will have access to the final trial dataset, and disclosure of contractual agreements that limit such access for investigators	30
Ancillary and post trial care	#30	Provisions, if any, for ancillary and post-trial care, and for compensation to those who suffer harm from trial participation	32
Dissemination policy: trial results	#31a	Plans for investigators and sponsor to communicate trial results to participants, healthcare professionals, the public, and other relevant groups (eg, via publication, reporting in results databases, or other data sharing arrangements), including any publication restrictions	31
Dissemination policy: authorship	#31b	Authorship eligibility guidelines and any intended use of professional writers	31
Dissemination policy: reproducible research	#31c	Plans, if any, for granting public access to the full protocol, participant-level dataset, and statistical code	31

Appendices

Informed consent materials	#32	Model consent form and other related documentation given to participants and authorised surrogates	(Supplementary Material)

For peer review only

Biological specimens	#33	Plans for collection, laboratory evaluation, and storage of biological specimens for genetic or molecular analysis in the current trial and for future use in ancillary studies, if applicable	(Supplementary Material)

None The SPIRIT Explanation and Elaboration paper is distributed under the terms of the Creative Commons Attribution License CC-BY-NC. This checklist can be completed online using https://www.goodreports.org/, a tool made by the EQUATOR Network in collaboration with Penelope.ai

For peer review only

4 Excluded Concomitant Medication:

**Excluded concomitant Medication:**
1. Systemic treatment with any of the following metabolising enzyme inducers and inhibitors should be avoided, unless there is no appropriate alternative medication for the participant's use (if there were to be a drug-drug interaction with an inducer, cyclophosphamide and bortezomib metabolite exposure would be increased):<br>a. Strong CYP3A inducers: rifampin, rifapentine, rifabutin, carbamazepine, phenytoin, and phenobarbital<br>b. Strong CYP3A inhibitors<br><br>2. Excluded foods and dietary supplements include St. John's Wort, green tea and supplements containing green tea extracts<br><br>3. The following medicinal products and procedures are **prohibited** during this study:<br>a. Any antineoplastic treatment with activity against MM, other than study drugs<br>b. Platelet transfusions within 3 days prior to study drug dosing for any dosing day, or prior to randomisation in order to meet eligibility criteria.<br>c. Additional steroid therapy (not as trial IMP) (except before commencing trial treatment up to a maximum of 160mg dexamethasone equivalent as per inclusion criteria or steroids used to manage hypersensitivity to trial medication)<br>d. Radiotherapy for the treatment of symptoms related to disease progression.<br>e. Continuous use of clarithromycin (short courses of 5-10 days are permitted for treatment of infective episodes when indicated)<br>f. Hormone replacement therapy and erythropoietic agents and other agents that increase the risk of thrombosis (applicable during treatment with lenalidomide)

For peer review only

Quality of Life and Health Economics questionnaires

Timepoints for all participants up to 100 days post-ASCT

- Baseline
- End of induction
- 100 days post-ASCT

***R1 pathway participants**

- After cycles 6, 12, 24 and 36 (for participants randomised to 'stop isatuximab' at R1, the cycle 24 and 36 questionnaires should still be completed at equivalent timepoints)

***Timepoints for R2 and R3 pathway participants receiving R or RIsa**

- After cycles 6, 12 and 24 of maintenance

***Timepoints for R2 and R3 pathway participants receiving RBorD+R or RBorIsaD+RIsa**

- After cycles 3, 9 and 21 of maintenance.

**Note that participants who stop trial treatment should complete QoL at the equivalent time points (unless the participant wishes to withdraw consent for any further data collection)*

For peer review only

5 Exploratory analysis

Exploratory analysis will consider; the effect of post-ASCT consolidation regimens utilising combinations of lenalidomide, bortezomib, dexamethasone, and isatuximab on immune function in the bone marrow of participants following ASCT, using high dimensional characterisation of the immune microenvironment, immune subsets, phenotype and transcriptome; T cell receptor (TCR) repertoire in the bone marrow of participants following ASCT and consolidation and/or maintenance; correlation of baseline immune profile, TCR diversity and function with disease response, including MRD status and timing of disease relapse; ex-vivo functional studies with participant derived samples used to generate explants or immune-competent patient derived xenografts (icPDX); and to review infection and re-infection rates of COVID-19 in participants during induction, post-ASCT and during post-ASCT consolidation and maintenance treatment. All exploratory analysis to be conducted by the trial statisticians will be pre-specified in the statistical analysis plan.

For peer review only

6 Subgroup analysis

Subgroup analysis will be conducted to determine whether a selection of patient characteristics, haematology / local serological results, biochemistry results and cytogenetic/molecular results as well as response assessments (full list below) are prognostic of the endpoints; PFS, PFS2, OS, attainment of ≥VGPR and attainment of MRD negativity, as defined for all randomisations. Note that the subgroups listed under the Response subheading will not be analysed for the last two endpoints.

Patient Characteristics

- Age (years) at trial registration (≤65, >65)
- Sex (Male / Female)
- ECOG performance status at trial registration (0, 1, ≥2)
- ISS at trial registration (Stage I, Stage II, Stage III)
- R-ISS at trial registration (Stage I, Stage II, Stage III)

Haematology / Local Serological Results

- Haemoglobin concentration at trial registration (<100, ≥100 g/L)
- Platelets at trial registration (<150, ≥150 x10^9/L)
- White blood cells at trial registration (< LLN (Lower limit of normal), ≥LLN)
- Neutrophil count at trial registration (<LLN, ≥LLN)
- Lymphocyte count at trial registration (<LLN, ≥LLN)
- Plasma cells (%) in bone marrow at trial registration (<60%, ≥60%)

Biochemistry Results

- β-2 microglobulin concentration at trial registration (<3.5, 3.5–<5.5, ≥5.5 mg/L)
- Serum creatinine concentration at trial registration (<175, ≥175 µmol/L)
- Corrected serum calcium concentration at trial registration (<2.75, ≥2.75 mmol/L)
- Serum albumin at trial registration (<ULN (Upper limit of normal) , ≥ULN)
- Lactate dehydrogenase at trial registration (<ULN, ≥ULN)
- C-Reactive protein at trial registration (<ULN, ≥ULN)
- Serum bilirubin at trial registration (<ULN, ≥ULN)
- ALT/AST at trial registration (<ULN, ≥ULN)

Cytogenetic/molecular Results

- t(4,14) at trial registration (detected, not detected)
- t(14,16) at trial registration (detected, not detected)
- t(14,20) at trial registration (detected, not detected)
- del(17p) at trial registration (detected, not detected)
- 1q gain at trial registration (detected, not detected)
- Hyperdiploidy at trial registration (detected, not detected)
- The number of high-risk adverse lesions at trial registration (R1 and R2: 0, 1 unable to determine, R3: 2, ≥3, or unable to determine)

Response

For peer review only

- Response to RCyBorD induction (<VGPR, ≥ VGPR)
- Response at 100 days post-ASCT (<VGPR, ≥ VGPR)
- Response at the end of consolidation (<VGPR, ≥ VGPR)
- Response to 12m post-ASCT treatment (<VGPR, ≥ VGPR)
- MRD response to RCyBorD induction (R3 Only: MRD positive, MRD Negative)
- MRD at 100 days post-ASCT (R3 Only: MRD positive, MRD negative)
- MRD response to 6m post-ASCT treatment (R3 Only: MRD positive, MRD Negative)
- MRD response to 12m post-ASCT treatment (R3 Only MRD positive, MRD Negative)

Further subgroup analyses may be undertaken, as appropriate and all the subgroups will be fully defined within the statistical analysis plan according to the current criteria [1-3].

For peer review only

7 Informed Consent Material

Bone Marrow Consent

Delete this line, then print on Trust/Hospital headed paper

Participant ID:	Initials:
Date of Birth:	NHS or CHI number for Scotland/Hospital Number :
EudraCT Number:	Principal Investigator:

RADAR (UK-MRA Myeloma XV)

INFORMED CONSENT DOCUMENT FOR BONE MARROW AND BLOOD SAMPLE

Please initial each box

1. I confirm that I have read and understand the information sheet for the bone marrow sample request and have had the opportunity to ask questions. ☐
2. I understand that my providing bone marrow samples is voluntary and that I am free to withdraw at any time without my medical care or legal rights being affected. ☐
3. I understand that the samples will be sent along with information about me, which will include NHS number or CHI number for Scotland and may include my name. ☐
4. I agree to these samples being stored and used for research investigations that form part of the RADAR study, and understand that this will include genetic research (for example, analysis of DNA). ☐

For peer review only

5. I understand that a unique reference number will be allocated to the samples which may allow them to be linked back to me in future for research purposes. ☐

6. I understand that my data may be shared on a collaborative basis with researchers in the UK and potentially, centres abroad. ☐

7. I agree to allow any information provided to be used for healthcare and/or further medical research upon the understanding that my identity will remain anonymous wherever possible. ☐

8. I understand that results of my laboratory tests may be shared with other research teams if I am taking part in another clinical study being run by the University of Leeds. ☐

9. I agree to a copy of this Consent Form being sent to the Clinical Trials Research Unit. ☐

10. I agree for my details (which will include date of birth and NHS number or CHI number for Scotland) to be submitted to NHS Digital or other central UK NHS bodies so that information about my health, and mortality should this become available, may be obtained by the CTRU if necessary. ☐

11. I agree to provide bone marrow samples. ☐

Please indicate your wishes in the below scenarios:

Please initial

A) If I am diagnosed with a plasma cell dyscrasia, other than myeloma, I give permission for the samples sent to the central laboratories to be stored and used in future research that receives ethical approval. I understand that the samples and data collected from them may be shared with researchers, possibly in other countries. Yes ☐ No ☐

B) If I am diagnosed with myeloma but do not take part in the RADAR study, I give permission for the samples sent to the central laboratories to be stored and used in future research that receives ethical approval. I understand that the samples and data collected from them may be shared with researchers, possibly outside the EEA. Yes ☐ No ☐

Patient (to be completed by the patient):

Signature……

Name (block capitals)………………………………………………………………………………………….

Date…….

Investigator:

For peer review only

I have explained the request to the above named patient and he/she has indicated his/her willingness for samples of bone marrow to be sent to the RADAR laboratories.

Signature………

Name (block capitals)……………………………………………………………………………………………………….

Date……….

(If used)Translator:

Signature………

Name (block capitals)……………………………………………………………………………………………………….

Date……….

(1 copy for patient; 1 for the CTRU; 1 held in patient notes, original stored in Investigator Site File)

For peer review only

Part A: Initial Treatment and Further Study Consent

Delete this line, then print on Trust/Hospital headed paper

Participant ID:	Initials:
Date of Birth:	NHS or CHI number for Scotland/Hospital Number:
EudraCT Number:	Principal Investigator:

RADAR (UK-MRA Myeloma XV)

INFORMED CONSENT DOCUMENT PART A

Initial Treatment and Further Study Information

PLEASE INITIAL EACH BOX

12. I confirm that I have read and understand the Part A information document for the RADAR study and have had the opportunity to ask questions.

13. I understand that my participation in this study is voluntary and that I am free to withdraw at any time without my medical care or legal rights being affected. I understand that even if I withdraw from the above study, the data and samples collected from me will be used in analysing the results of the study, and in some cases further information about any unwanted effects of my treatment may need to be collected by the study team.

14. I understand that my healthcare records may be looked at by authorised individuals from the study team, regulatory bodies or Sponsor in order to check that the study is being carried out correctly.

15. I am aware that I am required to have samples taken and sent to laboratories and I understand that this will include genetic research (for example, analysis of DNA). I understand that the samples will be sent along with information about me, which will include NHS number, or CHI number for Scotland, and may include my name.

For peer review only

16. I understand that results or reports from imaging scans, blood tests or other investigations (including for additional cancers) may be sent to the Clinical Trials Research Unit for central review by the research team.

17. I agree to allow any information or results arising from this study to be used for healthcare and/or further medical research upon the understanding that my identity will remain anonymous wherever possible.

18. I understand that my data and results of my laboratory tests (blood, urine and bone marrow sample collections) may be shared with other research teams if I am taking part in another clinical study being run by the University of Leeds.

19. I agree for my details (which will include date of birth and NHS number or CHI number for Scotland) to be submitted to NHS Digital or other central UK NHS bodies so that information about my health may be obtained by the CTRU if necessary.

20. I understand that my GP, or any other doctor treating me, will be notified of my participation in this study.

21. I understand that some of my data may be passed to other organisations, such as the pharmaceutical companies Sanofi and Celgene, (possibly in other countries where the data protection standards and laws may be different from the UK) to monitor the safety of the treatment(s) that I am receiving. I understand that my identity will remain anonymous.

22. I agree to a copy of this Consent Form being sent to the Clinical Trials Research Unit.

23. I agree to take part in the study.

The following parts of the study are OPTIONAL (see section 3 of the Participant Information Sheet). Even if you agree to take part in the RADAR study, you do not have to agree to these optional parts. Please initial yes or no.

Please initial

A) I agree to take part in the Quality of Life and Healthcare Resource Use studies by completing questionnaires at timepoints throughout the study.

Yes No

B) I give permission for samples that I provide to be used in future research that receives ethical approval, and I understand that this includes the bone marrow samples provided at the time of my diagnosis (if I consented separately to collection of the diagnostic bone marrow sample in anticipation of participation in this study). I understand that my samples and data collected from them may be shared on a collaborative basis with researchers in the UK and, potentially, centres abroad.

Yes No

For peer review only

Patient (to be completed by the patient):

I agree to take part in this study.

Signature……….

Name (block capitals)……………………………………………………………………………………………….

Date………...

Investigator:

I have explained the study to the above named patient, and he/she has indicated his/her willingness to participate in this study.

Signature………

Name (block capitals)……………………………………………………………………………………………….

Date……….

(If used) Translator:

Signature………

Name (block capitals)……………………………………………………………………………………………….

Date……….

(1 copy for patient; 1 for the CTRU; 1 held in patient notes, original stored in Investigator Site File)

For peer review only

Part B: Randomisation 1 Treatment Group

Delete this line, then print on Trust/Hospital headed paper

Participant ID:	Initials:
Date of Birth:	NHS or CHI number for Scotland/Hospital Number:
EudraCT Number:	Principal Investigator:

RADAR (UK-MRA Myeloma XV)

INFORMED CONSENT DOCUMENT PART B

R1 Treatment Group

PLEASE INITIAL EACH BOX

24. I confirm that I have read and understand the Part B information document for the RADAR study and have had the opportunity to ask questions. ☐

25. I agree to continue my participation in the study as part of the R1 Treatment Group. ☐

Patient (to be completed by the patient):

I agree to take part in this study.

Signature…….

Name (block capitals)……………………………………………………………………………………………….

Date……..

Investigator:

I have explained the study to the above named patient, and he/she has indicated his/her willingness to participate in this study.

Signature…….

Name (block capitals)……………………………………………………………………………………………….

Date……..

For peer review only

(If used) Translator:

Signature………………………………………….………………………………………………………………

Name (block capitals)……………………………………………….……………………………………………….

Date…………………………………………………..…………………………………………………………………

(1 copy for patient; 1 for the CTRU; 1 held in patient notes, original stored in Investigator Site File)

For peer review only

Part C: Randomisation 2 Treatment Group

Delete this line, then print on Trust/Hospital headed paper

Participant ID:	Initials:
Date of Birth:	NHS or CHI number for Scotland/Hospital Number:
EudraCT Number:	Principal Investigator:

CANCER RESEARCH UK

RADAR (UK-MRA Myeloma XV)

INFORMED CONSENT DOCUMENT PART C

R2 Treatment Group

PLEASE INITIAL EACH BOX

26.I confirm that I have read and understand the Part C information sheet for the RADAR study and have had the opportunity to ask questions.

27.I agree to continue my participation in the study as part of the R2 Treatment Group.

Patient (to be completed by the patient):

I agree to take part in this study.

Signature……

Name (block capitals)……………………………………………………………………………………………….

Date…….

Investigator:

I have explained the study to the above named patient, and he/she has indicated his/her willingness to participate in this study.

Signature……

Name (block capitals)……………………………………………………………………………………………….

For peer review only

Date……………………………………………….……………………………………………………………………

(If used) Translator:

Signature…………………………………………….…………………………………………………………………

Name (block capitals)………………………………………….…………………………………………………….

Date……………………………………………….……………………………………………………………………

(1 copy for patient; 1 for the CTRU; 1 held in patient notes, original stored in Investigator Site File)

For peer review only

Part D: Randomisation 3 Treatment Group

Delete this line, then print on Trust/Hospital headed paper

Participant ID:	Initials:
Date of Birth:	NHS or CHI number for Scotland/Hospital Number:
EudraCT Number:	Principal Investigator:

CANCER RESEARCH UK

RADAR (UK-MRA Myeloma XV)

INFORMED CONSENT DOCUMENT PART D

R3 Treatment Group

PLEASE INITIAL EACH BOX

28. I confirm that I have read and understand the Part D information sheet for the RADAR study and have had the opportunity to ask questions. ☐

29. I agree to continue my participation in the study as part of the R3 Treatment Group. ☐

For peer review only

Patient (to be completed by the patient):

I agree to take part in this study.

Signature……

Name (block capitals)………………………………………………………………………………………………………

Date………

Investigator:

I have explained the study to the above named patient, and he/she has indicated his/her willingness to participate in this study.

Signature……

Name (block capitals)………………………………………………………………………………………………………

Date………

(If used) Translator:

Signature……

Name (block capitals)……………………………………………………………………………………………….

Date………

(1 copy for patient; 1 for the CTRU; 1 held in patient notes, original stored in Investigator Site File)

For peer review only

8 Central Sample Analysis

The trial has 3 central laboratories receiving samples for trial purposes. The time points and the reason for analysis for each central sample can be found in Figure 2. Details on the analysis of each sample is below. Note that participants can consent for their samples to be used in future research and hence each laboratory can store their trial samples for use in future studies. The storage details are also included below.

Birmingham

The Birmingham University Clinical Immunology Laboratory holds UKAS ISO 15189:2012 - Medical Laboratories accreditation. The Trial-related Laboratory Work will be performed using standard assays to the same quality standard as during routine clinical care. The Laboratory will be responsible for the analysis of whole clotted blood, serum and urine collected from participants of the Research Project in accordance with the Protocol. The laboratory will measure disease response and Disease progression, defined according to the IMWG 2016 response criteria for Multiple Myeloma [21 26-28]. This will include assessment of prognostic markers, markers of disease activity and M-protein levels. Analyses will include serum levels of beta-2 microglobulin, immunoglobulins IgG, IgA and IgM, kappa and lambda free immunoglobulin light chains. Serum electrophoresis and immunofixation, M-protein quantitation including total protein and densitometry. Urine immunofixation and free light chain quantitation will be performed where appropriate. As the trial proceeds new biomarkers of myeloma disease activity are likely to become available and will also be measured. The Laboratory will ensure that the Materials are received, handled, analysed, stored and destroyed in line with the individual consent provided.

HMDS

The presence of minimal residual disease will be assessed in bone marrow aspirates using a validated flow cytometry assay (sensitivity $\leqslant$0.001%) performed at a single central laboratory (HMDS, Leeds Teaching Hospitals Trust). A minimum of 2,500,000 cells will be evaluated with an eight-colour antibody combination of CD138, CD38, CD45, CD19, CD56, CD27, CD81 and CD117. Additional antigens for detection of CD38-negative plasma cells will be utilised as required. % neoplastic plasma cells and % normal plasma cells as a percentage of leucocytes will be reported. Limit of detection (LoD) and limit of quantification (LoQ) will also be calculated for each sample.
Samples will be used for trial purposes only and will not be stored for future research.

For peer review only

UCL

Bone marrow aspirates, peripheral blood and bone marrow trephine processing within UCL Myeloma Lab for RADAR clinical trial

OBJECTIVES

To provide molecular and genetic data on bone marrow immune profiles of newly diagnosed myeloma patients prior and post autologous stem cell transplantation. This will also be analysed between study arms to compare how different treatment regimes alert immune landscape in patients in particular anti-CD38 mAb Isatuximab.

METHODS

BMA and PB:

1. Sites are requested to collect samples and ship as per Lab Manual together with completed sample request form.
2. All samples will be processed in Myeloma Lab at UCL Cancer Institute.
3. UCL ECMC GCLP Facility will serve as storage facility for the following materials: PB plasma, BM serum, pellets (BM, PB, CD138+ and CD138-), protein lysates, extracted RNA and DNA. All material to be stored at -80˚C.
4. Myeloma Lab will serve as processing facility for all samples and storing of the following materials: BM MNCs (mononuclear cells), PB MNCs, CD138+ cells and CD138- cells. All material to be stored in liquid nitrogen.
5. Both -80˚C freezers and liquid nitrogen tanks are temperature monitored with systems that alert when temperature is out of acceptable range.
6. All samples and material extracted will be logged into the trial specific lab book and database with a unique trial ID, lab ID and time-point.
7. Upon arrival bone marrow aspirates and peripheral blood will be centrifuged. Serum (from BMA) and plasma (from PB) will be collected, aliquoted into 1-1.5ml aliquots and frozen at -80˚C.
8. BMA and PB mononuclear cells will then be isolated and enriched using Ficoll density gradient centrifugation.
9. PB MNCs will then be frozen as pellets and/or cryopreserved.
10. Depending on the tumour burden in the BM MNCs samples will be either frozen as MNCs pellets and/or cryopreserved or tumour cells will be isolated.

11. Tumour will be isolated via column-based MACS cell positive selection using CD138 magnetic beads (MicroBeads).

12. Depending on the number of cells isolated, CD138+ plasma cells will be frozen and stored as pellets and/or protein lysates and/or cryopreserved.

13. CD138- cells will be frozen and stored as pellets and/or cryopreserved.

Bone marrow trephine:

1. Sites are requested to collect, process and ship samples as per Lab Manual together with completed sample request form.

2. Myeloma lab will serve as storage facility for bone marrow trephine slides.

3. Upon arrival all samples will be logged into trial specific database with a unique trial ID, lab ID and time-point.

4. Upon arrival slides will be labelled and stored in the air-tight slide storage box.

ANALYSIS

1. High-dimensional flow cytometry will be used to profile immune context of patient bone marrow mononuclear cells prior and post-transplant.

2. Selected samples will be analysed further using transcriptomic profiling, T cell receptor (TCR) sequencing or single cell sequencing and multiplex immunofluorescence/immunohistochemistry on bone marrow trephine biopsies.

For peer review only

9 References

1. Oxman, A.D., *Subgroup analyses.* BMJ, 2012. **344**.
2. Sun, X., et al., *Is a subgroup effect believable? Updating criteria to evaluate* the *credibility of subgroup analyses.* BMJ, 2010. **340**.
3. Sun, X., et al., *Credibility of claims of subgroup effects in randomised controlled trials: systematic review.* BMJ, 2012. **344**.

For peer review only
